# Detailed Molecular Mechanism and Potential Drugs for COL1A1 in Carboplatin-Resistant Ovarian Cancer

**DOI:** 10.3389/fonc.2020.576565

**Published:** 2021-02-17

**Authors:** Feng Yang, Ziyu Zhao, Shaoyi Cai, Li Ling, Leying Hong, Liang Tao, Qin Wang

**Affiliations:** ^1^ Zhongshan School of Medicine, Sun Yat-Sen University, Guangzhou, China; ^2^ School of Pharmacy, Sun Yat-Sen University, Guangzhou, China

**Keywords:** carboplatin, ovarian cancer, ceRNA, KEGG, drugs, virtual screening

## Abstract

Carboplatin resistance in ovarian cancer (OV) is a major medical problem. Thus, there is an urgent need to find novel therapeutic targets to improve the prognosis of patients with carboplatin-resistant OV. Accumulating evidence indicates that the gene COL1A1 (collagen type I alpha 1 chain) has an important role in chemoresistance and could be a therapeutic target. However, there have been no reports about the role of COL1A1 in carboplatin-resistant OV. This study aimed to establish the detailed molecular mechanism of COL1A1 and predict potential drugs for its treatment. We found that COL1A1 had a pivotal role in carboplatin resistance in OV by weighted gene correlation network analysis and survival analysis. Moreover, we constructed a competing endogenous RNA network (LINC00052/SMCR5-miR-98-COL1A1) based on multi-omics data and experiments to explore the upstream regulatory mechanisms of COL1A1. Two key pathways involving COL1A1 in carboplatin resistance were identified by co-expression analysis and pathway enrichment: the “ECM-receptor interaction” and “focal adhesion” Kyoto Encyclopedia of Genes and Genomes pathways. Furthermore, combining these results with those of cell viability assays, we proposed that ZINC000085537017 and quercetin were potential drugs for COL1A1 based on virtual screening and the TCMSP database, respectively. These results might help to improve the outcome of OV in the future.

## Introduction

Ovarian cancer (OV) is the leading cause of death among women with gynecological malignancies and is characterized by high recurrence and mortality rates (1). Each year, 225,500 new cases of ovarian cancer are diagnosed, with 140,200 cancer-specific deaths worldwide (2). Owing to the use of chemotherapy, which is the mainstay of OV treatment, the mortality rate has decreased in recent decades (1). Chemotherapy, especially carboplatin, is the primary treatment for OV and can improve patients’ overall survival and quality of life (3). However, most OV patients receiving carboplatin chemotherapy develop chemoresistance, which leads to treatment failure (4).

Recently, many studies have demonstrated that the COL1A1 (collagen type I alpha 1 chain) gene is a potential therapeutic target with an important role in chemoresistance (5, 6). Most of these studies focused on the relationship between the expression of COL1A1 and chemoresistance. Some researchers found that the expression of COL1A1 was associated with resistance of OV to taxol (7), cisplatin (8), paclitaxel, doxorubicin, topotecan, vincristine, and methotrexate (5). However, the molecular mechanism by which COL1A1 participates in carboplatin-resistant OV has remained unclear; thus, the development of potential targeted therapeutic drugs is challenging.

In the present study, we performed data mining based on large-scale multi-omics data to explore the detailed molecular mechanism of COL1A1 in carboplatin-resistant OV and to identify potential drugs to target COL1A1. Our study provides new insight into the chemoresistance of OV at the molecular level and explores potential therapeutic drugs to overcome carboplatin resistance in OV and improve the outcomes of OV patients.

## Materials and Methods

### Data Collection

The gene expression count profile (transcriptome sequencing and microRNA [miRNA] profiles) and corresponding clinical data of OV patients were collected from The Cancer Genome Atlas (TCGA) database (accessed on September 21, 2019). Protein-coding genes and long non-coding RNAs (lncRNAs) were isolated from the transcriptome sequencing data. Based on their clinical data and previous study (9), 98 OV patients were categorized into a carboplatin-nonresistant group (complete response and partial response; n = 84) and a carboplatin-resistant group (stable disease and progressive disease; n = 14). Patients’ clinical characteristics are shown in **Table S1**. The use of TCGA data in the present study was in accordance with TCGA publication guidelines. As the patient data originated from the TCGA database, no further ethical approval was required.

### Study Design

**Figure 1:**
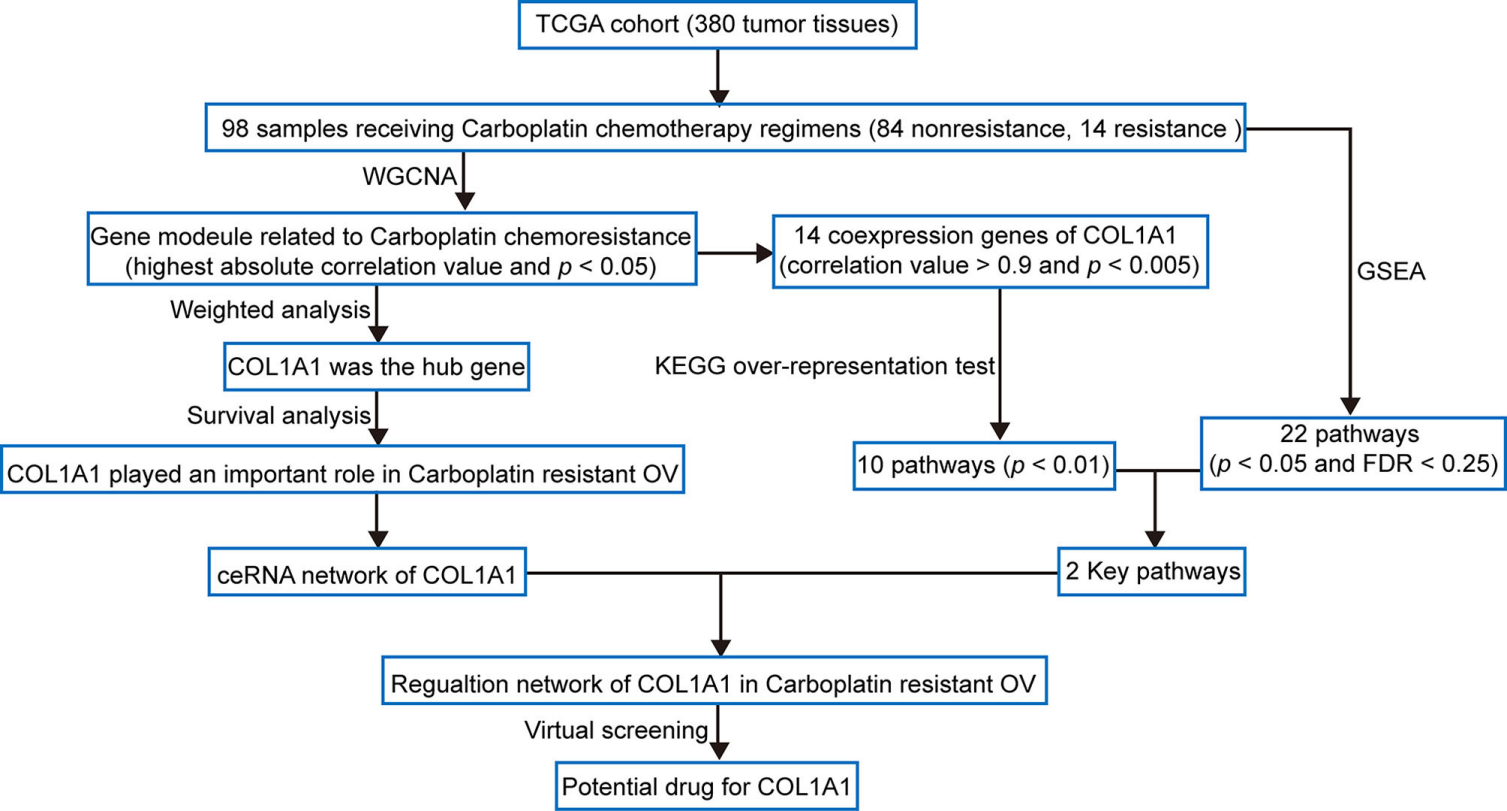
Flow diagram of the analysis procedure: data collection, preprocessing, analysis, and validation.

The workflow of this study is shown in **Figure 1**. In order to confirm the function of COL1A1 in carboplatin-resistant OV, we used weighted correlation network analysis (WGCNA), an unsupervised analysis method, to identify carboplatin-resistance-related genes. Then, we performed hub gene analysis and survival analysis to further validate the key role of COL1A1 in carboplatin-resistant OV. Subsequently, we explored the upstream regulatory mechanisms of COL1A1 by constructing a competing endogenous RNA (ceRNA) network. Moreover, we performed co-expression analysis and pathway enrichment to identify the downstream regulatory mechanisms of COL1A1. Finally, we identified candidates for drug-repurposing by virtual screening based on the structure of COL1A1 and the traditional Chinese medicines in the TCMSP database. Furthermore, we performed experiments to evaluate the results of the analysis.

### Identification of the Hub Genes in Carboplatin-Resistant OV

To obtain modules related to carboplatin resistance in OV, WGCNA was performed using R package WGCNA (10). A total of 8,791 genes with the top 70% median absolute deviations were screened from the database (n = 98) based on carboplatin response. Gene expression modules with similar patterns were identified by the dynamic tree cut method (minModuleSize = 50, mergeCutHeight = 0.25, and deepSplit = 1). An unsigned network type was used to retain relationships between modules and sample type (nonresistant or resistant). Modules with the highest absolute correlation values and *p* < 0.05 were considered to be significantly related to carboplatin resistance. Then, the identified carboplatin resistance genes were used to construct a network based on WGCNA. Subsequently, we employed two different methods to determine the hub gene in the carboplatin resistance module obtained by WGCNA. We assumed that a biological network G = (V, E) is an undirected network, where V is the collection of nodes within the network and E is the edge set. We used another notation, G = (V[G], E[G]), to represent a network, where V(G) is the collection of nodes in a network G, and E(G) is the collection of edges in a network G. For a set S, we used |S| to denote its cardinality (i.e., the number of elements in the set). Given a node v, N(v) denotes the collections of its neighbors. The two methods were as follows.

1. Degree method (Deg)

Deg(v)=|N(v)|

2. Maximum neighborhood component (MNC)

MNC(v)=|V(MC(v))|, where MC(v) is a maximum connected component of the G[N(v)] and G[N(v)] is the induced subgraph of G by N(v).

The gene ranked in first place by both methods was considered to be the hub gene in carboplatin-resistant OV. Finally, survival analysis was performed using KmPlot with an auto-selected best cutoff (11).

### Identification of Differentially Expressed mRNAs, miRNAs, and lncRNAs

We used the R package edgeR (12) to normalize and analyze significantly differentially expressed lncRNAs (DElncRNAs), miRNAs (DEmiRNAs), and mRNAs (DEmRNAs) between the carboplatin-resistant OV group (n = 14) and the carboplatin-nonresistant OV group (n = 84). According to the previous study (13), we wanted to obtain more candidates. So, the cutoff values were |log2 fold change| ≥ 0.4 and *p* < 0.05. The DElncRNAs, DEmiRNAs, and DEmRNAs were identified based on these thresholds.

### Construction of the ceRNA Network for COL1A1

We constructed a ceRNA network based on the DEmRNAs, DEmiRNAs, and DElncRNAs as described previously (14). To construct the mRNA (COL1A1)-related ceRNA network, interactions between COL1A1 and miRNAs were predicted using TargetScan (http://www.targetscan.org/vert_71/), and lncRNA–miRNA relationships were identified using miRcode (http://mircode.org/). According to the ceRNA hypothesis (15), lncRNAs act as miRNA sponges and negatively regulate miRNA-mediated gene silencing. The COL1A1-related ceRNA network was constructed and visualized using Cytoscape 3.7.1 (16).

### Co-Expression Analysis of COL1A1 and Pathway Enrichment Analysis

To explore the downstream regulatory mechanism of COL1A1, we performed co-expression analysis between COL1A1 and genes related to carboplatin resistance according to WGCNA, with cutoff values of Pearson correlation coefficient > 0.9 and *p* < 0.005. After obtaining the genes co-expressed with COL1A1, a KEGG over-representation test was performed using R package clusterProfiler (17) with a cutoff of *p* < 0.01. Gene set enrichment analysis (GSEA) (18) was also used to explore the potential molecular mechanisms in the carboplatin-resistant (n = 14) and carboplatin-nonresistant (n = 84) groups based on the expression profiles of all protein-coding genes, with cutoff values of false discovery rate (FDR) < 0.25 and *p* < 0.05. These results were combined to obtain the key pathways involving COL1A1 in carboplatin-resistant OV.

### Potential Drug-Repurposing and Traditional Chinese Medicine

The three-dimensional (3D) structure of COL1A1 was downloaded from the Protein Data Bank (PDB; 5CVB, https://www.rcsb.org/), and its binding sites were identified by Schrodinger Maestro (19). Then, we built a library of 2,106 US Food and Drug Administration (FDA)-approved drugs obtained from the ZINC15 database (20). Finally, we performed virtual screening and molecular docking with Schrodinger Maestro to identify potential drug-repurposing. We also used TCMSP (http://www.tcmspw.com/tcmsp.php) (21) to find traditional Chinese medicines that might target COL1A1.

### Chemicals

Carboplatin (≥ 98% purity, CAS: 41575-94-4), ZINC000085537017 (Cangrelor; ≥ 95% purity, CAS: 163706-36-3), and quercetin (≥ 98.5% purity, CAS: 117-39-5) were purchased from Aladdin (China). Stock solutions were prepared in dimethyl sulfoxide (≥99.7% purity, CAS 67-68-5, Sigma-Aldrich) and stored at 4°C. The fresh stock solution was made on a weekly basis. Other chemicals used in the study were analytic grade.

### Cell Culture

Pairs of parental and resistant SKOV3 and A2780 cell lines were provided by Soochow University. Cells were cultured in DMEM (Gibco, 12800017) supplemented with 10% fetal bovine serum (Gibco, 10270-106) containing penicillin (100 IU/ml) and streptomycin (100 μg/ml) (Gibco, 15140122) at 37°C in a 5% CO_2_ atmosphere.

### Real-Time Quantitative PCR

Total RNA was extracted using TRIzol reagent (Invitrogen). cDNA was synthesized using a cDNA synthesis kit (TIANGEN). Quantitative real-time PCR analysis was performed in triplicate with SYBR Green (TIANGEN) and specific primers (**Table S2**) using a CFX Connect Real-Time PCR Detection System (Bio-Rad, USA). U6 was used as an internal control for miRNAs. The relative expression levels of mRNAs or lncRNAs were evaluated relative to glyceraldehyde 3-phosphate dehydrogenase (GAPDH). Relative expression values were calculated using the 2^-△△Ct^ method.

### Cell Viability

Cell viability was detected by cell counting kit‐8 (CCK‐8; TongRen) assay following the manufacturer’s instructions. The CCK‐8 test solution was added 30 min before the end of treatment, and the absorbance was measured at 450 nm using a microplate reader. Carboplatin-resistant cells were exposed to a concentration gradient (0, 0.01, 0.1, 1, 10, and 100 μM) of ZINC000085537017 or quercetin for 24 h. To understand the influence of ZINC000085537017 and quercetin on sensitivity to carboplatin, resistant cells were pretreated with 1 μM ZINC000085537017 or 10 μM quercetin for 24 h, followed by incubation with 20% maximal inhibitory concentration (IC_20_) or IC_50_ doses of carboplatin for 48 h, and then subjected to cell viability assays.

### Statistical Analysis

Statistical analysis was performed using R 3.6.3 (R Foundation for Statistical Computing, Vienna, Austria). Normal distribution and homogeneity of variance tests were performed before the statistical analysis. The Wilcoxon test was used to evaluate the expression of COL1A1 between the carboplatin-resistant (n = 14) and carboplatin-nonresistant groups (n = 84), and t-tests were used to compare data between the two groups; *p* < 0.05 was considered statistically significant.

## Results

### COL1A1 Has an Important Role in Carboplatin-Resistant OV

**Figure 2:**
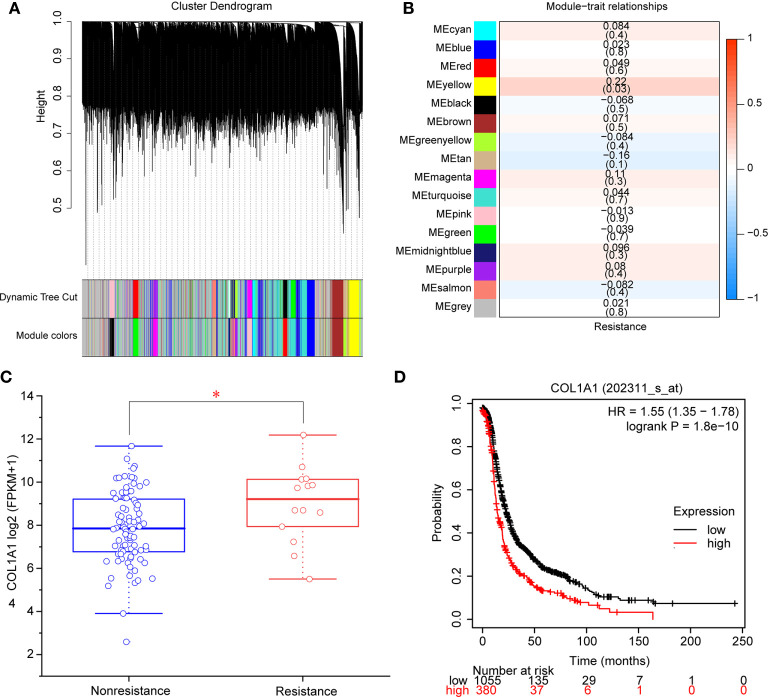
Identification of modules and genes associated with carboplatin-resistant OV. **(A)** Dendrogram of 8,791 genes in the top 70% median absolute deviations clustered based on a dissimilarity measure (1-TOM). **(B)** Heatmap of the correlation between module eigengenes and clinical traits. Each cell contains the correlation coefficient and *p*-value. **(C)** mRNA expression of COL1A1 in carboplatin-nonresistant OV (n = 84) and carboplatin-resistant OV (n = 14). *p < 0.05. **(D)** The overall survival of patients with high expression of COL1A1 was lower than that of patients with low expression (*p* < 0.05).

A total of 98 samples (84 nonresistant to carboplatin and 14 resistant to carboplatin) were included in WGCNA. We selected β = 6 as the appropriate soft-thresholding value to ensure a scale-free network, and 16 modules were identified. These modules are shown in distinct colors in **Figure 2A**. Then, the correlations between module eigengenes and the clinical trait of interest (resistance to carboplatin) were determined (**Figure 2B**, **Table S3**). The modules with the highest absolute correlation values and *p* < 0.05 were considered to be significant carboplatin-resistance-related modules. Based on the cutoffs used, the yellow module was screened as significantly related to carboplatin resistance in OV (**Figure 2B**). A total of 412 genes of the yellow module (**Table S4**) were found to be significantly related to carboplatin resistance by WGCNA. Subsequently, we constructed a network based on these 412 genes, then used the Deg and MNC methods to identify the hub gene involved in carboplatin-resistant OV. COL1A1 was ranked first place in the hub gene analysis by both methods (**Table S5**). We also found that COL1A1 mRNA was significantly overexpressed in the carboplatin-resistant OV group (*p* < 0.05, **Figure 2C**). We used overall survival analysis with KmPlot to further validate the role of COL1A1 in carboplatin resistance. The results showed that high mRNA expression of COL1A1 was associated with poor prognosis in OV (*p* < 0.05, **Figure 2D**). Taken together, these results showed that COL1A1 plays an important part in carboplatin resistance in OV.

### Constructing of the ceRNA Network of COL1A1

**Figure 3:**
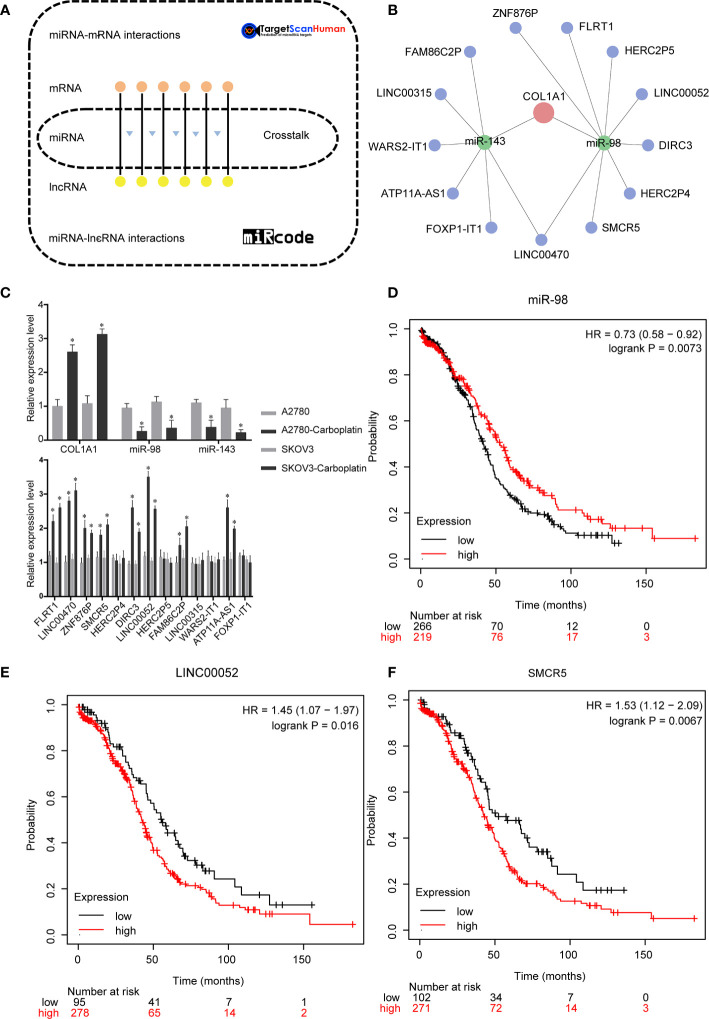
Upstream regulation mechanism of COL1A1 in carboplatin-resistant OV. **(A)** Bioinformatics methods were used to construct the ceRNA network of COL1A1. **(B)** ceRNA network of COL1A1. Red represents COL1A1, green represents miRNAs, blue represents lncRNAs. **(C)** Real-time PCR validation of candidates in ceRNA network. (mean ± SD, n =3). Asterisks indicate significant differences compared with the control group (*p < 0.05). Kaplan–Meier overall survival analyses for miR-98 **(D)**, LINC00052 **(E)**, and SMCR5 **(F)** in OV.

We performed bioinformatics analysis to explore the ceRNA network of COL1A1 in carboplatin-resistant OV (**Figure 3A**). A total of 845 DElncRNAs (**Table S6**), 96 DEmiRNAs (**Table S7**), and 1,684 DEmRNAs (**Table S8**) were identified according to the cutoff values (|log2 fold change| ≥ 0.4 and *p* < 0.05). Then, based on the ceRNA hypothesis and online databases (TargetScan and miRcode), the ceRNA network of COL1A1 was constructed. According to the results of Targetscan (**Table S9**), the conserved sites of miR-98 and miR-143 on 3′ untranslated region (3′-UTR) of COL1A1 were 789–795 and 152–158, respectively. To make the results more creditable, we also did anther analysis by miRsystem. According to the results from miRsystem (**Table S10**), there were 6 hits and 4 hits for miR-98 and miR-143, respectively. Finally, several lncRNAs (FAM86C2P, LINC00315, WARS2-IT1, ATP11A-AS1, FOXP1-IT1, LINC00470, SMCR5, HERC2P4, DIRC3, LINC00052, HERC2P5, FLRT1, and ZNF876P) sponged has-mir-143 and has-mir-98 to regulate COL1A1 (**Figure 3B**). As determined by real-time PCR (**Figure 3C**), the expression levels of COL1A1 mRNA, miRNAs (miR-143 and miR-98), and lncRNAs (FLRT1, LINC00470, ZNF876P, SMCR5, DIRC3, LINC00052, FAM86C2P, and ATP11A-IT1) were all significantly different between the carboplatin-sensitive and carboplatin-resistant cell lines (*p* < 0.05), which was in line with our preliminary expectations. There was no change in mRNA expression of lncRNAs (HERC2P4, HERC2P5, LINC00315, WARS2-IT1, and FOXP1-IT1) (**Figure 3C**), inconsistent with the analysis results. Subsequently, we performed KmPlot analysis to examine the relationships between these candidates and the prognosis of OV patients. Consistent with our expectations, overexpression of miR-98 was associated with better outcomes in OV (*p* < 0.05, **Figure 3D**). However, overexpression of miR-143 was related to poor prognosis (**Figure S1**). As shown in **Figures 3E** and **F**, overexpression of LINC00052 or SMCR5 was significantly related to poor outcomes in OV, which was consistent with our expectations. The remaining lncRNA candidates did not show relationships in accordance with our expectations (**Figure S1**). Taken together, these results suggest that the ceRNA network of COL1A1 is LINC00052/SMCR5-miR-98-COL1A1.

### Identification of the Downstream Regulatory Mechanism of COL1A1

**Figure 4:**
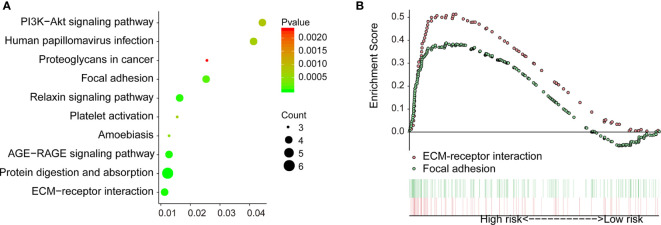
Downstream regulation mechanism of COL1A1 in carboplatin-resistant OV. **(A)** KEGG over-representation test pathway analysis of genes co-expressed with COL1A1 by clusterProfiler (*p* < 0.01). **(B)** Results of GSEA between carboplatin-resistant and nonresistant groups (*p* < 0.05 and FDR < 25%).

To explore the downstream mechanism of COL1A1 in carboplatin-resistant OV, we analyzed the genes co-expressed with COL1A1 among the 412 carboplatin-resistance-related genes. There were 14 genes co-expressed with COL1A1 (**Table S11**) according to the cutoff values of absolute Pearson correlation coefficient > 0.9 and *p* < 0.005. In addition, 11 KEGG pathways were enriched in the KEGG over-representation test based on the co-expressed genes of COL1A1 (**Figure 4A**). Another method for pathway analysis, GSEA targets the expression across the whole genome. GSEA analysis produced a total of five pathways (**Table S12**) in the carboplatin-resistant group and 22 pathways (**Table S13**) in the carboplatin-nonresistant group, using cutoff values of FDR < 25% and *p* < 0.05. Based on the KEGG over-representation test and GSEA results (**Figure 4B**), two overlapping pathways, “ECM receptor interaction” and “focal adhesion,” were identified.

### Potential Drug-Repurposing and Traditional Chinese Medicine

**Figure 5:**
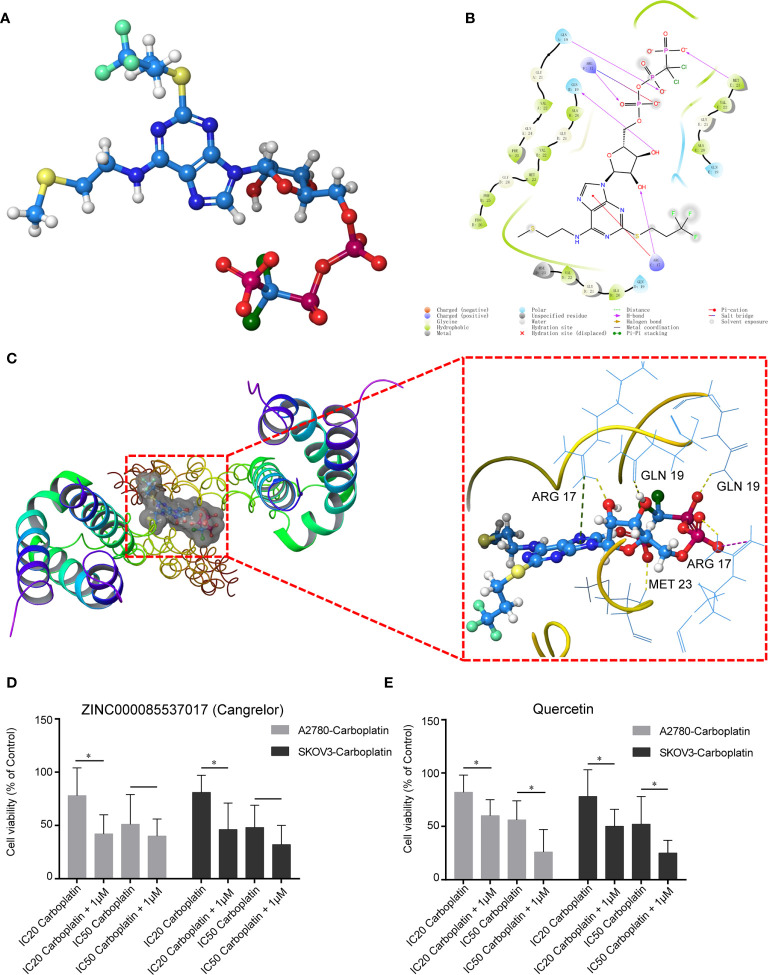
Potential drugs based on the structure of COL1A1. **(A)** Structure of ZINC000085537017. **(B)** 2D structure of ZINC000085537017’s binding mode in COL1A1. **(C)** 3D structure of ZINC000085537017’s binding mode in COL1A1. Yellow represents hydrogen bonds, green represents pi–pi interactions, and purple represents salt bridges. ZINC000085537017 **(D)** and quercetin **(E)** enhanced the cytotoxicity of carboplatin in resistant cells. Cells were pretreated with ZINC000085537017 or quercetin for 24 h, followed by incubation with carboplatin for 48 h, and were then subjected to cell viability assays. The results are presented as mean ± SD (n = 6) and were normalized to the control (*p < 0.05).

We used virtual screening with Schrodinger Maestro 2019-1 to identify potential drugs that could be repurposed to target COL1A1. The 3D protein structure of COL1A1 was downloaded from the PDB (5CVB, **Figure S2A**), and the active site was found by Schrodinger Maestro (**Figure S2B**). According to the glide scores (**Table S14**), ZINC000085537017 (Cangrelor) was the top hit from the structure-based virtual screening process. The 3D structure of ZINC000085537017 is shown in **Figure 5A**. There were six H-bonds and one pi–pi interaction in the ligand–protein complex (**Figure 5B**). The docking results for ZINC000085537017 and COL1A1 are shown in **Figure 5C**. Furthermore, we found that COL1A1 was targeted by quercetin based on the TCMSP database (**Table S15**). The docking results for quercetin and COL1A1 are shown in **Figure S2C**. To identify whether ZINC000085537017 or quercetin affected carboplatin sensitization in OV, first, carboplatin-resistant cell lines (A2780-carboplatin and SKOV3-carboplatin) were assayed for cell viability after treatment with a concentration gradient of ZINC000085537017 or quercetin (0, 0.01, 0.1, 1, 10, 100 μM) for 24 h. As shown in **Figures S3A–B and S3C–D**, 0.01–1.00 μM ZINC000085537017 and 0.01–10.0 μM quercetin did not significantly inhibit cell growth, but 10–100 μM ZINC000085537017 and 100 μM quercetin significantly reduced cell viability (*p* < 0.05). Then, based on previous research (22, 23), we selected 1 μM ZINC000085537017 and 10 μM quercetin for subsequent studies. Second, we calculated the IC_50_ values for carboplatin treatment for 48 h. The IC_50_ values of A2780, A2780-carboplatin, SKOV3, and SKOV3-carboplatin were 19.03 μM (7.066 μg/mL), 91.28 μM (33.89 μg/mL), 15.18 μM (5.635 μg/mL), and 68.96 μM (25.6 μg/mL), respectively. As shown in **Figure 5D**, 1 μM ZINC000085537017 significantly enhanced the carboplatin sensitivity (*p* < 0.05) of carboplatin-resistant cells treated with IC_20_ carboplatin, although it did not affect cell viability responses to IC_50_ carboplatin (**Figure 5D**). Similarly, 10 μM quercetin significantly enhanced the sensitivity of carboplatin-resistant cells to carboplatin (IC_20_ and IC_50_) after 48 h of treatment (*p* < 0.05, **Figure 5E**).

## Discussion

Carboplatin is the cornerstone of chemotherapy for OV. However, drug resistance to this agent continues to present challenges, leading to a poor prognosis for OV patients with a 5-year survival rate of only 25–30% (4, 24). Therefore, the mechanism of resistance to carboplatin in OV has become a focus of research in recent years. Increasing evidence has shown that COL1A1 has an important role in chemoresistance and could represent a potential therapeutic target (5, 6), but the mechanism of COL1A1 in carboplatin-resistant OV has remained unclear. In the present study, we determined the detailed molecular mechanism involving COL1A1 in carboplatin resistance and identified potential targeted drugs (both traditional Chinese medicine and FDA-approved drugs). These results provide new information and supporting data that could help to improve the outcomes of OV patients.

Many hub genes involved in carboplatin resistance have been identified by screening of differentially expressed genes (25–28). However, they were selected by an artificially set threshold, potentially excluding some important genes. In the present study, carboplatin resistance genes were screened by WGCNA, an unsupervised analysis method, making our results potentially more realistic and objective. We first screened carboplatin-resistance-related genes by WGCNA, then used hub gene analysis to identify COL1A1 as the hub gene. Furthermore, the expression of COL1A1 mRNA was found to be higher in carboplatin-resistant OV (*p* < 0.05), and survival analysis showed that high expression of COL1A1 mRNA was correlated with poor prognosis (*p* < 0.05), further demonstrating the pivotal role of COL1A1 in carboplatin resistance. The mRNA expression of COL1A1 was also found to be increased significantly (*p* < 0.05) in two carboplatin-resistant cells by real-time PCR. Although many studies have reported that the expression of COL1A1 was related to chemoresistance in OV (5–8), this was the first time that COL1A1 had been shown to play an important part in carboplatin-resistant OV. Furthermore, as type I collagen is composed of COL1A1 and COL1A2 (29), we speculated that COL1A2 might also have an important role in carboplatin-resistant OV, although there have been no reports about the role of COL1A2 in carboplatin resistance. According to the WGCNA and hub gene analysis results, COL1A2 was found in the 412 carboplatin resistance gene sets and was ranked 12th and 20th by Deg and MNC, respectively. Previously, Januchowski et al. reported that mRNA levels of COL1A2 and COL1A1 were significantly increased in OV cell lines resistant to cisplatin, paclitaxel, doxorubicin, topotecan, vincristine, and methotrexate (5). Taken together, these results suggest that COL1A1 and COL1A2 could be used as molecular targets for new antitumor drugs against carboplatin-resistant OV.

**Figure 6:**
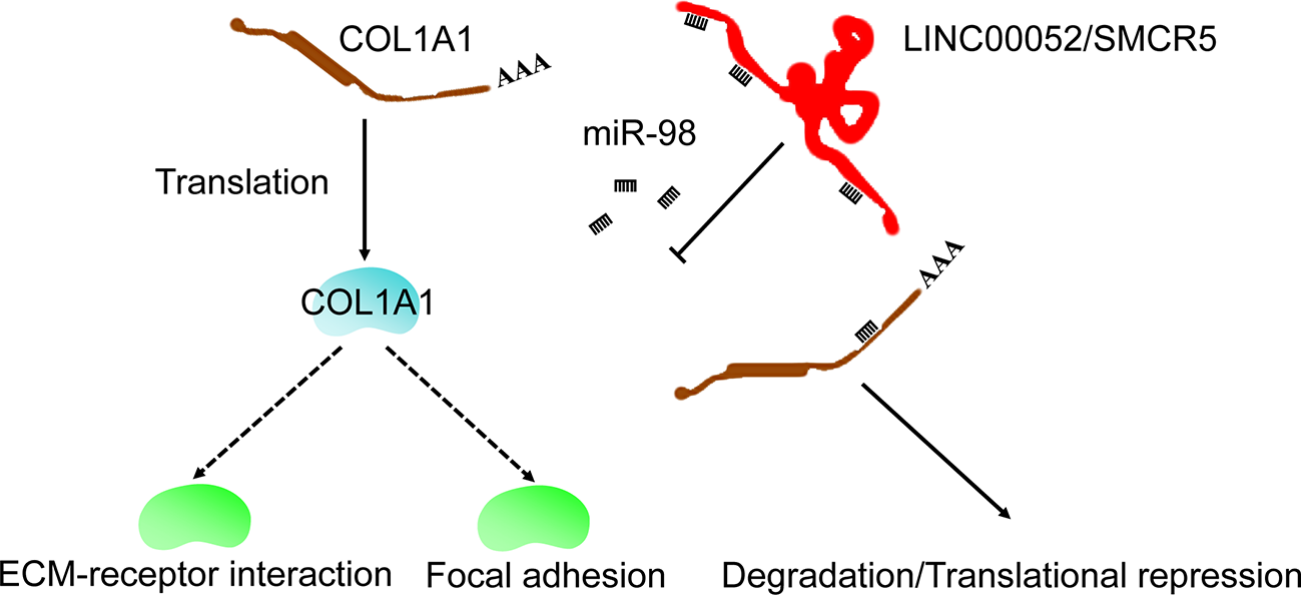
Possible mechanism of COL1A1 in carboplatin-resistant OV.

Emerging evidence indicates that ceRNA networks have an important role in chemoresistance to cancers (30, 31) and can provide therapeutic targets. In the present study, we used bioinformatics analysis to identify the ceRNA network of COL1A1. We also performed real-time PCR and KmPlot analysis to further confirm that the ceRNA network was LINC00052/SMCR5-miR-98-COL1A1 (**Figure 6**). Moreover, COL1A1 was previously found to be regulated by miR-98 in hypertrophic scarring (32) and muscular dystrophies (33); overexpression of miR-98 could increase cell apoptosis and enhance sensitivity to cisplatin in lung adenocarcinoma (34); and low miR-98 expression was correlated with temozolomide resistance of glioma (35). Although there have been no reports on the relationship between LINC00052/SMCR5 and chemoresistance, high expression of LINC00052 was found to promote gastric cancer cell proliferation and metastasis (36) and progression of head and neck squamous cell carcinoma (37). However, some of the experimental results in this study were inconsistent with our expectations. We propose two possible reasons for this. First, the associations observed between dysregulated expression of some candidates and prognosis of OV might not have been causal. Second, false positive results may have been generated in our analysis. Overall, in this study, we identified a ceRNA network (LINC00052/SMCR5-miR-98-COL1A1), which expanded our understanding of the upstream regulatory mechanism of COL1A1 in carboplatin-resistant OV and could provide therapeutic targets to improve the prognosis of OV.

Furthermore, the “ECM-receptor interaction” and “focal adhesion” KEGG pathways were identified as downstream pathways of COL1A1 involved in carboplatin-resistant OV. We identified these two key pathways using the KEGG over-representation test based on the co-expressed genes of COL1A1 and GSEA, suggesting that COL1A1 promoted carboplatin resistance in OV through these pathways. According to previous studies, the “ECM-receptor interaction” pathway is involved in platinum- (38), paclitaxel-, and topotecan-resistant OV (39), trastuzumab-resistant gastric cancer (40), and temozolomide-resistant glioblastoma (41). Moreover, to date, many drug resistance mechanisms involving the extracellular matrix have been identified across cancer types; these mechanisms have been classified into a range of categories including physical barriers to treatment (hypoxia, pH, and interstitial fluid pressure) and cell-adhesion-associated drug resistance (42). The “focal adhesion” KEGG pathway has been shown to be associated with taxol- (43) and cisplatin-resistant OV (44, 45). Taken together, these results indicated that COL1A1 was involved in carboplatin resistance in OV through the “ECM-receptor interaction” and “focal adhesion” KEGG pathways (**Figure 6**).

In the present study, we also explored potential drug-repurposing by virtual screening of FDA approved drugs and traditional Chinese medicines targeting COL1A1, which might expand potential therapeutic strategies for carboplatin-resistant OV treatment. We found that ZINC000085537017 and quercetin were potential drugs for treatment of COL1A1. Although 1 μM ZINC000085537017 did not affect cell viability in response to carboplatin, we expected that ZINC000085537017 and quercetin would enhance the sensitivity of carboplatin-resistant cells based on the cell viability assays. We speculated that the leaching toxicity of IC_50_ carboplatin in resistant cells might have exceeded the influence of 1 μM ZINC000085537017, resulting in no significant differences being found when IC_50_ carboplatin was combined with 1 μM ZINC000085537017 in these cells. Previous study showed that quercetin could increase the sensitivity of OV to cisplatin (46); however, in contrast to these studies, which focused on the relationship between the expression of COL1A1 and chemoresistance, we not only showed that COL1A1 was a therapeutic target but also identified some potential drugs. These results could help accelerate the development of drugs to improve the outcomes of carboplatin-resistant OV patients.

## Conclusion

In summary, we identified that COL1A1 has an important role in carboplatin-resistant OV by WGCNA; this result was further validated by survival analysis. Then, we constructed a ceRNA network for COL1A1 by bioinformatics analysis and experiments to expand understanding of the upstream regulatory mechanism of COL1A1 in carboplatin-resistant OV and identify potential therapeutic targets that could be used to improve the prognosis of OV. Moreover, we found that COL1A1 participated in carboplatin resistance in OV through the “ECM-receptor interaction” and “focal adhesion” KEGG pathways by co-expression analysis and pathway enrichment. Furthermore, combining these results with those of experiments, we found that ZINC000085537017 and quercetin were potential drugs for COL1A1 by virtual screening based on the structure of COL1A1 and the TCMSP database. These findings could accelerate drug development to improve the outcomes of carboplatin-resistant OV patients.

## Data Availability Statement

The original contributions presented in the study are included in the article/**Supplementary Material**, further inquiries can be directed to the corresponding authors.

## Author Contributions

LT and QW conceptualized and developed an outline for the manuscript and revised the manuscript. FY and ZZ conceived, designed, analyzed the data, and wrote the manuscript. LL and LH generated the figures and tables. SC collected the public data. All authors contributed to the article and approved the submitted version.

## Funding

This work was supported by the National Natural Science Foundation of China (Grant No. 81473234), the Joint Fund of the National Natural Science Foundation of China (Grant No. U1303221), the Fundamental Research Funds for the Central Universities (Grant No.16ykjc01), and the grant from Department of Science and Technology of Guangdong Province (Grant No.20160908).

## Conflict of Interest

The authors declare that the research was conducted in the absence of any commercial or financial relationships that could be construed as a potential conflict of interest.

## Abbreviations

COL1A1, Collagen Type I Alpha 1 chain; TCGA, The Cancer Genome Atlas; OV, Ovarian cancer; KEGG, Kyoto Encyclopedia of Genes and Genomes; WGCNA, Weighted gene correlation network analysis; GSEA, Gene Set Enrichment Analysis; ceRNA, Competing endogenous RNA; FDR, False discovery rate.
