# Supplementary material for: Detailed Molecular Mechanism and Potential Drugs for COL1A1 in Carboplatin-Resistant Ovarian Cancer
Source: Front Oncol. 2021 Feb 17;10:576565. doi: 10.3389/fonc.2020.576565 (PMC7928381; doi:10.3389/fonc.2020.576565)
Supplement: Supplementary file 1 [file DataSheet_1.docx]

**SUPLEMENTAL DATA**

**The detailed molecular mechanism and potential drug of COL1A1 in Carboplatin resistant Ovarian cancer**

Feng Yang^1^, Ziyu Zhao^1^, Shaoyi Cai^1^, Li Ling^1,2^, Leying Hong^1^, Liang Tao^1*^, Qin Wang^1*^

*1. Zhongshan School of Medicine, Sun Yat-Sen University, Guangzhou, 510080, China.*

*2. School of Pharmacy, Sun Yat-Sen University, Guangzhou, 510006, China.*

**Postal address:** NO. 74, Zhongshan Second Road, Guangzhou 510080, China

*Correspondence author:

Liang Tao, PhD, Zhongshan School of Medicine, Sun Yat-Sen University, Guangzhou, 510080,

China. E-mail: taol@mail.sysu.edu.cn.

Qin Wang, PhD, Zhongshan School of Medicine, Sun Yat-Sen University, Guangzhou, 510080,

China. E-mail: wangqin6@mail.sysu.edu.cn.

**Supplementary Figures:**

**
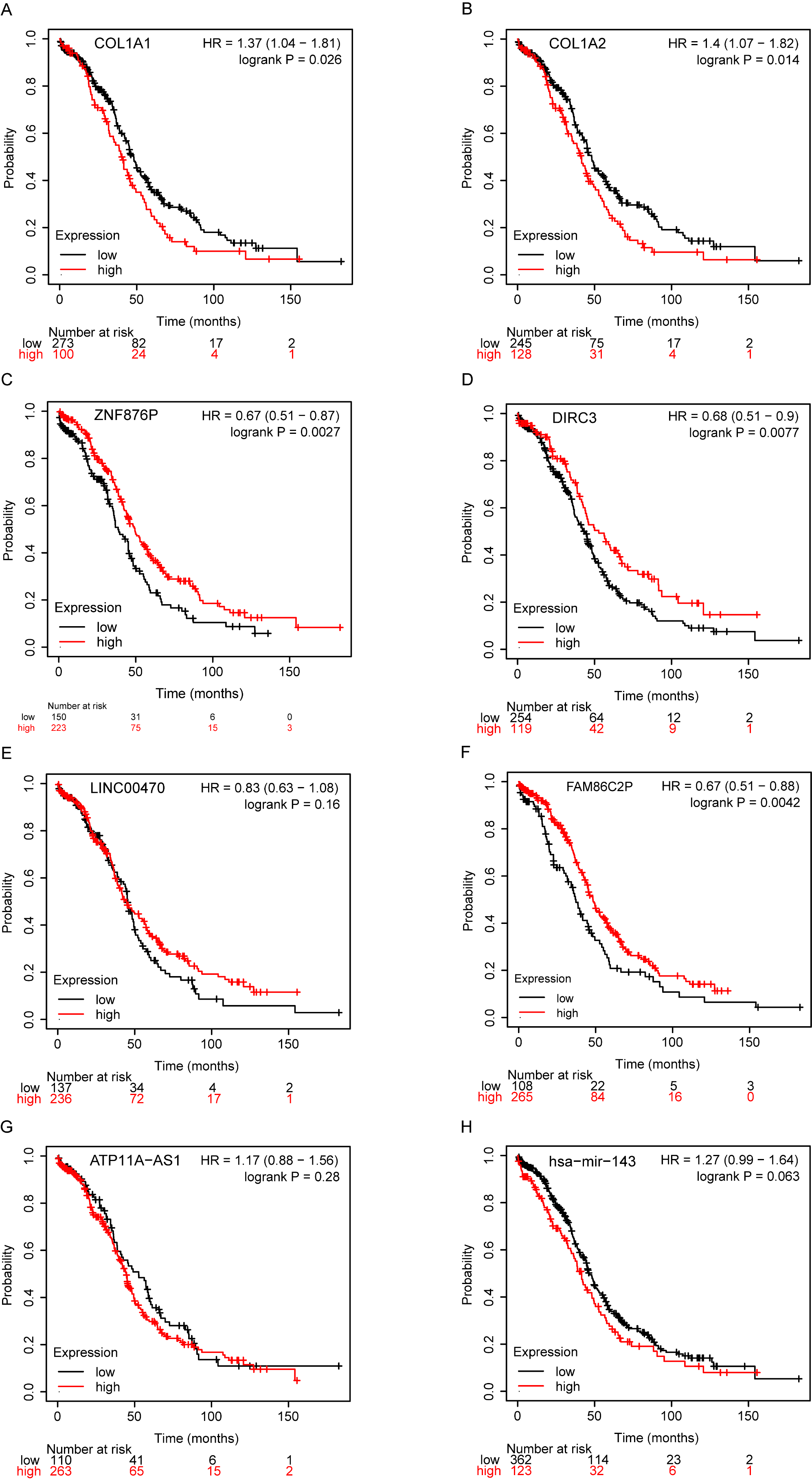
**

**Figure S1.** KM plot analysis of candidates in ceRNA network.

**
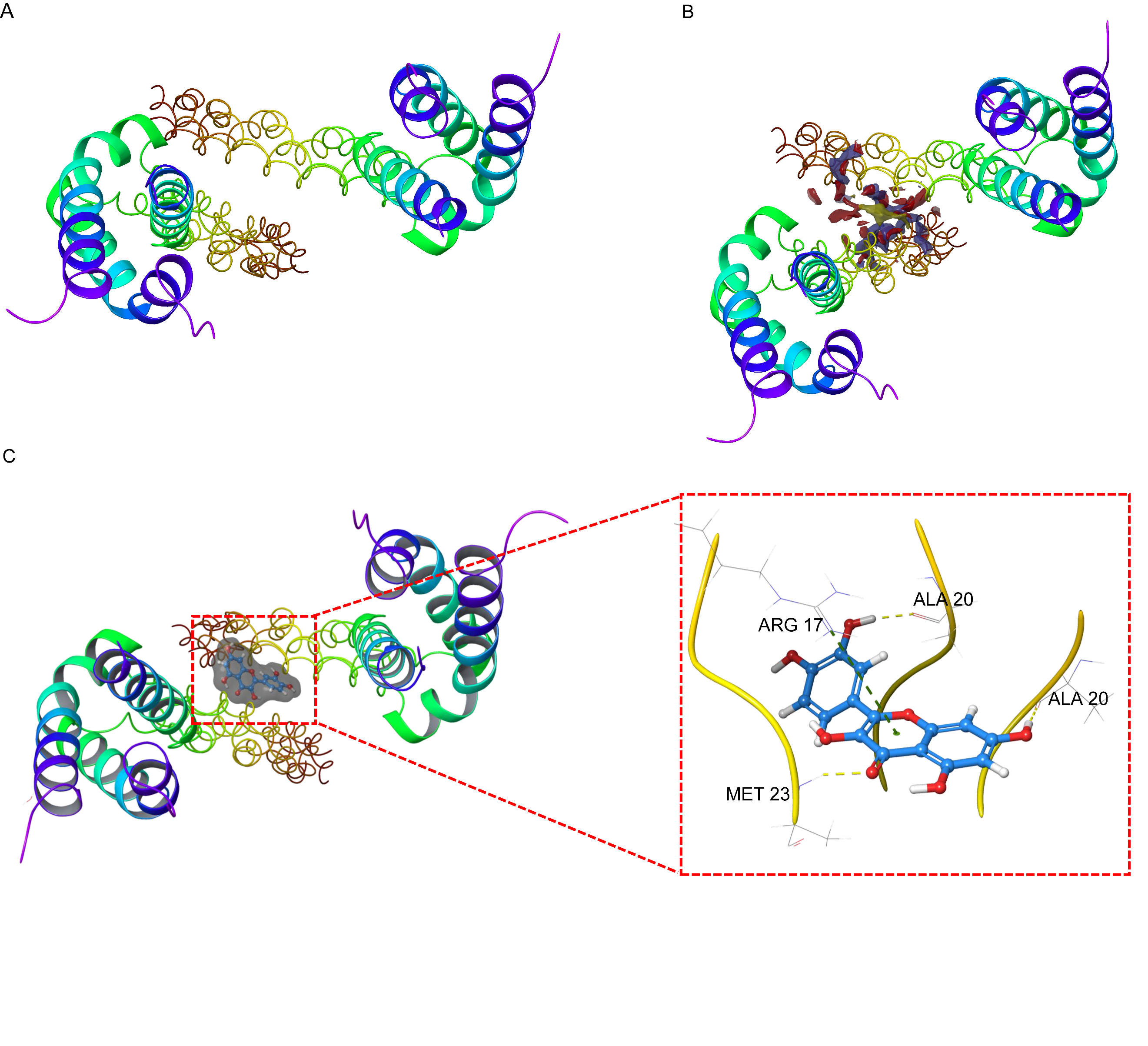
**

**Figure S2**. The structure of COL1A1. **(A)** The 3D structure of COL1A1. **(B)** The active site of COL1A1. **(C)** The docking complex of quercetin and COL1A1.

**
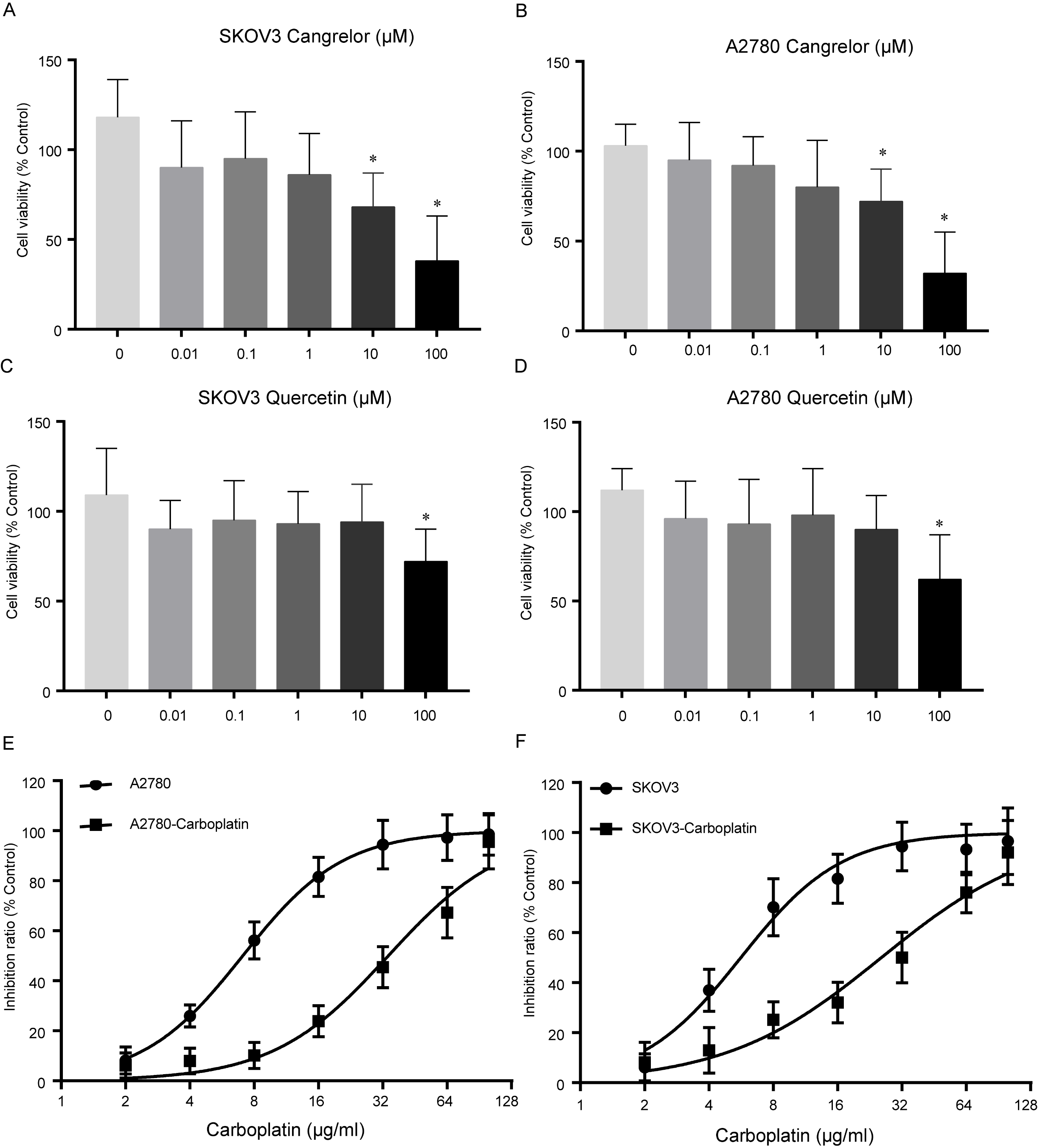
**

**Figure S3.** The cytotoxicity of compounds (Quercetin, Cangrelor and Carboplatin).

SKOV3-Caboplatin cells **(A)** or A2780-Carboplatin cells **(B)** treated with Cangrelor for 24 h and subjected to CCK-8 assay. SKOV3-Caboplatin cells **(C)** or A2780-Carboplatin cells **(D)** treated with Quercetin for 24 h and subjected to CCK-8 assay. Evaluating the Carboplatin index of A2780 or SKOV3. A2780 **(E)** or SKOV3 **(F)** were treated with Carboplatin for 48 h and subjected to CCk-8 assay. The values represented as mean ± SD are normalized to control (n = 6, * *p* < 0.05)

**Supplementary tables:**

**Table S1**. The clinical data of Carboplatin resistant OV in TCGA database.

| Id | Groups |
| --- | --- |
| TCGA-24-2281-01A | nonresistance |
| TCGA-24-2293-01A | nonresistance |
| TCGA-13-0911-01A | nonresistance |
| TCGA-24-2297-01A | nonresistance |
| TCGA-24-1413-01A | nonresistance |
| TCGA-25-2398-01A | nonresistance |
| TCGA-24-1103-01A | nonresistance |
| TCGA-31-1944-01A | nonresistance |
| TCGA-13-0725-01A | nonresistance |
| TCGA-04-1365-01A | nonresistance |
| TCGA-29-1785-01A | nonresistance |
| TCGA-13-0900-01B | nonresistance |
| TCGA-25-2393-01A | nonresistance |
| TCGA-10-0933-01A | nonresistance |
| TCGA-25-2404-01A | nonresistance |
| TCGA-13-1509-01A | nonresistance |
| TCGA-20-1682-01A | nonresistance |
| TCGA-36-1574-01A | nonresistance |
| TCGA-61-2104-01A | nonresistance |
| TCGA-OY-A56Q-01A | nonresistance |
| TCGA-09-1667-01C | nonresistance |
| TCGA-25-2409-01A | nonresistance |
| TCGA-25-1623-01A | nonresistance |
| TCGA-20-1687-01A | nonresistance |
| TCGA-13-0795-01A | nonresistance |
| TCGA-25-1328-01A | nonresistance |
| TCGA-13-0891-01A | nonresistance |
| TCGA-24-2023-01A | nonresistance |
| TCGA-61-2098-01A | nonresistance |
| TCGA-13-1506-01A | nonresistance |
| TCGA-29-2425-01A | nonresistance |
| TCGA-25-1318-01A | nonresistance |
| TCGA-24-0982-01A | nonresistance |
| TCGA-24-0979-01A | nonresistance |
| TCGA-13-1487-01A | nonresistance |
| TCGA-23-2077-01A | nonresistance |
| TCGA-24-2020-01A | nonresistance |
| TCGA-13-0913-02A | nonresistance |
| TCGA-24-1105-01A | nonresistance |
| TCGA-24-1418-01A | nonresistance |
| TCGA-13-0727-01A | nonresistance |
| TCGA-36-1577-01A | nonresistance |
| TCGA-24-1416-01A | nonresistance |
| TCGA-13-0797-01A | nonresistance |
| TCGA-23-2078-01A | nonresistance |
| TCGA-24-1616-01A | nonresistance |
| TCGA-25-1634-01A | nonresistance |
| TCGA-24-0970-01B | nonresistance |
| TCGA-61-2008-01A | nonresistance |
| TCGA-61-2008-02A | nonresistance |
| TCGA-29-1691-01A | nonresistance |
| TCGA-23-1119-01A | nonresistance |
| TCGA-13-1489-01A | nonresistance |
| TCGA-13-1489-02A | nonresistance |
| TCGA-09-1668-01B | nonresistance |
| TCGA-24-1842-01A | nonresistance |
| TCGA-24-1557-01A | nonresistance |
| TCGA-20-0991-01A | nonresistance |
| TCGA-10-0927-01A | nonresistance |
| TCGA-13-0726-01A | nonresistance |
| TCGA-5X-AA5U-01A | nonresistance |
| TCGA-24-1104-01A | nonresistance |
| TCGA-61-1910-01A | nonresistance |
| TCGA-24-1549-01A | nonresistance |
| TCGA-25-2400-01A | nonresistance |
| TCGA-61-2097-01A | nonresistance |
| TCGA-36-1581-01A | nonresistance |
| TCGA-25-1635-01A | nonresistance |
| TCGA-04-1347-01A | nonresistance |
| TCGA-24-1417-01A | nonresistance |
| TCGA-13-1405-01A | nonresistance |
| TCGA-23-1122-01A | nonresistance |
| TCGA-09-2056-01B | nonresistance |
| TCGA-24-1428-01A | nonresistance |
| TCGA-29-1701-01A | nonresistance |
| TCGA-24-1604-01A | nonresistance |
| TCGA-23-1026-01B | nonresistance |
| TCGA-13-0804-01A | nonresistance |
| TCGA-61-2092-01A | nonresistance |
| TCGA-29-1781-01A | nonresistance |
| TCGA-13-0897-01A | nonresistance |
| TCGA-13-0920-01A | nonresistance |
| TCGA-23-1118-01A | nonresistance |
| TCGA-61-1914-01A | nonresistance |
| TCGA-10-0938-01A | resistance |
| TCGA-29-1774-01A | resistance |
| TCGA-23-1123-01A | resistance |
| TCGA-25-2042-01A | resistance |
| TCGA-61-2002-01A | resistance |
| TCGA-29-1766-01A | resistance |
| TCGA-09-2054-01A | resistance |
| TCGA-25-1628-01A | resistance |
| TCGA-13-0724-01A | resistance |
| TCGA-04-1343-01A | resistance |
| TCGA-04-1361-01A | resistance |
| TCGA-23-1027-01A | resistance |
| TCGA-61-2003-01A | resistance |
| TCGA-29-1776-01A | resistance |

**Table S2.** Primers for Real-time PCR.

| Gene symbol | Primer Sequences | References |
| --- | --- | --- |
| COL1A1 | GCCAAGACGAAGACATCCCA | (Wu et al., 2020) |
|  | GCACCATCATTTCCACGAGC |  |
| miR-98 | CACCGCAGAAGCGGCACTTTATAAGCGAACT | (Niu et al., 2020) |
|  | TTATAAAGTGCCGCTTCTGCTTATAAGTTCGC |  |
| miR-143 | AGTGCGTGTCGTGGTGT | (Chen et al., 2019) |
|  | GCCTGAGATGAAGCACGTG |  |
| LINC00470 | CGTAAGGTGACGAGGAGCTG | (Liu et al., 2018) |
|  | GGGGAATGGCTTTTGGGTCA |  |
| DIRC3 | CCCACCCCACCACTATTCAC | (Coe et al., 2019) |
|  | CCACTGGGACAACCATCTCC |  |
| LINC00052 | CCTGAAGTTTCTCCATGAATTGTG | (Xiong et al., 2019) |
|  | GAGGGAGGGAGACTGAGATT |  |
| FOXP1-IT1 | GCCCCATTTAATTATTTGGAAAGAAGC | (Shi et al., 2019) |
|  | TCCATGTGAACCTTTTGAAATACTGGA |  |
| ATP11A-AS1 | TCAGGTAGCAACTGGAGCCT | Primer 3 |
|  | GTCGCAGTACGGATACCACC |  |
| HERC2P4 | TGGATCACGAGGTCAGAAGA | Primer 3 |
|  | GGTGTCCACCATCATACCTG |  |
| SMCR5 | CCCACAGTTCCCTGTCTTGA | Primer 3 |
|  | GCCTAAGAGCTTTGGGCAGTT |  |
| FLRT1 | CCCCAAGCATCTTCTCAAAG | Primer 3 |
|  | CCACTCTCCAAAGGCTCTGT |  |
| ZNF876P | GCGGATCACAAGTTCAGGAG | Primer 3 |
|  | TCCTGGGTTCACACCATTCT |  |
| HERC2P5 | GGGCACAGGAAACTGACAAT | Primer 3 |
|  | CGTTTGTTGCAGATCAATGG |  |
| FAM86C2P | GCTGTCCACACAGAGCCTTT | Primer 3 |
|  | GGGCCACATAGACTTGAGGA |  |
| LINC00315 | GCTTGACCCTCACCTCTCAC | Primer 3 |
|  | GGTACCAGAGCTTGCTGGAG |  |
| WARS2-IT1 | CAGGGGCAGAATGATATGGT | Primer 3 |
|  | AGCGGCAAGAGAAAAATGAG |  |
| FOXP1-IT1 | TGTTTTGCATGCCTTCTCTG | Primer 3 |
|  | CCAGCAGCGTCTTCTCTCTT |  |
| U6 | ATTGGAACGATACAG AGAAGATT | (Xiong et al., 2019) |
|  | GGAACGCTTCACGAATTTG |  |
| GAPDH | TCAAGAAGGTGGTGAAGCAG | (Xiong et al., 2019) |
|  | CGCTGTTGAAGTCAGAGGAG |  |

**Table S3**. The correlations between module eigengenes and clinical traits.

| Module | Trait | PersonCorrelationValue | Pvalue |
| --- | --- | --- | --- |
| MEyellow | Resistance | 0.215506843 | 0.033077147 |
| MEtan | Resistance | -0.159739176 | 0.116146766 |
| MEmagenta | Resistance | 0.105763399 | 0.299977828 |
| MEmidnightblue | Resistance | 0.095847189 | 0.347818891 |
| MEgreenyellow | Resistance | -0.084071489 | 0.410482228 |
| MEcyan | Resistance | 0.083526819 | 0.413531771 |
| MEsalmon | Resistance | -0.082308254 | 0.42040198 |
| MEpurple | Resistance | 0.080475587 | 0.430857722 |
| MEbrown | Resistance | 0.071367243 | 0.484973314 |
| MEblack | Resistance | -0.068404672 | 0.50332469 |
| MEred | Resistance | 0.048714932 | 0.633826037 |
| MEturquoise | Resistance | 0.043654366 | 0.669514131 |
| MEgreen | Resistance | -0.038523587 | 0.70646287 |
| MEblue | Resistance | 0.022872911 | 0.823104155 |
| MEgrey | Resistance | 0.021236975 | 0.835571544 |
| MEpink | Resistance | -0.012968772 | 0.899144316 |

**Table S4**. 412-carboplatin resistance-related genes in the yellow module.

| Gene symbol |
| --- |
| GLIS2 |
| FOXS1 |
| WNT11 |
| ITGA5 |
| KLF2 |
| NNMT |
| OLFML3 |
| LOXL4 |
| ZCCHC24 |
| ADRA1B |
| FNDC1 |
| VAT1 |
| PRRX1 |
| PAPSS2 |
| DNAJC22 |
| P3H3 |
| KDF1 |
| KCNC3 |
| COL6A3 |
| SORBS3 |
| RGS16 |
| CYP1B1 |
| NRP2 |
| BUD23 |
| VASH1 |
| PMP22 |
| EDNRA |
| SEC23A |
| COL6A1 |
| C1QTNF6 |
| TPBG |
| MICAL2 |
| MAPRE2 |
| SLIT3 |
| CCN1 |
| MRC2 |
| LAMA4 |
| DPYSL3 |
| MMP2 |
| CXCL12 |
| FAT1 |
| TGFB1I1 |
| LTBP2 |
| LXN |
| PDGFRA |
| NDN |
| KDELR3 |
| TMEM119 |
| SEPTIN11 |
| COL10A1 |
| LIX1 |
| NXN |
| SEC24D |
| SFRP4 |
| ERRFI1 |
| EGR2 |
| CYP27A1 |
| HTRA1 |
| CFH |
| FSTL3 |
| VCAM1 |
| SCARF2 |
| PDGFD |
| WWTR1 |
| FNDC4 |
| CEMIP |
| COL15A1 |
| CAV2 |
| APCDD1 |
| ACSL1 |
| SULF1 |
| GASK1B |
| LMCD1 |
| TGM2 |
| CTHRC1 |
| OSBPL10 |
| GAS1 |
| RGCC |
| NBL1 |
| CHSY1 |
| SULF2 |
| LATS2 |
| UNC5B |
| NKD2 |
| LPAR1 |
| CLIC3 |
| DNAJB5 |
| C1QTNF3 |
| CRISPLD2 |
| CNPY4 |
| CREB3L1 |
| SMOC2 |
| KLF10 |
| CDK14 |
| SPATA24 |
| LAMB1 |
| ITIH5 |
| DNAJB4 |
| KCTD10 |
| CALB2 |
| SGCB |
| PODN |
| RUNX1 |
| FSTL1 |
| PDPN |
| LIF |
| NRP1 |
| ENC1 |
| MAP1B |
| CHST15 |
| LAMP5 |
| CD99 |
| CPE |
| PLVAP |
| C1QTNF1 |
| NID1 |
| KRT23 |
| MYLK |
| SRPX2 |
| DNAJC1 |
| COL16A1 |
| PKD2 |
| FBLN5 |
| PODNL1 |
| TGFBI |
| COL11A1 |
| COPZ2 |
| SPON2 |
| IL1RAP |
| ITGB1 |
| ACTA2 |
| FAM89A |
| CLDN11 |
| SPHK1 |
| SNAI1 |
| SNAI2 |
| GNG12 |
| DIPK1A |
| THBS1 |
| GJB2 |
| PARVA |
| MFAP4 |
| PLS3 |
| CILP |
| SFRP1 |
| RGS2 |
| FBLN2 |
| CLEC11A |
| CCL21 |
| MYADM |
| GADD45B |
| COL6A2 |
| SLC39A14 |
| NR2F1 |
| BMP1 |
| SPARC |
| GALNT10 |
| BMP4 |
| LIMS2 |
| KAT8 |
| INHBA |
| BHLHE40 |
| MRAS |
| HBB |
| CDC42EP3 |
| AEBP1 |
| GJA1 |
| FILIP1L |
| NT5E |
| PMEPA1 |
| PLPP1 |
| TWIST1 |
| ID1 |
| GNG11 |
| SCD |
| FERMT2 |
| ADRA2A |
| EMILIN1 |
| P3H4 |
| PDLIM3 |
| TAGLN |
| MCAM |
| ELK3 |
| CNN2 |
| LIX1L |
| CSGALNACT2 |
| ACKR3 |
| S100A10 |
| RHOB |
| PDLIM7 |
| LIMA1 |
| ITGB5 |
| EPDR1 |
| SRPX |
| BAG3 |
| EFEMP2 |
| ENG |
| OSR2 |
| LRRN4 |
| LOXL1 |
| CILP2 |
| AMIGO2 |
| F3 |
| KLF9 |
| APOD |
| TMEM158 |
| SH3PXD2A |
| PDGFB |
| GALNT1 |
| PLPP4 |
| RARRES2 |
| SDC1 |
| ADAMTS2 |
| ST3GAL4 |
| IGFBP4 |
| CAVIN1 |
| PDLIM4 |
| OLFML2A |
| FBXL7 |
| PDGFRB |
| CYB5R3 |
| ISLR |
| ALDH1B1 |
| PXDN |
| C4A |
| CERCAM |
| FKBP7 |
| WIPI1 |
| PDLIM5 |
| CMTM3 |
| EGFL7 |
| POSTN |
| FHL3 |
| ANTXR1 |
| SLCO2A1 |
| TSKU |
| CDKN2B |
| GXYLT2 |
| MSC |
| SHB |
| ID3 |
| RAI14 |
| LUM |
| CHST14 |
| P3H1 |
| HTRA3 |
| MXRA8 |
| TNC |
| TIMP3 |
| PTGER2 |
| COL5A2 |
| PLAT |
| COL4A2 |
| ANGPTL2 |
| LHFPL6 |
| LMOD1 |
| EFEMP1 |
| GLT8D2 |
| THBS2 |
| AXL |
| COL8A2 |
| ELN |
| BASP1 |
| PROCR |
| LGALS1 |
| COLEC12 |
| IGFBP6 |
| COL3A1 |
| CCDC80 |
| CXCL14 |
| FAM20C |
| LOX |
| COMP |
| RCAN1 |
| F13A1 |
| C11orf96 |
| RCN3 |
| MMP14 |
| B3GNT9 |
| SH3D19 |
| MARVELD1 |
| SLC12A8 |
| PIM1 |
| BNIP3L |
| MARCKS |
| SFRP2 |
| TIMP2 |
| BMERB1 |
| PHLDA1 |
| VIM |
| TSPAN18 |
| ITGA11 |
| IGFBP7 |
| COL1A2 |
| SNX18 |
| DAAM2 |
| POGLUT2 |
| MLLT11 |
| HOPX |
| ISM1 |
| PDGFRL |
| MFAP5 |
| CNN3 |
| CAV1 |
| GYPC |
| DKK3 |
| CD248 |
| ROR2 |
| TUBB6 |
| SLC24A3 |
| PDGFC |
| OLFML2B |
| STC1 |
| FAM114A1 |
| ITGA3 |
| COL5A1 |
| TLE4 |
| SLC2A3 |
| ACTG2 |
| ITGA9 |
| FIBIN |
| PLAU |
| TPM2 |
| TSPAN9 |
| CTSK |
| SEMA3C |
| EMP1 |
| PLK2 |
| SYDE1 |
| PTGIS |
| GLI3 |
| PDZRN3 |
| COL26A1 |
| SERPINE1 |
| ETS1 |
| MXRA5 |
| LOXL2 |
| FZD1 |
| OLFML1 |
| STMN3 |
| SUSD2 |
| ANXA6 |
| COL1A1 |
| ADGRA2 |
| ECM1 |
| COL12A1 |
| CHPF |
| PPFIBP1 |
| FAM20A |
| TPM1 |
| MMP11 |
| CDH11 |
| AQP1 |
| PLOD2 |
| FN1 |
| SKIL |
| PCOLCE |
| PALLD |
| LEPROT |
| CPXM2 |
| CCN2 |
| COL8A1 |
| CNN1 |
| RGS5 |
| SOCS3 |
| COL4A1 |
| LBH |
| ARHGEF25 |
| CHN1 |
| VCAN |
| F2R |
| BGN |
| CDC42EP2 |
| SERPINH1 |
| TNFAIP6 |
| CHST11 |
| MFAP2 |
| SPARCL1 |
| FBLIM1 |
| MGP |
| RAB31 |
| FBN1 |
| CCL11 |
| MYL9 |
| SERPINF1 |
| SFXN3 |
| DUSP1 |
| FBLN1 |
| RTN4 |
| SMIM3 |
| EHD2 |
| SDC2 |
| ADGRG2 |
| PLXDC2 |
| PCSK1N |
| TWIST2 |
| SH3PXD2B |
| GPX8 |
| IKBIP |
| CD93 |
| HSPB8 |
| ESAM |
| THY1 |
| CALD1 |
| ANXA2 |
| DES |
| HEG1 |
| RASSF2 |
| TUBB2A |
| DCN |
| IGFBP3 |
| CAVIN3 |

**Table S5**. Hub gene analysis by Deg and MNC methods.

| Name | MNC | Degree |
| --- | --- | --- |
| COL1A1 | 25 | 35 |
| COL5A1 | 23 | 29 |
| SPARC | 25 | 27 |
| CDH11 | 21 | 29 |
| THBS2 | 23 | 27 |
| LUM | 22 | 27 |
| COL5A2 | 19 | 29 |
| COL3A1 | 19 | 28 |
| PMP22 | 17 | 20 |
| PDGFRB | 7 | 29 |
| COL6A3 | 18 | 26 |
| COL11A1 | 18 | 26 |
| FBN1 | 19 | 29 |
| PRRX1 | 20 | 26 |
| FN1 | 19 | 21 |
| ITGA11 | 20 | 26 |
| PDLIM3 | 12 | 20 |
| MMP2 | 23 | 27 |
| COL1A2 | 14 | 27 |
| MSC | 18 | 18 |
| VCAN | 12 | 23 |
| TMEM158 | 10 | 16 |
| ISM1 | 16 | 17 |
| INHBA | 12 | 19 |
| BGN | 14 | 17 |
| DCN | 8 | 22 |
| COL8A1 | 9 | 21 |
| COL10A1 | 14 | 15 |
| RAB31 | 14 | 21 |
| PODN | 14 | 16 |
| COL6A2 | 16 | 16 |
| SERPINF1 | 10 | 19 |
| LOX | 14 | 17 |
| CALB2 | 8 | 12 |
| CREB3L1 | 3 | 17 |
| FZD1 | 6 | 14 |
| CCDC80 | 8 | 16 |
| SULF1 | 8 | 12 |
| OLFML1 | 4 | 16 |
| SFRP2 | 11 | 14 |
| CTHRC1 | 7 | 15 |
| CTSK | 8 | 15 |
| ANGPTL2 | 12 | 13 |
| TIMP2 | 11 | 13 |
| AEBP1 | 9 | 15 |
| ANTXR1 | 8 | 15 |
| ECM1 | 12 | 18 |
| PODNL1 | 10 | 13 |
| CRISPLD2 | 7 | 18 |
| GLT8D2 | 11 | 17 |
| TGFB1I1 | 8 | 13 |
| PTGIS | 13 | 13 |
| EMILIN1 | 14 | 16 |
| THBS1 | 9 | 12 |
| LOXL1 | 6 | 14 |
| OLFML2B | 5 | 14 |
| CALD1 | 6 | 12 |
| COPZ2 | 7 | 19 |
| ZCCHC24 | 6 | 12 |
| ACTA2 | 6 | 14 |
| FILIP1L | 2 | 14 |
| GJB2 | 4 | 11 |
| C1QTNF3 | 7 | 10 |
| LAMA4 | 8 | 10 |
| VCAM1 | 9 | 10 |
| FNDC1 | 3 | 9 |
| POSTN | 11 | 12 |
| PDPN | 6 | 12 |
| ADAMTS2 | 9 | 14 |
| LRRN4 | 5 | 9 |
| HTRA1 | 3 | 10 |
| SERPINE1 | 7 | 8 |
| SNAI2 | 2 | 15 |
| AQP1 | 7 | 8 |
| MMP14 | 9 | 10 |
| NKD2 | 5 | 8 |
| CNN1 | 3 | 9 |
| PMEPA1 | 4 | 9 |
| NBL1 | 4 | 9 |
| TNFAIP6 | 3 | 7 |
| C1QTNF1 | 2 | 9 |
| SCARF2 | 3 | 7 |
| MMP11 | 3 | 9 |
| COL6A1 | 8 | 9 |
| CXCL14 | 5 | 8 |
| LOXL2 | 4 | 8 |
| SMIM3 | 3 | 8 |
| C1QTNF6 | 6 | 8 |
| PLAU | 7 | 9 |
| ACTG2 | 7 | 11 |
| SMOC2 | 4 | 7 |
| PCOLCE | 3 | 9 |
| EDNRA | 2 | 8 |
| HTRA3 | 4 | 8 |
| CXCL12 | 3 | 8 |
| SFRP4 | 4 | 8 |
| CCL11 | 2 | 7 |
| SRPX2 | 2 | 7 |
| MFAP5 | 3 | 6 |
| COLEC12 | 4 | 11 |
| MXRA5 | 3 | 6 |
| IGFBP6 | 4 | 5 |
| CCL21 | 1 | 6 |
| LHFPL6 | 3 | 7 |
| CD248 | 3 | 7 |
| LMOD1 | 2 | 8 |
| COMP | 4 | 7 |
| CERCAM | 6 | 7 |
| PARVA | 3 | 5 |
| ISLR | 3 | 7 |
| COL16A1 | 4 | 12 |
| P3H1 | 2 | 6 |
| GAS1 | 1 | 5 |
| F2R | 3 | 5 |
| FSTL1 | 3 | 7 |
| CCN1 | 2 | 5 |
| SPARCL1 | 1 | 5 |
| EFEMP1 | 4 | 6 |
| RUNX1 | 2 | 4 |
| LXN | 3 | 4 |
| BMP4 | 5 | 6 |
| MRC2 | 2 | 5 |
| TGFBI | 2 | 5 |
| TAGLN | 2 | 7 |
| KLF2 | 3 | 5 |
| PDGFRA | 1 | 6 |
| RGS16 | 2 | 5 |
| HOPX | 1 | 5 |
| PDGFD | 2 | 5 |
| CLDN11 | 1 | 5 |
| COL12A1 | 3 | 5 |
| SPHK1 | 1 | 6 |
| TWIST2 | 2 | 5 |
| GASK1B | 2 | 6 |
| ITGA5 | 2 | 6 |
| RCN3 | 2 | 5 |
| SRPX | 2 | 4 |
| OLFML3 | 3 | 5 |
| CMTM3 | 2 | 4 |
| IGFBP4 | 2 | 5 |
| TPM1 | 3 | 4 |
| THY1 | 1 | 4 |
| DPYSL3 | 2 | 3 |
| PKD2 | 2 | 3 |
| SLC12A8 | 2 | 4 |
| LIMA1 | 3 | 3 |
| TWIST1 | 1 | 3 |
| TIMP3 | 2 | 4 |
| NT5E | 1 | 3 |
| MFAP4 | 1 | 2 |
| GADD45B | 1 | 3 |
| FIBIN | 1 | 3 |
| GPX8 | 1 | 2 |
| ELN | 2 | 2 |
| CNN2 | 2 | 4 |
| ALDH1B1 | 1 | 2 |
| ROR2 | 2 | 2 |
| SEMA3C | 1 | 2 |
| TMEM119 | 1 | 3 |
| RARRES2 | 2 | 3 |
| CILP2 | 1 | 2 |
| COL4A1 | 1 | 2 |
| BMP1 | 2 | 2 |
| PLAT | 1 | 2 |
| FKBP7 | 1 | 2 |
| F13A1 | 1 | 2 |
| PALLD | 1 | 2 |
| CAVIN1 | 1 | 3 |
| CHN1 | 1 | 1 |
| PLVAP | 1 | 1 |
| GALNT10 | 1 | 1 |
| RCAN1 | 1 | 1 |
| MYADM | 1 | 1 |
| COL15A1 | 1 | 2 |
| FSTL3 | 1 | 2 |
| APCDD1 | 1 | 1 |
| TNC | 1 | 1 |
| MICAL2 | 1 | 1 |
| SNAI1 | 1 | 1 |
| CHST11 | 1 | 1 |
| PDGFRL | 1 | 1 |
| SEPTIN11 | 1 | 1 |
| ADRA2A | 1 | 1 |
| ETS1 | 1 | 1 |
| SH3D19 | 1 | 1 |
| EHD2 | 1 | 1 |
| HSPB8 | 1 | 1 |
| SPON2 | 1 | 1 |
| PAPSS2 | 1 | 1 |
| VIM | 1 | 1 |
| IGFBP7 | 2 | 2 |
| NID1 | 1 | 2 |
| CYP1B1 | 1 | 1 |
| PLK2 | 1 | 1 |
| COL8A2 | 1 | 1 |
| TPM2 | 1 | 1 |
| FHL3 | 1 | 1 |
| SULF2 | 1 | 1 |
| ADGRA2 | 1 | 1 |
| RTN4 | 0 | 0 |
| CNN3 | 0 | 0 |
| ANXA2 | 0 | 0 |
| MARCKS | 0 | 0 |
| MXRA8 | 0 | 0 |
| GXYLT2 | 0 | 0 |
| MYL9 | 0 | 0 |
| PLS3 | 0 | 0 |
| DIPK1A | 0 | 0 |
| C11orf96 | 0 | 0 |
| LAMP5 | 0 | 0 |
| CLIC3 | 0 | 0 |
| CSGALNACT2 | 0 | 0 |
| LIX1 | 0 | 0 |
| LTBP2 | 0 | 0 |
| EGR2 | 0 | 0 |
| DUSP1 | 0 | 0 |
| RASSF2 | 0 | 0 |
| HEG1 | 0 | 0 |
| DES | 0 | 0 |
| SERPINH1 | 0 | 0 |
| ANXA6 | 0 | 0 |
| BASP1 | 0 | 0 |
| AXL | 0 | 0 |
| COL4A2 | 0 | 0 |
| ID3 | 0 | 0 |
| FBXL7 | 0 | 0 |
| BHLHE40 | 0 | 0 |
| CILP | 0 | 0 |
| MYLK | 0 | 0 |
| LAMB1 | 0 | 0 |
| CCN2 | 0 | 0 |
| PHLDA1 | 0 | 0 |
| PDLIM5 | 0 | 0 |
| PLPP4 | 0 | 0 |
| PDLIM7 | 0 | 0 |
| PLPP1 | 0 | 0 |
| GJA1 | 0 | 0 |
| LPAR1 | 0 | 0 |
| TGM2 | 0 | 0 |
| CFH | 0 | 0 |
| SEC23A | 0 | 0 |
| PDZRN3 | 0 | 0 |
| LOXL4 | 0 | 0 |

**Table S6**. Differently expression lncRNA between Carboplatin resistant groups and Carboplatin nonresistant groups.

| lncRNA | ENSEMBL | logFC | PValue |
| --- | --- | --- | --- |
| AC010247.2 | ENSG00000259436 | 3.984174 | 3.85E-28 |
| AL163973.3 | ENSG00000258196 | 7.168781 | 1.56E-20 |
| LINC02365 | ENSG00000254233 | 4.213127 | 3.12E-17 |
| LINC01933 | ENSG00000254226 | 3.9491 | 5.19E-17 |
| FRG2FP | ENSG00000232783 | 6.581317 | 6.84E-14 |
| AC090340.1 | ENSG00000277837 | 3.962154 | 9.00E-14 |
| AC016885.1 | ENSG00000253960 | 4.422299 | 2.73E-12 |
| TMEM132D-AS1 | ENSG00000249196 | 4.957291 | 4.77E-12 |
| AC009133.3 | ENSG00000260719 | 3.2593 | 1.45E-10 |
| AC090826.2 | ENSG00000274937 | 3.412082 | 2.68E-09 |
| AP002807.1 | ENSG00000255031 | 1.782911 | 2.69E-09 |
| AL035078.1 | ENSG00000254584 | 2.369513 | 5.23E-09 |
| AC079466.1 | ENSG00000266976 | 2.977601 | 1.81E-08 |
| AC128685.1 | ENSG00000241168 | 3.887017 | 2.14E-08 |
| FAM238B | ENSG00000231976 | 3.264127 | 8.72E-08 |
| AL390961.1 | ENSG00000234296 | 3.656876 | 1.81E-07 |
| LINC00964 | ENSG00000249816 | 2.967742 | 1.91E-07 |
| LINC02180 | ENSG00000261749 | 4.295184 | 2.61E-07 |
| AC004485.1 | ENSG00000228944 | 3.952457 | 4.36E-07 |
| LINC01405 | ENSG00000185847 | 2.817814 | 1.12E-06 |
| LINC02675 | ENSG00000264404 | 2.931578 | 1.68E-06 |
| AC002351.1 | ENSG00000258240 | 3.631218 | 2.47E-06 |
| LINC00895 | ENSG00000281548 | 2.938462 | 2.76E-06 |
| AL034376.1 | ENSG00000205444 | 2.239136 | 2.99E-06 |
| AC118758.3 | ENSG00000279072 | 1.563053 | 4.39E-06 |
| AC084757.3 | ENSG00000259705 | 1.581286 | 5.21E-06 |
| AC004597.1 | ENSG00000268673 | 3.242699 | 8.03E-06 |
| AC083973.1 | ENSG00000253408 | 1.697019 | 8.21E-06 |
| LINC00632 | ENSG00000203930 | 1.930994 | 8.21E-06 |
| AC114488.2 | ENSG00000264078 | 1.733536 | 1.78E-05 |
| AC122694.1 | ENSG00000250122 | 2.563029 | 2.24E-05 |
| AC023983.2 | ENSG00000273321 | 1.620854 | 2.87E-05 |
| MYHAS | ENSG00000272975 | 2.071567 | 3.41E-05 |
| TMEM18-DT | ENSG00000233296 | -3.03953 | 3.52E-05 |
| AL162584.1 | ENSG00000227068 | 2.256089 | 3.54E-05 |
| AL121757.1 | ENSG00000230563 | 2.152722 | 4.66E-05 |
| LINC00607 | ENSG00000235770 | 1.286814 | 5.01E-05 |
| AC084880.3 | ENSG00000256609 | 2.471783 | 6.35E-05 |
| LINC00315 | ENSG00000184274 | 1.078607 | 6.78E-05 |
| LINC02591 | ENSG00000263590 | 1.914814 | 7.89E-05 |
| ANKRD20A19P | ENSG00000196593 | -6.05864 | 7.91E-05 |
| AC134312.1 | ENSG00000205037 | 1.84707 | 8.54E-05 |
| JAKMIP2-AS1 | ENSG00000280780 | 1.999416 | 8.94E-05 |
| AC122134.1 | ENSG00000261298 | 2.540662 | 9.66E-05 |
| AC092159.2 | ENSG00000233970 | -4.5174 | 0.000101 |
| LINC01852 | ENSG00000236914 | 1.035033 | 0.000103 |
| AC004158.1 | ENSG00000260664 | 1.558475 | 0.000117 |
| LINC01524 | ENSG00000234948 | 2.464399 | 0.000124 |
| SNHG22 | ENSG00000267322 | 0.924675 | 0.000153 |
| NFIA-AS2 | ENSG00000237928 | 1.647478 | 0.000157 |
| CISTR | ENSG00000260492 | 2.959813 | 0.000163 |
| AC012645.1 | ENSG00000250616 | 1.025602 | 0.000167 |
| AL031674.1 | ENSG00000232294 | 2.421263 | 0.000167 |
| OPCML-IT1 | ENSG00000254896 | 2.614785 | 0.000181 |
| LINC02629 | ENSG00000223470 | 2.154213 | 0.000182 |
| LINC00189 | ENSG00000215533 | 1.64842 | 0.000193 |
| NECTIN3-AS1 | ENSG00000242242 | 1.233777 | 0.00021 |
| AL136084.3 | ENSG00000270412 | 1.464734 | 0.000212 |
| AP003071.3 | ENSG00000261276 | 1.242441 | 0.000222 |
| COL18A1-AS2 | ENSG00000224574 | 1.422832 | 0.000242 |
| LINC01927 | ENSG00000266312 | 2.013806 | 0.000245 |
| AC005064.1 | ENSG00000234715 | -3.03402 | 0.000301 |
| AC026992.2 | ENSG00000261634 | 1.541387 | 0.000332 |
| LINC01338 | ENSG00000281327 | 1.772743 | 0.000352 |
| LINC02753 | ENSG00000248844 | 2.410065 | 0.000393 |
| AP000785.1 | ENSG00000254933 | 1.892751 | 0.000395 |
| AC209154.1 | ENSG00000276399 | 2.830325 | 0.000397 |
| AC116351.1 | ENSG00000215246 | 1.251978 | 0.000419 |
| AC003973.1 | ENSG00000269504 | 1.921833 | 0.000495 |
| AC079612.1 | ENSG00000196758 | 1.783229 | 0.00052 |
| ITPK1-AS1 | ENSG00000258730 | 1.186268 | 0.00052 |
| AL109946.1 | ENSG00000236064 | -2.52616 | 0.000522 |
| AC025280.2 | ENSG00000279622 | 1.693512 | 0.000531 |
| AP001189.3 | ENSG00000254810 | 1.347874 | 0.000637 |
| AC020934.1 | ENSG00000267424 | 2.009564 | 0.000674 |
| EWSAT1 | ENSG00000212766 | -1.66009 | 0.000683 |
| LINC01166 | ENSG00000232903 | -4.08479 | 0.000728 |
| LINC02043 | ENSG00000232233 | -1.47577 | 0.000748 |
| FLRT1 | ENSG00000256824 | 1.425718 | 0.000783 |
| AL359198.1 | ENSG00000272817 | 0.978945 | 0.000801 |
| LINC02557 | ENSG00000225413 | 2.215501 | 0.000802 |
| AL139353.2 | ENSG00000250365 | 0.753952 | 0.000816 |
| COL18A1-AS1 | ENSG00000183535 | 1.553191 | 0.000867 |
| AL049775.1 | ENSG00000205562 | 1.349552 | 0.000869 |
| SOX21-AS1 | ENSG00000227640 | -4.92144 | 0.000909 |
| AC011840.1 | ENSG00000231421 | 3.341941 | 0.000926 |
| LINC02434 | ENSG00000248370 | 3.124475 | 0.000967 |
| AC010601.1 | ENSG00000259929 | 3.055806 | 0.001013 |
| LINC02370 | ENSG00000230294 | 1.991374 | 0.001028 |
| OVAAL | ENSG00000236719 | -3.23073 | 0.001041 |
| AC018563.2 | ENSG00000259280 | 1.827304 | 0.001107 |
| LINC00237 | ENSG00000225127 | -5.96302 | 0.001116 |
| TEX26-AS1 | ENSG00000224743 | 1.600374 | 0.001163 |
| LINC02006 | ENSG00000238755 | -1.77424 | 0.001211 |
| AC027237.5 | ENSG00000259426 | 0.792671 | 0.001215 |
| SH3RF3-AS1 | ENSG00000259863 | 1.073705 | 0.001252 |
| AC131094.1 | ENSG00000249945 | -5.78036 | 0.001302 |
| AC231533.1 | ENSG00000232828 | 1.328531 | 0.001337 |
| LINC00636 | ENSG00000240423 | 1.388235 | 0.001342 |
| DSCR10 | ENSG00000233316 | 2.501544 | 0.001361 |
| AC087521.1 | ENSG00000244953 | 1.388332 | 0.001381 |
| CTNNA2-AS1 | ENSG00000229385 | 2.389894 | 0.001393 |
| AL049870.3 | ENSG00000258949 | -3.91715 | 0.001404 |
| AC005291.2 | ENSG00000273388 | 1.828379 | 0.001461 |
| AC226119.2 | ENSG00000271977 | -2.71942 | 0.001482 |
| AL109924.1 | ENSG00000229720 | 1.608404 | 0.00149 |
| AP000873.2 | ENSG00000247137 | 0.815716 | 0.001526 |
| LINC00595 | ENSG00000230417 | 1.588465 | 0.00153 |
| HOXB-AS4 | ENSG00000242207 | -3.6352 | 0.001534 |
| AC078909.2 | ENSG00000274444 | 1.456168 | 0.001605 |
| FLNC-AS1 | ENSG00000242902 | 1.901997 | 0.001607 |
| LINC01176 | ENSG00000281404 | -0.93489 | 0.001608 |
| AC007639.1 | ENSG00000263680 | 1.199032 | 0.001711 |
| AC012645.2 | ENSG00000261367 | 1.313226 | 0.001721 |
| NBEAP1 | ENSG00000258590 | -3.75739 | 0.001747 |
| AL356417.2 | ENSG00000233682 | 1.188801 | 0.001757 |
| LINC00581 | ENSG00000280989 | -2.01474 | 0.001759 |
| AP001029.2 | ENSG00000267199 | 0.935118 | 0.001784 |
| LINC00898 | ENSG00000205634 | 1.953209 | 0.0018 |
| LINC01639 | ENSG00000236117 | 2.729581 | 0.001838 |
| AC025280.1 | ENSG00000260859 | 1.627341 | 0.001846 |
| LINC00504 | ENSG00000248360 | 1.389119 | 0.001869 |
| FP671120.8 | ENSG00000281383 | 1.010529 | 0.001873 |
| AC134312.4 | ENSG00000261193 | 1.600565 | 0.001877 |
| AL391988.1 | ENSG00000277879 | -1.07215 | 0.001879 |
| AC246817.1 | ENSG00000253853 | 2.000023 | 0.001935 |
| AC092809.3 | ENSG00000232628 | 1.02397 | 0.001959 |
| AF131216.4 | ENSG00000280273 | 0.850077 | 0.002008 |
| AC100839.2 | ENSG00000259756 | 1.9317 | 0.002008 |
| MPRIP-AS1 | ENSG00000225442 | 1.124142 | 0.00201 |
| LINC01722 | ENSG00000233048 | 1.748478 | 0.002073 |
| LINC00702 | ENSG00000233117 | 1.392294 | 0.002074 |
| AL354864.1 | ENSG00000232335 | -5.78802 | 0.002093 |
| AC099811.4 | ENSG00000267758 | 1.521814 | 0.002109 |
| AC126763.1 | ENSG00000257391 | 1.503716 | 0.00211 |
| AC091946.1 | ENSG00000253766 | -2.76544 | 0.002146 |
| AC107072.1 | ENSG00000224218 | 1.714791 | 0.002179 |
| LINC02683 | ENSG00000254438 | -1.9075 | 0.002206 |
| AL138689.1 | ENSG00000274204 | 1.278818 | 0.002237 |
| AC005256.1 | ENSG00000267073 | -3.73563 | 0.002396 |
| AC012103.1 | ENSG00000253376 | 2.234637 | 0.002496 |
| AC078881.1 | ENSG00000250131 | 1.125905 | 0.002505 |
| HORMAD2-AS1 | ENSG00000227117 | -2.64949 | 0.00258 |
| AC100812.1 | ENSG00000272192 | 1.033878 | 0.002588 |
| AC025810.1 | ENSG00000260042 | 2.552954 | 0.002609 |
| DRAIC | ENSG00000245750 | 0.91636 | 0.002609 |
| AC079906.1 | ENSG00000274723 | -1.25812 | 0.002644 |
| AC139769.2 | ENSG00000267924 | 1.395541 | 0.002718 |
| AL034550.2 | ENSG00000277301 | 1.035977 | 0.002736 |
| AC010761.6 | ENSG00000267729 | -1.70771 | 0.002785 |
| ARHGEF26-AS1 | ENSG00000243069 | -1.66904 | 0.002895 |
| LINC01767 | ENSG00000223956 | -3.39502 | 0.002941 |
| AP003071.4 | ENSG00000261625 | 1.037019 | 0.003023 |
| AC090825.1 | ENSG00000259363 | 0.892434 | 0.003036 |
| AL121827.2 | ENSG00000275620 | 1.887996 | 0.003146 |
| AF131216.3 | ENSG00000255020 | -3.61292 | 0.003236 |
| AL035665.1 | ENSG00000229771 | 1.47596 | 0.003283 |
| AC007114.1 | ENSG00000263004 | -0.99149 | 0.003292 |
| LINC02126 | ENSG00000259847 | 1.454854 | 0.003353 |
| AC019254.1 | ENSG00000259182 | 0.971901 | 0.003383 |
| AC008014.1 | ENSG00000257261 | -0.93605 | 0.003518 |
| LINC01518 | ENSG00000233515 | -4.06892 | 0.00354 |
| AP003973.2 | ENSG00000271390 | 1.394119 | 0.003552 |
| AP001528.1 | ENSG00000255471 | 0.995944 | 0.003575 |
| LINC01932 | ENSG00000253315 | -1.88927 | 0.003576 |
| GRM7-AS1 | ENSG00000236202 | 1.133681 | 0.003607 |
| AP001527.1 | ENSG00000260008 | 1.054632 | 0.003846 |
| SLC26A4-AS1 | ENSG00000233705 | -1.96043 | 0.003859 |
| AL049646.1 | ENSG00000230010 | 1.835957 | 0.003877 |
| AC073316.3 | ENSG00000236708 | 1.446499 | 0.003879 |
| LINC01114 | ENSG00000234177 | -3.0136 | 0.003909 |
| AL133346.1 | ENSG00000227220 | 0.98203 | 0.003948 |
| SALRNA1 | ENSG00000258952 | -2.89737 | 0.003983 |
| AC023855.2 | ENSG00000267638 | 1.211967 | 0.004016 |
| CDKN2B-AS1 | ENSG00000240498 | -1.22237 | 0.004075 |
| AC010168.2 | ENSG00000261324 | -0.87729 | 0.004103 |
| MNX1-AS1 | ENSG00000243479 | -3.85523 | 0.004103 |
| AC022167.3 | ENSG00000260979 | 1.035552 | 0.004112 |
| LINC01608 | ENSG00000253877 | -5.62801 | 0.004159 |
| AC078845.1 | ENSG00000232053 | -2.41575 | 0.004182 |
| PCDHB17P | ENSG00000255622 | -2.14208 | 0.004184 |
| TPTEP1 | ENSG00000100181 | -2.35023 | 0.004204 |
| AP001432.1 | ENSG00000242553 | 0.729666 | 0.004219 |
| LINC01269 | ENSG00000258689 | -1.92913 | 0.00426 |
| AP000432.1 | ENSG00000226956 | 1.717065 | 0.004391 |
| NAV2-AS4 | ENSG00000254622 | 1.5806 | 0.004396 |
| LINC01586 | ENSG00000249487 | 1.150525 | 0.004435 |
| AC084866.1 | ENSG00000249955 | -5.4393 | 0.004488 |
| C8orf37-AS1 | ENSG00000253773 | -1.10207 | 0.004496 |
| LINC02048 | ENSG00000228271 | 1.153656 | 0.004617 |
| AC117422.1 | ENSG00000248607 | 1.413576 | 0.004656 |
| LINC01448 | ENSG00000238284 | 2.159671 | 0.004664 |
| AP000943.1 | ENSG00000233536 | 2.046065 | 0.004686 |
| LINC02141 | ENSG00000261807 | -2.83018 | 0.00471 |
| AC022211.4 | ENSG00000265800 | -0.98552 | 0.004746 |
| AC007881.2 | ENSG00000236469 | 1.569891 | 0.004838 |
| AC087636.1 | ENSG00000258773 | 2.079655 | 0.004874 |
| LINC02355 | ENSG00000248210 | -2.51221 | 0.00498 |
| AQP4-AS1 | ENSG00000260372 | -1.86806 | 0.005 |
| AC073343.2 | ENSG00000228010 | 0.88612 | 0.005005 |
| AP001099.1 | ENSG00000265728 | -2.14167 | 0.005049 |
| TMEM231P1 | ENSG00000262583 | -1.10143 | 0.005133 |
| AC010422.2 | ENSG00000268945 | 0.893717 | 0.005138 |
| AC131902.1 | ENSG00000260484 | -2.85652 | 0.005143 |
| AC099850.2 | ENSG00000224738 | -0.68547 | 0.00522 |
| AL162411.1 | ENSG00000236924 | -1.1539 | 0.005248 |
| AC073263.1 | ENSG00000217702 | 0.983107 | 0.005296 |
| AC100839.1 | ENSG00000259458 | 1.487301 | 0.005389 |
| AC079313.1 | ENSG00000258086 | 1.063909 | 0.005424 |
| AL049794.1 | ENSG00000273998 | 0.984807 | 0.005493 |
| CSTP1 | ENSG00000228476 | 1.746702 | 0.005518 |
| GRIK1-AS1 | ENSG00000174680 | 1.478284 | 0.005532 |
| AC004817.4 | ENSG00000269945 | -4.05327 | 0.0057 |
| AC004854.2 | ENSG00000272768 | 0.653146 | 0.00572 |
| CPNE8-AS1 | ENSG00000257718 | -1.4256 | 0.005745 |
| LINC00863 | ENSG00000224914 | 0.604312 | 0.005765 |
| AC009812.4 | ENSG00000260317 | -0.82261 | 0.0058 |
| AL133320.2 | ENSG00000275678 | -2.51609 | 0.005948 |
| AC087362.1 | ENSG00000254898 | 1.164608 | 0.006055 |
| NGF-AS1 | ENSG00000228035 | 1.297336 | 0.006089 |
| PRKG1-AS1 | ENSG00000236671 | 1.457959 | 0.006126 |
| SMCR5 | ENSG00000226746 | 1.090236 | 0.006133 |
| FO393401.1 | ENSG00000242507 | 1.391437 | 0.006344 |
| AC090983.1 | ENSG00000280118 | 1.784267 | 0.006353 |
| HPAT5 | ENSG00000280707 | 1.688192 | 0.006356 |
| LINC02677 | ENSG00000228951 | 1.21134 | 0.006358 |
| AC103702.2 | ENSG00000272763 | -2.08826 | 0.006421 |
| AC233266.2 | ENSG00000261600 | 1.417039 | 0.0065 |
| FP671120.7 | ENSG00000280800 | 1.448788 | 0.006524 |
| AP005264.3 | ENSG00000267247 | -2.85309 | 0.006533 |
| AC026469.1 | ENSG00000275088 | 1.035439 | 0.006602 |
| LINC01159 | ENSG00000229743 | -5.65014 | 0.00664 |
| LINC02544 | ENSG00000261039 | 1.332518 | 0.006649 |
| AC111170.2 | ENSG00000267016 | 1.112262 | 0.006674 |
| AC134312.3 | ENSG00000260166 | 1.644865 | 0.006719 |
| ADAMTSL4-AS2 | ENSG00000237781 | 0.826519 | 0.006724 |
| AC245100.1 | ENSG00000224481 | -1.27987 | 0.006727 |
| LINC01140 | ENSG00000267272 | 0.782876 | 0.00702 |
| LINC02806 | ENSG00000238107 | -1.67683 | 0.007047 |
| AC015813.1 | ENSG00000264112 | -0.91742 | 0.007128 |
| LINC02764 | ENSG00000255484 | -1.84563 | 0.007143 |
| LINC01541 | ENSG00000260676 | -7.34796 | 0.007153 |
| AC013451.1 | ENSG00000258425 | 1.085443 | 0.00728 |
| AC005759.1 | ENSG00000268650 | -1.13072 | 0.00734 |
| AC140118.1 | ENSG00000256484 | 2.11291 | 0.007533 |
| AC133785.1 | ENSG00000233221 | 1.315662 | 0.00756 |
| AL035401.1 | ENSG00000233358 | 2.150272 | 0.007567 |
| AC089998.1 | ENSG00000257431 | 1.540825 | 0.00762 |
| AC011510.1 | ENSG00000269652 | 1.213946 | 0.007676 |
| AP002336.3 | ENSG00000255539 | 1.208435 | 0.007896 |
| AC005532.1 | ENSG00000230825 | -1.20862 | 0.008235 |
| PKIA-AS1 | ENSG00000254266 | -2.16157 | 0.008329 |
| LINC01954 | ENSG00000271952 | -2.02786 | 0.008404 |
| LINC01251 | ENSG00000233776 | -3.40678 | 0.008502 |
| THORLNC | ENSG00000226856 | -1.30366 | 0.008527 |
| GUSBP1 | ENSG00000183666 | -0.60422 | 0.00854 |
| AC099542.1 | ENSG00000241593 | -2.03856 | 0.00856 |
| AL137025.1 | ENSG00000228484 | -1.48573 | 0.008579 |
| TMLHE-AS1 | ENSG00000224533 | -1.14259 | 0.008612 |
| AP005131.5 | ENSG00000267694 | 0.977901 | 0.008665 |
| AL021877.2 | ENSG00000280356 | -2.79522 | 0.008784 |
| FOXD2-AS1 | ENSG00000237424 | -1.26672 | 0.008797 |
| MYADM-AS1 | ENSG00000228323 | 0.853044 | 0.008885 |
| AC022893.3 | ENSG00000272254 | 1.20536 | 0.008993 |
| LINC01151 | ENSG00000253819 | -2.24388 | 0.009029 |
| AC004951.2 | ENSG00000239556 | 0.62627 | 0.009054 |
| AC123788.1 | ENSG00000254592 | -2.04737 | 0.009101 |
| AL160270.1 | ENSG00000230729 | 1.240623 | 0.009177 |
| AL161740.1 | ENSG00000236341 | 1.884202 | 0.009307 |
| LINC02723 | ENSG00000231680 | 1.106316 | 0.009309 |
| CARMN | ENSG00000249669 | 0.790888 | 0.009367 |
| AP001189.1 | ENSG00000236304 | 0.960743 | 0.009389 |
| CCDC183-AS1 | ENSG00000228544 | 0.735763 | 0.009397 |
| RAB30-DT | ENSG00000246067 | 0.741925 | 0.009413 |
| AL356020.1 | ENSG00000258731 | 0.837649 | 0.009446 |
| AC003965.2 | ENSG00000263325 | -1.06738 | 0.009664 |
| LINC02475 | ENSG00000251350 | 1.429778 | 0.009763 |
| AC005920.1 | ENSG00000247011 | -1.15972 | 0.00977 |
| LINC02406 | ENSG00000258144 | -3.08636 | 0.009851 |
| NEXN-AS1 | ENSG00000235927 | 0.864816 | 0.009855 |
| AC013564.1 | ENSG00000274995 | 1.142624 | 0.009869 |
| RAI1-AS1 | ENSG00000237328 | 0.875871 | 0.009947 |
| LINC01511 | ENSG00000250584 | -4.23492 | 0.009968 |
| MIR210HG | ENSG00000247095 | -0.75645 | 0.010006 |
| AP002336.1 | ENSG00000254484 | 0.955242 | 0.010036 |
| LINC02055 | ENSG00000254101 | -3.65674 | 0.010103 |
| BX324167.1 | ENSG00000235091 | -2.86506 | 0.010383 |
| CACNA1G-AS1 | ENSG00000250107 | 0.985609 | 0.010392 |
| FHAD1-AS1 | ENSG00000233485 | 1.126144 | 0.010396 |
| FARSA-AS1 | ENSG00000266975 | 0.83901 | 0.0104 |
| AC015845.2 | ENSG00000265542 | -1.25236 | 0.010434 |
| AC104316.2 | ENSG00000259631 | -1.86437 | 0.010648 |
| AL353194.1 | ENSG00000229539 | 0.638325 | 0.010659 |
| AC084782.1 | ENSG00000259941 | 1.258797 | 0.010691 |
| AL121821.2 | ENSG00000258636 | 1.303265 | 0.010846 |
| ZNF674-AS1 | ENSG00000230844 | 0.5451 | 0.010949 |
| AL356652.1 | ENSG00000278367 | 0.84854 | 0.010994 |
| AC027807.2 | ENSG00000274128 | 1.127331 | 0.01108 |
| AC011008.1 | ENSG00000253695 | 1.145116 | 0.0111 |
| AC007950.2 | ENSG00000276651 | 0.607546 | 0.011101 |
| EDRF1-DT | ENSG00000224023 | -0.76607 | 0.011102 |
| TESC-AS1 | ENSG00000258285 | 1.114408 | 0.011114 |
| AC055811.1 | ENSG00000263624 | 0.94231 | 0.01113 |
| ANAPC1P1 | ENSG00000233673 | -1.7621 | 0.011202 |
| CHL1-AS2 | ENSG00000224318 | -1.52718 | 0.011331 |
| AL049874.3 | ENSG00000258670 | -3.58235 | 0.011366 |
| LINC00621 | ENSG00000262619 | 0.983772 | 0.011366 |
| AC105384.1 | ENSG00000249706 | 1.289159 | 0.011428 |
| AC007848.2 | ENSG00000256218 | 1.321996 | 0.01144 |
| AL109615.3 | ENSG00000237686 | -0.83244 | 0.011484 |
| AL109947.1 | ENSG00000223537 | -1.31308 | 0.011489 |
| AP000487.2 | ENSG00000254495 | 0.763566 | 0.011512 |
| AL359313.1 | ENSG00000236366 | -2.43301 | 0.011577 |
| AC064875.1 | ENSG00000225649 | 0.991235 | 0.011636 |
| LINC02649 | ENSG00000215244 | -0.90507 | 0.011674 |
| AC079160.1 | ENSG00000250546 | 1.406918 | 0.011808 |
| AC021613.1 | ENSG00000254092 | -2.56902 | 0.011829 |
| GTF2H2B | ENSG00000226259 | -1.20934 | 0.011833 |
| AC105219.4 | ENSG00000254973 | -0.91203 | 0.011842 |
| AC008060.4 | ENSG00000260426 | -4.69347 | 0.01185 |
| LINC01305 | ENSG00000231453 | -3.54658 | 0.011855 |
| LRIG2-DT | ENSG00000238198 | 0.569296 | 0.012199 |
| AC021491.4 | ENSG00000275897 | -0.94292 | 0.012209 |
| AL355073.1 | ENSG00000259483 | -1.0317 | 0.012295 |
| CELP | ENSG00000170827 | -2.23552 | 0.012357 |
| AC134878.2 | ENSG00000278212 | 1.120072 | 0.012369 |
| ITGA6-AS1 | ENSG00000232788 | -0.87116 | 0.012516 |
| FRG1-DT | ENSG00000245685 | 0.747371 | 0.012636 |
| USP27X-AS1 | ENSG00000234390 | 0.570387 | 0.012645 |
| HERC2P4 | ENSG00000230267 | 1.277156 | 0.012658 |
| AL360093.1 | ENSG00000223842 | 1.191885 | 0.0127 |
| FAM85B | ENSG00000253893 | 0.917044 | 0.012771 |
| GACAT2 | ENSG00000265962 | 1.696806 | 0.012882 |
| AC068492.1 | ENSG00000237262 | -1.79815 | 0.013019 |
| LINC01960 | ENSG00000260868 | 0.98274 | 0.013169 |
| AL109945.1 | ENSG00000233775 | 0.834996 | 0.013233 |
| AP002336.2 | ENSG00000254604 | 0.642309 | 0.01324 |
| AL513327.3 | ENSG00000270115 | 0.812546 | 0.013299 |
| AC005586.2 | ENSG00000261305 | 0.890296 | 0.013302 |
| DTD1-AS1 | ENSG00000233993 | 1.120857 | 0.013344 |
| AL157414.1 | ENSG00000203266 | 1.561468 | 0.013367 |
| AC114401.1 | ENSG00000241884 | -1.43337 | 0.013402 |
| MIR924HG | ENSG00000267374 | -1.05865 | 0.01345 |
| AL023881.1 | ENSG00000276984 | 0.934628 | 0.013456 |
| AC020558.1 | ENSG00000264666 | 0.643555 | 0.013466 |
| MIS18A-AS1 | ENSG00000227256 | 0.631314 | 0.013624 |
| AC005899.7 | ENSG00000274341 | -0.9278 | 0.013682 |
| AC034195.1 | ENSG00000223727 | -1.94749 | 0.013715 |
| LINC01711 | ENSG00000268941 | 0.98349 | 0.013823 |
| ITFG1-AS1 | ENSG00000260281 | -0.88274 | 0.013852 |
| AL033523.1 | ENSG00000228793 | -1.83279 | 0.01389 |
| VIM-AS1 | ENSG00000229124 | -0.94363 | 0.013903 |
| FAM53B-AS1 | ENSG00000233334 | 0.825845 | 0.01407 |
| LINC01940 | ENSG00000227744 | 1.29121 | 0.014071 |
| AC017067.1 | ENSG00000224549 | 1.533716 | 0.014078 |
| AC013287.1 | ENSG00000228566 | 1.620607 | 0.014086 |
| AL591643.1 | ENSG00000260683 | -4.99586 | 0.014206 |
| ZNF350-AS1 | ENSG00000269235 | 1.174631 | 0.014342 |
| AC008060.1 | ENSG00000218672 | -4.67914 | 0.014386 |
| AC090282.1 | ENSG00000261848 | 1.498198 | 0.01443 |
| AP003555.2 | ENSG00000255191 | 1.050404 | 0.014573 |
| AC012213.4 | ENSG00000271830 | -2.45794 | 0.014856 |
| AC239584.1 | ENSG00000249942 | 1.321012 | 0.015 |
| AC048337.1 | ENSG00000258435 | 0.864025 | 0.015069 |
| PGM5-AS1 | ENSG00000224958 | -1.96477 | 0.015138 |
| AC232271.1 | ENSG00000270012 | 0.60286 | 0.015153 |
| AC241644.2 | ENSG00000234190 | -2.1969 | 0.015459 |
| AC090515.6 | ENSG00000259402 | 0.891138 | 0.015468 |
| AC119396.2 | ENSG00000267852 | -0.96079 | 0.015481 |
| LINC00377 | ENSG00000229246 | -2.35548 | 0.015501 |
| NDP-AS1 | ENSG00000236276 | -3.35444 | 0.015567 |
| AL592546.2 | ENSG00000234393 | -1.51186 | 0.015634 |
| OLMALINC | ENSG00000235823 | -0.55555 | 0.015668 |
| AC034111.1 | ENSG00000260093 | 0.825254 | 0.015701 |
| AP005131.6 | ENSG00000267702 | 0.717358 | 0.01583 |
| AC007342.5 | ENSG00000262714 | -1.45663 | 0.016039 |
| AC005332.7 | ENSG00000278740 | -1.28536 | 0.016127 |
| LINC00526 | ENSG00000264575 | 0.619314 | 0.016129 |
| AC010336.1 | ENSG00000214248 | -0.74323 | 0.016289 |
| AC090136.3 | ENSG00000254102 | 1.245116 | 0.016459 |
| AC078802.1 | ENSG00000269889 | -0.79361 | 0.016578 |
| NR4A1AS | ENSG00000259884 | 1.014006 | 0.016641 |
| LINC02634 | ENSG00000233200 | -2.10694 | 0.016796 |
| LINC02086 | ENSG00000244649 | -1.74956 | 0.016798 |
| SLCO4A1-AS1 | ENSG00000232803 | 1.036502 | 0.016819 |
| CATIP-AS2 | ENSG00000237281 | 0.674021 | 0.016833 |
| ERVK-28 | ENSG00000267696 | -2.78947 | 0.016863 |
| AC022898.2 | ENSG00000273792 | 1.034151 | 0.016888 |
| FREM2-AS1 | ENSG00000225350 | -5.97913 | 0.016952 |
| AC004066.2 | ENSG00000250522 | -2.03883 | 0.016983 |
| LINC01108 | ENSG00000226673 | 1.18173 | 0.016989 |
| AL161756.1 | ENSG00000214770 | -0.61617 | 0.017366 |
| AC023509.3 | ENSG00000270175 | 0.626978 | 0.017381 |
| U95743.1 | ENSG00000262267 | 1.982158 | 0.017492 |
| AL109910.1 | ENSG00000230960 | 1.739677 | 0.0175 |
| AC104695.4 | ENSG00000270640 | 0.838576 | 0.017548 |
| AC074011.1 | ENSG00000230730 | -1.00376 | 0.017612 |
| AP001453.2 | ENSG00000256116 | 0.586355 | 0.017613 |
| LINC01854 | ENSG00000204460 | 2.04107 | 0.017686 |
| ERCC8-AS1 | ENSG00000233847 | 0.892339 | 0.017762 |
| WASIR1 | ENSG00000185203 | 1.211924 | 0.017944 |
| THRB-AS1 | ENSG00000228791 | -0.86512 | 0.018081 |
| LINC02595 | ENSG00000231566 | 0.728143 | 0.018092 |
| AP000688.2 | ENSG00000233393 | -1.5954 | 0.018098 |
| AC114760.2 | ENSG00000272211 | 0.869156 | 0.018296 |
| HERC2P5 | ENSG00000260644 | 1.206285 | 0.018312 |
| BX255923.2 | ENSG00000276462 | -1.60678 | 0.018405 |
| AL050403.2 | ENSG00000270792 | 0.846373 | 0.018449 |
| AP000704.2 | ENSG00000224790 | 0.513359 | 0.018467 |
| AC025034.1 | ENSG00000258302 | -0.85907 | 0.018484 |
| AL732292.2 | ENSG00000273416 | 0.731455 | 0.018499 |
| LINC01257 | ENSG00000204603 | 1.15575 | 0.018556 |
| AC020659.2 | ENSG00000250379 | 1.01813 | 0.018563 |
| AL132780.2 | ENSG00000258457 | 0.596945 | 0.018597 |
| DNM3OS | ENSG00000230630 | 0.623874 | 0.018627 |
| AC079766.1 | ENSG00000272646 | 0.81551 | 0.018631 |
| AC078850.1 | ENSG00000248187 | 1.075807 | 0.018638 |
| AL358216.1 | ENSG00000225140 | 1.119252 | 0.018718 |
| AC025211.1 | ENSG00000264458 | 1.198404 | 0.01881 |
| AC005082.1 | ENSG00000226816 | -1.36127 | 0.018853 |
| AC099521.2 | ENSG00000274698 | 0.896714 | 0.018925 |
| AC090241.2 | ENSG00000270112 | -1.43624 | 0.019011 |
| AC012531.2 | ENSG00000273046 | 1.111059 | 0.019033 |
| PDCD6IPP1 | ENSG00000275325 | -1.43114 | 0.019122 |
| ENPP7P8 | ENSG00000255319 | 0.955681 | 0.019143 |
| AC091100.1 | ENSG00000259422 | 0.937242 | 0.019176 |
| ANKRD62P1 | ENSG00000259271 | -3.1267 | 0.019187 |
| AC006504.1 | ENSG00000261770 | 0.709867 | 0.019253 |
| AC104435.2 | ENSG00000244345 | -1.57741 | 0.01945 |
| AC100791.2 | ENSG00000262768 | 1.4841 | 0.019512 |
| AC093788.1 | ENSG00000273449 | 0.6768 | 0.019517 |
| OR52A4P | ENSG00000205494 | -4.50902 | 0.019523 |
| ATP11A-AS1 | ENSG00000232684 | 0.962129 | 0.019534 |
| LINC00052 | ENSG00000259527 | 1.647441 | 0.019558 |
| AC107973.1 | ENSG00000254734 | -4.37062 | 0.019597 |
| FAM167A-AS1 | ENSG00000184608 | -5.31103 | 0.019736 |
| AC009228.1 | ENSG00000242628 | 0.817733 | 0.019875 |
| LINC02139 | ENSG00000278214 | 1.002339 | 0.019961 |
| MIR663AHG | ENSG00000227195 | 1.087197 | 0.020106 |
| CASC18 | ENSG00000257859 | 0.916651 | 0.020119 |
| AC087749.2 | ENSG00000264829 | 1.021584 | 0.020157 |
| AC004817.1 | ENSG00000258517 | -3.52887 | 0.020163 |
| MYRF-AS1 | ENSG00000124915 | 0.960204 | 0.020182 |
| AC104964.3 | ENSG00000261451 | 0.910723 | 0.020184 |
| CYP2B7P | ENSG00000256612 | -1.5276 | 0.020252 |
| AC093525.7 | ENSG00000269937 | 0.916037 | 0.020289 |
| AC026368.1 | ENSG00000277840 | 0.751536 | 0.020319 |
| AL035461.3 | ENSG00000275632 | 0.707231 | 0.020369 |
| LINC00937 | ENSG00000226091 | -0.78293 | 0.020371 |
| MIR583HG | ENSG00000250551 | 1.208637 | 0.020421 |
| AC005476.2 | ENSG00000259146 | -0.70119 | 0.020432 |
| LINC00524 | ENSG00000259023 | -2.32801 | 0.020469 |
| AL079303.1 | ENSG00000258661 | -2.63891 | 0.02047 |
| LINC01445 | ENSG00000231427 | -3.02787 | 0.020476 |
| AL139147.1 | ENSG00000248458 | 1.184974 | 0.020553 |
| LINC01641 | ENSG00000234277 | 0.903473 | 0.020603 |
| AC106799.3 | ENSG00000260763 | 1.775855 | 0.02062 |
| AC005355.1 | ENSG00000250994 | 1.29777 | 0.02066 |
| AC007342.4 | ENSG00000261804 | -1.43688 | 0.020736 |
| PANTR1 | ENSG00000233639 | -4.99304 | 0.020802 |
| AL512506.1 | ENSG00000274001 | 0.866713 | 0.02087 |
| AC008875.1 | ENSG00000271788 | -1.08951 | 0.02089 |
| AC023794.3 | ENSG00000248576 | -1.97421 | 0.020898 |
| ANKRD20A8P | ENSG00000229089 | -1.87729 | 0.020919 |
| LDLRAD4-AS1 | ENSG00000267690 | 0.890387 | 0.020955 |
| LINC01549 | ENSG00000232560 | -2.61511 | 0.020986 |
| AC051618.1 | ENSG00000236958 | 1.469109 | 0.020986 |
| LINC01819 | ENSG00000231826 | -1.81989 | 0.021071 |
| AC069061.2 | ENSG00000264734 | -4.45994 | 0.021111 |
| BHLHE40-AS1 | ENSG00000235831 | -0.77934 | 0.021212 |
| AC245041.1 | ENSG00000273760 | 1.317879 | 0.021234 |
| LINC01229 | ENSG00000260876 | -2.59008 | 0.021248 |
| TPM1-AS | ENSG00000259498 | 0.743264 | 0.021268 |
| AC024901.1 | ENSG00000255910 | 1.168051 | 0.02127 |
| IGFBP7-AS1 | ENSG00000245067 | 0.740263 | 0.021373 |
| AC126773.4 | ENSG00000260577 | 0.695444 | 0.021418 |
| AC093535.1 | ENSG00000250602 | -0.87489 | 0.021424 |
| AL591767.1 | ENSG00000258400 | 0.957951 | 0.021476 |
| AC007878.1 | ENSG00000281195 | 0.828554 | 0.021514 |
| AP001025.1 | ENSG00000266578 | 0.855448 | 0.021624 |
| AL078604.2 | ENSG00000237927 | -1.32393 | 0.021703 |
| AL158835.1 | ENSG00000228021 | -0.7294 | 0.021955 |
| HOXC13-AS | ENSG00000249641 | -3.93978 | 0.021971 |
| AC116096.1 | ENSG00000261734 | 1.189815 | 0.022144 |
| AL139383.1 | ENSG00000230490 | -1.00322 | 0.022144 |
| MCF2L-AS1 | ENSG00000235280 | -1.10315 | 0.022208 |
| AC002091.1 | ENSG00000265975 | 0.6716 | 0.022224 |
| AC079298.3 | ENSG00000280241 | 1.188568 | 0.022242 |
| ALDH1L1-AS2 | ENSG00000246022 | 1.170104 | 0.022247 |
| AL157832.1 | ENSG00000227374 | 0.707387 | 0.022288 |
| ATP13A5-AS1 | ENSG00000236508 | -1.22303 | 0.022296 |
| AL132655.2 | ENSG00000268649 | 0.943757 | 0.022322 |
| AC022973.3 | ENSG00000253720 | 0.77574 | 0.022366 |
| AC023824.5 | ENSG00000261800 | 1.166217 | 0.02246 |
| LINC01133 | ENSG00000224259 | -1.45053 | 0.02251 |
| DLGAP1-AS4 | ENSG00000263878 | 1.558761 | 0.022934 |
| AC017104.3 | ENSG00000233538 | -1.53922 | 0.022967 |
| AL354714.1 | ENSG00000198358 | -2.06338 | 0.023052 |
| GSTA9P | ENSG00000243236 | -2.02189 | 0.023103 |
| RASSF1-AS1 | ENSG00000281358 | -0.78654 | 0.023183 |
| SLC2A1-AS1 | ENSG00000227533 | -0.6585 | 0.023268 |
| AL021154.1 | ENSG00000235052 | 0.73776 | 0.023277 |
| AC011379.1 | ENSG00000250069 | 0.748131 | 0.023307 |
| AL023284.4 | ENSG00000260418 | 0.699892 | 0.023431 |
| AC024940.2 | ENSG00000177359 | -1.43695 | 0.023443 |
| AC008938.1 | ENSG00000260072 | -2.36161 | 0.023479 |
| FAM198B-AS1 | ENSG00000248429 | 0.630962 | 0.023666 |
| LYPLAL1-DT | ENSG00000228063 | 1.321493 | 0.023686 |
| AC090877.2 | ENSG00000259721 | 1.21426 | 0.023893 |
| FOXCUT | ENSG00000280916 | -1.94397 | 0.024025 |
| ELDR | ENSG00000280890 | 1.007019 | 0.024037 |
| AC138512.1 | ENSG00000261273 | 1.033537 | 0.024074 |
| AL162419.1 | ENSG00000228322 | -1.29029 | 0.024234 |
| GPC5-AS1 | ENSG00000235984 | -2.69438 | 0.024352 |
| AL356215.1 | ENSG00000255521 | -1.69274 | 0.024379 |
| AC004241.2 | ENSG00000274902 | -0.73376 | 0.02441 |
| AP001107.5 | ENSG00000254510 | 0.957833 | 0.024516 |
| AL445465.2 | ENSG00000237174 | -2.86671 | 0.024528 |
| AL935212.1 | ENSG00000204814 | 1.245021 | 0.024546 |
| AC131097.1 | ENSG00000215023 | -1.18514 | 0.02455 |
| AL163636.2 | ENSG00000258451 | 0.927452 | 0.024615 |
| AC011444.2 | ENSG00000267262 | 1.153576 | 0.024649 |
| NBAT1 | ENSG00000260455 | -2.1604 | 0.024655 |
| AC069234.4 | ENSG00000276188 | 0.833612 | 0.024661 |
| LINC01582 | ENSG00000259611 | -4.85481 | 0.02477 |
| HTR5A-AS1 | ENSG00000220575 | 1.956318 | 0.024801 |
| AP000808.1 | ENSG00000255741 | 0.671852 | 0.024836 |
| AL109615.2 | ENSG00000231881 | -1.04587 | 0.024898 |
| AC008641.1 | ENSG00000254298 | 1.22699 | 0.024995 |
| LINC01127 | ENSG00000281162 | -1.19308 | 0.025222 |
| AC005840.2 | ENSG00000256433 | 0.63135 | 0.025266 |
| AC093904.3 | ENSG00000244310 | -2.32643 | 0.025395 |
| AC092535.2 | ENSG00000227189 | 1.106995 | 0.025422 |
| AC008897.2 | ENSG00000247372 | -1.60665 | 0.025507 |
| CLMAT3 | ENSG00000249035 | 0.853767 | 0.025572 |
| LINC01931 | ENSG00000162947 | -1.31978 | 0.025582 |
| AL021937.1 | ENSG00000230736 | -0.85497 | 0.025583 |
| AC020978.4 | ENSG00000261469 | 0.684668 | 0.025632 |
| AC112178.1 | ENSG00000249803 | -3.56006 | 0.025688 |
| LINC00654 | ENSG00000205181 | 0.775148 | 0.02576 |
| FAM66E | ENSG00000225725 | 0.969729 | 0.025776 |
| AC004817.2 | ENSG00000259907 | -2.4503 | 0.025818 |
| AL358074.1 | ENSG00000229582 | 0.818825 | 0.025848 |
| AL354813.1 | ENSG00000255438 | 0.985522 | 0.025859 |
| AF127936.1 | ENSG00000232884 | 0.970306 | 0.025875 |
| AP000941.1 | ENSG00000255176 | 0.769614 | 0.026074 |
| AC006504.5 | ENSG00000267575 | 0.447983 | 0.026143 |
| AL355574.1 | ENSG00000238058 | 0.488974 | 0.026249 |
| AC010503.5 | ENSG00000275234 | -0.56457 | 0.026427 |
| AL132801.2 | ENSG00000259508 | -1.73673 | 0.026483 |
| AL354893.2 | ENSG00000229065 | 0.903754 | 0.026531 |
| AC036222.3 | ENSG00000264451 | -1.59203 | 0.026532 |
| BX640514.2 | ENSG00000273812 | -1.12625 | 0.026535 |
| AC009542.2 | ENSG00000276067 | -1.49338 | 0.026692 |
| AL118522.1 | ENSG00000276223 | -1.16691 | 0.026826 |
| AC011373.1 | ENSG00000272239 | 0.998379 | 0.026869 |
| AC226119.1 | ENSG00000253917 | -2.72876 | 0.026899 |
| AC083867.2 | ENSG00000225559 | 1.298178 | 0.02695 |
| AC004471.1 | ENSG00000223461 | -0.83165 | 0.027098 |
| AL353764.1 | ENSG00000233081 | -0.94533 | 0.02735 |
| DDX59-AS1 | ENSG00000260088 | -1.08674 | 0.027371 |
| AL158071.5 | ENSG00000273381 | -0.79165 | 0.027376 |
| ROR1-AS1 | ENSG00000223949 | 0.776774 | 0.02746 |
| AP000911.1 | ENSG00000254648 | 0.9005 | 0.027473 |
| AC093422.2 | ENSG00000225063 | 1.007189 | 0.027514 |
| LINC01887 | ENSG00000266604 | -1.84167 | 0.027692 |
| AL161669.3 | ENSG00000278071 | -0.92041 | 0.027696 |
| AC008567.2 | ENSG00000267273 | 0.868249 | 0.027699 |
| AP000462.2 | ENSG00000256315 | 1.253356 | 0.027739 |
| AC090796.1 | ENSG00000253821 | 1.303032 | 0.027749 |
| AC099754.1 | ENSG00000225386 | -2.28308 | 0.027765 |
| PTPRD-AS1 | ENSG00000225706 | 0.991344 | 0.027815 |
| AL445222.2 | ENSG00000271833 | 0.579016 | 0.027894 |
| NFIA-AS1 | ENSG00000237853 | 0.876039 | 0.028019 |
| AC109635.3 | ENSG00000255042 | -3.54367 | 0.028098 |
| LINC02280 | ENSG00000260792 | 0.740861 | 0.028113 |
| AC064834.1 | ENSG00000271893 | -3.65109 | 0.028145 |
| KDM7A-DT | ENSG00000260231 | -0.63816 | 0.028271 |
| AP003071.2 | ENSG00000261070 | 1.167121 | 0.028285 |
| AC097381.1 | ENSG00000228919 | 0.862055 | 0.028316 |
| AC012485.2 | ENSG00000232306 | -1.83184 | 0.028326 |
| AC037487.2 | ENSG00000266598 | 1.087168 | 0.028425 |
| AC012442.3 | ENSG00000243389 | 0.585513 | 0.028495 |
| AC016831.5 | ENSG00000271522 | -1.59917 | 0.028595 |
| LINC00698 | ENSG00000244342 | -2.86812 | 0.028619 |
| AL008718.2 | ENSG00000273287 | 0.958309 | 0.028636 |
| AC025188.1 | ENSG00000225407 | 1.125431 | 0.028667 |
| Z82246.1 | ENSG00000236054 | 1.336034 | 0.028766 |
| AC018730.1 | ENSG00000269707 | -4.53848 | 0.028852 |
| LINC02751 | ENSG00000254946 | -4.3752 | 0.029082 |
| AC006372.2 | ENSG00000233191 | -2.31511 | 0.029203 |
| AL355773.1 | ENSG00000258784 | -1.49696 | 0.029264 |
| ID2-AS1 | ENSG00000235092 | 0.677038 | 0.029339 |
| RNFT1-DT | ENSG00000267302 | -0.81094 | 0.029443 |
| LINC00470 | ENSG00000132204 | 1.17075 | 0.029453 |
| AC091544.4 | ENSG00000260337 | -0.92886 | 0.029653 |
| BX323046.1 | ENSG00000273175 | 0.809099 | 0.029729 |
| LINC02611 | ENSG00000226791 | 0.644596 | 0.02976 |
| HOTAIR | ENSG00000228630 | -3.21812 | 0.02982 |
| AL359397.1 | ENSG00000258857 | -1.15062 | 0.029884 |
| LINC02409 | ENSG00000257920 | 1.268286 | 0.030069 |
| AC100800.1 | ENSG00000187229 | 1.107394 | 0.0302 |
| AC093627.7 | ENSG00000261795 | -1.51264 | 0.030201 |
| AC025171.5 | ENSG00000272382 | -0.80456 | 0.030418 |
| DLG3-AS1 | ENSG00000231651 | -1.07837 | 0.030538 |
| AC010491.1 | ENSG00000261360 | -0.60045 | 0.030594 |
| AP005120.1 | ENSG00000263952 | -2.60743 | 0.030603 |
| AL583722.4 | ENSG00000258858 | 1.182709 | 0.030728 |
| ERVE-1 | ENSG00000267259 | -1.44514 | 0.030827 |
| LINC02167 | ENSG00000261122 | 1.393058 | 0.031137 |
| ARHGEF2-AS1 | ENSG00000224276 | -1.2267 | 0.031142 |
| FAM66B | ENSG00000215374 | 0.66897 | 0.031152 |
| AC092535.4 | ENSG00000251652 | 1.083566 | 0.031407 |
| AL133492.1 | ENSG00000275139 | 1.027028 | 0.03144 |
| AP000253.1 | ENSG00000234509 | 0.593777 | 0.031654 |
| AC100788.1 | ENSG00000267491 | 0.88739 | 0.031674 |
| LINC00645 | ENSG00000258548 | -2.86382 | 0.031772 |
| LINC02474 | ENSG00000228437 | 1.464919 | 0.031875 |
| AC239809.3 | ENSG00000227733 | 0.579134 | 0.031876 |
| LINC02303 | ENSG00000258616 | 1.122882 | 0.031893 |
| AC004923.1 | ENSG00000254610 | -1.21888 | 0.031905 |
| SRGAP3-AS2 | ENSG00000228723 | -1.87846 | 0.032111 |
| AC092834.1 | ENSG00000249453 | -2.84667 | 0.032129 |
| AC009118.2 | ENSG00000276131 | 0.668867 | 0.032132 |
| DSCR4-IT1 | ENSG00000223608 | 1.70698 | 0.032187 |
| AP001596.1 | ENSG00000232692 | -1.68471 | 0.032504 |
| AC090971.3 | ENSG00000259201 | 0.683816 | 0.032624 |
| LINC01267 | ENSG00000251576 | -2.11626 | 0.032666 |
| MED15P9 | ENSG00000223760 | 1.562122 | 0.032696 |
| LINC02663 | ENSG00000228636 | 2.020115 | 0.032781 |
| FAM230I | ENSG00000178248 | -1.31736 | 0.03294 |
| AP005137.2 | ENSG00000272703 | -1.42756 | 0.033 |
| LINC01733 | ENSG00000226203 | 1.100118 | 0.033362 |
| AC093866.1 | ENSG00000251095 | -2.29016 | 0.033383 |
| AL356804.1 | ENSG00000259033 | -3.67216 | 0.03354 |
| AC244502.3 | ENSG00000259042 | 1.089898 | 0.033618 |
| AC025263.1 | ENSG00000247131 | -0.8685 | 0.03363 |
| AC068025.1 | ENSG00000264808 | -1.14975 | 0.03363 |
| SMIM25 | ENSG00000224397 | -0.79111 | 0.033674 |
| SCARNA9 | ENSG00000254911 | 0.606039 | 0.033752 |
| AC007786.2 | ENSG00000276251 | 0.837759 | 0.033758 |
| AC025171.1 | ENSG00000177738 | -0.6489 | 0.033831 |
| AC004917.1 | ENSG00000243797 | -1.53254 | 0.033915 |
| AL133370.1 | ENSG00000258837 | -1.93832 | 0.034025 |
| AC011676.3 | ENSG00000254197 | 1.151778 | 0.034095 |
| AC026992.1 | ENSG00000259504 | 1.132332 | 0.034155 |
| SPON1-AS1 | ENSG00000254418 | -0.83797 | 0.034394 |
| FLVCR1-DT | ENSG00000198468 | -0.64084 | 0.034428 |
| AL121827.1 | ENSG00000203900 | -2.75066 | 0.034519 |
| AC027763.2 | ENSG00000263316 | -0.80214 | 0.034596 |
| BRWD1-AS1 | ENSG00000238141 | 0.649365 | 0.034669 |
| RNU6ATAC35P | ENSG00000221571 | 0.885504 | 0.034684 |
| AC005845.1 | ENSG00000255775 | 0.947728 | 0.034786 |
| LINC01750 | ENSG00000231437 | -1.18444 | 0.03482 |
| AL442224.1 | ENSG00000231990 | -2.35957 | 0.034842 |
| LNCOG | ENSG00000257219 | 0.792865 | 0.034962 |
| AC018755.4 | ENSG00000273837 | -1.08959 | 0.034995 |
| AP000866.1 | ENSG00000245498 | 0.509594 | 0.035188 |
| AC010478.1 | ENSG00000259663 | 1.230242 | 0.035201 |
| AL441992.2 | ENSG00000223478 | -0.63572 | 0.035201 |
| AL109930.1 | ENSG00000271774 | -0.85244 | 0.035271 |
| AP001972.4 | ENSG00000255395 | 1.290361 | 0.035281 |
| PTCSC3 | ENSG00000259104 | 1.402013 | 0.035323 |
| LINC01299 | ENSG00000254081 | -2.80004 | 0.035326 |
| AC003035.2 | ENSG00000233535 | -1.76933 | 0.035339 |
| AC092620.2 | ENSG00000260059 | -1.25694 | 0.035408 |
| LINC01978 | ENSG00000262188 | -0.90316 | 0.035513 |
| TSPEAR-AS1 | ENSG00000235890 | 1.309158 | 0.035607 |
| AC087301.1 | ENSG00000265010 | -0.76787 | 0.035672 |
| AC040173.2 | ENSG00000263257 | -1.05267 | 0.03582 |
| AL050344.1 | ENSG00000233755 | -1.7956 | 0.035843 |
| AL391056.1 | ENSG00000227619 | -1.41564 | 0.036027 |
| PI4KAP1 | ENSG00000274602 | -0.82123 | 0.036031 |
| AP000866.5 | ENSG00000255045 | 0.836408 | 0.036098 |
| AC004160.1 | ENSG00000230333 | 0.768945 | 0.036108 |
| AC022509.3 | ENSG00000256894 | -0.94982 | 0.036135 |
| AL365181.4 | ENSG00000272971 | -2.15834 | 0.036153 |
| AP005131.1 | ENSG00000267136 | 0.826659 | 0.036168 |
| AC110285.2 | ENSG00000262877 | 1.098951 | 0.036237 |
| ACTA2-AS1 | ENSG00000180139 | 0.855837 | 0.036307 |
| SIRLNT | ENSG00000253802 | -4.05944 | 0.036341 |
| AC002519.1 | ENSG00000259810 | -1.36526 | 0.03639 |
| MEIS1-AS3 | ENSG00000226819 | 0.881815 | 0.036473 |
| AP000842.3 | ENSG00000260209 | 0.944979 | 0.036492 |
| PGM5P4-AS1 | ENSG00000231943 | 1.092023 | 0.036523 |
| AC005162.1 | ENSG00000233517 | 0.910383 | 0.036661 |
| AC008443.1 | ENSG00000233937 | -0.49815 | 0.036833 |
| OR2A1-AS1 | ENSG00000244479 | -0.69188 | 0.036904 |
| AC092757.3 | ENSG00000277144 | -0.83556 | 0.036993 |
| AC025154.2 | ENSG00000257588 | -0.9122 | 0.037023 |
| LINC01525 | ENSG00000235202 | -1.62762 | 0.037263 |
| AC090241.3 | ENSG00000274776 | -0.83157 | 0.037326 |
| AC093677.2 | ENSG00000269559 | 0.686186 | 0.03735 |
| AL023581.2 | ENSG00000227192 | -0.82805 | 0.037433 |
| AP000439.1 | ENSG00000255606 | 0.987708 | 0.037545 |
| AC090197.1 | ENSG00000253837 | 0.517056 | 0.037753 |
| AC027601.1 | ENSG00000185332 | 1.233131 | 0.037798 |
| AP002884.3 | ENSG00000268472 | 0.835133 | 0.037845 |
| AC104667.1 | ENSG00000227107 | 0.690565 | 0.038036 |
| AC117395.1 | ENSG00000242791 | -1.0148 | 0.038042 |
| AC073316.2 | ENSG00000231892 | 1.0411 | 0.038066 |
| LINC02588 | ENSG00000257842 | 1.66531 | 0.03816 |
| TBX5-AS1 | ENSG00000255399 | 1.160482 | 0.038192 |
| LINC00520 | ENSG00000258791 | -1.33632 | 0.038215 |
| AP000812.1 | ENSG00000204971 | 0.877206 | 0.038241 |
| LINC02122 | ENSG00000248474 | -2.9106 | 0.038419 |
| AP002770.1 | ENSG00000256034 | 0.732841 | 0.038557 |
| LINC01858 | ENSG00000261615 | 0.898588 | 0.038618 |
| LINC02743 | ENSG00000255258 | 1.472214 | 0.038771 |
| AL161645.1 | ENSG00000278518 | 0.780347 | 0.038775 |
| LINC00612 | ENSG00000214851 | 0.577403 | 0.038792 |
| AC004771.1 | ENSG00000234203 | 0.657628 | 0.038798 |
| AC007686.2 | ENSG00000258473 | -1.97157 | 0.038817 |
| LINC00885 | ENSG00000224652 | -1.42712 | 0.038888 |
| AC000032.1 | ENSG00000260246 | 1.329684 | 0.038941 |
| LINC00639 | ENSG00000259070 | -1.26132 | 0.039005 |
| AC016629.2 | ENSG00000269600 | -2.13289 | 0.039022 |
| AP001010.1 | ENSG00000266954 | 0.801631 | 0.03908 |
| AC104667.2 | ENSG00000234949 | 0.616585 | 0.039222 |
| AC025171.3 | ENSG00000251131 | -0.98093 | 0.039331 |
| AC002551.1 | ENSG00000261736 | 0.749091 | 0.039413 |
| LINC02763 | ENSG00000254968 | -1.07235 | 0.039436 |
| MGAT4FP | ENSG00000234761 | -3.41854 | 0.039684 |
| AC090204.1 | ENSG00000247134 | 0.812787 | 0.039724 |
| AC125494.2 | ENSG00000269892 | 0.697133 | 0.039783 |
| AC254633.1 | ENSG00000272482 | -0.96933 | 0.039882 |
| MAP4K3-DT | ENSG00000231312 | -0.46403 | 0.039988 |
| AC092720.1 | ENSG00000260750 | 0.707966 | 0.040017 |
| LINC02821 | ENSG00000257139 | 0.902379 | 0.040062 |
| LINC01705 | ENSG00000232679 | -1.48269 | 0.04014 |
| SNAP47-AS1 | ENSG00000230005 | 0.871223 | 0.040311 |
| AL606519.1 | ENSG00000225598 | -2.08436 | 0.040343 |
| IMPA1P1 | ENSG00000251521 | -1.75363 | 0.040569 |
| LINC02754 | ENSG00000251637 | -1.11353 | 0.040646 |
| PCOTH | ENSG00000205861 | -0.77274 | 0.040787 |
| AL512363.1 | ENSG00000224984 | -1.7734 | 0.040832 |
| LINC01588 | ENSG00000214900 | -0.63511 | 0.040925 |
| AL355353.2 | ENSG00000270761 | -0.67396 | 0.04094 |
| AC027277.2 | ENSG00000260310 | 0.941596 | 0.040953 |
| PKP4-AS1 | ENSG00000204380 | -0.66582 | 0.041025 |
| P2RX6P | ENSG00000206145 | -1.85975 | 0.041087 |
| AC022730.4 | ENSG00000253967 | -2.31253 | 0.04109 |
| AC015802.3 | ENSG00000267543 | -0.54788 | 0.041107 |
| AP001207.3 | ENSG00000254024 | -1.18774 | 0.041152 |
| WARS2-IT1 | ENSG00000224238 | 0.695226 | 0.041211 |
| HECW2-AS1 | ENSG00000229056 | 0.922547 | 0.041231 |
| AP003066.1 | ENSG00000254587 | -3.37115 | 0.041294 |
| AC105105.4 | ENSG00000276403 | -3.16713 | 0.041617 |
| AC022034.3 | ENSG00000253369 | -2.07659 | 0.04165 |
| FOXP1-IT1 | ENSG00000242094 | 0.632905 | 0.041667 |
| OR7E28P | ENSG00000251491 | -1.34678 | 0.041679 |
| AC139887.2 | ENSG00000249592 | 0.421198 | 0.041701 |
| AC006262.3 | ENSG00000269745 | 1.619789 | 0.041883 |
| Z93930.2 | ENSG00000226471 | -0.52919 | 0.041888 |
| SIGLEC17P | ENSG00000171101 | 0.867923 | 0.041898 |
| HCG14 | ENSG00000224157 | -0.84204 | 0.041934 |
| AL356364.1 | ENSG00000238232 | -2.86499 | 0.041958 |
| AC023906.4 | ENSG00000259709 | 0.784857 | 0.042086 |
| AC093274.1 | ENSG00000249359 | -1.09983 | 0.042191 |
| MRGPRF-AS1 | ENSG00000256508 | 0.751442 | 0.042227 |
| AC119677.1 | ENSG00000229528 | -1.40227 | 0.042266 |
| AC089998.2 | ENSG00000257837 | 1.508993 | 0.04239 |
| C10orf91 | ENSG00000180066 | -1.09482 | 0.042619 |
| AC009041.3 | ENSG00000276931 | 0.726338 | 0.04273 |
| AC073578.1 | ENSG00000256209 | 1.069933 | 0.042748 |
| DSCR4 | ENSG00000184029 | 1.792751 | 0.042876 |
| AP001831.1 | ENSG00000254733 | -2.18154 | 0.043069 |
| AL357093.2 | ENSG00000258752 | -2.27714 | 0.043098 |
| GUSBP6 | ENSG00000224458 | -2.2063 | 0.043175 |
| AC005730.3 | ENSG00000266126 | 0.599462 | 0.043443 |
| CES5AP1 | ENSG00000215478 | -1.18841 | 0.043484 |
| AC018953.1 | ENSG00000253583 | 1.552888 | 0.043499 |
| AL354977.1 | ENSG00000226669 | -1.28887 | 0.043568 |
| AP000919.4 | ENSG00000272625 | 0.648973 | 0.043632 |
| AP005329.1 | ENSG00000264235 | 0.475104 | 0.043725 |
| AC099568.2 | ENSG00000272931 | -0.61232 | 0.04374 |
| LINC00968 | ENSG00000246430 | 0.861783 | 0.043853 |
| AC096741.1 | ENSG00000251171 | 0.981951 | 0.043941 |
| AC015802.5 | ENSG00000272386 | -0.65001 | 0.043954 |
| AC093843.1 | ENSG00000224819 | -2.17469 | 0.044011 |
| LINC01686 | ENSG00000261504 | 0.671452 | 0.044247 |
| AC022306.3 | ENSG00000276593 | 0.881743 | 0.044281 |
| AC100872.2 | ENSG00000263443 | -1.41589 | 0.044348 |
| AL354726.1 | ENSG00000236896 | -1.32672 | 0.044367 |
| AC092894.1 | ENSG00000243081 | -1.81274 | 0.044478 |
| AL603840.1 | ENSG00000234810 | -1.88359 | 0.044497 |
| AL355336.2 | ENSG00000225092 | 0.722659 | 0.044569 |
| AC010336.5 | ENSG00000269139 | -0.94102 | 0.044996 |
| CCDC13-AS1 | ENSG00000173811 | -0.73573 | 0.045062 |
| LINC00525 | ENSG00000146666 | -1.45689 | 0.045128 |
| AC012213.1 | ENSG00000253477 | -1.73956 | 0.045187 |
| LINC01829 | ENSG00000236780 | -1.19981 | 0.045418 |
| IDI2-AS1 | ENSG00000232656 | 0.656556 | 0.045454 |
| AC067930.2 | ENSG00000254812 | -0.81949 | 0.045468 |
| AC015802.4 | ENSG00000267546 | -0.52499 | 0.045514 |
| ZNF876P | ENSG00000198155 | 0.549952 | 0.045581 |
| HLA-V | ENSG00000181126 | -1.12644 | 0.045674 |
| PTCHD1-AS | ENSG00000233067 | -1.49558 | 0.045781 |
| FAM242C | ENSG00000250432 | -1.36448 | 0.045785 |
| AC084757.2 | ENSG00000259469 | 0.86821 | 0.045863 |
| MIR3659HG | ENSG00000224592 | -1.43965 | 0.046094 |
| AC120498.9 | ENSG00000274751 | 0.571345 | 0.046101 |
| AC010198.1 | ENSG00000246331 | -1.09062 | 0.046173 |
| AL121895.2 | ENSG00000278035 | -0.84337 | 0.046577 |
| AC005307.1 | ENSG00000260725 | -1.93672 | 0.046588 |
| AC011468.5 | ENSG00000275055 | 0.562897 | 0.046684 |
| AL009178.2 | ENSG00000269155 | -1.14833 | 0.046684 |
| LINC01197 | ENSG00000248441 | 0.657479 | 0.046722 |
| CDKN2A-DT | ENSG00000224854 | -1.18105 | 0.046768 |
| AC008551.1 | ENSG00000263331 | 1.32892 | 0.046783 |
| AL136234.1 | ENSG00000224326 | -1.99223 | 0.046949 |
| AC083809.1 | ENSG00000277247 | -1.70692 | 0.047069 |
| LINC02345 | ENSG00000259225 | -1.04555 | 0.047081 |
| AC069366.2 | ENSG00000265801 | 1.048384 | 0.047173 |
| HAND2-AS1 | ENSG00000237125 | 0.830364 | 0.047311 |
| TEX41 | ENSG00000226674 | 0.938046 | 0.047409 |
| AC078860.2 | ENSG00000257761 | -1.31127 | 0.047539 |
| LINC00667 | ENSG00000263753 | 0.421964 | 0.047593 |
| FAM86C2P | ENSG00000160172 | 0.452033 | 0.047627 |
| FP236241.2 | ENSG00000277991 | 0.720904 | 0.047648 |
| AC108448.1 | ENSG00000234791 | 1.531157 | 0.047811 |
| AC068722.1 | ENSG00000259200 | -2.11283 | 0.047826 |
| LINC00615 | ENSG00000196243 | -1.71104 | 0.048074 |
| LINC01623 | ENSG00000225595 | 0.914646 | 0.048219 |
| AC091946.2 | ENSG00000272130 | 0.885073 | 0.048421 |
| LINC02004 | ENSG00000240006 | -1.00426 | 0.048503 |
| ZDHHC8P1 | ENSG00000133519 | -0.81453 | 0.048524 |
| DIRC3 | ENSG00000231672 | 0.779021 | 0.048535 |
| AC093817.1 | ENSG00000249275 | -1.31152 | 0.048599 |
| KIR3DX1 | ENSG00000104970 | -0.99645 | 0.048601 |
| AC110048.2 | ENSG00000277152 | 0.650594 | 0.048604 |
| LINC01968 | ENSG00000237222 | -0.99553 | 0.048678 |
| LINC02228 | ENSG00000251273 | -1.76765 | 0.048839 |
| AC021549.2 | ENSG00000266521 | 0.915183 | 0.048883 |
| AC100793.2 | ENSG00000267042 | 0.684093 | 0.048923 |
| AC025171.2 | ENSG00000215068 | -0.76509 | 0.04897 |
| AC087633.1 | ENSG00000258433 | -3.83004 | 0.04928 |
| HDHD5-AS1 | ENSG00000185837 | -0.67691 | 0.049284 |
| AC004846.1 | ENSG00000258376 | 0.813934 | 0.049456 |
| AC092118.2 | ENSG00000276166 | 0.545768 | 0.049495 |
| LINC02816 | ENSG00000225857 | -1.10463 | 0.049542 |
| LINC02864 | ENSG00000263711 | -3.5001 | 0.049614 |
| AC087071.2 | ENSG00000240571 | -0.73902 | 0.049632 |
| AC136424.2 | ENSG00000253811 | 0.954612 | 0.049843 |
| AC099548.2 | ENSG00000253882 | -0.76952 | 0.049954 |

**Table S7**. Differently expression miRNA between Carboplatin resistant groups and Carboplatin nonresistant groups.

| miRNA | logFC | PValue |
| --- | --- | --- |
| hsa-mir-219a-2 | 4.938594 | 1.06E-08 |
| hsa-mir-101-1 | -3.78909 | 6.79E-06 |
| hsa-mir-101-2 | -3.77989 | 7.46E-06 |
| hsa-mir-486-1 | 1.240489 | 3.28E-05 |
| hsa-mir-374a | -4.46206 | 4.67E-05 |
| hsa-mir-486-2 | 1.218948 | 5.76E-05 |
| hsa-mir-184 | -3.3433 | 0.000113 |
| hsa-mir-135b | -2.74471 | 0.000172 |
| hsa-mir-19b-1 | -2.77023 | 0.000391 |
| hsa-mir-20a | -2.36168 | 0.000478 |
| hsa-mir-193b | 0.74558 | 0.000511 |
| hsa-mir-19b-2 | -3.91535 | 0.000592 |
| hsa-mir-151a | -0.94026 | 0.000802 |
| hsa-mir-141 | -1.49273 | 0.001311 |
| hsa-mir-1270 | 1.207226 | 0.001433 |
| hsa-mir-429 | -1.47686 | 0.001924 |
| hsa-mir-516a-2 | -2.56877 | 0.00197 |
| hsa-mir-374b | -1.36633 | 0.002191 |
| hsa-mir-3940 | 0.959248 | 0.002366 |
| hsa-mir-375 | 1.42084 | 0.002407 |
| hsa-mir-708 | -1.85449 | 0.00245 |
| hsa-mir-4433a | 1.562698 | 0.002507 |
| hsa-mir-29c | -1.20588 | 0.002633 |
| hsa-mir-30b | -0.98127 | 0.002955 |
| hsa-mir-30e | -0.81503 | 0.003093 |
| hsa-mir-582 | -2.08783 | 0.003366 |
| hsa-mir-140 | 0.757355 | 0.003745 |
| hsa-mir-1292 | 0.611059 | 0.003857 |
| hsa-mir-219a-1 | 0.835937 | 0.004046 |
| hsa-mir-96 | -1.87006 | 0.004226 |
| hsa-mir-206 | -3.35495 | 0.004481 |
| hsa-mir-26a-2 | -0.88943 | 0.004575 |
| hsa-mir-98 | -0.94639 | 0.004599 |
| hsa-mir-200b | -0.85961 | 0.004746 |
| hsa-mir-26a-1 | -0.89253 | 0.005072 |
| hsa-mir-542 | -1.64691 | 0.005361 |
| hsa-mir-19a | -3.78714 | 0.005885 |
| hsa-mir-21 | -1.1673 | 0.006944 |
| hsa-mir-516a-1 | -1.50084 | 0.007037 |
| hsa-let-7i | -0.68957 | 0.007454 |
| hsa-mir-10b | -1.21059 | 0.008337 |
| hsa-let-7f-2 | -1.042 | 0.008681 |
| hsa-mir-6849 | 1.218419 | 0.008781 |
| hsa-let-7f-1 | -1.02221 | 0.009928 |
| hsa-mir-514a-2 | -2.50128 | 0.010727 |
| hsa-mir-143 | -0.8953 | 0.010906 |
| hsa-mir-454 | -1.38383 | 0.010983 |
| hsa-mir-889 | -1.82788 | 0.011474 |
| hsa-mir-320d-2 | -1.22917 | 0.011929 |
| hsa-mir-3913-1 | -1.60472 | 0.012124 |
| hsa-mir-24-2 | -0.59767 | 0.012328 |
| hsa-mir-99a | -0.93855 | 0.01258 |
| hsa-mir-450b | -2.89753 | 0.012667 |
| hsa-mir-26b | -0.8002 | 0.012727 |
| hsa-mir-17 | -0.9224 | 0.013508 |
| hsa-mir-518b | 1.783602 | 0.014089 |
| hsa-mir-24-1 | -0.55681 | 0.014342 |
| hsa-let-7g | -0.71827 | 0.015146 |
| hsa-mir-4788 | 1.142206 | 0.016193 |
| hsa-mir-514a-1 | -2.38571 | 0.016789 |
| hsa-mir-7-1 | -0.81845 | 0.01866 |
| hsa-mir-6509 | -0.74646 | 0.020018 |
| hsa-mir-379 | -1.01696 | 0.020635 |
| hsa-mir-10a | -1.00126 | 0.021402 |
| hsa-mir-128-2 | -0.68119 | 0.022373 |
| hsa-mir-514a-3 | -2.30001 | 0.02299 |
| hsa-mir-6730 | -1.08345 | 0.02427 |
| hsa-mir-182 | -0.95167 | 0.024801 |
| hsa-mir-148a | -1.0104 | 0.025124 |
| hsa-mir-521-1 | -1.66399 | 0.025569 |
| hsa-mir-490 | -1.98188 | 0.02592 |
| hsa-mir-411 | -1.18847 | 0.028076 |
| hsa-mir-590 | -2.56657 | 0.02831 |
| hsa-mir-130a | -0.77694 | 0.028467 |
| hsa-mir-1245a | 0.995449 | 0.028495 |
| hsa-mir-588 | -1.40664 | 0.029038 |
| hsa-mir-369 | -1.00156 | 0.030959 |
| hsa-mir-5579 | -1.91276 | 0.032679 |
| hsa-mir-142 | -1.17161 | 0.032933 |
| hsa-mir-136 | -1.19609 | 0.03402 |
| hsa-mir-27b | -0.56121 | 0.03431 |
| hsa-mir-4661 | 0.617998 | 0.034864 |
| hsa-mir-23a | -0.53177 | 0.035379 |
| hsa-mir-29b-1 | -1.01587 | 0.035827 |
| hsa-mir-4784 | -1.06417 | 0.036461 |
| hsa-mir-662 | -1.13561 | 0.037034 |
| hsa-mir-4732 | 0.839962 | 0.039911 |
| hsa-mir-4647 | -1.0798 | 0.040724 |
| hsa-mir-221 | -0.58542 | 0.041369 |
| hsa-mir-195 | -0.66298 | 0.044813 |
| hsa-mir-144 | -1.69564 | 0.045522 |
| hsa-mir-511 | -0.81878 | 0.04591 |
| hsa-mir-4488 | -2.09217 | 0.048415 |
| hsa-mir-449b | 1.187012 | 0.049085 |
| hsa-mir-135a-1 | -1.32948 | 0.049736 |
| hsa-mir-5581 | -0.94769 | 0.049977 |

**Table S8**. Differently expression mRNA between Carboplatin resistant groups and Carboplatin nonresistant groups.

| mRNA | logFC | PValue |
| --- | --- | --- |
| GAL | 4.591056 | 5.43E-22 |
| PAPPA2 | 3.587674 | 4.41E-19 |
| INSL6 | 4.939971 | 8.20E-19 |
| CFC1 | 6.008247 | 2.36E-17 |
| OC90 | 6.780409 | 2.41E-17 |
| YIPF7 | 3.638402 | 6.82E-17 |
| SHC4 | 2.605144 | 1.28E-15 |
| PYGM | 3.379265 | 4.97E-15 |
| CYP2B6 | 4.964842 | 7.37E-15 |
| VSX2 | 4.189459 | 9.48E-15 |
| PNLDC1 | 3.655247 | 1.69E-14 |
| NR4A3 | 3.10029 | 5.81E-14 |
| CARTPT | 6.162377 | 7.08E-14 |
| SLC35F4 | 3.424004 | 3.78E-13 |
| TMEM189-UBE2V1 | 3.553946 | 9.22E-13 |
| TMC2 | 2.795789 | 2.73E-12 |
| MMP10 | 3.929055 | 3.17E-12 |
| CTAG2 | 6.435086 | 9.33E-12 |
| SEPTIN14 | 4.836357 | 9.52E-12 |
| BHLHE23 | 4.55324 | 2.10E-11 |
| MEIS3 | 2.265006 | 5.41E-11 |
| NEUROG3 | 3.278006 | 1.08E-10 |
| SPATA22 | 3.311984 | 1.50E-10 |
| THSD1 | 1.663462 | 2.23E-10 |
| ANO4 | 2.550303 | 3.63E-10 |
| AZU1 | 3.072895 | 5.55E-10 |
| PRG4 | 2.667805 | 1.00E-09 |
| LIN28A | 2.777062 | 1.12E-09 |
| SLC17A6 | 5.200016 | 1.17E-09 |
| EDN3 | 4.274266 | 2.49E-09 |
| LRRC74A | 2.586367 | 2.63E-09 |
| ARC | 2.509331 | 6.30E-09 |
| KDR | 1.352347 | 6.77E-09 |
| OTX2 | 4.697039 | 7.67E-09 |
| VTN | 2.340126 | 1.38E-08 |
| TRPM8 | 2.594594 | 3.65E-08 |
| SCRG1 | 2.192916 | 4.75E-08 |
| SSTR2 | 1.649424 | 5.34E-08 |
| ACCSL | 3.243391 | 6.57E-08 |
| CHAD | 1.871102 | 6.81E-08 |
| ALOX12 | 1.637673 | 1.23E-07 |
| CHST8 | 2.530683 | 1.62E-07 |
| RHOXF1 | 2.855257 | 1.76E-07 |
| DLC1 | 1.429637 | 1.78E-07 |
| RGS4 | 1.823731 | 2.48E-07 |
| GAGE12H | 5.326323 | 5.31E-07 |
| TM4SF4 | 2.74706 | 7.78E-07 |
| HAS1 | 2.560892 | 8.24E-07 |
| NPFFR2 | 3.160153 | 9.00E-07 |
| ONECUT2 | 2.62273 | 9.11E-07 |
| XAGE3 | 3.688113 | 1.05E-06 |
| OR2C3 | 2.676291 | 1.14E-06 |
| TCEAL7 | 1.572202 | 1.16E-06 |
| CLEC3A | 3.54046 | 1.37E-06 |
| PCDHGA3 | 2.162854 | 1.66E-06 |
| WNT2B | 1.716665 | 1.82E-06 |
| BTBD17 | 3.41468 | 1.85E-06 |
| PDE1C | 1.712492 | 2.23E-06 |
| SMOC1 | 2.310932 | 2.26E-06 |
| DPYS | 2.096617 | 2.29E-06 |
| FBXL22 | 1.102879 | 3.03E-06 |
| ST8SIA3 | 3.805739 | 3.04E-06 |
| ZNF831 | 1.909012 | 3.21E-06 |
| FAM181B | 1.945602 | 3.77E-06 |
| NXPH1 | 2.867467 | 3.78E-06 |
| RUNX1T1 | 1.811292 | 4.35E-06 |
| HAS3 | 1.410286 | 4.84E-06 |
| ITGA8 | 1.250206 | 5.49E-06 |
| PAGE1 | 4.172717 | 6.80E-06 |
| NKX1-2 | 3.148321 | 6.93E-06 |
| SLC17A8 | 2.977368 | 7.06E-06 |
| MGAT4C | 2.248283 | 7.25E-06 |
| CNN1 | 1.553377 | 7.28E-06 |
| ASPN | 1.872954 | 7.41E-06 |
| XIRP1 | 2.190658 | 7.42E-06 |
| RAB3C | 2.04332 | 7.47E-06 |
| TBX5 | 2.366263 | 7.64E-06 |
| SPHKAP | 2.954272 | 8.38E-06 |
| SLC4A1 | 1.754441 | 9.06E-06 |
| TRIML1 | 3.323613 | 9.44E-06 |
| VAV3 | -2.71218 | 9.53E-06 |
| MRGPRF | 1.159516 | 1.04E-05 |
| HBA1 | 1.776015 | 1.04E-05 |
| OR1L8 | 2.154162 | 1.06E-05 |
| PCDHGA11 | 1.799032 | 1.09E-05 |
| MYH2 | 2.296605 | 1.25E-05 |
| TAGLN | 1.330811 | 1.27E-05 |
| APELA | 2.01624 | 1.29E-05 |
| KLF2 | 1.142686 | 1.53E-05 |
| IRX4 | 2.776876 | 1.54E-05 |
| LMOD1 | 1.396716 | 1.67E-05 |
| SIX1 | -3.34442 | 1.69E-05 |
| EFCAB3 | 2.95615 | 1.71E-05 |
| TESMIN | 0.969232 | 1.74E-05 |
| GEM | 1.40509 | 1.83E-05 |
| H4C13 | 1.936397 | 1.92E-05 |
| FAM83C | 2.475311 | 2.04E-05 |
| FOXE1 | 2.862514 | 2.08E-05 |
| ITIH3 | 1.331722 | 2.16E-05 |
| GLI3 | 1.189139 | 2.16E-05 |
| GLP2R | 1.640006 | 2.17E-05 |
| SLC6A20 | -3.24873 | 2.44E-05 |
| NTF3 | 1.963721 | 2.89E-05 |
| FOXS1 | 1.229051 | 3.07E-05 |
| DAND5 | 1.722884 | 3.10E-05 |
| HSPA12B | 1.063595 | 3.15E-05 |
| SERPINE1 | 1.775607 | 3.30E-05 |
| EFEMP2 | 0.898741 | 3.48E-05 |
| SLC22A8 | 2.700764 | 3.60E-05 |
| ILDR2 | 1.633551 | 4.18E-05 |
| MEIS2 | 1.397221 | 4.35E-05 |
| FNDC5 | 1.23399 | 4.49E-05 |
| OSR1 | 1.608148 | 4.51E-05 |
| WNT11 | 1.773321 | 4.57E-05 |
| DDX4 | 2.062103 | 4.58E-05 |
| TNFRSF8 | 1.441922 | 4.59E-05 |
| FXYD2 | 1.50384 | 4.73E-05 |
| DES | 1.620179 | 4.79E-05 |
| PRAP1 | 1.5511 | 4.87E-05 |
| GALNT15 | 1.368203 | 4.94E-05 |
| XPNPEP2 | 1.712184 | 5.29E-05 |
| KIF26B | 1.478546 | 5.76E-05 |
| NTRK3 | 2.111459 | 5.94E-05 |
| PTH1R | 1.507685 | 6.04E-05 |
| ADH1A | 1.783102 | 6.07E-05 |
| TGFB3 | 1.15809 | 6.07E-05 |
| CACNA1G | 1.453506 | 6.21E-05 |
| ADGRA2 | 1.119013 | 6.44E-05 |
| RXRG | 2.237733 | 7.21E-05 |
| SLC1A6 | 1.927763 | 7.46E-05 |
| POU3F3 | -6.13168 | 7.94E-05 |
| DKK1 | 2.127375 | 7.99E-05 |
| PAGE4 | 2.811449 | 8.87E-05 |
| RASGRP2 | 1.252253 | 9.03E-05 |
| LTO1 | 0.747182 | 9.06E-05 |
| NPAS4 | 1.787377 | 9.27E-05 |
| TSKS | 1.675522 | 9.31E-05 |
| SFRP4 | 1.690369 | 9.68E-05 |
| KCNC2 | 2.784217 | 9.94E-05 |
| SELE | 1.631317 | 1.00E-04 |
| NR4A1 | 1.431962 | 0.000103 |
| EPHA1 | -1.05923 | 0.000104 |
| MEDAG | 1.581854 | 0.000108 |
| CTRC | -7.14837 | 0.000108 |
| IQCF3 | 2.569894 | 0.000109 |
| ZDHHC22 | 2.016306 | 0.000111 |
| KRTAP10-2 | 1.926758 | 0.000114 |
| SLC51B | 1.875205 | 0.000114 |
| RIPOR3 | 1.084355 | 0.000124 |
| ZNF469 | 1.359705 | 0.000125 |
| PODN | 1.415209 | 0.000127 |
| CRISPLD2 | 1.453291 | 0.00013 |
| GHRL | 1.049234 | 0.000131 |
| GAGE1 | 3.962915 | 0.000132 |
| MSRB3 | 1.043783 | 0.000134 |
| MYH11 | 1.411155 | 0.000144 |
| C10orf105 | 1.660303 | 0.000145 |
| JPH4 | 1.530065 | 0.000147 |
| PTGIS | 1.474888 | 0.000155 |
| AKR1C4 | 2.743526 | 0.000155 |
| EN2 | -4.46444 | 0.000158 |
| DHH | 1.315661 | 0.000159 |
| AOC3 | 1.273988 | 0.000162 |
| APOA2 | 1.995361 | 0.000163 |
| BMP2 | 1.439932 | 0.000164 |
| GTSF1 | 2.333422 | 0.000164 |
| STRIP2 | 1.240633 | 0.000165 |
| CDR1 | 1.700918 | 0.000168 |
| IRX2 | 1.924022 | 0.000172 |
| FOXD1 | -4.15725 | 0.000173 |
| ST6GALNAC3 | 1.144207 | 0.000174 |
| CILP2 | 1.245655 | 0.000177 |
| IFITM5 | 1.914562 | 0.000189 |
| PIGR | -4.0222 | 0.000197 |
| ELN | 1.342512 | 0.000198 |
| FAM180A | 1.449021 | 0.000199 |
| UAP1L1 | 0.936499 | 0.00021 |
| ELK1 | 0.588016 | 0.000211 |
| ADAM2 | 2.66106 | 0.000218 |
| PROCR | 0.812062 | 0.000223 |
| FBN1 | 1.235896 | 0.000224 |
| FLRT2 | 1.340785 | 0.000227 |
| CD1E | 1.730871 | 0.000231 |
| HSPB7 | 1.308057 | 0.000236 |
| SSC5D | 1.303592 | 0.000238 |
| THBS4 | 1.366718 | 0.000238 |
| PRELID3A | 0.939902 | 0.00024 |
| C3orf22 | 1.524418 | 0.000245 |
| HPR | 1.455785 | 0.000254 |
| COL24A1 | 1.488886 | 0.000262 |
| SCGB1D1 | 2.128156 | 0.000265 |
| VAT1L | 1.501415 | 0.000276 |
| CCN1 | 1.102518 | 0.00028 |
| CLPS | -6.47754 | 0.000287 |
| AMPH | 1.541578 | 0.000293 |
| DACT3 | 1.091064 | 0.000307 |
| H4C6 | 1.756808 | 0.000309 |
| SYCP1 | 2.22009 | 0.000314 |
| ADRA1A | 1.829052 | 0.000319 |
| HIC1 | 0.983371 | 0.000321 |
| MUC12 | 1.847871 | 0.000322 |
| CADM2 | -3.86009 | 0.00034 |
| HMX3 | 3.43706 | 0.000342 |
| IFNL1 | 1.740114 | 0.000342 |
| GAGE10 | 1.886919 | 0.000344 |
| SPARC | 1.077245 | 0.000347 |
| ADGRD1 | 1.439741 | 0.00035 |
| SLC38A5 | 1.359033 | 0.000364 |
| ABCA8 | 1.300944 | 0.000381 |
| FBLN5 | 0.928156 | 0.000385 |
| DAB1 | 1.74785 | 0.000392 |
| EMILIN1 | 1.08122 | 0.000393 |
| CCDC198 | 2.644875 | 0.000414 |
| GLYATL2 | -4.1931 | 0.000422 |
| MUC6 | -3.46649 | 0.000425 |
| PRR26 | 1.316864 | 0.000425 |
| SPARCL1 | 0.762496 | 0.000427 |
| C20orf204 | 1.406325 | 0.000437 |
| SCN1A | 2.211294 | 0.000439 |
| ISX | 2.399489 | 0.000447 |
| NLRP4 | 2.460412 | 0.00045 |
| RBFOX3 | 1.781849 | 0.000453 |
| GPR101 | 2.386941 | 0.000454 |
| MYH8 | 2.173438 | 0.000458 |
| PAQR9 | 2.241292 | 0.000461 |
| FOSB | 1.781947 | 0.000471 |
| SRRM4 | 1.914246 | 0.000476 |
| LPIN2 | 0.67765 | 0.000487 |
| NTSR1 | 1.528678 | 0.000514 |
| F5 | 1.400579 | 0.000515 |
| VTCN1 | -1.83024 | 0.000521 |
| AEBP1 | 1.160926 | 0.000536 |
| PYY | -6.02804 | 0.000539 |
| ARHGEF40 | 0.780224 | 0.000541 |
| EREG | 1.67843 | 0.000555 |
| NLRP7 | 1.761834 | 0.000564 |
| ECM2 | 1.061054 | 0.000573 |
| PAGE2 | 3.256263 | 0.000578 |
| SP8 | -4.83978 | 0.000584 |
| FLNC | 1.396588 | 0.000588 |
| SOX21 | -5.51216 | 0.000589 |
| TCIRG1 | 0.895254 | 0.000609 |
| PAX9 | -3.59624 | 0.000616 |
| LOXL1 | 0.891903 | 0.000621 |
| TEX48 | -2.5467 | 0.000631 |
| TPCN2 | 0.699853 | 0.000632 |
| SPON2 | 0.89939 | 0.000636 |
| IGFN1 | 1.767304 | 0.000649 |
| NID2 | 1.111538 | 0.00066 |
| EPB42 | 1.266838 | 0.000673 |
| MUC5B | -2.59049 | 0.000674 |
| SVEP1 | 1.45355 | 0.0007 |
| LGI4 | 0.941166 | 0.000708 |
| INMT | 1.203481 | 0.000719 |
| DMD | 0.918234 | 0.000737 |
| VGF | 1.17826 | 0.000743 |
| OTOR | 2.02473 | 0.000759 |
| ANKRD42 | 0.769504 | 0.000783 |
| KRTAP5-10 | 1.22483 | 0.000805 |
| ZSCAN10 | 1.753177 | 0.000808 |
| MAP3K15 | 1.314472 | 0.000819 |
| COL16A1 | 1.04251 | 0.000821 |
| IGFBP6 | 1.113342 | 0.000828 |
| ACTA2 | 1.184051 | 0.000844 |
| ACOT6 | 1.352617 | 0.000852 |
| BGN | 0.970187 | 0.000871 |
| DCHS1 | 0.993598 | 0.000883 |
| SMOC2 | 1.008533 | 0.00089 |
| SELP | 1.187987 | 0.0009 |
| DGKK | -3.29134 | 0.00092 |
| CALB1 | -3.26247 | 0.000927 |
| RASSF2 | 0.765866 | 0.000942 |
| COMP | 1.648127 | 0.000944 |
| CACNA1C | 1.074288 | 0.000967 |
| MPPED1 | 2.034813 | 0.000969 |
| CNRIP1 | 0.812016 | 0.000981 |
| CCN2 | 1.106832 | 0.000982 |
| MEP1A | -5.58752 | 0.001 |
| C5orf58 | -2.98314 | 0.001008 |
| TPH2 | 1.720042 | 0.001017 |
| COL5A1 | 1.306359 | 0.001042 |
| GPR33 | 1.870991 | 0.001042 |
| S100A7 | -3.26506 | 0.001053 |
| TFAP2A | -1.68463 | 0.001061 |
| GASK1B | 0.82762 | 0.001077 |
| C4BPA | -2.6926 | 0.001086 |
| SLC23A1 | -1.91102 | 0.001093 |
| CALY | 1.523905 | 0.001096 |
| STXBP6 | -1.41459 | 0.001139 |
| ZNF81 | 0.675938 | 0.001152 |
| CAVIN1 | 0.848392 | 0.001164 |
| MGP | 0.942373 | 0.001167 |
| IGHMBP2 | 0.569015 | 0.001187 |
| PRR36 | -1.25356 | 0.001189 |
| LIPT2 | 0.789035 | 0.0012 |
| EFCAB8 | 1.363645 | 0.00121 |
| GATA1 | 1.172893 | 0.001214 |
| CEL | -3.38254 | 0.001238 |
| RBPJL | -4.65466 | 0.001244 |
| HGD | -2.39168 | 0.001263 |
| SLC9B2 | 0.875848 | 0.00128 |
| HES5 | 1.127711 | 0.001294 |
| SH3D19 | 0.647378 | 0.001296 |
| ABCA6 | 1.075648 | 0.001307 |
| ADAMTS2 | 1.158427 | 0.001327 |
| SIK1B | 1.01027 | 0.001355 |
| SPEF1 | 1.242792 | 0.001366 |
| CYP2C18 | -4.42214 | 0.00137 |
| CLMP | 1.073078 | 0.001386 |
| ZNF157 | 1.006243 | 0.001396 |
| PREX1 | 0.693339 | 0.001402 |
| NOG | 1.378754 | 0.001431 |
| AP006333.1 | 1.266599 | 0.001436 |
| CPA2 | -5.47597 | 0.00144 |
| DPY19L2 | 1.084158 | 0.001442 |
| FHL5 | 1.366796 | 0.001447 |
| OVGP1 | -3.63353 | 0.001461 |
| MMP2 | 1.082473 | 0.001462 |
| C10orf120 | 2.019362 | 0.001479 |
| ZEB1 | 0.818013 | 0.001486 |
| PDGFRB | 0.83972 | 0.001511 |
| ACTRT2 | 2.699146 | 0.001511 |
| MRVI1 | 1.011667 | 0.001517 |
| LRRC31 | -4.15774 | 0.001527 |
| CHST9 | -2.96376 | 0.001533 |
| P4HA3 | 0.973595 | 0.001535 |
| ZCCHC24 | 0.898761 | 0.001537 |
| OMD | 1.55189 | 0.001556 |
| DIO1 | -2.30009 | 0.001572 |
| FNDC1 | 1.249295 | 0.001587 |
| OR7D2 | -2.18454 | 0.001596 |
| FAM43B | 1.266508 | 0.001606 |
| COL1A1 | 1.177791 | 0.001609 |
| CBLN4 | 1.358235 | 0.00161 |
| NFATC2 | 0.764873 | 0.001617 |
| TMCC2 | 0.862871 | 0.001617 |
| TDRD1 | -3.18397 | 0.001623 |
| SCGB1D4 | 2.341565 | 0.001628 |
| NXF2B | 2.254846 | 0.001654 |
| GREM2 | 1.307571 | 0.001698 |
| TULP2 | 1.103618 | 0.001722 |
| PHYHIP | 0.841592 | 0.001757 |
| ARHGAP31 | 0.758579 | 0.001779 |
| BPIFB3 | -3.51788 | 0.00178 |
| HBB | 1.305635 | 0.001811 |
| DACT1 | 1.020382 | 0.001813 |
| THBS1 | 1.015119 | 0.001824 |
| PROC | 0.953363 | 0.001824 |
| PHACTR3 | 1.285586 | 0.001861 |
| MUC2 | -3.79627 | 0.001864 |
| ARSI | 1.233378 | 0.001874 |
| COL8A1 | 1.276767 | 0.001884 |
| PPP6R3 | 0.430222 | 0.001903 |
| P3H4 | 0.524452 | 0.001917 |
| SRPX2 | 0.927461 | 0.001926 |
| ZCCHC12 | 1.414762 | 0.001928 |
| PUM3 | -0.59595 | 0.00193 |
| CLEC2A | 2.067204 | 0.001938 |
| TLX3 | 2.156138 | 0.001954 |
| SAMD10 | 0.699281 | 0.001973 |
| CCIN | 1.264765 | 0.001979 |
| DLX1 | -2.91053 | 0.001993 |
| LGI2 | 1.05365 | 0.001994 |
| TRIM67 | 0.922041 | 0.001995 |
| SYT4 | 1.736203 | 0.001999 |
| PIEZO2 | 1.274274 | 0.002 |
| HMX2 | 2.735376 | 0.002013 |
| CERCAM | 0.751133 | 0.002043 |
| RGR | -4.96204 | 0.002049 |
| COL3A1 | 1.186035 | 0.002056 |
| SLC16A11 | 1.253902 | 0.002079 |
| NLRP1 | 0.832457 | 0.002104 |
| SLC14A2 | 1.724268 | 0.002113 |
| LGALS12 | 1.260524 | 0.002121 |
| C1QTNF1 | 0.941106 | 0.002123 |
| CYSRT1 | 0.857797 | 0.002125 |
| ERMP1 | -0.66933 | 0.002172 |
| ATOH1 | 2.795821 | 0.002175 |
| MYRFL | -1.86819 | 0.002176 |
| IGF1 | 1.210111 | 0.002191 |
| H3C11 | 1.389781 | 0.002192 |
| JAM3 | 0.695947 | 0.002211 |
| GAGE2A | 3.161417 | 0.002259 |
| CSDC2 | 1.110051 | 0.002272 |
| PAX7 | -4.94447 | 0.002321 |
| PRSS3 | -2.34621 | 0.002328 |
| FUT6 | -2.60048 | 0.002339 |
| HOXB13 | -4.07026 | 0.002355 |
| ALX4 | -3.29698 | 0.002396 |
| NPY1R | -3.24497 | 0.002403 |
| C1QL4 | 1.581853 | 0.002409 |
| PAX6 | -1.76047 | 0.002417 |
| FOXC1 | -1.73982 | 0.002426 |
| HOXC13 | -4.36137 | 0.002449 |
| CCDC80 | 1.166717 | 0.002451 |
| ENPP6 | 1.579457 | 0.00247 |
| ALPG | -3.32796 | 0.002472 |
| NEUROD2 | 1.577244 | 0.002483 |
| XYLT1 | 1.055397 | 0.002511 |
| PDX1 | -7.67763 | 0.002511 |
| RASL10B | 0.923927 | 0.002513 |
| C1orf167 | 1.248371 | 0.002527 |
| SPDYC | 1.555896 | 0.002548 |
| PPP1R12B | 0.687619 | 0.002551 |
| C1QTNF4 | 1.065272 | 0.002591 |
| AP1M2 | -0.74119 | 0.0026 |
| IFRD1 | -0.58598 | 0.002602 |
| CRYGD | -5.34201 | 0.002609 |
| KRT13 | 1.575047 | 0.002621 |
| CHKA | 0.617818 | 0.002623 |
| ST8SIA5 | -1.55328 | 0.002626 |
| EVA1B | 0.732621 | 0.002643 |
| TPMT | -0.57299 | 0.002645 |
| AGXT | -4.5985 | 0.002697 |
| NDRG1 | -0.91409 | 0.002724 |
| LRP1 | 0.786785 | 0.002733 |
| PIWIL1 | -2.02295 | 0.002746 |
| SPTSSB | -1.92041 | 0.002753 |
| C8B | 2.033656 | 0.002765 |
| RNF183 | -1.32174 | 0.002769 |
| PDE4C | -1.46407 | 0.002778 |
| HBA2 | 1.16321 | 0.002802 |
| COL1A2 | 0.994269 | 0.002802 |
| H1-5 | 1.319449 | 0.002848 |
| CHADL | 0.807347 | 0.002858 |
| MTMR9 | 0.536326 | 0.002863 |
| CSGALNACT1 | 0.649342 | 0.002883 |
| ID3 | 0.746553 | 0.002902 |
| IRX6 | 1.657862 | 0.002907 |
| CALCA | 1.87059 | 0.002908 |
| OLIG1 | 1.639918 | 0.002916 |
| HOXB1 | -4.39842 | 0.002917 |
| ADAMTS8 | 1.155916 | 0.002937 |
| RAPSN | 1.357465 | 0.002938 |
| C19orf84 | 1.227245 | 0.002982 |
| MMP14 | 0.816545 | 0.002995 |
| C3orf70 | 0.899189 | 0.003029 |
| GPR176 | 0.916522 | 0.003029 |
| NDUFA5 | -0.48038 | 0.003032 |
| TBC1D25 | 0.523575 | 0.003089 |
| FMO3 | -2.45858 | 0.003092 |
| ZNF460 | 0.77944 | 0.003106 |
| SCT | 1.079804 | 0.003114 |
| CXCL12 | 1.047627 | 0.003125 |
| PCP4L1 | -2.39903 | 0.003135 |
| PLGRKT | -0.68604 | 0.003136 |
| MAS1 | 1.540672 | 0.003158 |
| UNC13C | 1.403214 | 0.003188 |
| PAGE5 | -4.5481 | 0.00319 |
| PLN | 1.341649 | 0.003194 |
| WBP1L | 0.467672 | 0.003224 |
| FZD1 | 0.744857 | 0.003237 |
| DGKB | -2.77949 | 0.003279 |
| ZNF491 | 0.940645 | 0.00328 |
| TARS1 | -0.49018 | 0.003318 |
| DQX1 | 1.345267 | 0.003332 |
| AKR1C8P | 1.544921 | 0.003335 |
| CCDC116 | 0.946034 | 0.003341 |
| MNX1 | -2.77195 | 0.003351 |
| EBLN1 | 1.884355 | 0.0034 |
| RIMS1 | 1.344176 | 0.003409 |
| CHST14 | 0.504088 | 0.003415 |
| OR7G2 | -4.31971 | 0.003428 |
| PRLH | 1.79181 | 0.003428 |
| RAG1 | 0.783152 | 0.003444 |
| DBX1 | 2.026441 | 0.003469 |
| MSC | 1.119538 | 0.003471 |
| C2orf66 | -1.54327 | 0.003519 |
| MMP19 | 0.891123 | 0.00352 |
| TXK | -1.20456 | 0.003521 |
| C22orf15 | 1.191341 | 0.003521 |
| PIWIL3 | 2.077559 | 0.003537 |
| S100A2 | -1.56369 | 0.003544 |
| FBXO15 | 1.048219 | 0.003557 |
| UGT2B17 | -4.03255 | 0.00356 |
| ASNS | -0.57176 | 0.003571 |
| CD248 | 0.825156 | 0.00358 |
| OGN | 1.319117 | 0.003595 |
| LYPD2 | 1.706402 | 0.003597 |
| PRAC2 | -4.86777 | 0.003617 |
| COL15A1 | 0.731547 | 0.003626 |
| SHH | -2.87116 | 0.003652 |
| HOXA2 | 1.605182 | 0.003652 |
| CPT1C | 1.001116 | 0.003676 |
| CD8B2 | 1.369594 | 0.00368 |
| FAM187B | 1.431944 | 0.003685 |
| SLIT1 | 1.404166 | 0.003695 |
| DCD | -5.30438 | 0.003712 |
| SCD5 | 0.826936 | 0.003719 |
| LRRC52 | 1.560292 | 0.003725 |
| CASTOR2 | 0.780482 | 0.003735 |
| COL5A2 | 1.101133 | 0.003757 |
| TTC5 | 0.510591 | 0.003774 |
| DUSP2 | 0.765863 | 0.003796 |
| BMP4 | 1.05011 | 0.0038 |
| CD70 | -2.36185 | 0.003834 |
| MVB12B | 0.629887 | 0.003865 |
| PON1 | -2.4624 | 0.003875 |
| LRP5 | 0.541886 | 0.003879 |
| KCTD4 | 1.322799 | 0.003881 |
| MAGEA8 | -2.97439 | 0.0039 |
| IER3 | 0.850592 | 0.003905 |
| OLFM2 | -1.26944 | 0.003971 |
| FEZ1 | 0.745322 | 0.004004 |
| CYP4A22 | -2.10522 | 0.004014 |
| NAPEPLD | -0.56873 | 0.00404 |
| SULF2 | 0.951796 | 0.004044 |
| RECK | 0.648866 | 0.004053 |
| SLC2A1 | -0.84489 | 0.004111 |
| IGFL1 | 1.504692 | 0.004112 |
| INSL4 | 1.717533 | 0.004155 |
| ST8SIA1 | 0.941569 | 0.004209 |
| MEGF11 | -1.68954 | 0.004242 |
| ARMH4 | 0.790272 | 0.004243 |
| THRB | -0.95238 | 0.004247 |
| MRAP | 1.370504 | 0.004266 |
| CCL18 | -2.394 | 0.004266 |
| CTSK | 1.075347 | 0.004271 |
| LHFPL5 | -2.26093 | 0.004278 |
| NLRP5 | 2.010683 | 0.004306 |
| KLRF1 | -1.42524 | 0.004317 |
| ADAM12 | 1.143102 | 0.004342 |
| CCL14 | 1.25949 | 0.004343 |
| ETNPPL | -2.10026 | 0.004349 |
| EGR3 | 1.188149 | 0.004405 |
| H3C12 | 1.112383 | 0.004411 |
| TKTL2 | 1.376902 | 0.00442 |
| NUTM2D | 0.820726 | 0.004422 |
| MTIF3 | -0.45204 | 0.004431 |
| CDK15 | 1.085338 | 0.004433 |
| SPINK1 | -4.6861 | 0.004487 |
| RNF17 | -3.04036 | 0.004492 |
| RAD52 | 0.605216 | 0.004492 |
| PRR20G | 2.082188 | 0.004494 |
| AKNAD1 | 1.650718 | 0.004507 |
| WFDC13 | 1.386243 | 0.004515 |
| EBF2 | 1.183585 | 0.004515 |
| C17orf78 | 1.002766 | 0.004549 |
| TRIM43B | -2.66346 | 0.004608 |
| CCND1 | 0.985119 | 0.004612 |
| LMOD3 | -1.83397 | 0.004612 |
| CD302 | 0.68652 | 0.004642 |
| LUM | 1.197935 | 0.004715 |
| EGF | -1.99794 | 0.00473 |
| HSPG2 | 0.814295 | 0.004756 |
| TRIM55 | -2.30711 | 0.004758 |
| KCNJ8 | 0.741942 | 0.004795 |
| SFTPC | 1.478777 | 0.004803 |
| DRD5 | 1.386717 | 0.004836 |
| ANKRD53 | 0.859895 | 0.004843 |
| DDR2 | 0.840766 | 0.004874 |
| TMEM213 | -2.30802 | 0.004886 |
| TCL1A | 1.711084 | 0.004926 |
| PTCH2 | 0.810907 | 0.004988 |
| SMIM18 | 1.203951 | 0.004989 |
| LATS2 | 0.603206 | 0.004995 |
| TCF21 | 1.298028 | 0.005002 |
| DKK3 | 0.736451 | 0.005011 |
| CMTM3 | 0.51624 | 0.005019 |
| PPP1R3B | 0.689881 | 0.005073 |
| GAS2 | 1.250822 | 0.005098 |
| H2BC3 | 1.348544 | 0.005181 |
| CORO2A | -0.73717 | 0.005186 |
| PNMA8C | 1.442592 | 0.005285 |
| RPE65 | 1.563934 | 0.005299 |
| PRSS12 | -1.38028 | 0.005346 |
| FTH1 | -0.80737 | 0.005419 |
| BMP5 | -3.72224 | 0.005444 |
| CCNO | 1.136775 | 0.005447 |
| FFAR3 | 1.321216 | 0.00545 |
| BAALC | 0.976313 | 0.005454 |
| PLP1 | 1.388776 | 0.005457 |
| NGLY1 | -0.42429 | 0.005465 |
| APLNR | 0.811045 | 0.005465 |
| POMC | -2.55045 | 0.00552 |
| NECAB2 | 1.428232 | 0.005522 |
| AC115220.1 | 2.481589 | 0.005525 |
| SCARF2 | 0.731417 | 0.005604 |
| PLXND1 | 0.627156 | 0.005618 |
| NAV3 | 0.825957 | 0.005634 |
| ANKEF1 | 0.48736 | 0.005728 |
| HTR2A | 1.206487 | 0.005728 |
| BHLHE22 | 1.244691 | 0.00575 |
| JADE3 | 0.482524 | 0.005788 |
| THBS2 | 1.129893 | 0.005838 |
| TFF3 | -2.49152 | 0.005842 |
| HFM1 | 1.025176 | 0.005899 |
| ENG | 0.487136 | 0.005908 |
| CCL13 | -2.00838 | 0.005915 |
| ASPA | 1.17778 | 0.005927 |
| SEMA7A | 0.721098 | 0.005951 |
| VGLL3 | 1.114575 | 0.005952 |
| CDH11 | 0.911273 | 0.005959 |
| PCDHGA12 | 0.998464 | 0.005996 |
| PLD5 | 1.589275 | 0.006001 |
| PLIN4 | 0.977106 | 0.006012 |
| ZNF677 | 0.651262 | 0.006024 |
| ORM2 | -2.39982 | 0.006051 |
| RAPH1 | 0.687168 | 0.006056 |
| C9orf152 | -1.52705 | 0.006057 |
| ABCC4 | -0.79626 | 0.006064 |
| ADAMTS6 | 0.973116 | 0.00608 |
| TUBB4A | -1.54 | 0.006085 |
| SFN | -1.12019 | 0.006126 |
| PRKG1 | 0.848912 | 0.006129 |
| SCTR | 1.488252 | 0.006175 |
| CHST6 | -1.21513 | 0.006186 |
| LDLRAD2 | 0.711051 | 0.006204 |
| MYL3 | 1.078404 | 0.006212 |
| PRSS41 | 1.483887 | 0.006227 |
| IYD | -2.24665 | 0.006258 |
| HHIPL2 | 1.40393 | 0.00627 |
| TBATA | 1.522263 | 0.006278 |
| ANGPTL2 | 0.700432 | 0.006286 |
| TMPRSS15 | -1.83441 | 0.006355 |
| KANK4 | 1.156934 | 0.006368 |
| PI16 | 1.333556 | 0.006375 |
| VGLL2 | 1.582064 | 0.006385 |
| PLAA | -0.46066 | 0.006408 |
| DNAJB1 | -0.52254 | 0.006417 |
| CHRNA4 | -3.03936 | 0.006426 |
| MRC2 | 0.692803 | 0.006432 |
| MUCL1 | -2.49836 | 0.0065 |
| ATF3 | 0.989906 | 0.006614 |
| CENPV | 0.905757 | 0.006652 |
| H1-4 | 0.9859 | 0.006657 |
| PRSS1 | -2.10494 | 0.006668 |
| NRXN1 | -2.77475 | 0.006674 |
| RIN2 | 0.408766 | 0.0067 |
| UGT2B15 | -3.67005 | 0.006702 |
| GFRA2 | -2.26686 | 0.006708 |
| MRPL21 | 0.599429 | 0.006714 |
| RIMS2 | 1.449582 | 0.006718 |
| GADD45B | 0.730246 | 0.006781 |
| S100A6 | -1.1768 | 0.006795 |
| ANTXR2 | 0.733662 | 0.006859 |
| CSMD1 | 1.710204 | 0.006886 |
| TRIM50 | -1.97884 | 0.006928 |
| CXXC4 | 1.239605 | 0.006953 |
| SLC26A5 | -1.20874 | 0.006971 |
| TCF15 | 1.00005 | 0.007005 |
| MARVELD1 | 0.633363 | 0.007065 |
| ABCA12 | -1.52552 | 0.007139 |
| TPK1 | -1.24156 | 0.007147 |
| KPNA7 | -1.68879 | 0.007157 |
| OPN4 | -3.36371 | 0.007204 |
| PLA2G2A | 1.474844 | 0.00722 |
| ZNF182 | 0.479383 | 0.007235 |
| LEFTY1 | -2.53467 | 0.007263 |
| SARDH | 0.766887 | 0.007276 |
| SLITRK2 | -1.82018 | 0.007282 |
| CLPSL1 | -1.75496 | 0.0073 |
| OR2V1 | -2.78074 | 0.00733 |
| PPFIA2 | -2.00563 | 0.007335 |
| FEZF2 | 1.30101 | 0.007357 |
| PCOLCE | 0.734651 | 0.007364 |
| PLEKHS1 | -1.93517 | 0.007396 |
| NPB | 1.051662 | 0.007438 |
| ADGRF1 | -1.47569 | 0.007449 |
| ID2 | 0.630304 | 0.007453 |
| CFHR1 | 1.424026 | 0.007457 |
| SLC16A6 | -0.8149 | 0.007461 |
| BNIP3 | -0.75173 | 0.007461 |
| C10orf99 | -2.97606 | 0.007468 |
| ADM | -1.01747 | 0.007477 |
| PSMD12 | -0.44752 | 0.007483 |
| MT-ND1 | -0.6152 | 0.007484 |
| GAP43 | 1.204527 | 0.007535 |
| LIMS2 | 0.653315 | 0.007572 |
| ANAPC2 | 0.450008 | 0.007578 |
| RAB31 | 0.648893 | 0.007643 |
| ABCB11 | 1.421414 | 0.007665 |
| DUSP1 | 0.874293 | 0.007693 |
| NOX5 | -1.51197 | 0.00772 |
| DENND2A | 0.825989 | 0.007784 |
| NXPH3 | 0.736944 | 0.007806 |
| AGBL3 | -0.67567 | 0.007833 |
| C6orf223 | -1.29599 | 0.007842 |
| PID1 | 0.975037 | 0.007888 |
| PHLDA2 | -1.10827 | 0.007969 |
| LRTM2 | -2.68897 | 0.007979 |
| IGSF23 | -1.78707 | 0.008034 |
| TFAP2B | 2.002856 | 0.008063 |
| LHX3 | -4.74705 | 0.008065 |
| JCAD | 0.813012 | 0.008066 |
| ENDOU | -1.40656 | 0.008076 |
| COL6A2 | 0.799423 | 0.008093 |
| SFTPD | 1.10083 | 0.008134 |
| STARD9 | 0.690067 | 0.00817 |
| PRSS2 | -1.89174 | 0.008179 |
| ERFE | -2.02544 | 0.008193 |
| KCNJ13 | -1.75585 | 0.008227 |
| SLC1A1 | -0.83281 | 0.008238 |
| FOXD4L1 | -1.22889 | 0.008268 |
| ZNF219 | 0.640275 | 0.008272 |
| DCN | 0.901103 | 0.008317 |
| GCM1 | -1.60762 | 0.008329 |
| STAC2 | -1.95091 | 0.008344 |
| FSTL1 | 0.700749 | 0.008351 |
| SEC14L5 | -1.34852 | 0.008396 |
| SEMA3D | 1.239114 | 0.008431 |
| HOXD9 | -1.25716 | 0.008438 |
| LIPK | -2.53243 | 0.008471 |
| TUBB6 | 0.606619 | 0.008474 |
| S100A9 | -1.66474 | 0.008475 |
| RNPC3 | -0.62649 | 0.008475 |
| GUCA2A | 1.720113 | 0.008483 |
| C2orf78 | -4.97806 | 0.008484 |
| TRIM43 | -2.10777 | 0.008494 |
| KRBOX4 | 0.459425 | 0.008514 |
| LRFN5 | 1.215048 | 0.008582 |
| GNG2 | 0.631205 | 0.008584 |
| S100A8 | -1.60652 | 0.008596 |
| CDC37L1 | -0.48154 | 0.008604 |
| PAH | 1.663809 | 0.008637 |
| MAGEB1 | -4.37783 | 0.008658 |
| LRRC32 | 0.86762 | 0.008684 |
| DLG2 | 0.79326 | 0.008707 |
| IGFBP4 | 0.661714 | 0.008771 |
| CELA3B | -4.66407 | 0.008781 |
| IGSF5 | 1.010586 | 0.008787 |
| OLFM3 | -2.8707 | 0.008788 |
| RAP2B | -0.54872 | 0.008795 |
| GABRB3 | 1.398929 | 0.008798 |
| ISM1 | 1.003217 | 0.008805 |
| SLC7A14 | 1.000692 | 0.008824 |
| ZNF716 | 1.942575 | 0.008839 |
| OLFML3 | 0.753928 | 0.008863 |
| ANGPT2 | -0.69361 | 0.008872 |
| UBXN8 | 0.579192 | 0.00888 |
| ITGBL1 | 1.241281 | 0.008887 |
| CIDEC | 1.445628 | 0.008922 |
| H4C8 | -1.245 | 0.008944 |
| HTRA3 | 0.853783 | 0.008963 |
| NOL11 | -0.40568 | 0.008998 |
| PLIN1 | 1.320807 | 0.009066 |
| KLHL4 | 0.991334 | 0.009084 |
| SMIM31 | -3.62511 | 0.00914 |
| CPB1 | -2.2137 | 0.009149 |
| GJB6 | -1.44979 | 0.009166 |
| CA5A | 0.89026 | 0.009277 |
| MXRA5 | 0.801581 | 0.009282 |
| POM121L2 | -1.99052 | 0.009283 |
| PRR9 | 1.54535 | 0.00929 |
| AC138811.2 | -1.52304 | 0.009308 |
| FAM155A | 0.97099 | 0.00931 |
| BHLHB9 | 0.485472 | 0.009321 |
| ABCA13 | -1.35577 | 0.009339 |
| CD2AP | -0.46989 | 0.009347 |
| SERPINH1 | 0.450292 | 0.009358 |
| PGPEP1L | 1.352146 | 0.009366 |
| NR1I2 | 0.819656 | 0.009413 |
| LOX | 0.945712 | 0.009437 |
| SLC25A23 | -0.64143 | 0.009441 |
| PASD1 | 2.241664 | 0.009447 |
| SYN1 | 0.769183 | 0.009483 |
| BCL2L15 | -1.46213 | 0.009483 |
| PLPP7 | 0.804812 | 0.009503 |
| NRK | 1.085881 | 0.009507 |
| ALDH1A3 | 0.880216 | 0.009537 |
| SGCA | 1.007981 | 0.009545 |
| STC1 | -0.93831 | 0.00956 |
| GALNT8 | 1.345773 | 0.009664 |
| NAT8 | 1.002999 | 0.00967 |
| TSPAN18 | 0.800513 | 0.00968 |
| PER1 | 0.632135 | 0.009744 |
| FGF7 | 1.176455 | 0.009833 |
| DMRTC2 | 2.096239 | 0.00985 |
| C1QTNF9B | -1.73868 | 0.009865 |
| GAS1 | 0.795851 | 0.009889 |
| PHOSPHO1 | 1.120598 | 0.009916 |
| C11orf88 | 1.647617 | 0.009948 |
| DOK6 | 0.861874 | 0.00995 |
| SDHA | -0.53783 | 0.009967 |
| OR2AT4 | 1.780356 | 0.009991 |
| FGF12 | -1.12581 | 0.010043 |
| SLC22A18 | -0.79535 | 0.010063 |
| EPHA8 | 1.058019 | 0.010158 |
| KCNA1 | 1.409378 | 0.010165 |
| EPCAM | -0.52996 | 0.010171 |
| LEP | 1.072974 | 0.010172 |
| GPC3 | 1.305806 | 0.010212 |
| CD19 | 0.945501 | 0.010225 |
| RXFP3 | 1.196685 | 0.010225 |
| TMEM200A | 0.775257 | 0.010227 |
| NID1 | 0.616761 | 0.010231 |
| VWF | 0.584808 | 0.010272 |
| HKDC1 | -1.1544 | 0.010278 |
| PKD1L1 | -1.2158 | 0.010324 |
| GDA | -1.90506 | 0.010333 |
| POGLUT2 | 0.605119 | 0.010365 |
| SLAMF9 | -1.148 | 0.010414 |
| COPZ2 | 0.817666 | 0.010436 |
| NDOR1 | 0.488545 | 0.010518 |
| H1-3 | 1.010285 | 0.01059 |
| NEFL | 1.81138 | 0.010634 |
| COL6A3 | 0.841854 | 0.010656 |
| SBK3 | 1.184815 | 0.010744 |
| STARD10 | 0.642923 | 0.010776 |
| THEG | -4.09197 | 0.010792 |
| PLPPR3 | 1.260674 | 0.010812 |
| FADD | 0.445139 | 0.010836 |
| CERS1 | 0.94588 | 0.010849 |
| MS4A2 | 1.225573 | 0.010898 |
| COL25A1 | -1.8356 | 0.010932 |
| ZNF202 | 0.419258 | 0.011015 |
| ZNF705E | -1.3723 | 0.01106 |
| C8orf48 | 1.00714 | 0.011067 |
| CTSG | 1.323547 | 0.011112 |
| PLEKHA6 | -0.84742 | 0.011161 |
| IGFBP7 | 0.588133 | 0.011187 |
| FZD4 | 0.68761 | 0.011199 |
| CLPSL2 | -1.58219 | 0.011215 |
| TMEM209 | -0.43398 | 0.011231 |
| AQP1 | 0.602422 | 0.0113 |
| TMEM255A | 0.925258 | 0.011322 |
| TNFRSF21 | -0.66203 | 0.011322 |
| PRTG | 0.994061 | 0.011355 |
| BLK | 1.271671 | 0.011362 |
| CAMKV | -2.02324 | 0.011434 |
| CBLB | -0.49591 | 0.011448 |
| NNT | -0.48369 | 0.011468 |
| C11orf96 | 0.88377 | 0.011476 |
| TMPRSS11E | -2.59023 | 0.011513 |
| CHRD | 0.78151 | 0.011513 |
| FAM13C | 0.890162 | 0.011541 |
| MYL9 | 0.627196 | 0.011575 |
| MTCL1 | 1.020601 | 0.011586 |
| ITFG2 | 0.572492 | 0.011592 |
| LZTS1 | 0.768106 | 0.011597 |
| DTD2 | 0.496505 | 0.011629 |
| GLIPR1 | 0.637501 | 0.011649 |
| GPM6A | 1.158812 | 0.011709 |
| SPATA4 | 1.273006 | 0.011725 |
| RAB3D | -0.68555 | 0.011777 |
| ADAMTS12 | 0.89933 | 0.011818 |
| SPIRE1 | 0.667706 | 0.011878 |
| TFE3 | 0.435695 | 0.011884 |
| C1QTNF6 | 0.683192 | 0.011886 |
| SH3PXD2A | 0.613705 | 0.012035 |
| PPBP | 1.24146 | 0.012104 |
| PPM1L | -1.00116 | 0.012115 |
| POU4F1 | -1.6174 | 0.012146 |
| F13A1 | 0.830183 | 0.012174 |
| GIP | 1.212811 | 0.012194 |
| KCNE4 | 0.993637 | 0.012197 |
| MCHR2 | 1.706105 | 0.012203 |
| CD1C | 1.034462 | 0.012205 |
| SLC13A2 | -2.77161 | 0.012205 |
| TSPEAR | 1.283116 | 0.012221 |
| MATK | 0.848211 | 0.012229 |
| SLC22A18AS | -1.25174 | 0.012265 |
| PLPP1 | 0.484966 | 0.012404 |
| TERB2 | -4.18175 | 0.012406 |
| HMMR | -0.6842 | 0.012453 |
| PLXDC2 | 0.553188 | 0.012457 |
| CRNN | -2.90193 | 0.012472 |
| GRID1 | 1.044976 | 0.012493 |
| NR4A2 | 0.801153 | 0.01257 |
| CNTNAP4 | 1.746161 | 0.012582 |
| IGSF1 | -1.48345 | 0.012648 |
| LCE3E | 2.127037 | 0.01269 |
| AK3 | -0.50022 | 0.0127 |
| CYP4F22 | 1.013849 | 0.012716 |
| CLDN16 | 1.227703 | 0.012813 |
| NCAN | -2.15644 | 0.012841 |
| LEMD1 | -1.17782 | 0.01286 |
| PDGFRA | 0.886696 | 0.012895 |
| SLC3A1 | -1.98628 | 0.012938 |
| OR2B6 | -1.4294 | 0.012979 |
| BMX | 0.784106 | 0.012997 |
| CPA3 | 1.255542 | 0.013003 |
| RTL5 | 0.668392 | 0.013025 |
| FAM3D | -1.5617 | 0.013054 |
| LDLRAD4 | 0.626254 | 0.013094 |
| KERA | 1.361993 | 0.013128 |
| CA10 | -2.75845 | 0.013139 |
| KPNA2 | -0.56848 | 0.013167 |
| PSORS1C2 | 0.986539 | 0.01318 |
| TCP10L | 0.680206 | 0.013207 |
| COL2A1 | 1.595226 | 0.01323 |
| CYP4X1 | -1.11277 | 0.013238 |
| CALML3 | 1.565148 | 0.013359 |
| LRRN4CL | 0.828948 | 0.013387 |
| CREB3L1 | 0.798031 | 0.013399 |
| RMI1 | -0.54434 | 0.013419 |
| MMP28 | 0.867204 | 0.013436 |
| PEAR1 | 0.592494 | 0.013455 |
| SULT2A1 | -4.79717 | 0.013532 |
| H1-1 | 1.221064 | 0.013615 |
| DENND4C | -0.51378 | 0.013727 |
| RAMP3 | 0.577763 | 0.013743 |
| SHE | 0.663954 | 0.01384 |
| CLGN | -1.13775 | 0.013859 |
| OLFML2A | 0.790022 | 0.01402 |
| CDAN1 | 0.447773 | 0.014052 |
| SFRP2 | 1.388557 | 0.014092 |
| ZNF154 | 0.684039 | 0.014112 |
| CAMKK1 | 0.63306 | 0.014274 |
| KISS1R | -1.47515 | 0.014288 |
| PRND | 1.128146 | 0.01431 |
| AL136295.4 | 0.73729 | 0.014342 |
| STC2 | -0.81915 | 0.014416 |
| CCDC160 | -1.11038 | 0.014496 |
| OR52E2 | -5.35946 | 0.014523 |
| HOPX | 0.792022 | 0.014557 |
| TMEM238L | -2.58495 | 0.014561 |
| PDLIM3 | 0.879896 | 0.014602 |
| TUBA1A | 0.645281 | 0.014608 |
| SNTB1 | -0.7844 | 0.014611 |
| OTUD5 | 0.426097 | 0.014616 |
| REM2 | -0.6741 | 0.014633 |
| PABPC1L2A | -2.87477 | 0.014662 |
| ADAMTS20 | 1.426354 | 0.014721 |
| MIIP | 0.418661 | 0.014812 |
| SPINK8 | -2.57054 | 0.014832 |
| LYPLAL1 | 0.879441 | 0.014847 |
| CST5 | -1.96816 | 0.014857 |
| SLX4IP | 0.495955 | 0.014891 |
| PSKH1 | 0.460131 | 0.014948 |
| MYLK3 | 0.94562 | 0.015003 |
| CYP4F3 | -1.40762 | 0.015024 |
| MAP1A | 0.62597 | 0.015047 |
| KCNAB3 | 0.625895 | 0.015052 |
| OLFM4 | -4.11416 | 0.015063 |
| COLEC11 | 1.31495 | 0.01507 |
| MMP12 | -2.00071 | 0.015088 |
| HSPA6 | -1.02239 | 0.015091 |
| ANTXR1 | 0.711942 | 0.015092 |
| RP2 | 0.428697 | 0.015097 |
| ANGPTL5 | 1.434363 | 0.015097 |
| CHI3L2 | -1.81611 | 0.01511 |
| RHOXF2 | -2.86109 | 0.015184 |
| TCAF2 | -0.81343 | 0.015205 |
| CCDC141 | 0.995382 | 0.015248 |
| CALML5 | -2.45184 | 0.015267 |
| AC135178.3 | 0.829011 | 0.015269 |
| TKTL1 | 1.92715 | 0.015291 |
| DMRT1 | -2.05352 | 0.015309 |
| AC015688.4 | 0.914965 | 0.01536 |
| HEY1 | 0.915237 | 0.015363 |
| PDGFD | 0.698183 | 0.015397 |
| VASH1 | 0.540768 | 0.015399 |
| FCGR3B | 1.10116 | 0.015416 |
| SGK1 | 0.642508 | 0.015439 |
| GOLGA7B | 0.858321 | 0.015543 |
| ESM1 | -0.72038 | 0.015574 |
| ZNF772 | 0.572362 | 0.015584 |
| TGFB1I1 | 0.587479 | 0.015593 |
| PHACTR2 | 0.481591 | 0.015598 |
| REN | -2.1721 | 0.015606 |
| CCN4 | 0.879023 | 0.015648 |
| PNPO | -0.55718 | 0.015651 |
| GRID2IP | -1.32936 | 0.015658 |
| NACAD | 0.788951 | 0.015672 |
| TNN | 0.986428 | 0.015691 |
| CHSY1 | 0.423209 | 0.01571 |
| ZNF674 | 0.471134 | 0.01572 |
| TMEM205 | -0.64024 | 0.015722 |
| ABCC9 | 0.837644 | 0.01576 |
| NDN | 0.799546 | 0.015842 |
| NPY4R | 1.21098 | 0.015848 |
| AWAT1 | -2.45608 | 0.015854 |
| FER1L5 | 0.930591 | 0.015978 |
| CLINT1 | -0.46497 | 0.016012 |
| THPO | 0.85778 | 0.016086 |
| CAMSAP3 | -0.63502 | 0.016095 |
| CMTM5 | 1.564018 | 0.016173 |
| XG | -1.36109 | 0.01618 |
| AC109583.1 | -1.49486 | 0.016224 |
| TLE7 | -3.46769 | 0.016243 |
| DDC | -1.92376 | 0.016291 |
| PDPN | 0.790649 | 0.016379 |
| OLFML2B | 0.79978 | 0.016389 |
| GCLM | -0.51153 | 0.016472 |
| SMAD6 | 0.575627 | 0.016498 |
| CLDN18 | -2.83855 | 0.016566 |
| TDRD9 | -2.08063 | 0.016617 |
| ABCA9 | 0.893516 | 0.016642 |
| BMP3 | -1.84861 | 0.016665 |
| ACSL4 | -0.48359 | 0.016672 |
| C1orf141 | -2.4246 | 0.016727 |
| H2AC14 | 1.126602 | 0.016728 |
| AKAP1 | -0.40132 | 0.016734 |
| VCAN | 0.93596 | 0.016811 |
| HNRNPAB | -0.41709 | 0.016833 |
| TIMP2 | 0.584667 | 0.016879 |
| ANK3 | -0.67817 | 0.016889 |
| ADGRB2 | 0.706866 | 0.016915 |
| PGM2L1 | -0.73686 | 0.016917 |
| CFAP298 | 0.474174 | 0.016943 |
| TEK | 0.634637 | 0.01696 |
| UNC93A | -2.07996 | 0.016961 |
| AC011473.4 | -1.16116 | 0.016988 |
| TTBK1 | 0.824555 | 0.017093 |
| H2BC21 | -0.83036 | 0.017104 |
| MT-ND2 | -0.50898 | 0.017124 |
| CT83 | -4.85164 | 0.017175 |
| ART3 | 0.968913 | 0.017188 |
| TBX18 | -1.86869 | 0.017255 |
| LPO | -1.13742 | 0.0173 |
| PCDHGC3 | 0.745072 | 0.017456 |
| SCUBE1 | 1.038897 | 0.01749 |
| CCNB3 | 0.852535 | 0.01749 |
| FAM71E2 | 1.321702 | 0.017516 |
| CSPG4 | 0.656088 | 0.017607 |
| TBC1D3D | -3.18164 | 0.017609 |
| AXL | 0.573657 | 0.017634 |
| MYOCD | 1.22773 | 0.017641 |
| SLAIN1 | -0.72571 | 0.017643 |
| SMR3B | 1.464569 | 0.017654 |
| PLPP4 | 0.997521 | 0.017664 |
| H2BC14 | 1.243741 | 0.017677 |
| CA6 | -2.09908 | 0.017797 |
| FAM163A | 1.053592 | 0.017802 |
| UPK3A | -1.56712 | 0.017813 |
| SRD5A1 | -0.52507 | 0.017858 |
| GTF3A | -0.43784 | 0.017858 |
| KLHL13 | -1.34481 | 0.017862 |
| EPGN | 1.3721 | 0.017869 |
| DACH2 | -2.26773 | 0.017882 |
| FYN | 0.567878 | 0.017967 |
| ANK1 | -1.24044 | 0.017991 |
| PRR23C | -5.18936 | 0.018036 |
| PURG | 0.985922 | 0.018115 |
| PDZRN3 | 0.712785 | 0.018118 |
| HES4 | 0.819237 | 0.018137 |
| C7orf33 | 2.021382 | 0.018235 |
| KLRG2 | -1.22664 | 0.018241 |
| HSPB8 | 0.89581 | 0.01827 |
| DPPA4 | -2.4607 | 0.018279 |
| HOXB5 | -1.55983 | 0.018292 |
| SMAD7 | 0.457551 | 0.018405 |
| HSD3B1 | 1.733195 | 0.018443 |
| LHFPL6 | 0.604158 | 0.018525 |
| SLC1A7 | 0.853728 | 0.018559 |
| TCL1B | 1.754722 | 0.018577 |
| ALDH1A2 | 1.331627 | 0.018597 |
| CPLX1 | 0.656714 | 0.018604 |
| PRR5 | 0.476113 | 0.018615 |
| CTAGE1 | 0.881227 | 0.018674 |
| ATOH8 | 0.797124 | 0.018731 |
| PENK | -2.93602 | 0.018792 |
| MXRA8 | 0.783243 | 0.018798 |
| PARP4 | -0.50954 | 0.018806 |
| SLC6A3 | -1.46355 | 0.018867 |
| HEG1 | 0.710775 | 0.018881 |
| FAM47C | 1.353261 | 0.018893 |
| SPSB4 | 1.173669 | 0.018897 |
| SYNDIG1L | -1.21662 | 0.01891 |
| CYP4A11 | -2.01919 | 0.018929 |
| SIPA1L2 | 0.570585 | 0.018946 |
| FAM90A26 | 1.013001 | 0.018954 |
| GLT8D2 | 0.764845 | 0.018963 |
| PRDM14 | -2.93533 | 0.018993 |
| FERMT2 | 0.440736 | 0.01904 |
| TAS2R1 | 1.495102 | 0.019047 |
| KCNH5 | 1.68825 | 0.019089 |
| CPXM1 | 0.769254 | 0.019138 |
| APCS | -5.74628 | 0.01914 |
| MUCL3 | 1.257425 | 0.019244 |
| PRSS16 | -0.93764 | 0.019287 |
| CHL1 | -1.27707 | 0.019292 |
| ERV3-1 | 0.669019 | 0.019304 |
| CCNI2 | 0.722521 | 0.019363 |
| ONECUT3 | -2.99103 | 0.019378 |
| LTK | 0.98728 | 0.019582 |
| SYNJ2BP-COX16 | 0.58965 | 0.019592 |
| SYT2 | -1.48866 | 0.019632 |
| MAGEB2 | -4.49142 | 0.019702 |
| FOXI1 | -2.02627 | 0.019755 |
| AHSG | 1.171276 | 0.019785 |
| GGT1 | -1.01298 | 0.019836 |
| KIF21A | -0.52731 | 0.019904 |
| LRFN4 | 0.434196 | 0.019919 |
| FABP4 | 1.617303 | 0.019961 |
| CCDC63 | 1.308128 | 0.019997 |
| BAIAP2L1 | -0.46132 | 0.020004 |
| ATP5PB | -0.40462 | 0.020011 |
| MC4R | 1.26458 | 0.020032 |
| ZIC3 | -3.41467 | 0.020049 |
| TRO | 0.710776 | 0.020061 |
| FAM3C | -0.49062 | 0.020077 |
| EGR4 | 1.119303 | 0.020145 |
| SLC15A1 | -1.28158 | 0.020158 |
| ACRV1 | 0.708806 | 0.020166 |
| WDR83 | 0.528191 | 0.02022 |
| H1-2 | -1.09877 | 0.020508 |
| HOXA6 | 1.359789 | 0.02052 |
| EFCC1 | 0.840807 | 0.020627 |
| DNAJC2 | -0.40783 | 0.020651 |
| SPDYE4 | 1.181018 | 0.020653 |
| ANXA6 | 0.455199 | 0.020683 |
| GALNT5 | 0.897182 | 0.020702 |
| INSYN2A | 0.590949 | 0.020707 |
| OR2A7 | -1.08938 | 0.020761 |
| PIPOX | -1.11057 | 0.020803 |
| UCKL1 | 0.403558 | 0.020818 |
| CLDN11 | 0.787357 | 0.020836 |
| LSAMP | 0.857811 | 0.020861 |
| AC021066.1 | -0.75796 | 0.020916 |
| NPHS1 | 1.341832 | 0.020937 |
| HOXB6 | -1.46193 | 0.020967 |
| HAUS6 | -0.48371 | 0.020976 |
| HTRA1 | 0.673716 | 0.02098 |
| BPIFB1 | -1.92334 | 0.021004 |
| CXCL2 | 0.963042 | 0.021008 |
| HPSE2 | -2.85953 | 0.021012 |
| ZNF362 | 0.421938 | 0.021013 |
| NXNL1 | -2.06841 | 0.021065 |
| CNTNAP1 | 0.570018 | 0.021083 |
| PAMR1 | 0.778585 | 0.021189 |
| MTRNR2L7 | 1.13523 | 0.021234 |
| TENM1 | -1.76054 | 0.021275 |
| WTIP | 0.575249 | 0.021307 |
| FAT4 | 0.70578 | 0.021343 |
| CPA1 | -3.01564 | 0.021442 |
| ZNF732 | 0.723267 | 0.021499 |
| TANGO6 | 0.516593 | 0.021515 |
| KIRREL3 | 1.201497 | 0.021531 |
| FOXQ1 | 0.864126 | 0.021572 |
| FOXD2 | -1.34766 | 0.02159 |
| F11 | -3.09481 | 0.021615 |
| CLRN3 | -2.54465 | 0.02165 |
| C1QTNF7 | 0.825167 | 0.021658 |
| ITGA5 | 0.615812 | 0.02169 |
| PHLDB2 | 0.741841 | 0.021714 |
| AGMO | -1.6794 | 0.021734 |
| WBP2NL | -0.78587 | 0.021864 |
| GABRG1 | -2.92086 | 0.021912 |
| PSG5 | 1.330907 | 0.02194 |
| ABCA5 | -0.63833 | 0.021972 |
| SMIM23 | -1.7953 | 0.022006 |
| PLA2G1B | -2.137 | 0.022042 |
| FUT3 | -1.1011 | 0.022045 |
| NR5A2 | -1.35192 | 0.022066 |
| PF4 | -3.02868 | 0.022148 |
| TCTEX1D2 | -0.73522 | 0.022162 |
| GCNT3 | -1.16222 | 0.022209 |
| ADAMTSL4 | 0.637636 | 0.02221 |
| GPC5 | -1.85035 | 0.022348 |
| SH2D3A | -0.602 | 0.022358 |
| UBOX5 | 0.417052 | 0.02239 |
| HTR7 | 0.80847 | 0.02241 |
| ATP5PD | -0.568 | 0.022458 |
| ITIH1 | 0.936484 | 0.022521 |
| GRIN2C | 0.928521 | 0.022527 |
| TBC1D31 | -0.4766 | 0.022545 |
| TMEM212 | -2.88555 | 0.022707 |
| KRT2 | -2.70769 | 0.022719 |
| BARHL2 | 1.517817 | 0.022768 |
| IL6 | 0.988585 | 0.022787 |
| HOXA3 | 1.207892 | 0.022793 |
| ALDOA | -0.44826 | 0.022826 |
| PCDH11X | -2.54338 | 0.023231 |
| KLK1 | -1.57668 | 0.023239 |
| MAPKBP1 | 0.447934 | 0.023304 |
| MYO3A | -1.65616 | 0.023309 |
| TIE1 | 0.435635 | 0.023484 |
| S100A5 | -1.07166 | 0.023501 |
| LRRC3B | -1.63485 | 0.023583 |
| UGT2A3 | -4.60953 | 0.023654 |
| LRRN4 | 0.915313 | 0.023682 |
| OR2L2 | 1.340513 | 0.023699 |
| HAL | 0.976447 | 0.023724 |
| FILIP1L | 0.66275 | 0.023775 |
| DTHD1 | -2.02044 | 0.023781 |
| PIP5K1B | -1.14449 | 0.023788 |
| MYH13 | 1.226859 | 0.023844 |
| FAM53A | 0.602368 | 0.023857 |
| KIAA2026 | -0.47485 | 0.023956 |
| GRIK3 | -1.82526 | 0.024001 |
| GNB3 | 0.819836 | 0.024006 |
| C17orf58 | -0.48071 | 0.024065 |
| LTBP2 | 0.578231 | 0.024069 |
| ACTG2 | 0.911338 | 0.024082 |
| NCR2 | -1.89478 | 0.024183 |
| SI | -3.89305 | 0.024215 |
| BBOX1 | -1.09699 | 0.024245 |
| NAP1L3 | 0.746832 | 0.024316 |
| ZNF649 | 0.501422 | 0.024447 |
| TG | -1.06799 | 0.024459 |
| FOXN4 | 1.222322 | 0.024496 |
| PKD2L1 | 0.788264 | 0.024662 |
| QPCT | -1.14597 | 0.024665 |
| PCDHB6 | -1.30237 | 0.024682 |
| TMC1 | -1.3883 | 0.024712 |
| AQP7 | 0.953499 | 0.024773 |
| GXYLT2 | 0.627942 | 0.024862 |
| SLC37A2 | 0.672091 | 0.0249 |
| DGKI | 0.662424 | 0.024903 |
| AMTN | -2.97767 | 0.02493 |
| GPRC5C | -0.63091 | 0.024976 |
| SH3GL2 | -1.50301 | 0.025217 |
| MN1 | 0.83497 | 0.025283 |
| SSTR5 | -2.81803 | 0.025301 |
| NEXN | 0.697477 | 0.025313 |
| WDFY2 | -0.51242 | 0.025314 |
| CPN2 | 1.033013 | 0.025381 |
| TRAPPC5 | -0.57907 | 0.025422 |
| POSTN | 1.122772 | 0.025444 |
| XCL1 | 0.878923 | 0.025504 |
| CD1B | 1.188894 | 0.025855 |
| SPATA6L | -0.77341 | 0.025906 |
| HAND1 | -3.19235 | 0.025953 |
| GPX7 | 0.561708 | 0.025965 |
| SLC52A1 | 0.747903 | 0.025996 |
| MCU | -0.40543 | 0.026054 |
| LAMA2 | 0.700794 | 0.026141 |
| SIK1 | 1.066492 | 0.026212 |
| PIK3CD | 0.516461 | 0.026215 |
| FOXA1 | -2.41842 | 0.026217 |
| AMBP | -2.39101 | 0.026241 |
| BCCIP | -0.41734 | 0.026253 |
| NHSL2 | 0.65829 | 0.02627 |
| MFAP2 | 0.818704 | 0.026327 |
| GLB1L3 | -1.54238 | 0.026338 |
| WDR13 | 0.40661 | 0.026359 |
| SMIM32 | 1.333456 | 0.026394 |
| LCN10 | 1.013307 | 0.026423 |
| SLIT2 | 0.696505 | 0.026454 |
| EMCN | 0.550835 | 0.026483 |
| CTHRC1 | 0.772553 | 0.026498 |
| SRD5A2 | -1.79617 | 0.026532 |
| TMEM130 | 0.811529 | 0.02671 |
| LHFPL3 | -2.08951 | 0.026735 |
| RCAN2 | 0.603355 | 0.026736 |
| LDHA | -0.51253 | 0.026769 |
| LAMA3 | -1.06511 | 0.026843 |
| KCNF1 | -1.4753 | 0.026908 |
| RGPD6 | 0.70677 | 0.026986 |
| CALD1 | 0.5636 | 0.026994 |
| MYO6 | -0.42724 | 0.027025 |
| NTSR2 | 1.143854 | 0.027078 |
| PPM1N | 0.882483 | 0.027101 |
| OPTC | 1.086897 | 0.027125 |
| GPR1 | 0.922844 | 0.027228 |
| FCER1A | 0.978711 | 0.027228 |
| FAM228A | 0.891602 | 0.027317 |
| MFAP4 | 0.934246 | 0.02733 |
| FAM111B | -0.71803 | 0.027362 |
| LDLRAD1 | -1.33085 | 0.027422 |
| MAGEB10 | -2.39657 | 0.02748 |
| KLHL31 | -0.895 | 0.02758 |
| LTF | 1.293243 | 0.027665 |
| STAC | -1.15213 | 0.027686 |
| TMPRSS4 | -1.00433 | 0.027696 |
| CLEC1B | 1.100856 | 0.027777 |
| RET | -1.03247 | 0.027884 |
| VPREB3 | -0.93015 | 0.02796 |
| SCIN | -1.14808 | 0.028029 |
| VWDE | -1.04327 | 0.028082 |
| ERP27 | -1.1316 | 0.028205 |
| NUAK1 | 0.680242 | 0.028299 |
| RCN3 | 0.613584 | 0.028409 |
| H2BC4 | -0.85245 | 0.028451 |
| OR51E2 | 0.874259 | 0.028473 |
| LCT | -1.28796 | 0.0285 |
| CEACAM7 | 1.83453 | 0.028521 |
| DISP2 | 0.694274 | 0.028545 |
| RASSF6 | -1.01518 | 0.02863 |
| ZNF365 | 0.703693 | 0.028697 |
| CFAP410 | 0.418231 | 0.028764 |
| MYCL | -1.13009 | 0.028809 |
| PBOV1 | -1.36586 | 0.02883 |
| SKOR2 | -2.49945 | 0.028834 |
| PLEKHG7 | -1.41598 | 0.02884 |
| DAAM2 | 0.676196 | 0.028943 |
| UNC13A | -0.95465 | 0.028943 |
| GRHL3 | -0.85662 | 0.028972 |
| IFNL2 | -1.51298 | 0.028981 |
| FAM133A | 1.36752 | 0.029029 |
| LMCD1 | 0.483887 | 0.029074 |
| MARCHF3 | 0.615759 | 0.029222 |
| EMX1 | -1.64617 | 0.029231 |
| SLCO4C1 | -1.08953 | 0.029354 |
| JPT1 | -0.56861 | 0.029362 |
| BPIFB4 | -1.89359 | 0.029455 |
| RASGRF1 | -1.28901 | 0.0295 |
| TTPA | -1.272 | 0.029518 |
| SLC7A11 | -0.87587 | 0.029555 |
| ATP5IF1 | -0.5664 | 0.029759 |
| GALNTL5 | 1.345871 | 0.029843 |
| C1QTNF3 | 0.800014 | 0.029886 |
| TGFBI | 0.643215 | 0.029966 |
| PRRX1 | 0.873761 | 0.029974 |
| ANG | 0.669683 | 0.029976 |
| LONRF1 | 0.468695 | 0.030006 |
| AQP5 | -0.99471 | 0.03001 |
| OR3A2 | 1.784375 | 0.030046 |
| ARSB | 0.465107 | 0.030142 |
| ARHGAP28 | 0.687478 | 0.030148 |
| ZKSCAN2 | 0.480741 | 0.030236 |
| MISP | -0.66213 | 0.030435 |
| PCDHA9 | -1.26997 | 0.030527 |
| OR2T8 | 1.095204 | 0.030547 |
| AOC1 | -1.56841 | 0.030572 |
| C10orf53 | 1.18083 | 0.030583 |
| COL14A1 | 0.856517 | 0.030601 |
| UBE2H | -0.41471 | 0.03075 |
| C16orf92 | -1.71658 | 0.030904 |
| PHLDB1 | 0.472899 | 0.030938 |
| MYADM | 0.539484 | 0.030946 |
| PLAC9 | 0.620612 | 0.030961 |
| FGF3 | 1.789865 | 0.03101 |
| PPP1R1A | -1.4012 | 0.031113 |
| GUCA1C | -3.94626 | 0.031216 |
| ITGA11 | 0.861052 | 0.03124 |
| SCGB2A2 | 1.652414 | 0.031278 |
| FHL1 | 0.614366 | 0.031296 |
| ADAMTS10 | 0.680474 | 0.031329 |
| NDUFA6 | -0.5204 | 0.031449 |
| AOC2 | -0.92131 | 0.031499 |
| OLFML1 | 0.662575 | 0.031504 |
| SUSD5 | 0.636497 | 0.031526 |
| TRAF2 | 0.416489 | 0.03153 |
| TNFRSF18 | -1.32042 | 0.031731 |
| KCNAB1 | 0.522652 | 0.031748 |
| PON2 | -0.44449 | 0.031814 |
| CRB3 | -0.52279 | 0.031877 |
| RASD1 | 0.763804 | 0.031895 |
| S100A7A | -2.54679 | 0.031943 |
| APOBEC1 | -2.08041 | 0.031951 |
| PLS1 | -0.53487 | 0.032018 |
| NDP | -1.38864 | 0.032099 |
| NDNF | 0.912361 | 0.032112 |
| EPN3 | -0.71882 | 0.032131 |
| TMEM119 | 0.847349 | 0.032264 |
| ADGRG7 | -3.41663 | 0.032267 |
| RHOBTB2 | 0.444487 | 0.03229 |
| DPT | 1.031433 | 0.032435 |
| AREG | 1.067154 | 0.032437 |
| CACNA2D3 | -0.96327 | 0.032511 |
| SCG3 | 1.16269 | 0.032664 |
| HELT | 1.455233 | 0.032729 |
| TBXA2R | 0.531052 | 0.032769 |
| CDH16 | -2.47407 | 0.032776 |
| NUTM2E | 1.329648 | 0.032817 |
| PRKD1 | 0.550641 | 0.032837 |
| ETS1 | 0.430271 | 0.032891 |
| SIAE | 0.577674 | 0.032998 |
| CLYBL | -0.55235 | 0.033032 |
| AP4B1 | -0.42245 | 0.033164 |
| NRARP | 0.536864 | 0.033251 |
| ECT2 | -0.44257 | 0.033299 |
| ZEB2 | 0.534034 | 0.033397 |
| HTR3A | -1.37018 | 0.033483 |
| FGF1 | 0.791354 | 0.0335 |
| DEFB125 | -4.47705 | 0.033568 |
| MOXD1 | 0.82989 | 0.033643 |
| AVP | 1.128675 | 0.033724 |
| UPP1 | -0.80281 | 0.033822 |
| HOXC10 | -2.32848 | 0.033853 |
| PGLYRP2 | -1.04836 | 0.033891 |
| HEPH | 0.634396 | 0.033898 |
| TEX22 | -0.86684 | 0.0339 |
| IL16 | 0.59011 | 0.033934 |
| JPH2 | 0.777375 | 0.033986 |
| HOXA7 | 1.105103 | 0.034052 |
| PGK1 | -0.45412 | 0.034106 |
| LOXL3 | 0.460869 | 0.034111 |
| SERPINF1 | 0.754023 | 0.034121 |
| GDPD4 | -1.19414 | 0.034134 |
| SORBS3 | 0.461521 | 0.034136 |
| TNFAIP8L3 | 0.655578 | 0.034137 |
| BMP8B | -1.05881 | 0.034178 |
| REL | 0.426345 | 0.0342 |
| FXYD7 | 1.041191 | 0.034204 |
| ZSCAN23 | 0.786282 | 0.034299 |
| PPP1R15A | 0.475696 | 0.034319 |
| SUGCT | 0.687927 | 0.034331 |
| PTCHD1 | -1.38702 | 0.034354 |
| CDCP1 | -0.56148 | 0.034494 |
| FAP | 0.958375 | 0.034553 |
| APBA2 | 0.619943 | 0.034597 |
| BLVRB | -0.57488 | 0.034656 |
| PALLD | 0.556336 | 0.034705 |
| CH25H | 0.786325 | 0.034818 |
| S100A14 | -0.79368 | 0.034901 |
| ETFRF1 | -0.61655 | 0.034935 |
| DYNC2H1 | 0.529797 | 0.034992 |
| AC134669.1 | 0.879026 | 0.035007 |
| ANXA2R | -0.77446 | 0.035032 |
| CPLX4 | -2.02368 | 0.035081 |
| APOD | 0.649024 | 0.035103 |
| RFPL3 | 0.859499 | 0.03516 |
| CYP4Z1 | -1.29894 | 0.035193 |
| HMGN5 | -0.68199 | 0.035212 |
| MYO18B | 1.331196 | 0.035262 |
| ANKS4B | -3.11386 | 0.035347 |
| CTXND1 | -1.58436 | 0.035352 |
| VWA3B | -1.2797 | 0.035373 |
| GNG11 | 0.479397 | 0.035428 |
| PTPRU | 0.533227 | 0.035434 |
| CMAS | -0.40818 | 0.035454 |
| MIA | -1.13175 | 0.035519 |
| AC005747.1 | 0.558602 | 0.035542 |
| H2BC13 | 0.831285 | 0.035561 |
| CXCL17 | -1.5311 | 0.035585 |
| CRB1 | -1.68902 | 0.035611 |
| SLC41A2 | -0.67179 | 0.035944 |
| CYP4F11 | -1.02072 | 0.035994 |
| SEMA6B | 0.445264 | 0.036086 |
| GPX8 | 0.416121 | 0.036122 |
| KRTAP5-8 | 0.840466 | 0.036146 |
| MYO16 | 0.90223 | 0.036225 |
| CD300LG | 1.025531 | 0.036258 |
| SLC39A5 | -1.63518 | 0.036388 |
| EID3 | 0.539462 | 0.036455 |
| TMPRSS11D | -1.35682 | 0.036456 |
| PIK3R3 | -0.63734 | 0.036477 |
| XIRP2 | 1.283764 | 0.03649 |
| SIX4 | -0.48414 | 0.036509 |
| KLF10 | 0.491158 | 0.036558 |
| RDH5 | -0.84925 | 0.03656 |
| TNFSF9 | -1.21789 | 0.036599 |
| TRIQK | 0.413238 | 0.036675 |
| MT-CO3 | -0.44572 | 0.036762 |
| PCDHGA7 | 0.838814 | 0.036768 |
| EDNRB | -0.9499 | 0.036785 |
| NR2E1 | -1.84345 | 0.03682 |
| PLAAT2 | -1.04209 | 0.03687 |
| HOXA4 | 1.24381 | 0.036886 |
| ZNF578 | 0.680164 | 0.036932 |
| TNKS | 0.409732 | 0.036936 |
| NAV1 | 0.60486 | 0.036947 |
| SH3RF3 | 0.607925 | 0.03701 |
| CAMK2A | 0.902814 | 0.037053 |
| CD177 | 1.052008 | 0.037062 |
| ASB10 | -1.65514 | 0.037064 |
| TRPM5 | 0.92344 | 0.037107 |
| FAM71F2 | -0.73531 | 0.037159 |
| GPKOW | 0.452181 | 0.037265 |
| MUC13 | -1.82991 | 0.037274 |
| SLC43A1 | 0.612243 | 0.037309 |
| ATP6V0D2 | 0.918042 | 0.037348 |
| GRIA2 | -1.43322 | 0.037495 |
| RGN | 0.725871 | 0.037538 |
| DENND2D | -0.4077 | 0.03762 |
| SEPTIN4 | 0.438721 | 0.037642 |
| OR11H4 | 1.378858 | 0.037678 |
| MUC3A | -1.28678 | 0.037713 |
| PRDM8 | 0.619898 | 0.037791 |
| VSTM4 | 0.722568 | 0.037802 |
| GRAMD2B | -0.59535 | 0.037806 |
| ELP4 | -0.40444 | 0.037869 |
| HMGCS2 | -2.02634 | 0.037933 |
| HAND2 | 0.839734 | 0.037949 |
| TCP10 | 0.978892 | 0.038035 |
| BPGM | -0.41931 | 0.038051 |
| CTNNAL1 | -0.51029 | 0.038307 |
| H4C5 | 0.756183 | 0.038339 |
| ATP1A2 | 1.166324 | 0.038377 |
| FGFBP2 | 0.73813 | 0.038414 |
| CDKN1C | 0.629073 | 0.038428 |
| OR1N1 | 1.758077 | 0.038442 |
| LYNX1 | 0.907846 | 0.038452 |
| SNTN | -2.0446 | 0.038455 |
| HOXA9 | 1.312118 | 0.038459 |
| NPY5R | -3.04017 | 0.038467 |
| BHLHE41 | -0.70442 | 0.03849 |
| MRPS26 | 0.40988 | 0.038493 |
| BTNL9 | -0.96158 | 0.038493 |
| PNMA8B | 0.552136 | 0.0385 |
| MSH4 | -1.11893 | 0.038523 |
| AP4S1 | 0.444957 | 0.038638 |
| TAS2R38 | -1.95534 | 0.038662 |
| NKD1 | 0.669689 | 0.038671 |
| UHRF2 | -0.4158 | 0.038708 |
| COL6A1 | 0.648287 | 0.038802 |
| SPTB | -0.97381 | 0.038849 |
| COL13A1 | -1.05099 | 0.038885 |
| PITX2 | -1.86829 | 0.038929 |
| ACKR1 | 0.831136 | 0.038988 |
| ARSF | 0.921918 | 0.039021 |
| DMAC1 | -0.52782 | 0.039035 |
| S100A11 | -0.58081 | 0.03904 |
| KLHL34 | -1.98716 | 0.039155 |
| PCDHGB3 | 0.895353 | 0.039299 |
| GDF6 | 0.867222 | 0.039333 |
| NTRK2 | 0.739729 | 0.039367 |
| IL1R2 | -1.01687 | 0.039381 |
| ATP6V0A4 | -1.13448 | 0.039423 |
| SMC4 | -0.46823 | 0.039536 |
| CPA5 | -1.10959 | 0.039552 |
| IL9R | 0.775042 | 0.039589 |
| COLEC10 | -0.99848 | 0.039597 |
| PLXDC1 | 0.54921 | 0.039616 |
| CD24 | -0.72159 | 0.039682 |
| SDK1 | 0.744224 | 0.039683 |
| HOXC11 | -2.78742 | 0.039709 |
| CLDN5 | 0.538604 | 0.039805 |
| CD36 | 0.81794 | 0.039808 |
| WDR91 | -0.47004 | 0.039888 |
| HRH3 | 1.196875 | 0.039888 |
| OR1J4 | -1.35025 | 0.039895 |
| C16orf54 | 0.775151 | 0.039901 |
| HTR3C | -2.91756 | 0.039918 |
| VN1R2 | 1.00679 | 0.039924 |
| UMODL1 | 0.991497 | 0.039958 |
| SSBP1 | -0.41811 | 0.04 |
| USHBP1 | 0.631136 | 0.040006 |
| NOL3 | -0.48264 | 0.040015 |
| NXNL2 | -0.95059 | 0.040076 |
| PGF | 0.552345 | 0.040142 |
| SEMA3C | 0.535445 | 0.040151 |
| SNAI2 | 0.703996 | 0.040265 |
| ADPRHL1 | -0.63951 | 0.040291 |
| SOST | 1.026694 | 0.040298 |
| DDIT4 | -0.72463 | 0.0403 |
| COX7A1 | 0.475341 | 0.04036 |
| PCDHB7 | -1.1002 | 0.040364 |
| FBP1 | -0.96313 | 0.040371 |
| PIK3C2G | -2.00624 | 0.040409 |
| WFIKKN2 | 1.176588 | 0.04043 |
| MUC7 | -2.9876 | 0.040462 |
| REEP6 | -0.83197 | 0.040494 |
| TNP1 | -3.4087 | 0.040534 |
| FSTL4 | 0.995601 | 0.040776 |
| DAB2 | 0.452513 | 0.040855 |
| RAPGEF3 | -0.70036 | 0.040879 |
| LYVE1 | 0.708062 | 0.040898 |
| AMER3 | 1.180009 | 0.040986 |
| TMEM88 | 0.73617 | 0.041174 |
| GEMIN8 | -0.51144 | 0.04121 |
| SLC39A8 | -0.80021 | 0.041225 |
| ATAD2 | -0.44033 | 0.041274 |
| RFX4 | 1.581052 | 0.041299 |
| DLGAP2 | 0.905855 | 0.041355 |
| MEST | 0.743021 | 0.041395 |
| ADAM23 | 0.845239 | 0.041417 |
| GRIN2B | 1.065273 | 0.041456 |
| ALX1 | 1.315943 | 0.041522 |
| OR5B12 | -2.16055 | 0.041542 |
| EFS | 0.73846 | 0.041551 |
| KCNQ5 | -1.44002 | 0.041566 |
| OR56A5 | 1.181046 | 0.041584 |
| KRT19 | -0.5395 | 0.041656 |
| MIOX | -0.90176 | 0.041666 |
| EIF3G | -0.46133 | 0.041699 |
| P2RX5 | 0.739259 | 0.04173 |
| NTN3 | 0.838931 | 0.041863 |
| MFNG | 0.484567 | 0.041877 |
| NPM1 | -0.42272 | 0.04226 |
| SREBF2 | -0.47141 | 0.042274 |
| EHD2 | 0.556294 | 0.042332 |
| ARID3A | -0.73029 | 0.042347 |
| CAV1 | 0.471427 | 0.042388 |
| CDKN2A | -0.91564 | 0.042472 |
| KCNMB1 | 0.589592 | 0.042511 |
| SLC10A6 | 0.822126 | 0.042561 |
| TNXB | 0.78879 | 0.042582 |
| CILP | 0.852381 | 0.042679 |
| CCZ1 | -0.4075 | 0.042862 |
| TFRC | -0.57365 | 0.042978 |
| LURAP1L | -0.72742 | 0.043021 |
| RASGRF2 | 0.597971 | 0.043043 |
| ARHGEF15 | 0.444232 | 0.043145 |
| THRSP | -1.24797 | 0.043171 |
| KNCN | 1.262575 | 0.043222 |
| FSD2 | 0.65806 | 0.043307 |
| RCL1 | -0.40047 | 0.043328 |
| C17orf64 | -1.58252 | 0.043385 |
| LBHD1 | 0.440329 | 0.043495 |
| TP53TG3D | 0.894169 | 0.043515 |
| RHPN2 | -0.48739 | 0.043587 |
| CLIC5 | -0.75041 | 0.043587 |
| COX7B | -0.58504 | 0.043675 |
| TPD52 | -0.40347 | 0.0437 |
| CACNA1F | 0.699957 | 0.043809 |
| CYCS | -0.42832 | 0.043824 |
| DKK4 | -1.65525 | 0.043988 |
| BCHE | 1.078908 | 0.044106 |
| LRRC37A3 | -0.66275 | 0.044169 |
| MUC16 | -0.98752 | 0.044298 |
| PSD3 | 0.517461 | 0.044374 |
| CRCT1 | 1.574683 | 0.044425 |
| TEPP | 0.77652 | 0.044431 |
| LRRC23 | 0.56673 | 0.044499 |
| GYPE | 0.822418 | 0.044568 |
| LRRC8E | -0.66606 | 0.044587 |
| SLC40A1 | -0.78399 | 0.044727 |
| GDF7 | 0.659315 | 0.044761 |
| CTSF | 0.460654 | 0.044984 |
| GRM5 | -2.02719 | 0.045022 |
| SLC4A3 | 0.533138 | 0.045059 |
| CCN5 | 0.724734 | 0.045172 |
| EXTL1 | 0.676452 | 0.045188 |
| FAM71F1 | -1.23072 | 0.045225 |
| CA7 | -1.62262 | 0.045228 |
| GABRB1 | -2.30493 | 0.045258 |
| FN1 | 0.795423 | 0.045269 |
| LACTB2 | -0.52444 | 0.045269 |
| MS4A8 | -2.11373 | 0.045279 |
| SHISA7 | 0.759017 | 0.045365 |
| PRSS33 | -1.82986 | 0.045368 |
| KRT78 | -0.96975 | 0.045507 |
| FGF8 | -1.64163 | 0.04556 |
| CCR4 | 0.771024 | 0.045675 |
| C11orf16 | 1.124496 | 0.045694 |
| CCDC190 | -1.79316 | 0.04577 |
| CATSPERD | -1.77769 | 0.045915 |
| PHLDA1 | 0.59763 | 0.045964 |
| CCR8 | -1.35606 | 0.045986 |
| FRMD6 | 0.542569 | 0.046032 |
| ELFN1 | 0.569717 | 0.04607 |
| ORM1 | -2.06631 | 0.046102 |
| AC068580.4 | -0.85717 | 0.04612 |
| ROR2 | 0.664647 | 0.046148 |
| TRIM73 | -0.80576 | 0.046318 |
| SYCE1 | 1.340726 | 0.046329 |
| RASIP1 | 0.63302 | 0.046364 |
| PRODH | -1.45576 | 0.046377 |
| PDE7B | 0.517576 | 0.046519 |
| THBS3 | 0.445146 | 0.046637 |
| HNF4G | -1.58379 | 0.046672 |
| DOCK11 | 0.572607 | 0.046715 |
| RPS6KA6 | -0.93322 | 0.046728 |
| PCYT1B | 0.803752 | 0.046847 |
| ARRB1 | 0.440449 | 0.046862 |
| GLT1D1 | 0.963956 | 0.046903 |
| SYTL3 | -0.56798 | 0.04696 |
| KRT72 | -1.61766 | 0.046977 |
| ARHGAP8 | -0.59239 | 0.04699 |
| LIN28B | 1.408493 | 0.047007 |
| ADAMTSL3 | 0.685237 | 0.04706 |
| CRYM | -1.19217 | 0.047062 |
| CCL7 | -1.37769 | 0.047141 |
| PLXNC1 | 0.418807 | 0.047172 |
| TMCO5A | -1.899 | 0.047229 |
| ENAM | -1.4789 | 0.047237 |
| PDZD3 | 0.742253 | 0.047246 |
| ZFP62 | -0.40306 | 0.047368 |
| PROM2 | -0.86625 | 0.047395 |
| ERFL | 0.713477 | 0.047431 |
| PRSS8 | -0.47261 | 0.047441 |
| PTGFR | 1.07108 | 0.047479 |
| GRIN3B | -1.01442 | 0.047534 |
| CYGB | 0.503232 | 0.047605 |
| PLB1 | -0.83266 | 0.047695 |
| F2R | 0.49612 | 0.047743 |
| TTC9 | -0.71302 | 0.047774 |
| PLPPR4 | 0.747777 | 0.047866 |
| CYP27B1 | -0.82408 | 0.047947 |
| ADH1B | 1.239358 | 0.048148 |
| KIFC3 | 0.478389 | 0.04815 |
| GPRASP1 | 0.560333 | 0.048306 |
| FNBP1 | -0.41096 | 0.048338 |
| GRM8 | -1.14327 | 0.048352 |
| DIRAS2 | -0.86621 | 0.048397 |
| CD55 | -0.56462 | 0.048416 |
| CITED1 | -1.3916 | 0.048422 |
| MYOZ3 | 0.717988 | 0.048428 |
| DPPA5 | -3.23159 | 0.048455 |
| EFNB2 | 0.517759 | 0.048587 |
| PDE6B | 0.484688 | 0.048663 |
| PADI3 | 1.361434 | 0.048675 |
| TBPL2 | -1.20902 | 0.048711 |
| GJA8 | 1.139644 | 0.048738 |
| CCL11 | 1.027153 | 0.048782 |
| NATD1 | 0.405934 | 0.048804 |
| HMGCLL1 | -1.47691 | 0.048812 |
| A4GNT | 0.920103 | 0.048892 |
| H2BC11 | -0.9212 | 0.04891 |
| RFC3 | -0.41774 | 0.048978 |
| TENT5C | -0.86697 | 0.04903 |
| KLKB1 | -0.998 | 0.049069 |
| PRELP | 0.712036 | 0.049112 |
| NPEPL1 | 0.419804 | 0.049261 |
| TSPAN11 | 0.619748 | 0.049271 |
| HMGCS1 | -0.46104 | 0.049279 |
| GDF10 | -1.81026 | 0.049319 |
| NXF5 | -1.61056 | 0.049327 |
| PIGX | -0.40335 | 0.049354 |
| HHIPL1 | 0.598936 | 0.04937 |
| ZBED6 | 0.579141 | 0.049391 |
| WFDC1 | 0.681877 | 0.04945 |
| KCNK17 | 0.604147 | 0.049543 |
| CSF3 | -1.91154 | 0.049577 |
| ARSD | -0.45104 | 0.049653 |
| ANGPT4 | 0.694257 | 0.049665 |
| HSPA4L | -0.76568 | 0.049747 |
| CLEC2L | 1.278914 | 0.049811 |
| CWH43 | -1.76166 | 0.049926 |

**Table S9.** COL1A1 targets were predicted by TargetScan.

| **Name** | **Position in the UTR** | **seed match** | **context++ score** | **context++ score percentile** | **weighted context++ score** | **conserved branch length** | **Pct** |
| --- | --- | --- | --- | --- | --- | --- | --- |
| miR-98 | 789-795 | 7mer-m8 | -0.27 | 86 | -0.27 | 4.756 | 0.89 |
| miR-143 | 152-158 | 7mer-m8 | -0.24 | 95 | -0.24 | 6.078 | 0.24 |

**Table S10.** COL1A1 targets were predicted by miRSystem.

| **MIRNA** | **TOTAL_HIT** | **VALIDATION** | **DIANA** | **MIRANDA** | **MIRBRIDGE** | **PICTAR** | **PITA** | **RNA22** | **TARGETSCAN** |
| --- | --- | --- | --- | --- | --- | --- | --- | --- | --- |
| miR-98 | 6 |  | √ |  | √ | √ | √ | √ | √ |
| miR-143 | 4 |  | √ | √ |  |  | √ |  | √ |

**Table S11**. The coexpression genes of COL1A1 among the Carboplatin resistant related genes by WGCNA.

| Gene symbol |
| --- |
| COL6A3 |
| MMP2 |
| CRISPLD2 |
| SPARC |
| ADAMTS2 |
| COL5A2 |
| THBS2 |
| COL3A1 |
| COL1A2 |
| COL5A1 |
| COL1A1 |
| CDH11 |
| COL8A1 |
| VCAN |

**Table S12**. The results of Gene Set Analysis (GSEA) in Carboplatin resistant groups.

| GS follow link to MSigDB | SIZE | ES | NES | NOM p-val | FDR q-val | FWER p-val | RANK AT MAX | LEADING EDGE |
| --- | --- | --- | --- | --- | --- | --- | --- | --- |
| KEGG_ECM_RECEPTOR_INTERACTION | 84 | 0.51 | 2.07 | 0 | 0.002 | 0.003 | 3710 | tags=46%, list=19%, signal=57% |
| KEGG_GLYCOSAMINOGLYCAN_BIOSYNTHESIS_CHONDROITIN_SULFATE | 22 | 0.6 | 1.84 | 0 | 0.026 | 0.07 | 5912 | tags=68%, list=30%, signal=98% |
| KEGG_FOCAL_ADHESION | 197 | 0.39 | 1.79 | 0 | 0.035 | 0.136 | 2963 | tags=31%, list=15%, signal=36% |
| KEGG_TGF_BETA_SIGNALING_PATHWAY | 85 | 0.43 | 1.74 | 0.001 | 0.05 | 0.252 | 3991 | tags=39%, list=20%, signal=49% |
| KEGG_NOTCH_SIGNALING_PATHWAY | 47 | 0.44 | 1.6 | 0.006 | 0.164 | 0.667 | 4608 | tags=36%, list=24%, signal=47% |

**Table S13**. The results of Gene Set Analysis (GSEA) in Carboplatin nonresistant groups.

| GS follow link to MSigDB | SIZE | ES | NES | NOM p-val | FDR q-val | FWER p-val | RANK AT MAX | LEADING EDGE |
| --- | --- | --- | --- | --- | --- | --- | --- | --- |
| KEGG_OXIDATIVE_PHOSPHORYLATION | 129 | -0.52 | -2.52 | 0 | 0 | 0 | 5154 | tags=56%, list=26%, signal=75% |
| KEGG_PARKINSONS_DISEASE | 125 | -0.5 | -2.39 | 0 | 0 | 0 | 3755 | tags=40%, list=19%, signal=49% |
| KEGG_PROTEASOME | 44 | -0.54 | -2.16 | 0 | 0.002 | 0.003 | 4412 | tags=45%, list=23%, signal=59% |
| KEGG_HUNTINGTONS_DISEASE | 177 | -0.39 | -1.99 | 0 | 0.006 | 0.016 | 3906 | tags=33%, list=20%, signal=41% |
| KEGG_ANTIGEN_PROCESSING_AND_PRESENTATION | 80 | -0.44 | -1.98 | 0 | 0.006 | 0.018 | 4724 | tags=38%, list=24%, signal=49% |
| KEGG_TERPENOID_BACKBONE_BIOSYNTHESIS | 15 | -0.65 | -1.94 | 0.008 | 0.009 | 0.032 | 5459 | tags=80%, list=28%, signal=111% |
| KEGG_ALZHEIMERS_DISEASE | 163 | -0.36 | -1.83 | 0 | 0.021 | 0.089 | 3382 | tags=29%, list=17%, signal=35% |
| KEGG_CITRATE_CYCLE_TCA_CYCLE | 29 | -0.5 | -1.78 | 0.003 | 0.031 | 0.144 | 3972 | tags=41%, list=20%, signal=52% |
| KEGG_GLUTATHIONE_METABOLISM | 47 | -0.43 | -1.71 | 0.006 | 0.048 | 0.242 | 3234 | tags=34%, list=17%, signal=41% |
| KEGG_VALINE_LEUCINE_AND_ISOLEUCINE_DEGRADATION | 44 | -0.43 | -1.68 | 0.006 | 0.058 | 0.312 | 3038 | tags=36%, list=16%, signal=43% |
| KEGG_PENTOSE_PHOSPHATE_PATHWAY | 26 | -0.47 | -1.66 | 0.012 | 0.065 | 0.368 | 4202 | tags=46%, list=21%, signal=59% |
| KEGG_GLYCOLYSIS_GLUCONEOGENESIS | 61 | -0.4 | -1.65 | 0 | 0.062 | 0.381 | 2209 | tags=26%, list=11%, signal=29% |
| KEGG_GLYOXYLATE_AND_DICARBOXYLATE_METABOLISM | 15 | -0.55 | -1.61 | 0.023 | 0.079 | 0.482 | 1327 | tags=33%, list=7%, signal=36% |
| KEGG_PEROXISOME | 78 | -0.36 | -1.6 | 0 | 0.078 | 0.501 | 6593 | tags=55%, list=34%, signal=83% |
| KEGG_FRUCTOSE_AND_MANNOSE_METABOLISM | 34 | -0.41 | -1.56 | 0.025 | 0.099 | 0.617 | 1966 | tags=29%, list=10%, signal=33% |
| KEGG_DNA_REPLICATION | 36 | -0.4 | -1.51 | 0.019 | 0.133 | 0.749 | 3002 | tags=28%, list=15%, signal=33% |
| KEGG_MISMATCH_REPAIR | 23 | -0.44 | -1.49 | 0.047 | 0.14 | 0.79 | 3949 | tags=35%, list=20%, signal=44% |
| KEGG_STARCH_AND_SUCROSE_METABOLISM | 51 | -0.36 | -1.47 | 0.027 | 0.149 | 0.826 | 1369 | tags=25%, list=7%, signal=27% |
| KEGG_BUTANOATE_METABOLISM | 34 | -0.39 | -1.45 | 0.036 | 0.165 | 0.874 | 2876 | tags=29%, list=15%, signal=34% |
| KEGG_AUTOIMMUNE_THYROID_DISEASE | 50 | -0.34 | -1.4 | 0.042 | 0.198 | 0.938 | 6342 | tags=42%, list=32%, signal=62% |
| KEGG_CARDIAC_MUSCLE_CONTRACTION | 77 | -0.32 | -1.39 | 0.034 | 0.201 | 0.947 | 4751 | tags=42%, list=24%, signal=55% |
| KEGG_SPLICEOSOME | 126 | -0.28 | -1.36 | 0.027 | 0.219 | 0.97 | 4776 | tags=34%, list=24%, signal=45% |

**Table S14**. The results of virtual screen by Schrodinger Maestro

| Title | Entry Name | docking score | glide score |
| --- | --- | --- | --- |
| ZINC000085537017 | fda.2090 | -8.19 | -8.204 |
| ZINC000085537017 | fda.2091 | -8.19 | -8.204 |
| ZINC000085537017 | fda.2092 | -8.19 | -8.204 |
| ZINC000003802690 | fda.227 | -7.638 | -7.638 |
| ZINC000000018635 | fda.26 | -7.626 | -7.626 |
| ZINC000003810860 | fda.1710 | -7.497 | -7.498 |
| ZINC000011677837 | fda.483 | -7.487 | -7.487 |
| ZINC000013585233 | fda.272 | -7.426 | -7.445 |
| ZINC000013585233 | fda.274 | -7.426 | -7.445 |
| ZINC000001530922 | fda.1120 | -7.358 | -7.405 |
| ZINC000000002279 | fda.477 | -7.313 | -7.313 |
| ZINC000000011012 | fda.1721 | -7.313 | -7.313 |
| ZINC000001530922 | fda.1119 | -7.305 | -7.352 |
| ZINC000001530922 | fda.1121 | -7.305 | -7.352 |
| ZINC000013585233 | fda.273 | -7.294 | -7.314 |
| ZINC000001530930 | fda.1953 | -7.189 | -7.247 |
| ZINC000003876069 | fda.1979 | -7.189 | -7.247 |
| ZINC000003775644 | fda.969 | -7.096 | -7.096 |
| ZINC000002539827 | fda.1383 | -7.088 | -7.108 |
| ZINC000002539827 | fda.1385 | -7.088 | -7.108 |
| ZINC000000895154 | fda.1406 | -7.088 | -7.108 |
| ZINC000000895154 | fda.1408 | -7.088 | -7.108 |
| ZINC000000057624 | fda.630 | -7.029 | -7.046 |
| ZINC000002015035 | fda.1967 | -7.014 | -7.014 |
| ZINC000002015035 | fda.1968 | -7.014 | -7.014 |
| ZINC000000039089 | fda.1157 | -7 | -7.011 |
| ZINC000003874498 | fda.358 | -6.988 | -6.988 |
| ZINC000003918453 | fda.1456 | -6.959 | -7.008 |
| ZINC000013986658 | fda.541 | -6.945 | -6.966 |
| ZINC000002539827 | fda.1384 | -6.905 | -6.925 |
| ZINC000000895154 | fda.1407 | -6.905 | -6.925 |
| ZINC000018279854 | fda.747 | -6.867 | -6.873 |
| ZINC000049783788 | fda.2086 | -6.856 | -6.856 |
| ZINC000026985532 | fda.355 | -6.828 | -6.841 |
| ZINC000000000740 | fda.1413 | -6.777 | -6.777 |
| ZINC000000896634 | fda.1079 | -6.777 | -6.777 |
| ZINC000049783788 | fda.2084 | -6.764 | -6.765 |
| ZINC000001543475 | fda.1852 | -6.761 | -7.342 |
| ZINC000001543475 | fda.1853 | -6.761 | -7.342 |
| ZINC000001543475 | fda.1854 | -6.761 | -7.342 |
| ZINC000001530930 | fda.1952 | -6.755 | -6.812 |
| ZINC000003876069 | fda.1978 | -6.755 | -6.812 |
| ZINC000001481815 | fda.792 | -6.754 | -6.759 |
| ZINC000049783788 | fda.2085 | -6.743 | -6.743 |
| ZINC000003978005 | fda.1235 | -6.731 | -6.781 |
| ZINC000003978005 | fda.1236 | -6.731 | -6.781 |
| ZINC000003978005 | fda.1237 | -6.731 | -6.781 |
| ZINC000028973441 | fda.1995 | -6.685 | -6.685 |
| ZINC000028973446 | fda.1996 | -6.685 | -6.685 |
| ZINC000028232750 | fda.175 | -6.665 | -6.665 |
| ZINC000000002688 | fda.1945 | -6.656 | -7.314 |
| ZINC000085537017 | fda.2092 | -6.652 | -6.666 |
| ZINC000084589076 | fda.1911 | -6.637 | -6.658 |
| ZINC000100006264 | fda.1917 | -6.637 | -6.658 |
| ZINC000001482049 | fda.165 | -6.634 | -6.634 |
| ZINC000008101127 | fda.87 | -6.603 | -6.603 |
| ZINC000004228258 | fda.1062 | -6.603 | -6.622 |
| ZINC000004228257 | fda.258 | -6.603 | -6.622 |
| ZINC000001540998 | fda.1956 | -6.58 | -6.58 |
| ZINC000001482049 | fda.164 | -6.561 | -6.561 |
| ZINC000013648755 | fda.418 | -6.558 | -6.67 |
| ZINC000013648755 | fda.417 | -6.554 | -6.666 |
| ZINC000002015928 | fda.672 | -6.532 | -6.538 |
| ZINC000000002281 | fda.1029 | -6.527 | -6.542 |
| ZINC000000000746 | fda.149 | -6.527 | -6.542 |
| ZINC000001530929 | fda.1950 | -6.509 | -6.566 |
| ZINC000003876068 | fda.1976 | -6.509 | -6.566 |
| ZINC000003823492 | fda.1642 | -6.492 | -6.492 |
| ZINC000002036848 | fda.279 | -6.457 | -6.457 |
| ZINC000002036848 | fda.280 | -6.457 | -6.457 |
| ZINC000052955754 | fda.129 | -6.45 | -6.627 |
| ZINC000052955754 | fda.130 | -6.45 | -6.627 |
| ZINC000052955754 | fda.131 | -6.45 | -6.627 |
| ZINC000001530929 | fda.1951 | -6.42 | -6.477 |
| ZINC000003876068 | fda.1977 | -6.42 | -6.477 |
| ZINC000026664090 | fda.352 | -6.413 | -6.426 |
| ZINC000026664090 | fda.353 | -6.413 | -6.426 |
| ZINC000003830947 | fda.1399 | -6.401 | -6.401 |
| ZINC000003927870 | fda.1491 | -6.39 | -7.539 |
| ZINC000003927870 | fda.1492 | -6.39 | -7.539 |
| ZINC000002005305 | fda.22 | -6.385 | -6.401 |
| ZINC000002005305 | fda.23 | -6.385 | -6.401 |
| ZINC000085537017 | fda.2090 | -6.369 | -6.383 |
| ZINC000085537017 | fda.2091 | -6.369 | -6.383 |
| ZINC000000057254 | fda.1071 | -6.36 | -6.365 |
| ZINC000000057254 | fda.1072 | -6.36 | -6.365 |
| ZINC000000057253 | fda.1272 | -6.36 | -6.365 |
| ZINC000000057253 | fda.1273 | -6.36 | -6.365 |
| ZINC000001035331 | fda.1872 | -6.348 | -6.348 |
| ZINC000001530948 | fda.699 | -6.344 | -6.344 |
| ZINC000001530947 | fda.885 | -6.344 | -6.344 |
| ZINC000029571072 | fda.2033 | -6.332 | -6.764 |
| ZINC000049637509 | fda.2042 | -6.332 | -6.764 |
| ZINC000002005305 | fda.22 | -6.327 | -6.343 |
| ZINC000002005305 | fda.23 | -6.327 | -6.343 |
| ZINC000003604264 | fda.518 | -6.323 | -6.323 |
| ZINC000001997127 | fda.984 | -6.323 | -6.323 |
| ZINC000002005305 | fda.21 | -6.321 | -6.338 |
| ZINC000000002273 | fda.938 | -6.308 | -6.322 |
| ZINC000000000469 | fda.1514 | -6.308 | -6.322 |
| ZINC000009302239 | fda.502 | -6.303 | -6.303 |
| ZINC000004258316 | fda.180 | -6.3 | -6.769 |
| ZINC000001995484 | fda.1275 | -6.297 | -6.51 |
| ZINC000000006156 | fda.465 | -6.295 | -6.295 |
| ZINC000003831139 | fda.1700 | -6.295 | -6.295 |
| ZINC000001530929 | fda.1950 | -6.281 | -6.339 |
| ZINC000003876068 | fda.1976 | -6.281 | -6.339 |
| ZINC000028232750 | fda.175 | -6.275 | -6.275 |
| ZINC000028232750 | fda.176 | -6.261 | -6.262 |
| ZINC000001530974 | fda.690 | -6.26 | -6.639 |
| ZINC000001530973 | fda.884 | -6.26 | -6.639 |
| ZINC000043100953 | fda.1804 | -6.255 | -6.34 |
| ZINC000043100953 | fda.1805 | -6.255 | -6.34 |
| ZINC000013597823 | fda.750 | -6.246 | -6.328 |
| ZINC000013597823 | fda.751 | -6.242 | -6.325 |
| ZINC000000013156 | fda.862 | -6.228 | -6.23 |
| ZINC000005133378 | fda.1065 | -6.215 | -6.215 |
| ZINC000028232750 | fda.174 | -6.193 | -6.193 |
| ZINC000000034157 | fda.635 | -6.192 | -6.21 |
| ZINC000003843198 | fda.1002 | -6.187 | -6.19 |
| ZINC000003800475 | fda.973 | -6.187 | -6.187 |
| ZINC000000137884 | fda.1868 | -6.167 | -6.168 |
| ZINC000002539827 | fda.1383 | -6.164 | -6.184 |
| ZINC000002539827 | fda.1385 | -6.164 | -6.184 |
| ZINC000000895154 | fda.1406 | -6.164 | -6.184 |
| ZINC000000895154 | fda.1408 | -6.164 | -6.184 |
| ZINC000000002028 | fda.1255 | -6.163 | -6.167 |
| ZINC000000113355 | fda.1053 | -6.161 | -6.168 |
| ZINC000000002281 | fda.1029 | -6.155 | -6.169 |
| ZINC000000000746 | fda.149 | -6.155 | -6.169 |
| ZINC000005162311 | fda.1030 | -6.148 | -6.148 |
| ZINC000000034157 | fda.635 | -6.147 | -6.165 |
| ZINC000049036447 | fda.1202 | -6.144 | -6.144 |
| ZINC000012495062 | fda.1269 | -6.142 | -6.144 |
| ZINC000006382803 | fda.1024 | -6.14 | -6.208 |
| ZINC000003860453 | fda.960 | -6.121 | -6.337 |
| ZINC000003861768 | fda.135 | -6.104 | -6.523 |
| ZINC000000083315 | fda.1473 | -6.103 | -6.103 |
| ZINC000006467621 | fda.1988 | -6.098 | -6.664 |
| ZINC000003813003 | fda.179 | -6.097 | -6.097 |
| ZINC000001997125 | fda.983 | -6.096 | -6.096 |
| ZINC000003940470 | fda.1922 | -6.096 | -6.096 |
| ZINC000003807917 | fda.264 | -6.088 | -6.088 |
| ZINC000001485935 | fda.434 | -6.082 | -6.083 |
| ZINC000002539827 | fda.1384 | -6.078 | -6.097 |
| ZINC000000895154 | fda.1407 | -6.078 | -6.097 |
| ZINC000000896731 | fda.1356 | -6.077 | -6.096 |
| ZINC000000000850 | fda.1147 | -6.077 | -6.096 |
| ZINC000026985532 | fda.356 | -6.07 | -6.083 |
| ZINC000026985532 | fda.357 | -6.07 | -6.083 |
| ZINC000003806262 | fda.410 | -6.068 | -6.097 |
| ZINC000003806262 | fda.411 | -6.068 | -6.097 |
| ZINC000003861768 | fda.135 | -6.066 | -6.485 |
| ZINC000003995807 | fda.2073 | -6.061 | -6.061 |
| ZINC000004475353 | fda.872 | -6.059 | -6.059 |
| ZINC000003786192 | fda.1685 | -6.059 | -6.059 |
| ZINC000000967597 | fda.1861 | -6.055 | -6.055 |
| ZINC000001543475 | fda.1852 | -6.053 | -6.331 |
| ZINC000001543475 | fda.1854 | -6.053 | -6.331 |
| ZINC000003813078 | fda.603 | -6.047 | -6.05 |
| ZINC000043206370 | fda.514 | -6.047 | -6.047 |
| ZINC000000113355 | fda.1053 | -6.045 | -6.052 |
| ZINC000013298313 | fda.593 | -6.04 | -6.049 |
| ZINC000003918138 | fda.964 | -6.026 | -6.026 |
| ZINC000000009342 | fda.314 | -6.026 | -6.026 |
| ZINC000000009342 | fda.315 | -6.026 | -6.026 |
| ZINC000002525885 | fda.878 | -6.026 | -6.026 |
| ZINC000002525885 | fda.879 | -6.026 | -6.026 |
| ZINC000000002159 | fda.1048 | -6.021 | -6.022 |
| ZINC000000004785 | fda.767 | -6.02 | -6.02 |
| ZINC000001035331 | fda.1872 | -6.018 | -6.018 |
| ZINC000003830264 | fda.1107 | -6.014 | -6.014 |
| ZINC000004216238 | fda.70 | -6.01 | -6.01 |
| ZINC000003813088 | fda.1460 | -6.008 | -6.011 |
| ZINC000008855117 | fda.1348 | -6.002 | -6.002 |
| ZINC000008855117 | fda.1349 | -6.002 | -6.002 |
| ZINC000000896731 | fda.1355 | -5.991 | -6.01 |
| ZINC000000000850 | fda.1146 | -5.991 | -6.01 |
| ZINC000000004321 | fda.947 | -5.99 | -5.992 |
| ZINC000000113428 | fda.435 | -5.984 | -5.984 |
| ZINC000000113426 | fda.1006 | -5.984 | -5.984 |
| ZINC000087515509 | fda.1287 | -5.983 | -6.591 |
| ZINC000003922770 | fda.554 | -5.974 | -6.396 |
| ZINC000000002273 | fda.938 | -5.974 | -5.988 |
| ZINC000000000469 | fda.1514 | -5.974 | -5.988 |
| ZINC000001547851 | fda.488 | -5.968 | -5.968 |
| ZINC000003830990 | fda.1818 | -5.968 | -5.968 |
| ZINC000029571072 | fda.2032 | -5.96 | -6.464 |
| ZINC000049637509 | fda.2041 | -5.96 | -6.464 |
| ZINC000004474682 | fda.1230 | -5.953 | -5.953 |
| ZINC000001540998 | fda.1956 | -5.944 | -5.944 |
| ZINC000084668739 | fda.533 | -5.94 | -5.94 |
| ZINC000000001688 | fda.1150 | -5.938 | -5.941 |
| ZINC000100055899 | fda.1313 | -5.93 | -5.93 |
| ZINC000000896731 | fda.1356 | -5.929 | -5.947 |
| ZINC000000000850 | fda.1147 | -5.929 | -5.947 |
| ZINC000003810860 | fda.1710 | -5.925 | -5.926 |
| ZINC000000000853 | fda.1148 | -5.924 | -5.926 |
| ZINC000000896731 | fda.1355 | -5.924 | -5.943 |
| ZINC000000000850 | fda.1146 | -5.924 | -5.943 |
| ZINC000003875980 | fda.963 | -5.923 | -5.923 |
| ZINC000003872520 | fda.1396 | -5.922 | -5.923 |
| ZINC000085540219 | fda.1788 | -5.915 | -5.915 |
| ZINC000085540223 | fda.1789 | -5.915 | -5.915 |
| ZINC000000074836 | fda.319 | -5.913 | -5.92 |
| ZINC000000009073 | fda.1036 | -5.912 | -5.956 |
| ZINC000000009073 | fda.1037 | -5.912 | -5.956 |
| ZINC000000009073 | fda.1038 | -5.912 | -5.956 |
| ZINC000000014257 | fda.450 | -5.908 | -5.908 |
| ZINC000000896703 | fda.1354 | -5.908 | -5.908 |
| ZINC000003819392 | fda.556 | -5.905 | -5.908 |
| ZINC000000001688 | fda.1149 | -5.9 | -5.903 |
| ZINC000029571072 | fda.2033 | -5.9 | -6.404 |
| ZINC000049637509 | fda.2042 | -5.9 | -6.404 |
| ZINC000118912517 | fda.1314 | -5.9 | -5.9 |
| ZINC000000137884 | fda.1868 | -5.899 | -5.9 |
| ZINC000003798064 | fda.972 | -5.893 | -5.893 |
| ZINC000001482184 | fda.680 | -5.893 | -5.894 |
| ZINC000000000882 | fda.642 | -5.883 | -5.904 |
| ZINC000000000882 | fda.643 | -5.883 | -5.904 |
| ZINC000053084692 | fda.74 | -5.881 | -6.066 |
| ZINC000085205451 | fda.1933 | -5.877 | -5.877 |
| ZINC000001543475 | fda.1853 | -5.873 | -6.151 |
| ZINC000003842753 | fda.1735 | -5.872 | -5.957 |
| ZINC000095616603 | fda.1376 | -5.86 | -5.87 |
| ZINC000003927822 | fda.607 | -5.853 | -5.854 |
| ZINC000003927822 | fda.608 | -5.853 | -5.854 |
| ZINC000000000416 | fda.1487 | -5.852 | -5.879 |
| ZINC000000000416 | fda.1488 | -5.852 | -5.879 |
| ZINC000000403011 | fda.1771 | -5.852 | -5.879 |
| ZINC000000403011 | fda.1772 | -5.852 | -5.879 |
| ZINC000000006016 | fda.47 | -5.849 | -5.85 |
| ZINC000000006016 | fda.48 | -5.849 | -5.85 |
| ZINC000000896698 | fda.1424 | -5.849 | -5.85 |
| ZINC000000896698 | fda.1425 | -5.849 | -5.85 |
| ZINC000003875439 | fda.1883 | -5.845 | -5.845 |
| ZINC000001996117 | fda.980 | -5.844 | -5.845 |
| ZINC000000002101 | fda.201 | -5.843 | -5.843 |
| ZINC000001530806 | fda.1620 | -5.842 | -5.843 |
| ZINC000001530805 | fda.1632 | -5.842 | -5.843 |
| ZINC000000057624 | fda.630 | -5.841 | -5.859 |
| ZINC000000000271 | fda.2003 | -5.841 | -5.841 |
| ZINC000000897291 | fda.2013 | -5.841 | -5.841 |
| ZINC000006467621 | fda.1988 | -5.84 | -6.251 |
| ZINC000001530788 | fda.1873 | -5.839 | -5.839 |
| ZINC000003927870 | fda.1491 | -5.827 | -5.919 |
| ZINC000003927870 | fda.1492 | -5.827 | -5.919 |
| ZINC000001612996 | fda.10 | -5.824 | -5.827 |
| ZINC000000596881 | fda.311 | -5.822 | -6.029 |
| ZINC000000643055 | fda.1500 | -5.822 | -6.029 |
| ZINC000003927870 | fda.1491 | -5.82 | -5.912 |
| ZINC000003927870 | fda.1492 | -5.82 | -5.912 |
| ZINC000003927870 | fda.1493 | -5.82 | -5.912 |
| ZINC000003938482 | fda.1755 | -5.82 | -5.838 |
| ZINC000003982483 | fda.1892 | -5.82 | -5.83 |
| ZINC000001547851 | fda.488 | -5.817 | -5.817 |
| ZINC000003830990 | fda.1818 | -5.817 | -5.817 |
| ZINC000000895302 | fda.1057 | -5.815 | -5.815 |
| ZINC000000057464 | fda.1075 | -5.815 | -5.914 |
| ZINC000003927870 | fda.1493 | -5.803 | -5.895 |
| ZINC000000538550 | fda.941 | -5.802 | -6.472 |
| ZINC000000538550 | fda.942 | -5.802 | -6.472 |
| ZINC000000020253 | fda.842 | -5.802 | -5.803 |
| ZINC000000057255 | fda.1766 | -5.802 | -5.803 |
| ZINC000016929327 | fda.1113 | -5.799 | -6.218 |
| ZINC000000007782 | fda.860 | -5.798 | -5.798 |
| ZINC000000001084 | fda.276 | -5.79 | -5.79 |
| ZINC000018203737 | fda.367 | -5.784 | -6.135 |
| ZINC000018203737 | fda.368 | -5.784 | -6.135 |
| ZINC000003830569 | fda.119 | -5.779 | -5.779 |
| ZINC000000013156 | fda.861 | -5.776 | -5.804 |
| ZINC000000057146 | fda.1099 | -5.776 | -5.776 |
| ZINC000000057147 | fda.1100 | -5.776 | -5.776 |
| ZINC000003830264 | fda.1107 | -5.774 | -5.774 |
| ZINC000003871701 | fda.597 | -5.771 | -5.771 |
| ZINC000004216238 | fda.70 | -5.768 | -5.768 |
| ZINC000002005305 | fda.21 | -5.764 | -5.78 |
| ZINC000000509440 | fda.1477 | -5.76 | -5.76 |
| ZINC000000000575 | fda.1831 | -5.76 | -5.76 |
| ZINC000003831430 | fda.1008 | -5.752 | -5.752 |
| ZINC000003831429 | fda.1816 | -5.752 | -5.752 |
| ZINC000002599970 | fda.57 | -5.751 | -5.763 |
| ZINC000000897256 | fda.2010 | -5.751 | -5.763 |
| ZINC000003787060 | fda.1875 | -5.75 | -5.75 |
| ZINC000003871967 | fda.1395 | -5.747 | -5.747 |
| ZINC000100296832 | fda.528 | -5.746 | -5.751 |
| ZINC000100296832 | fda.529 | -5.746 | -5.751 |
| ZINC000000001758 | fda.1672 | -5.745 | -5.779 |
| ZINC000003791297 | fda.1651 | -5.744 | -5.939 |
| ZINC000001530922 | fda.1119 | -5.74 | -7.291 |
| ZINC000001530922 | fda.1121 | -5.74 | -7.291 |
| ZINC000003873921 | fda.363 | -5.739 | -5.75 |
| ZINC000001481815 | fda.791 | -5.735 | -5.741 |
| ZINC000000136138 | fda.150 | -5.731 | -5.767 |
| ZINC000000136138 | fda.151 | -5.73 | -5.766 |
| ZINC000000004009 | fda.1614 | -5.73 | -5.73 |
| ZINC000003798247 | fda.1708 | -5.727 | -5.727 |
| ZINC000003802417 | fda.2063 | -5.717 | -5.722 |
| ZINC000000601254 | fda.866 | -5.717 | -5.719 |
| ZINC000000896755 | fda.1656 | -5.717 | -5.719 |
| ZINC000003952881 | fda.649 | -5.714 | -5.72 |
| ZINC000000001464 | fda.865 | -5.706 | -5.707 |
| ZINC000003831151 | fda.1701 | -5.705 | -5.705 |
| ZINC000000000323 | fda.1841 | -5.703 | -5.704 |
| ZINC000000008667 | fda.1845 | -5.703 | -5.704 |
| ZINC000004258316 | fda.180 | -5.703 | -6.927 |
| ZINC000000009073 | fda.1036 | -5.697 | -5.741 |
| ZINC000000009073 | fda.1037 | -5.697 | -5.741 |
| ZINC000000009073 | fda.1038 | -5.697 | -5.741 |
| ZINC000003918453 | fda.1456 | -5.697 | -5.746 |
| ZINC000003800475 | fda.973 | -5.695 | -5.695 |
| ZINC000022059926 | fda.645 | -5.694 | -5.735 |
| ZINC000022059930 | fda.1165 | -5.694 | -5.735 |
| ZINC000003830947 | fda.1399 | -5.693 | -5.693 |
| ZINC000001542392 | fda.140 | -5.688 | -5.688 |
| ZINC000001843099 | fda.1781 | -5.688 | -5.688 |
| ZINC000003775644 | fda.970 | -5.682 | -5.713 |
| ZINC000003860453 | fda.960 | -5.68 | -5.896 |
| ZINC000001530639 | fda.359 | -5.678 | -5.68 |
| ZINC000100032379 | fda.1294 | -5.678 | -5.853 |
| ZINC000100032379 | fda.1295 | -5.678 | -5.853 |
| ZINC000007997568 | fda.1570 | -5.676 | -5.748 |
| ZINC000007997568 | fda.1571 | -5.676 | -5.748 |
| ZINC000003798247 | fda.1708 | -5.675 | -5.675 |
| ZINC000003913937 | fda.1885 | -5.672 | -5.674 |
| ZINC000000001655 | fda.1443 | -5.672 | -5.672 |
| ZINC000072316335 | fda.1928 | -5.671 | -5.74 |
| ZINC000004228258 | fda.1062 | -5.67 | -5.69 |
| ZINC000004228257 | fda.258 | -5.67 | -5.69 |
| ZINC000013912394 | fda.2077 | -5.67 | -5.67 |
| ZINC000013587680 | fda.88 | -5.67 | -5.673 |
| ZINC000003784120 | fda.167 | -5.67 | -5.673 |
| ZINC000004097343 | fda.955 | -5.668 | -5.69 |
| ZINC000004097344 | fda.1094 | -5.668 | -5.69 |
| ZINC000008855117 | fda.1348 | -5.668 | -5.668 |
| ZINC000008855117 | fda.1349 | -5.668 | -5.668 |
| ZINC000022002214 | fda.631 | -5.665 | -5.717 |
| ZINC000022002214 | fda.632 | -5.665 | -5.717 |
| ZINC000022002218 | fda.633 | -5.665 | -5.717 |
| ZINC000022002218 | fda.634 | -5.665 | -5.717 |
| ZINC000003938482 | fda.1755 | -5.665 | -5.683 |
| ZINC000003629271 | fda.1705 | -5.664 | -5.664 |
| ZINC000001843099 | fda.1783 | -5.661 | -5.661 |
| ZINC000002016037 | fda.656 | -5.659 | -5.919 |
| ZINC000000606383 | fda.1227 | -5.659 | -5.919 |
| ZINC000253476025 | fda.366 | -5.658 | -5.658 |
| ZINC000100015780 | fda.1283 | -5.658 | -5.658 |
| ZINC000014263142 | fda.721 | -5.657 | -5.657 |
| ZINC000000968303 | fda.1780 | -5.649 | -5.649 |
| ZINC000000057146 | fda.1099 | -5.643 | -5.643 |
| ZINC000000057147 | fda.1100 | -5.643 | -5.643 |
| ZINC000000402909 | fda.1005 | -5.64 | -5.641 |
| ZINC000000113382 | fda.1101 | -5.634 | -5.634 |
| ZINC000003831405 | fda.28 | -5.633 | -5.647 |
| ZINC000003831405 | fda.29 | -5.633 | -5.647 |
| ZINC000003978005 | fda.1235 | -5.63 | -5.68 |
| ZINC000003978005 | fda.1236 | -5.63 | -5.68 |
| ZINC000003978005 | fda.1237 | -5.63 | -5.68 |
| ZINC000003806413 | fda.377 | -5.627 | -5.627 |
| ZINC000000113382 | fda.1102 | -5.624 | -5.624 |
| ZINC000003604264 | fda.518 | -5.619 | -5.619 |
| ZINC000001997127 | fda.984 | -5.619 | -5.619 |
| ZINC000002519740 | fda.1124 | -5.619 | -5.677 |
| ZINC000002519740 | fda.1125 | -5.619 | -5.677 |
| ZINC000002519740 | fda.1126 | -5.619 | -5.677 |
| ZINC000001690324 | fda.1961 | -5.618 | -5.657 |
| ZINC000001690324 | fda.1962 | -5.618 | -5.657 |
| ZINC000000020243 | fda.1811 | -5.615 | -5.615 |
| ZINC000000968345 | fda.116 | -5.614 | -5.683 |
| ZINC000003651680 | fda.1689 | -5.614 | -5.683 |
| ZINC000000105216 | fda.1867 | -5.613 | -5.614 |
| ZINC000000006016 | fda.47 | -5.611 | -5.612 |
| ZINC000000006016 | fda.48 | -5.611 | -5.612 |
| ZINC000000896698 | fda.1424 | -5.611 | -5.612 |
| ZINC000000896698 | fda.1425 | -5.611 | -5.612 |
| ZINC000011679756 | fda.468 | -5.609 | -5.82 |
| ZINC000004212854 | fda.833 | -5.607 | -5.607 |
| ZINC000100015048 | fda.254 | -5.607 | -5.607 |
| ZINC000004474682 | fda.1230 | -5.606 | -5.606 |
| ZINC000000105216 | fda.1867 | -5.604 | -5.605 |
| ZINC000000897085 | fda.1170 | -5.596 | -5.599 |
| ZINC000000537964 | fda.2067 | -5.596 | -5.599 |
| ZINC000001886617 | fda.1073 | -5.595 | -5.597 |
| ZINC000002015928 | fda.672 | -5.593 | -5.598 |
| ZINC000002539702 | fda.237 | -5.592 | -5.592 |
| ZINC000019796155 | fda.711 | -5.589 | -5.615 |
| ZINC000019796155 | fda.712 | -5.589 | -5.615 |
| ZINC000001482164 | fda.615 | -5.584 | -5.584 |
| ZINC000000113382 | fda.1101 | -5.584 | -5.584 |
| ZINC000012503187 | fda.568 | -5.581 | -6.358 |
| ZINC000003830500 | fda.595 | -5.58 | -5.827 |
| ZINC000058581064 | fda.1199 | -5.58 | -6.205 |
| ZINC000009302239 | fda.502 | -5.579 | -5.579 |
| ZINC000000020255 | fda.1422 | -5.579 | -5.579 |
| ZINC000000005823 | fda.45 | -5.577 | -5.577 |
| ZINC000003813010 | fda.105 | -5.575 | -5.605 |
| ZINC000003830347 | fda.196 | -5.573 | -7.064 |
| ZINC000003830347 | fda.197 | -5.573 | -7.064 |
| ZINC000018043251 | fda.284 | -5.573 | -5.605 |
| ZINC000000008492 | fda.7 | -5.569 | -5.587 |
| ZINC000000004166 | fda.449 | -5.566 | -5.588 |
| ZINC000000896819 | fda.1081 | -5.566 | -5.588 |
| ZINC000000003642 | fda.1156 | -5.564 | -5.565 |
| ZINC000000057313 | fda.1274 | -5.564 | -5.565 |
| ZINC000004228258 | fda.1061 | -5.564 | -5.584 |
| ZINC000004228258 | fda.1063 | -5.564 | -5.584 |
| ZINC000004228257 | fda.257 | -5.564 | -5.584 |
| ZINC000004228257 | fda.259 | -5.564 | -5.584 |
| ZINC000027990463 | fda.1320 | -5.563 | -5.566 |
| ZINC000003819138 | fda.1686 | -5.563 | -5.563 |
| ZINC000000000431 | fda.1518 | -5.562 | -5.562 |
| ZINC000000896595 | fda.1643 | -5.562 | -5.562 |
| ZINC000002599970 | fda.56 | -5.559 | -5.572 |
| ZINC000000897256 | fda.2009 | -5.559 | -5.572 |
| ZINC000002032615 | fda.943 | -5.558 | -5.558 |
| ZINC000001850377 | fda.1430 | -5.558 | -5.558 |
| ZINC000003798757 | fda.1740 | -5.551 | -5.756 |
| ZINC000085540219 | fda.1788 | -5.549 | -5.549 |
| ZINC000085540223 | fda.1789 | -5.549 | -5.549 |
| ZINC000002599970 | fda.57 | -5.548 | -5.56 |
| ZINC000000897256 | fda.2010 | -5.548 | -5.56 |
| ZINC000005844788 | fda.188 | -5.545 | -5.565 |
| ZINC000000968328 | fda.403 | -5.545 | -5.987 |
| ZINC000000968330 | fda.1173 | -5.545 | -5.987 |
| ZINC000001543475 | fda.1852 | -5.538 | -5.816 |
| ZINC000001543475 | fda.1854 | -5.538 | -5.816 |
| ZINC000035328014 | fda.1555 | -5.537 | -5.946 |
| ZINC000006827695 | fda.1409 | -5.534 | -6.235 |
| ZINC000006827695 | fda.1410 | -5.534 | -6.235 |
| ZINC000006827695 | fda.1411 | -5.534 | -6.235 |
| ZINC000002000707 | fda.354 | -5.533 | -5.533 |
| ZINC000000004724 | fda.1360 | -5.533 | -5.533 |
| ZINC000005844788 | fda.187 | -5.532 | -5.552 |
| ZINC000011677857 | fda.479 | -5.532 | -5.569 |
| ZINC000012503187 | fda.567 | -5.532 | -6.308 |
| ZINC000040899447 | fda.600 | -5.53 | -5.53 |
| ZINC000000120319 | fda.1515 | -5.526 | -5.682 |
| ZINC000000120319 | fda.1516 | -5.526 | -5.682 |
| ZINC000095616603 | fda.1376 | -5.525 | -5.535 |
| ZINC000000607986 | fda.63 | -5.523 | -5.543 |
| ZINC000000895199 | fda.1044 | -5.523 | -5.523 |
| ZINC000003806721 | fda.1714 | -5.522 | -5.567 |
| ZINC000006858022 | fda.1405 | -5.519 | -5.523 |
| ZINC000001995484 | fda.1276 | -5.518 | -5.732 |
| ZINC000001883067 | fda.1865 | -5.515 | -5.515 |
| ZINC000000601250 | fda.1505 | -5.514 | -5.845 |
| ZINC000003873371 | fda.1092 | -5.514 | -5.845 |
| ZINC000003873371 | fda.1093 | -5.514 | -5.845 |
| ZINC000004097343 | fda.955 | -5.511 | -5.532 |
| ZINC000004097344 | fda.1094 | -5.511 | -5.532 |
| ZINC000003939013 | fda.1085 | -5.51 | -5.516 |
| ZINC000028467879 | fda.1903 | -5.507 | -5.507 |
| ZINC000028467879 | fda.1904 | -5.507 | -5.507 |
| ZINC000003982483 | fda.1891 | -5.506 | -5.516 |
| ZINC000003982483 | fda.1893 | -5.506 | -5.516 |
| ZINC000003798064 | fda.972 | -5.505 | -5.505 |
| ZINC000026664090 | fda.352 | -5.502 | -5.515 |
| ZINC000026664090 | fda.353 | -5.502 | -5.515 |
| ZINC000006382803 | fda.1023 | -5.5 | -5.65 |
| ZINC000000005151 | fda.1418 | -5.5 | -5.506 |
| ZINC000000005151 | fda.1419 | -5.5 | -5.506 |
| ZINC000000001773 | fda.1843 | -5.497 | -5.549 |
| ZINC000000001773 | fda.1844 | -5.497 | -5.549 |
| ZINC000100296828 | fda.526 | -5.49 | -5.495 |
| ZINC000100296828 | fda.527 | -5.49 | -5.495 |
| ZINC000035342789 | fda.1544 | -5.49 | -5.493 |
| ZINC000003803652 | fda.830 | -5.49 | -5.996 |
| ZINC000000113382 | fda.1102 | -5.49 | -5.49 |
| ZINC000027428713 | fda.1301 | -5.49 | -5.49 |
| ZINC000053084692 | fda.75 | -5.485 | -5.671 |
| ZINC000003784182 | fda.1696 | -5.485 | -5.485 |
| ZINC000019796080 | fda.714 | -5.484 | -5.582 |
| ZINC000019796080 | fda.715 | -5.484 | -5.582 |
| ZINC000019796080 | fda.716 | -5.484 | -5.582 |
| ZINC000003921872 | fda.198 | -5.48 | -5.48 |
| ZINC000000601283 | fda.869 | -5.479 | -5.479 |
| ZINC000003842753 | fda.1734 | -5.478 | -5.563 |
| ZINC000000001644 | fda.1412 | -5.477 | -5.507 |
| ZINC000001543475 | fda.1853 | -5.477 | -5.755 |
| ZINC000003831282 | fda.1108 | -5.475 | -5.475 |
| ZINC000000895199 | fda.1044 | -5.475 | -5.475 |
| ZINC000004693575 | fda.323 | -5.475 | -5.568 |
| ZINC000004693574 | fda.1217 | -5.475 | -5.568 |
| ZINC000001530929 | fda.1951 | -5.475 | -5.532 |
| ZINC000003876068 | fda.1977 | -5.475 | -5.532 |
| ZINC000036701290 | fda.519 | -5.475 | -5.681 |
| ZINC000036701290 | fda.520 | -5.475 | -5.681 |
| ZINC000003827556 | fda.1972 | -5.474 | -5.518 |
| ZINC000003871967 | fda.1395 | -5.473 | -5.473 |
| ZINC000000056652 | fda.1051 | -5.472 | -5.472 |
| ZINC000000056653 | fda.1052 | -5.472 | -5.472 |
| ZINC000000007295 | fda.816 | -5.472 | -5.472 |
| ZINC000072318121 | fda.2001 | -5.47 | -6.288 |
| ZINC000072318121 | fda.2002 | -5.47 | -6.288 |
| ZINC000003806063 | fda.106 | -5.466 | -5.469 |
| ZINC000035801098 | fda.1548 | -5.464 | -5.506 |
| ZINC000003812869 | fda.2047 | -5.459 | -5.708 |
| ZINC000000001261 | fda.1556 | -5.457 | -5.458 |
| ZINC000000105196 | fda.610 | -5.456 | -5.456 |
| ZINC000003927870 | fda.1493 | -5.456 | -6.605 |
| ZINC000001530283 | fda.1393 | -5.456 | -5.456 |
| ZINC000006021033 | fda.1021 | -5.453 | -5.454 |
| ZINC000006036847 | fda.1022 | -5.453 | -5.454 |
| ZINC000003842753 | fda.1735 | -5.453 | -5.537 |
| ZINC000253632968 | fda.2093 | -5.452 | -5.458 |
| ZINC000036520252 | fda.1594 | -5.451 | -5.451 |
| ZINC000014210642 | fda.784 | -5.45 | -5.45 |
| ZINC000014210642 | fda.785 | -5.45 | -5.45 |
| ZINC000000897408 | fda.445 | -5.45 | -5.45 |
| ZINC000000538564 | fda.110 | -5.45 | -5.45 |
| ZINC000052957434 | fda.1207 | -5.447 | -5.49 |
| ZINC000052957434 | fda.1208 | -5.447 | -5.49 |
| ZINC000000074836 | fda.319 | -5.435 | -5.442 |
| ZINC000000001132 | fda.1941 | -5.435 | -5.436 |
| ZINC000003812869 | fda.2048 | -5.434 | -5.684 |
| ZINC000003922770 | fda.554 | -5.431 | -5.831 |
| ZINC000002008310 | fda.1966 | -5.428 | -5.428 |
| ZINC000003812983 | fda.1482 | -5.426 | -5.484 |
| ZINC000003812983 | fda.1483 | -5.426 | -5.484 |
| ZINC000003812862 | fda.429 | -5.425 | -5.536 |
| ZINC000000599985 | fda.1494 | -5.424 | -5.424 |
| ZINC000116473771 | fda.539 | -5.423 | -5.43 |
| ZINC000100017856 | fda.1959 | -5.423 | -5.43 |
| ZINC000100017856 | fda.1960 | -5.423 | -5.43 |
| ZINC000118912393 | fda.1248 | -5.421 | -5.421 |
| ZINC000000000323 | fda.1841 | -5.421 | -5.422 |
| ZINC000000008667 | fda.1845 | -5.421 | -5.422 |
| ZINC000028973441 | fda.1995 | -5.418 | -5.418 |
| ZINC000028973446 | fda.1996 | -5.418 | -5.418 |
| ZINC000000020250 | fda.1609 | -5.417 | -5.418 |
| ZINC000000056647 | fda.1271 | -5.417 | -5.418 |
| ZINC000003812862 | fda.430 | -5.413 | -5.524 |
| ZINC000001996784 | fda.982 | -5.413 | -6.264 |
| ZINC000005133329 | fda.1247 | -5.413 | -5.413 |
| ZINC000000020220 | fda.369 | -5.411 | -5.511 |
| ZINC000003806262 | fda.410 | -5.411 | -5.44 |
| ZINC000003806262 | fda.411 | -5.411 | -5.44 |
| ZINC000004693575 | fda.324 | -5.41 | -5.444 |
| ZINC000004693574 | fda.1218 | -5.41 | -5.444 |
| ZINC000003914810 | fda.267 | -5.409 | -5.414 |
| ZINC000014961096 | fda.19 | -5.409 | -5.409 |
| ZINC000003830391 | fda.362 | -5.406 | -5.615 |
| ZINC000003871960 | fda.1737 | -5.402 | -5.402 |
| ZINC000001886617 | fda.1073 | -5.399 | -5.4 |
| ZINC000001280665 | fda.412 | -5.398 | -5.427 |
| ZINC000001280665 | fda.414 | -5.398 | -5.427 |
| ZINC000003830713 | fda.1389 | -5.395 | -5.398 |
| ZINC000001530806 | fda.1620 | -5.395 | -5.396 |
| ZINC000001530805 | fda.1632 | -5.395 | -5.396 |
| ZINC000003812863 | fda.1648 | -5.395 | -5.517 |
| ZINC000000057624 | fda.630 | -5.392 | -7.486 |
| ZINC000003812984 | fda.1640 | -5.391 | -5.395 |
| ZINC000003812984 | fda.1641 | -5.391 | -5.395 |
| ZINC000019632917 | fda.710 | -5.389 | -5.485 |
| ZINC000000049154 | fda.644 | -5.389 | -5.613 |
| ZINC000100007011 | fda.1377 | -5.388 | -5.388 |
| ZINC000100071256 | fda.1918 | -5.388 | -5.388 |
| ZINC000003964126 | fda.651 | -5.388 | -5.388 |
| ZINC000000000853 | fda.1148 | -5.387 | -5.39 |
| ZINC000003813010 | fda.104 | -5.387 | -5.417 |
| ZINC000003824921 | fda.208 | -5.386 | -5.386 |
| ZINC000003872566 | fda.216 | -5.386 | -5.386 |
| ZINC000013682481 | fda.752 | -5.385 | -5.522 |
| ZINC000013682481 | fda.753 | -5.385 | -5.522 |
| ZINC000000034157 | fda.635 | -5.382 | -7.477 |
| ZINC000013648755 | fda.418 | -5.377 | -6.418 |
| ZINC000087515509 | fda.1287 | -5.376 | -6.304 |
| ZINC000100006770 | fda.278 | -5.375 | -5.375 |
| ZINC000001482164 | fda.615 | -5.374 | -5.374 |
| ZINC000035328014 | fda.1554 | -5.373 | -5.373 |
| ZINC000003812863 | fda.1648 | -5.369 | -5.491 |
| ZINC000000025958 | fda.61 | -5.366 | -5.381 |
| ZINC000000000931 | fda.1670 | -5.365 | -5.379 |
| ZINC000000000931 | fda.1671 | -5.365 | -5.379 |
| ZINC000027428713 | fda.1302 | -5.361 | -5.361 |
| ZINC000000020259 | fda.706 | -5.36 | -5.367 |
| ZINC000001531009 | fda.931 | -5.358 | -5.546 |
| ZINC000000389747 | fda.32 | -5.357 | -5.481 |
| ZINC000000389747 | fda.33 | -5.357 | -5.481 |
| ZINC000000389747 | fda.34 | -5.357 | -5.481 |
| ZINC000003830959 | fda.628 | -5.357 | -5.357 |
| ZINC000003830957 | fda.1739 | -5.357 | -5.357 |
| ZINC000001611274 | fda.585 | -5.355 | -5.623 |
| ZINC000001611274 | fda.586 | -5.355 | -5.623 |
| ZINC000003875368 | fda.336 | -5.354 | -5.427 |
| ZINC000003875368 | fda.337 | -5.354 | -5.427 |
| ZINC000000001706 | fda.1254 | -5.353 | -5.353 |
| ZINC000003806721 | fda.1715 | -5.353 | -5.398 |
| ZINC000000005878 | fda.1180 | -5.352 | -5.352 |
| ZINC000029571072 | fda.2032 | -5.351 | -7.256 |
| ZINC000049637509 | fda.2041 | -5.351 | -7.256 |
| ZINC000001535101 | fda.1667 | -5.35 | -5.352 |
| ZINC000022010649 | fda.485 | -5.35 | -5.377 |
| ZINC000001530776 | fda.1103 | -5.347 | -5.347 |
| ZINC000100378061 | fda.531 | -5.347 | -5.713 |
| ZINC000000599985 | fda.1495 | -5.339 | -5.343 |
| ZINC000052955754 | fda.129 | -5.336 | -6.201 |
| ZINC000052955754 | fda.130 | -5.336 | -6.201 |
| ZINC000052955754 | fda.131 | -5.336 | -6.201 |
| ZINC000000002191 | fda.1660 | -5.334 | -5.334 |
| ZINC000002548959 | fda.27 | -5.333 | -5.333 |
| ZINC000100014475 | fda.77 | -5.333 | -5.333 |
| ZINC000000584092 | fda.1525 | -5.332 | -5.332 |
| ZINC000019796158 | fda.726 | -5.33 | -5.363 |
| ZINC000019796158 | fda.727 | -5.33 | -5.363 |
| ZINC000003830500 | fda.596 | -5.33 | -5.577 |
| ZINC000003816292 | fda.2060 | -5.33 | -5.33 |
| ZINC000028232750 | fda.176 | -5.323 | -5.323 |
| ZINC000043207238 | fda.1851 | -5.32 | -5.32 |
| ZINC000001530752 | fda.152 | -5.318 | -5.397 |
| ZINC000001530752 | fda.153 | -5.318 | -5.397 |
| ZINC000001530751 | fda.202 | -5.318 | -5.397 |
| ZINC000001530751 | fda.203 | -5.318 | -5.397 |
| ZINC000003831151 | fda.1701 | -5.317 | -5.317 |
| ZINC000003831404 | fda.30 | -5.316 | -5.331 |
| ZINC000003831404 | fda.31 | -5.316 | -5.331 |
| ZINC000000518554 | fda.1536 | -5.316 | -5.316 |
| ZINC000003802690 | fda.227 | -5.314 | -5.314 |
| ZINC000011681534 | fda.2021 | -5.312 | -5.332 |
| ZINC000016929327 | fda.1113 | -5.312 | -5.731 |
| ZINC000034806477 | fda.484 | -5.312 | -5.312 |
| ZINC000000002279 | fda.477 | -5.308 | -5.308 |
| ZINC000000011012 | fda.1721 | -5.308 | -5.308 |
| ZINC000001853205 | fda.1750 | -5.307 | -5.512 |
| ZINC000030691736 | fda.1341 | -5.307 | -5.307 |
| ZINC000000538550 | fda.941 | -5.305 | -5.536 |
| ZINC000000538550 | fda.942 | -5.305 | -5.536 |
| ZINC000000491073 | fda.977 | -5.304 | -5.321 |
| ZINC000000491073 | fda.978 | -5.304 | -5.321 |
| ZINC000000491073 | fda.979 | -5.304 | -5.321 |
| ZINC000000056652 | fda.1051 | -5.304 | -5.304 |
| ZINC000000056653 | fda.1052 | -5.304 | -5.304 |
| ZINC000006382803 | fda.1023 | -5.302 | -6.222 |
| ZINC000034636383 | fda.2083 | -5.301 | -5.308 |
| ZINC000043100953 | fda.1804 | -5.301 | -5.386 |
| ZINC000043100953 | fda.1805 | -5.301 | -5.386 |
| ZINC000043100953 | fda.1806 | -5.301 | -5.386 |
| ZINC000036520252 | fda.1594 | -5.299 | -5.299 |
| ZINC000003995809 | fda.1239 | -5.298 | -5.298 |
| ZINC000000005423 | fda.442 | -5.298 | -5.299 |
| ZINC000004228258 | fda.1061 | -5.296 | -5.316 |
| ZINC000004228258 | fda.1063 | -5.296 | -5.316 |
| ZINC000004228257 | fda.257 | -5.296 | -5.316 |
| ZINC000004228257 | fda.259 | -5.296 | -5.316 |
| ZINC000002169830 | fda.1400 | -5.296 | -5.296 |
| ZINC000008214703 | fda.511 | -5.295 | -5.298 |
| ZINC000003914596 | fda.551 | -5.295 | -5.308 |
| ZINC000001485935 | fda.434 | -5.293 | -5.294 |
| ZINC000000002688 | fda.1945 | -5.293 | -5.529 |
| ZINC000000049153 | fda.277 | -5.292 | -5.349 |
| ZINC000052716421 | fda.1185 | -5.291 | -5.302 |
| ZINC000052716421 | fda.1186 | -5.291 | -5.302 |
| ZINC000001542392 | fda.141 | -5.289 | -5.297 |
| ZINC000000001554 | fda.1045 | -5.288 | -5.288 |
| ZINC000000000941 | fda.1440 | -5.287 | -5.287 |
| ZINC000013818943 | fda.398 | -5.281 | -5.281 |
| ZINC000003927822 | fda.607 | -5.28 | -5.281 |
| ZINC000003927822 | fda.608 | -5.28 | -5.281 |
| ZINC000000901736 | fda.609 | -5.279 | -5.279 |
| ZINC000000895457 | fda.614 | -5.279 | -5.279 |
| ZINC000000388462 | fda.1478 | -5.277 | -5.278 |
| ZINC000011681534 | fda.2022 | -5.277 | -5.297 |
| ZINC000001280665 | fda.413 | -5.277 | -5.306 |
| ZINC000001611274 | fda.586 | -5.275 | -5.543 |
| ZINC000001550499 | fda.1859 | -5.274 | -5.285 |
| ZINC000003812869 | fda.2047 | -5.272 | -5.521 |
| ZINC000000020250 | fda.1609 | -5.271 | -5.273 |
| ZINC000000056647 | fda.1271 | -5.271 | -5.273 |
| ZINC000035024346 | fda.1252 | -5.271 | -5.275 |
| ZINC000001531009 | fda.930 | -5.271 | -5.459 |
| ZINC000022002214 | fda.631 | -5.271 | -5.323 |
| ZINC000022002214 | fda.632 | -5.271 | -5.323 |
| ZINC000022002218 | fda.633 | -5.271 | -5.323 |
| ZINC000022002218 | fda.634 | -5.271 | -5.323 |
| ZINC000008403947 | fda.128 | -5.27 | -5.271 |
| ZINC000001489478 | fda.458 | -5.266 | -5.269 |
| ZINC000000402909 | fda.1005 | -5.265 | -5.265 |
| ZINC000003871960 | fda.1737 | -5.265 | -5.265 |
| ZINC000000014864 | fda.451 | -5.264 | -5.264 |
| ZINC000003831282 | fda.1108 | -5.264 | -5.264 |
| ZINC000003806413 | fda.378 | -5.261 | -5.261 |
| ZINC000000491073 | fda.977 | -5.26 | -5.277 |
| ZINC000000491073 | fda.978 | -5.26 | -5.277 |
| ZINC000000491073 | fda.979 | -5.26 | -5.277 |
| ZINC000000012346 | fda.1592 | -5.258 | -5.258 |
| ZINC000000006156 | fda.465 | -5.257 | -5.257 |
| ZINC000003831139 | fda.1700 | -5.257 | -5.257 |
| ZINC000000000922 | fda.1035 | -5.257 | -5.258 |
| ZINC000022448696 | fda.1823 | -5.257 | -5.899 |
| ZINC000022448696 | fda.1824 | -5.257 | -5.899 |
| ZINC000022448696 | fda.1827 | -5.257 | -5.899 |
| ZINC000006827695 | fda.1409 | -5.256 | -5.489 |
| ZINC000006827695 | fda.1410 | -5.256 | -5.489 |
| ZINC000006827695 | fda.1411 | -5.256 | -5.489 |
| ZINC000001482049 | fda.164 | -5.255 | -5.255 |
| ZINC000003813010 | fda.105 | -5.255 | -5.285 |
| ZINC000003927198 | fda.1490 | -5.253 | -5.253 |
| ZINC000002539702 | fda.237 | -5.252 | -5.253 |
| ZINC000000002272 | fda.476 | -5.25 | -5.25 |
| ZINC000000005560 | fda.41 | -5.25 | -5.25 |
| ZINC000000537805 | fda.581 | -5.249 | -5.27 |
| ZINC000000537805 | fda.582 | -5.249 | -5.27 |
| ZINC000000537805 | fda.583 | -5.249 | -5.27 |
| ZINC000001997125 | fda.983 | -5.246 | -5.246 |
| ZINC000003940470 | fda.1922 | -5.246 | -5.246 |
| ZINC000085540215 | fda.1787 | -5.243 | -5.243 |
| ZINC000008214418 | fda.1992 | -5.243 | -5.243 |
| ZINC000000897408 | fda.445 | -5.239 | -5.239 |
| ZINC000000538564 | fda.110 | -5.239 | -5.239 |
| ZINC000000538621 | fda.853 | -5.236 | -5.334 |
| ZINC000000000061 | fda.219 | -5.235 | -5.235 |
| ZINC000000085733 | fda.220 | -5.235 | -5.235 |
| ZINC000000004076 | fda.291 | -5.235 | -5.237 |
| ZINC000011677376 | fda.2018 | -5.233 | -5.237 |
| ZINC000011677376 | fda.2019 | -5.233 | -5.237 |
| ZINC000011677376 | fda.2020 | -5.233 | -5.237 |
| ZINC000000897385 | fda.1427 | -5.232 | -5.491 |
| ZINC000000608101 | fda.1229 | -5.232 | -5.491 |
| ZINC000013587680 | fda.88 | -5.232 | -5.235 |
| ZINC000003784120 | fda.167 | -5.232 | -5.235 |
| ZINC000002847375 | fda.1527 | -5.231 | -5.375 |
| ZINC000002847375 | fda.1528 | -5.231 | -5.375 |
| ZINC000000000215 | fda.1837 | -5.231 | -5.375 |
| ZINC000000000215 | fda.1838 | -5.231 | -5.375 |
| ZINC000043206370 | fda.514 | -5.229 | -5.229 |
| ZINC000003990451 | fda.1238 | -5.228 | -5.232 |
| ZINC000001530948 | fda.699 | -5.227 | -5.227 |
| ZINC000001530947 | fda.885 | -5.227 | -5.227 |
| ZINC000164760756 | fda.1091 | -5.227 | -5.233 |
| ZINC000094566093 | fda.1304 | -5.221 | -5.654 |
| ZINC000052971887 | fda.1188 | -5.219 | -5.499 |
| ZINC000053022902 | fda.1209 | -5.219 | -5.499 |
| ZINC000053022902 | fda.1210 | -5.219 | -5.499 |
| ZINC000003861806 | fda.24 | -5.219 | -5.219 |
| ZINC000003874715 | fda.1386 | -5.219 | -5.219 |
| ZINC000000000053 | fda.1025 | -5.218 | -5.218 |
| ZINC000003927198 | fda.1489 | -5.218 | -5.218 |
| ZINC000003875560 | fda.1392 | -5.217 | -5.217 |
| ZINC000084668739 | fda.533 | -5.21 | -5.21 |
| ZINC000003830441 | fda.2061 | -5.21 | -5.21 |
| ZINC000100378061 | fda.530 | -5.21 | -5.576 |
| ZINC000100378061 | fda.532 | -5.21 | -5.576 |
| ZINC000003812862 | fda.431 | -5.21 | -5.32 |
| ZINC000007997568 | fda.1570 | -5.209 | -6.49 |
| ZINC000007997568 | fda.1571 | -5.209 | -6.49 |
| ZINC000003824921 | fda.208 | -5.208 | -5.208 |
| ZINC000003872566 | fda.216 | -5.208 | -5.208 |
| ZINC000001531009 | fda.932 | -5.208 | -5.397 |
| ZINC000000002159 | fda.1048 | -5.206 | -5.207 |
| ZINC000003982483 | fda.1892 | -5.204 | -5.214 |
| ZINC000003812863 | fda.1649 | -5.204 | -5.326 |
| ZINC000000538658 | fda.864 | -5.203 | -5.203 |
| ZINC000000001382 | fda.1000 | -5.202 | -5.202 |
| ZINC000004213946 | fda.950 | -5.201 | -5.221 |
| ZINC000005844792 | fda.1310 | -5.201 | -5.221 |
| ZINC000000113428 | fda.435 | -5.2 | -5.2 |
| ZINC000000113426 | fda.1006 | -5.2 | -5.2 |
| ZINC000006021033 | fda.1021 | -5.2 | -5.2 |
| ZINC000006036847 | fda.1022 | -5.2 | -5.2 |
| ZINC000000537795 | fda.847 | -5.198 | -5.198 |
| ZINC000009164421 | fda.2017 | -5.194 | -5.196 |
| ZINC000000010164 | fda.379 | -5.194 | -5.196 |
| ZINC000000010164 | fda.380 | -5.194 | -5.196 |
| ZINC000000010163 | fda.524 | -5.194 | -5.196 |
| ZINC000000010163 | fda.525 | -5.194 | -5.196 |
| ZINC000001530974 | fda.689 | -5.192 | -5.571 |
| ZINC000001530973 | fda.883 | -5.192 | -5.571 |
| ZINC000002005550 | fda.154 | -5.192 | -5.289 |
| ZINC000002005550 | fda.155 | -5.192 | -5.289 |
| ZINC000003874950 | fda.229 | -5.192 | -5.289 |
| ZINC000003874950 | fda.230 | -5.192 | -5.289 |
| ZINC000002570817 | fda.55 | -5.191 | -5.192 |
| ZINC000000000509 | fda.1549 | -5.191 | -5.279 |
| ZINC000000000509 | fda.1550 | -5.191 | -5.279 |
| ZINC000000000509 | fda.1551 | -5.191 | -5.279 |
| ZINC000003803652 | fda.830 | -5.19 | -5.734 |
| ZINC000028639340 | fda.177 | -5.19 | -5.208 |
| ZINC000000015515 | fda.1604 | -5.188 | -5.192 |
| ZINC000013831141 | fda.2075 | -5.187 | -5.187 |
| ZINC000000402954 | fda.965 | -5.187 | -5.248 |
| ZINC000000402954 | fda.966 | -5.187 | -5.248 |
| ZINC000000402954 | fda.967 | -5.187 | -5.248 |
| ZINC000019632706 | fda.382 | -5.187 | -5.187 |
| ZINC000000591993 | fda.383 | -5.187 | -5.187 |
| ZINC000001530600 | fda.1143 | -5.186 | -5.739 |
| ZINC000001530600 | fda.1144 | -5.186 | -5.739 |
| ZINC000001530600 | fda.1145 | -5.186 | -5.739 |
| ZINC000000000271 | fda.2003 | -5.184 | -5.184 |
| ZINC000000897291 | fda.2013 | -5.184 | -5.184 |
| ZINC000003876186 | fda.1390 | -5.183 | -5.183 |
| ZINC000002036848 | fda.279 | -5.181 | -5.181 |
| ZINC000002036848 | fda.280 | -5.181 | -5.181 |
| ZINC000000020259 | fda.706 | -5.181 | -5.187 |
| ZINC000000014257 | fda.450 | -5.179 | -5.179 |
| ZINC000000896703 | fda.1354 | -5.179 | -5.179 |
| ZINC000000010164 | fda.379 | -5.177 | -5.179 |
| ZINC000000010164 | fda.380 | -5.177 | -5.179 |
| ZINC000000010163 | fda.524 | -5.177 | -5.179 |
| ZINC000000010163 | fda.525 | -5.177 | -5.179 |
| ZINC000000033882 | fda.1067 | -5.174 | -5.174 |
| ZINC000000006226 | fda.1866 | -5.172 | -5.172 |
| ZINC000004215736 | fda.421 | -5.171 | -5.185 |
| ZINC000004215736 | fda.422 | -5.171 | -5.185 |
| ZINC000000968328 | fda.402 | -5.171 | -5.738 |
| ZINC000000968330 | fda.1172 | -5.171 | -5.738 |
| ZINC000003812862 | fda.430 | -5.171 | -5.281 |
| ZINC000034220093 | fda.1366 | -5.169 | -5.183 |
| ZINC000003830986 | fda.1012 | -5.169 | -5.188 |
| ZINC000000599734 | fda.1496 | -5.169 | -5.188 |
| ZINC000000601250 | fda.1504 | -5.168 | -5.499 |
| ZINC000009212428 | fda.270 | -5.167 | -5.167 |
| ZINC000009212427 | fda.1351 | -5.167 | -5.167 |
| ZINC000003830813 | fda.1653 | -5.167 | -5.413 |
| ZINC000013597823 | fda.751 | -5.163 | -5.245 |
| ZINC000003812862 | fda.429 | -5.162 | -5.273 |
| ZINC000001530975 | fda.888 | -5.162 | -5.295 |
| ZINC000001530975 | fda.889 | -5.162 | -5.295 |
| ZINC000003812897 | fda.1059 | -5.16 | -5.16 |
| ZINC000043207851 | fda.1830 | -5.159 | -5.159 |
| ZINC000035902489 | fda.1538 | -5.157 | -5.157 |
| ZINC000004215736 | fda.421 | -5.155 | -5.169 |
| ZINC000004215736 | fda.422 | -5.155 | -5.169 |
| ZINC000000004893 | fda.2004 | -5.153 | -5.157 |
| ZINC000000004893 | fda.2005 | -5.153 | -5.157 |
| ZINC000000004893 | fda.2006 | -5.153 | -5.157 |
| ZINC000003801919 | fda.1466 | -5.151 | -5.35 |
| ZINC000001850374 | fda.2049 | -5.151 | -5.151 |
| ZINC000003872931 | fda.546 | -5.15 | -5.156 |
| ZINC000000001145 | fda.803 | -5.148 | -5.148 |
| ZINC000000001145 | fda.804 | -5.148 | -5.148 |
| ZINC000003989268 | fda.2071 | -5.143 | -5.144 |
| ZINC000096006023 | fda.2098 | -5.143 | -5.144 |
| ZINC000006858022 | fda.1405 | -5.14 | -5.144 |
| ZINC000000004166 | fda.449 | -5.139 | -5.16 |
| ZINC000000896819 | fda.1081 | -5.139 | -5.16 |
| ZINC000003776970 | fda.1692 | -5.139 | -5.421 |
| ZINC000019632917 | fda.710 | -5.138 | -7.003 |
| ZINC000005179119 | fda.1031 | -5.138 | -5.155 |
| ZINC000003936683 | fda.1437 | -5.137 | -5.14 |
| ZINC000003936683 | fda.1438 | -5.137 | -5.14 |
| ZINC000000395010 | fda.534 | -5.137 | -5.204 |
| ZINC000001530600 | fda.1143 | -5.137 | -5.433 |
| ZINC000001530600 | fda.1145 | -5.137 | -5.433 |
| ZINC000000119344 | fda.1444 | -5.133 | -5.134 |
| ZINC000058581064 | fda.1199 | -5.132 | -5.419 |
| ZINC000003782807 | fda.971 | -5.132 | -5.132 |
| ZINC000004428529 | fda.1382 | -5.131 | -5.131 |
| ZINC000002016037 | fda.655 | -5.131 | -5.39 |
| ZINC000000606383 | fda.1226 | -5.131 | -5.39 |
| ZINC000000602128 | fda.49 | -5.131 | -5.136 |
| ZINC000003871703 | fda.1451 | -5.131 | -5.136 |
| ZINC000004099200 | fda.836 | -5.13 | -5.432 |
| ZINC000004676424 | fda.881 | -5.13 | -5.432 |
| ZINC000013912394 | fda.2077 | -5.13 | -5.13 |
| ZINC000003830986 | fda.1012 | -5.13 | -5.149 |
| ZINC000000599734 | fda.1496 | -5.13 | -5.149 |
| ZINC000004097427 | fda.1215 | -5.127 | -5.283 |
| ZINC000013648755 | fda.417 | -5.125 | -6.167 |
| ZINC000003815424 | fda.132 | -5.122 | -5.122 |
| ZINC000003986735 | fda.990 | -5.12 | -5.542 |
| ZINC000003986735 | fda.991 | -5.12 | -5.542 |
| ZINC000003939013 | fda.1085 | -5.118 | -5.124 |
| ZINC000002015035 | fda.1967 | -5.118 | -5.118 |
| ZINC000002015035 | fda.1968 | -5.118 | -5.118 |
| ZINC000029319828 | fda.1284 | -5.117 | -5.15 |
| ZINC000000403533 | fda.1176 | -5.117 | -5.214 |
| ZINC000000403533 | fda.1177 | -5.117 | -5.214 |
| ZINC000000403533 | fda.1178 | -5.117 | -5.214 |
| ZINC000013818943 | fda.398 | -5.116 | -5.116 |
| ZINC000000035804 | fda.1605 | -5.115 | -5.115 |
| ZINC000003830391 | fda.361 | -5.113 | -5.322 |
| ZINC000026011099 | fda.39 | -5.112 | -5.225 |
| ZINC000026011099 | fda.40 | -5.112 | -5.225 |
| ZINC000019632917 | fda.710 | -5.111 | -5.207 |
| ZINC000000968328 | fda.402 | -5.109 | -5.675 |
| ZINC000000968330 | fda.1172 | -5.109 | -5.675 |
| ZINC000072267023 | fda.1703 | -5.106 | -5.112 |
| ZINC000072267023 | fda.1704 | -5.106 | -5.112 |
| ZINC000000004028 | fda.1615 | -5.105 | -5.111 |
| ZINC000000004028 | fda.1616 | -5.105 | -5.111 |
| ZINC000003806413 | fda.378 | -5.104 | -5.104 |
| ZINC000000896546 | fda.11 | -5.104 | -5.105 |
| ZINC000000896546 | fda.12 | -5.104 | -5.105 |
| ZINC000000083315 | fda.1473 | -5.104 | -5.104 |
| ZINC000000000061 | fda.219 | -5.103 | -5.103 |
| ZINC000000085733 | fda.220 | -5.103 | -5.103 |
| ZINC000003808779 | fda.974 | -5.102 | -5.131 |
| ZINC000003801919 | fda.1468 | -5.102 | -5.301 |
| ZINC000038212689 | fda.1508 | -5.101 | -5.301 |
| ZINC000038212689 | fda.1509 | -5.101 | -5.301 |
| ZINC000000002272 | fda.476 | -5.1 | -5.101 |
| ZINC000000005560 | fda.41 | -5.1 | -5.101 |
| ZINC000004475353 | fda.872 | -5.1 | -5.1 |
| ZINC000003786192 | fda.1685 | -5.1 | -5.1 |
| ZINC000084441937 | fda.1908 | -5.099 | -5.138 |
| ZINC000084441937 | fda.1910 | -5.099 | -5.138 |
| ZINC000043100953 | fda.1806 | -5.097 | -5.182 |
| ZINC000000901791 | fda.234 | -5.097 | -5.097 |
| ZINC000000004893 | fda.2004 | -5.097 | -5.101 |
| ZINC000000004893 | fda.2005 | -5.097 | -5.101 |
| ZINC000000004893 | fda.2006 | -5.097 | -5.101 |
| ZINC000000020221 | fda.843 | -5.097 | -5.097 |
| ZINC000004097225 | fda.956 | -5.096 | -5.096 |
| ZINC000003812863 | fda.1649 | -5.096 | -6.092 |
| ZINC000000006157 | fda.814 | -5.095 | -5.133 |
| ZINC000001999487 | fda.1757 | -5.095 | -5.133 |
| ZINC000003830813 | fda.1652 | -5.095 | -5.341 |
| ZINC000003830813 | fda.1654 | -5.095 | -5.341 |
| ZINC000000044027 | fda.1501 | -5.094 | -5.095 |
| ZINC000000000509 | fda.1549 | -5.092 | -5.18 |
| ZINC000000000509 | fda.1550 | -5.092 | -5.18 |
| ZINC000000000509 | fda.1551 | -5.092 | -5.18 |
| ZINC000018279854 | fda.747 | -5.092 | -5.098 |
| ZINC000003079340 | fda.1470 | -5.091 | -5.091 |
| ZINC000100009383 | fda.25 | -5.09 | -5.09 |
| ZINC000004392649 | fda.495 | -5.09 | -5.09 |
| ZINC000001531009 | fda.931 | -5.09 | -5.278 |
| ZINC000000018635 | fda.26 | -5.09 | -5.09 |
| ZINC000001531009 | fda.931 | -5.089 | -5.881 |
| ZINC000003782818 | fda.2058 | -5.087 | -5.666 |
| ZINC000100036924 | fda.1105 | -5.086 | -5.125 |
| ZINC000006627681 | fda.1095 | -5.085 | -5.718 |
| ZINC000001530752 | fda.152 | -5.08 | -5.159 |
| ZINC000001530752 | fda.153 | -5.08 | -5.159 |
| ZINC000001530751 | fda.202 | -5.08 | -5.159 |
| ZINC000001530751 | fda.203 | -5.08 | -5.159 |
| ZINC000013986658 | fda.541 | -5.08 | -7.077 |
| ZINC000006716957 | fda.870 | -5.08 | -5.22 |
| ZINC000006716957 | fda.871 | -5.08 | -5.22 |
| ZINC000001850376 | fda.1777 | -5.077 | -5.077 |
| ZINC000000057533 | fda.985 | -5.076 | -5.084 |
| ZINC000000057532 | fda.1018 | -5.076 | -5.084 |
| ZINC000003629271 | fda.1705 | -5.076 | -5.076 |
| ZINC000004393164 | fda.844 | -5.074 | -5.079 |
| ZINC000011616925 | fda.144 | -5.072 | -5.114 |
| ZINC000001536109 | fda.221 | -5.072 | -5.114 |
| ZINC000004217732 | fda.364 | -5.071 | -5.075 |
| ZINC000000001011 | fda.244 | -5.068 | -5.069 |
| ZINC000000057001 | fda.1070 | -5.068 | -5.068 |
| ZINC000029319828 | fda.1285 | -5.064 | -5.097 |
| ZINC000005733652 | fda.1265 | -5.063 | -5.099 |
| ZINC000005733652 | fda.1268 | -5.063 | -5.099 |
| ZINC000000592419 | fda.120 | -5.063 | -5.063 |
| ZINC000003872055 | fda.1398 | -5.06 | -5.374 |
| ZINC000100003902 | fda.59 | -5.059 | -5.059 |
| ZINC000003201907 | fda.2051 | -5.056 | -5.06 |
| ZINC000001999441 | fda.490 | -5.056 | -5.076 |
| ZINC000005733652 | fda.1266 | -5.056 | -5.092 |
| ZINC000005733652 | fda.1267 | -5.056 | -5.092 |
| ZINC000001187543 | fda.598 | -5.055 | -5.055 |
| ZINC000003812897 | fda.1059 | -5.054 | -5.054 |
| ZINC000003812851 | fda.266 | -5.053 | -5.18 |
| ZINC000013520815 | fda.1338 | -5.051 | -5.051 |
| ZINC000000000905 | fda.1154 | -5.051 | -5.1 |
| ZINC000003830959 | fda.628 | -5.051 | -5.051 |
| ZINC000003830957 | fda.1739 | -5.051 | -5.051 |
| ZINC000003830891 | fda.239 | -5.051 | -5.057 |
| ZINC000035328014 | fda.1555 | -5.05 | -5.459 |
| ZINC000000057278 | fda.989 | -5.05 | -5.05 |
| ZINC000000003911 | fda.1680 | -5.05 | -5.05 |
| ZINC000003782818 | fda.2058 | -5.049 | -5.329 |
| ZINC000008214651 | fda.1131 | -5.047 | -5.047 |
| ZINC000100032379 | fda.1295 | -5.047 | -5.856 |
| ZINC000019632834 | fda.739 | -5.046 | -5.154 |
| ZINC000019632834 | fda.740 | -5.046 | -5.154 |
| ZINC000019632834 | fda.741 | -5.046 | -5.154 |
| ZINC000013648755 | fda.418 | -5.046 | -5.158 |
| ZINC000008552123 | fda.1567 | -5.045 | -5.095 |
| ZINC000008552123 | fda.1568 | -5.045 | -5.095 |
| ZINC000003875393 | fda.1719 | -5.045 | -5.095 |
| ZINC000013537284 | fda.748 | -5.045 | -5.948 |
| ZINC000003782818 | fda.2059 | -5.044 | -5.324 |
| ZINC000000896484 | fda.298 | -5.043 | -5.066 |
| ZINC000000896484 | fda.299 | -5.043 | -5.066 |
| ZINC000034636383 | fda.2083 | -5.043 | -5.05 |
| ZINC000001999441 | fda.489 | -5.042 | -5.062 |
| ZINC000003807917 | fda.264 | -5.041 | -5.041 |
| ZINC000003872277 | fda.1738 | -5.041 | -5.049 |
| ZINC000000020228 | fda.325 | -5.038 | -5.275 |
| ZINC000000057206 | fda.987 | -5.038 | -5.275 |
| ZINC000253498282 | fda.189 | -5.038 | -5.038 |
| ZINC000003831474 | fda.1973 | -5.037 | -5.037 |
| ZINC000001895505 | fda.968 | -5.037 | -5.047 |
| ZINC000040430143 | fda.349 | -5.037 | -5.037 |
| ZINC000003830569 | fda.119 | -5.036 | -5.036 |
| ZINC000003801919 | fda.1467 | -5.035 | -5.234 |
| ZINC000000002647 | fda.939 | -5.035 | -5.036 |
| ZINC000000113398 | fda.1776 | -5.035 | -5.036 |
| ZINC000003973334 | fda.652 | -5.035 | -5.035 |
| ZINC000003875392 | fda.562 | -5.035 | -5.084 |
| ZINC000000000751 | fda.1414 | -5.035 | -5.084 |
| ZINC000000596881 | fda.311 | -5.034 | -5.241 |
| ZINC000000643055 | fda.1500 | -5.034 | -5.241 |
| ZINC000001530555 | fda.123 | -5.033 | -5.033 |
| ZINC000013648755 | fda.417 | -5.032 | -5.144 |
| ZINC000003830453 | fda.1695 | -5.032 | -5.032 |
| ZINC000028108825 | fda.89 | -5.031 | -5.123 |
| ZINC000028108825 | fda.90 | -5.031 | -5.123 |
| ZINC000028108825 | fda.91 | -5.031 | -5.123 |
| ZINC000100022637 | fda.1292 | -5.03 | -5.962 |
| ZINC000100022637 | fda.1293 | -5.03 | -5.962 |
| ZINC000100016058 | fda.2078 | -5.03 | -5.962 |
| ZINC000100016058 | fda.2079 | -5.03 | -5.962 |
| ZINC000000607939 | fda.50 | -5.03 | -5.03 |
| ZINC000100001918 | fda.1319 | -5.03 | -5.03 |
| ZINC000000897385 | fda.1426 | -5.029 | -5.288 |
| ZINC000000608101 | fda.1228 | -5.029 | -5.288 |
| ZINC000000968328 | fda.403 | -5.029 | -5.47 |
| ZINC000000968330 | fda.1173 | -5.029 | -5.47 |
| ZINC000006733300 | fda.1234 | -5.027 | -5.029 |
| ZINC000009212428 | fda.270 | -5.025 | -5.025 |
| ZINC000009212427 | fda.1351 | -5.025 | -5.025 |
| ZINC000004213946 | fda.950 | -5.024 | -5.044 |
| ZINC000005844792 | fda.1310 | -5.024 | -5.044 |
| ZINC000000901736 | fda.609 | -5.024 | -5.024 |
| ZINC000000895457 | fda.614 | -5.024 | -5.024 |
| ZINC000000001984 | fda.1944 | -5.023 | -5.023 |
| ZINC000000156395 | fda.1256 | -5.023 | -5.023 |
| ZINC000001530741 | fda.1117 | -5.021 | -5.022 |
| ZINC000000968328 | fda.403 | -5.02 | -5.419 |
| ZINC000000968330 | fda.1173 | -5.02 | -5.419 |
| ZINC000003831475 | fda.1009 | -5.02 | -5.02 |
| ZINC000003831477 | fda.1974 | -5.02 | -5.02 |
| ZINC000000156792 | fda.905 | -5.019 | -5.021 |
| ZINC000000968255 | fda.1779 | -5.019 | -5.021 |
| ZINC000003806413 | fda.377 | -5.018 | -5.018 |
| ZINC000100074252 | fda.1300 | -5.017 | -5.02 |
| ZINC000003819138 | fda.1686 | -5.013 | -5.013 |
| ZINC000003814422 | fda.1878 | -5.011 | -5.011 |
| ZINC000004097416 | fda.1618 | -5.009 | -5.009 |
| ZINC000014263142 | fda.721 | -5.007 | -5.007 |
| ZINC000009212428 | fda.271 | -5.007 | -5.007 |
| ZINC000009212427 | fda.1352 | -5.007 | -5.007 |
| ZINC000085537014 | fda.906 | -5.006 | -5.318 |
| ZINC000085537014 | fda.907 | -5.006 | -5.318 |
| ZINC000000001735 | fda.1033 | -5.006 | -5.006 |
| ZINC000004099200 | fda.835 | -5.005 | -5.314 |
| ZINC000004676424 | fda.880 | -5.005 | -5.314 |
| ZINC000028232750 | fda.174 | -5.005 | -5.006 |
| ZINC000003830813 | fda.1652 | -5.005 | -5.251 |
| ZINC000003830813 | fda.1654 | -5.005 | -5.251 |
| ZINC000003927870 | fda.1493 | -5.005 | -6.154 |
| ZINC000013537284 | fda.748 | -5.003 | -5.299 |
| ZINC000001530760 | fda.808 | -5.003 | -5.006 |
| ZINC000001530759 | fda.894 | -5.003 | -5.006 |
| ZINC000004213946 | fda.951 | -5.003 | -5.022 |
| ZINC000005844792 | fda.1311 | -5.003 | -5.022 |
| ZINC000000897385 | fda.1427 | -5.003 | -5.262 |
| ZINC000000608101 | fda.1229 | -5.003 | -5.262 |
| ZINC000000601317 | fda.2043 | -5.001 | -5.017 |
| ZINC000003823492 | fda.1642 | -5 | -5 |
| ZINC000003813047 | fda.2066 | -5 | -5 |
| ZINC000003792417 | fda.557 | -4.998 | -5.013 |
| ZINC000003812851 | fda.265 | -4.997 | -5.124 |
| ZINC000009224016 | fda.124 | -4.996 | -5.015 |
| ZINC000009224016 | fda.125 | -4.996 | -5.015 |
| ZINC000001530977 | fda.890 | -4.995 | -5.074 |
| ZINC000001530977 | fda.891 | -4.995 | -5.074 |
| ZINC000001530977 | fda.892 | -4.995 | -5.074 |
| ZINC000001531009 | fda.932 | -4.994 | -5.183 |
| ZINC000000596881 | fda.310 | -4.993 | -5.201 |
| ZINC000000643055 | fda.1499 | -4.993 | -5.201 |
| ZINC000098023177 | fda.1931 | -4.993 | -5.001 |
| ZINC000098023177 | fda.1932 | -4.993 | -5.001 |
| ZINC000004474443 | fda.510 | -4.993 | -5.275 |
| ZINC000053084692 | fda.75 | -4.993 | -5.771 |
| ZINC000049643479 | fda.1184 | -4.991 | -4.993 |
| ZINC000000002212 | fda.629 | -4.991 | -4.992 |
| ZINC000003813047 | fda.2066 | -4.989 | -4.989 |
| ZINC000003964126 | fda.651 | -4.987 | -4.987 |
| ZINC000100009280 | fda.1243 | -4.986 | -4.988 |
| ZINC000003792417 | fda.557 | -4.985 | -5 |
| ZINC000003985982 | fda.1278 | -4.985 | -4.985 |
| ZINC000053084692 | fda.74 | -4.985 | -5.763 |
| ZINC000000020220 | fda.370 | -4.983 | -5.083 |
| ZINC000253476027 | fda.497 | -4.983 | -4.983 |
| ZINC000100015775 | fda.1282 | -4.983 | -4.983 |
| ZINC000000020783 | fda.588 | -4.983 | -4.983 |
| ZINC000000509440 | fda.1477 | -4.981 | -4.981 |
| ZINC000000000575 | fda.1831 | -4.981 | -4.981 |
| ZINC000004097308 | fda.899 | -4.979 | -4.979 |
| ZINC000000156395 | fda.1256 | -4.978 | -4.978 |
| ZINC000000896740 | fda.1498 | -4.977 | -5.184 |
| ZINC000000607971 | fda.1507 | -4.977 | -5.184 |
| ZINC000000009689 | fda.1298 | -4.977 | -4.977 |
| ZINC000019632917 | fda.709 | -4.976 | -7.109 |
| ZINC000003201907 | fda.2050 | -4.974 | -4.978 |
| ZINC000003776970 | fda.1691 | -4.973 | -5.255 |
| ZINC000028467879 | fda.1903 | -4.972 | -4.972 |
| ZINC000028467879 | fda.1904 | -4.972 | -4.972 |
| ZINC000012360535 | fda.99 | -4.972 | -4.972 |
| ZINC000003795098 | fda.625 | -4.971 | -4.971 |
| ZINC000006733300 | fda.1234 | -4.971 | -4.972 |
| ZINC000000004949 | fda.521 | -4.97 | -4.97 |
| ZINC000003808779 | fda.974 | -4.968 | -4.997 |
| ZINC000000007601 | fda.1591 | -4.967 | -4.967 |
| ZINC000000020230 | fda.1608 | -4.967 | -4.967 |
| ZINC000003914808 | fda.606 | -4.966 | -4.971 |
| ZINC000003806721 | fda.1714 | -4.964 | -5.01 |
| ZINC000003977981 | fda.1013 | -4.964 | -4.964 |
| ZINC000022116608 | fda.646 | -4.964 | -4.99 |
| ZINC000022116612 | fda.1166 | -4.964 | -4.99 |
| ZINC000003871541 | fda.1736 | -4.963 | -4.963 |
| ZINC000003925861 | fda.1778 | -4.963 | -4.974 |
| ZINC000000968310 | fda.331 | -4.961 | -5.049 |
| ZINC000000968310 | fda.332 | -4.961 | -5.049 |
| ZINC000000968310 | fda.333 | -4.961 | -5.049 |
| ZINC000001530599 | fda.919 | -4.96 | -4.96 |
| ZINC000003979899 | fda.2070 | -4.959 | -4.959 |
| ZINC000003922770 | fda.554 | -4.959 | -5.381 |
| ZINC000001530283 | fda.1393 | -4.956 | -4.956 |
| ZINC000001481833 | fda.793 | -4.955 | -4.955 |
| ZINC000006661227 | fda.1197 | -4.955 | -5.411 |
| ZINC000006661227 | fda.1198 | -4.955 | -5.411 |
| ZINC000011677376 | fda.2018 | -4.954 | -4.958 |
| ZINC000011677376 | fda.2019 | -4.954 | -4.958 |
| ZINC000011677376 | fda.2020 | -4.954 | -4.958 |
| ZINC000001530810 | fda.1097 | -4.953 | -4.953 |
| ZINC000001571009 | fda.1957 | -4.95 | -4.95 |
| ZINC000001531009 | fda.930 | -4.949 | -5.137 |
| ZINC000003914809 | fda.1391 | -4.948 | -4.952 |
| ZINC000000006157 | fda.814 | -4.947 | -4.985 |
| ZINC000001999487 | fda.1757 | -4.947 | -4.985 |
| ZINC000013537284 | fda.749 | -4.947 | -5.242 |
| ZINC000013550868 | fda.1112 | -4.946 | -4.947 |
| ZINC000000897288 | fda.1745 | -4.945 | -4.945 |
| ZINC000004474405 | fda.322 | -4.942 | -4.942 |
| ZINC000004097309 | fda.900 | -4.94 | -4.941 |
| ZINC000003920355 | fda.1394 | -4.94 | -4.941 |
| ZINC000034051848 | fda.1224 | -4.939 | -4.945 |
| ZINC000001530710 | fda.787 | -4.936 | -4.936 |
| ZINC000003830449 | fda.1699 | -4.936 | -4.936 |
| ZINC000027990463 | fda.1320 | -4.936 | -4.939 |
| ZINC000000120319 | fda.1515 | -4.935 | -5.804 |
| ZINC000000120319 | fda.1516 | -4.935 | -5.804 |
| ZINC000001999441 | fda.490 | -4.935 | -4.955 |
| ZINC000000001341 | fda.1558 | -4.933 | -5.004 |
| ZINC000000897322 | fda.1434 | -4.933 | -4.933 |
| ZINC000000000471 | fda.1484 | -4.933 | -4.933 |
| ZINC000003830218 | fda.281 | -4.932 | -5.16 |
| ZINC000013682481 | fda.752 | -4.932 | -5.069 |
| ZINC000013682481 | fda.753 | -4.932 | -5.069 |
| ZINC000038212689 | fda.1508 | -4.932 | -5.674 |
| ZINC000038212689 | fda.1509 | -4.932 | -5.674 |
| ZINC000003779042 | fda.2054 | -4.931 | -4.931 |
| ZINC000003978006 | fda.1890 | -4.929 | -4.929 |
| ZINC000004474405 | fda.322 | -4.927 | -4.927 |
| ZINC000001611274 | fda.585 | -4.926 | -5.194 |
| ZINC000011680067 | fda.1619 | -4.925 | -4.925 |
| ZINC000004213946 | fda.951 | -4.925 | -4.945 |
| ZINC000005844792 | fda.1311 | -4.925 | -4.945 |
| ZINC000000001341 | fda.1559 | -4.922 | -4.993 |
| ZINC000000607986 | fda.63 | -4.92 | -4.94 |
| ZINC000003830218 | fda.282 | -4.919 | -5.147 |
| ZINC000003797541 | fda.1681 | -4.918 | -4.939 |
| ZINC000084441937 | fda.1908 | -4.917 | -4.956 |
| ZINC000084441937 | fda.1910 | -4.917 | -4.956 |
| ZINC000004099200 | fda.835 | -4.916 | -5.225 |
| ZINC000004676424 | fda.880 | -4.916 | -5.225 |
| ZINC000003830347 | fda.196 | -4.915 | -5.02 |
| ZINC000003830347 | fda.197 | -4.915 | -5.02 |
| ZINC000000002299 | fda.78 | -4.914 | -4.918 |
| ZINC000000002299 | fda.79 | -4.914 | -4.918 |
| ZINC000000002299 | fda.80 | -4.914 | -4.918 |
| ZINC000019632917 | fda.709 | -4.913 | -4.929 |
| ZINC000001996117 | fda.981 | -4.911 | -4.913 |
| ZINC000000968328 | fda.402 | -4.909 | -6.286 |
| ZINC000000968330 | fda.1172 | -4.909 | -6.286 |
| ZINC000000518554 | fda.1536 | -4.908 | -4.909 |
| ZINC000034051848 | fda.1223 | -4.906 | -4.912 |
| ZINC000000057464 | fda.1075 | -4.905 | -5.004 |
| ZINC000100005670 | fda.1958 | -4.904 | -4.935 |
| ZINC000003914809 | fda.1391 | -4.904 | -4.908 |
| ZINC000013986658 | fda.542 | -4.903 | -6.901 |
| ZINC000000001850 | fda.1810 | -4.902 | -4.902 |
| ZINC000004258316 | fda.180 | -4.901 | -6.504 |
| ZINC000003831404 | fda.30 | -4.9 | -4.915 |
| ZINC000003831404 | fda.31 | -4.9 | -4.915 |
| ZINC000001530922 | fda.1120 | -4.899 | -6.45 |
| ZINC000049783788 | fda.2086 | -4.899 | -4.9 |
| ZINC000022116608 | fda.646 | -4.899 | -4.925 |
| ZINC000022116612 | fda.1166 | -4.899 | -4.925 |
| ZINC000003794711 | fda.407 | -4.899 | -4.918 |
| ZINC000003794711 | fda.408 | -4.899 | -4.918 |
| ZINC000003794711 | fda.409 | -4.899 | -4.918 |
| ZINC000118912393 | fda.1248 | -4.899 | -4.899 |
| ZINC000000643138 | fda.1028 | -4.898 | -5.222 |
| ZINC000000643143 | fda.253 | -4.898 | -5.222 |
| ZINC000009212428 | fda.269 | -4.895 | -4.895 |
| ZINC000009212427 | fda.1350 | -4.895 | -4.895 |
| ZINC000070466416 | fda.536 | -4.893 | -4.938 |
| ZINC000001530639 | fda.359 | -4.893 | -4.895 |
| ZINC000085540215 | fda.1787 | -4.893 | -4.893 |
| ZINC000008214418 | fda.1992 | -4.893 | -4.893 |
| ZINC000000000507 | fda.1563 | -4.893 | -5.001 |
| ZINC000000403079 | fda.1677 | -4.893 | -5.001 |
| ZINC000001530975 | fda.888 | -4.891 | -5.024 |
| ZINC000001530975 | fda.889 | -4.891 | -5.024 |
| ZINC000003795098 | fda.625 | -4.889 | -4.889 |
| ZINC000003801163 | fda.1709 | -4.887 | -5.041 |
| ZINC000003979899 | fda.2070 | -4.885 | -4.885 |
| ZINC000000002055 | fda.1046 | -4.885 | -4.923 |
| ZINC000000002055 | fda.1047 | -4.885 | -4.923 |
| ZINC000012503068 | fda.574 | -4.884 | -4.884 |
| ZINC000000001408 | fda.1560 | -4.884 | -4.894 |
| ZINC000013585233 | fda.272 | -4.883 | -6.91 |
| ZINC000013585233 | fda.273 | -4.883 | -6.91 |
| ZINC000013585233 | fda.274 | -4.883 | -6.91 |
| ZINC000019168887 | fda.1590 | -4.883 | -4.883 |
| ZINC000000020253 | fda.842 | -4.879 | -4.88 |
| ZINC000000057255 | fda.1766 | -4.879 | -4.88 |
| ZINC000003830215 | fda.1246 | -4.877 | -5.07 |
| ZINC000001851149 | fda.1784 | -4.877 | -5.636 |
| ZINC000001851149 | fda.1785 | -4.877 | -5.636 |
| ZINC000095616601 | fda.18 | -4.876 | -4.883 |
| ZINC000001531009 | fda.930 | -4.876 | -5.668 |
| ZINC000002008866 | fda.654 | -4.874 | -4.874 |
| ZINC000022116608 | fda.647 | -4.874 | -4.9 |
| ZINC000022116612 | fda.1167 | -4.874 | -4.9 |
| ZINC000084758235 | fda.828 | -4.873 | -4.9 |
| ZINC000000039089 | fda.1157 | -4.871 | -4.882 |
| ZINC000000968326 | fda.114 | -4.87 | -5.352 |
| ZINC000000968326 | fda.115 | -4.87 | -5.352 |
| ZINC000000968327 | fda.1747 | -4.87 | -5.352 |
| ZINC000000968327 | fda.1748 | -4.87 | -5.352 |
| ZINC000014879972 | fda.731 | -4.87 | -4.953 |
| ZINC000252679615 | fda.341 | -4.869 | -4.942 |
| ZINC000252679615 | fda.342 | -4.869 | -4.942 |
| ZINC000002568036 | fda.53 | -4.869 | -4.942 |
| ZINC000002568036 | fda.54 | -4.869 | -4.942 |
| ZINC000007997966 | fda.1990 | -4.869 | -4.942 |
| ZINC000007997966 | fda.1991 | -4.869 | -4.942 |
| ZINC000008577218 | fda.251 | -4.869 | -5.149 |
| ZINC000003986735 | fda.992 | -4.869 | -5.291 |
| ZINC000013911941 | fda.2076 | -4.868 | -4.868 |
| ZINC000004212809 | fda.84 | -4.868 | -4.868 |
| ZINC000000537795 | fda.847 | -4.866 | -4.866 |
| ZINC000000049154 | fda.644 | -4.864 | -5.579 |
| ZINC000004474443 | fda.509 | -4.864 | -5.147 |
| ZINC000000121541 | fda.1517 | -4.864 | -5.587 |
| ZINC000013520815 | fda.1338 | -4.859 | -4.859 |
| ZINC000100018854 | fda.500 | -4.859 | -4.86 |
| ZINC000001542930 | fda.327 | -4.859 | -4.859 |
| ZINC000253498282 | fda.189 | -4.859 | -4.859 |
| ZINC000003818808 | fda.491 | -4.859 | -4.87 |
| ZINC000003201907 | fda.2050 | -4.859 | -4.863 |
| ZINC000004099200 | fda.835 | -4.858 | -6.876 |
| ZINC000004676424 | fda.880 | -4.858 | -6.876 |
| ZINC000001534965 | fda.601 | -4.858 | -4.867 |
| ZINC000003989268 | fda.2071 | -4.857 | -4.858 |
| ZINC000096006023 | fda.2098 | -4.857 | -4.858 |
| ZINC000068202099 | fda.214 | -4.856 | -4.856 |
| ZINC000000000347 | fda.1938 | -4.855 | -4.856 |
| ZINC000012360535 | fda.101 | -4.853 | -5.178 |
| ZINC000000003642 | fda.1156 | -4.852 | -4.853 |
| ZINC000000057313 | fda.1274 | -4.852 | -4.853 |
| ZINC000003861599 | fda.1882 | -4.852 | -4.852 |
| ZINC000001530716 | fda.790 | -4.852 | -4.856 |
| ZINC000000000353 | fda.705 | -4.847 | -4.847 |
| ZINC000000394284 | fda.1181 | -4.847 | -4.847 |
| ZINC000087515509 | fda.1287 | -4.847 | -5.775 |
| ZINC000000119717 | fda.436 | -4.845 | -4.845 |
| ZINC000072316335 | fda.1929 | -4.845 | -4.914 |
| ZINC000029571072 | fda.2032 | -4.845 | -5.277 |
| ZINC000049637509 | fda.2041 | -4.845 | -5.277 |
| ZINC000001280665 | fda.413 | -4.843 | -4.873 |
| ZINC000084589076 | fda.1911 | -4.843 | -4.864 |
| ZINC000100006264 | fda.1917 | -4.843 | -4.864 |
| ZINC000002539827 | fda.1383 | -4.843 | -6.87 |
| ZINC000002539827 | fda.1384 | -4.843 | -6.87 |
| ZINC000002539827 | fda.1385 | -4.843 | -6.87 |
| ZINC000000895154 | fda.1406 | -4.843 | -6.87 |
| ZINC000000895154 | fda.1407 | -4.843 | -6.87 |
| ZINC000000895154 | fda.1408 | -4.843 | -6.87 |
| ZINC000100007011 | fda.1377 | -4.842 | -4.842 |
| ZINC000100071256 | fda.1918 | -4.842 | -4.842 |
| ZINC000000968326 | fda.115 | -4.841 | -5.281 |
| ZINC000000968327 | fda.1748 | -4.841 | -5.281 |
| ZINC000001489478 | fda.457 | -4.838 | -4.841 |
| ZINC000003812988 | fda.2055 | -4.836 | -4.847 |
| ZINC000003812988 | fda.2056 | -4.836 | -4.847 |
| ZINC000003973334 | fda.652 | -4.836 | -4.836 |
| ZINC000003813061 | fda.1638 | -4.833 | -4.833 |
| ZINC000060325170 | fda.1200 | -4.831 | -4.834 |
| ZINC000116473771 | fda.538 | -4.831 | -4.831 |
| ZINC000035653007 | fda.1545 | -4.831 | -5.057 |
| ZINC000035653009 | fda.1546 | -4.831 | -5.057 |
| ZINC000087515509 | fda.1287 | -4.83 | -6.408 |
| ZINC000000000565 | fda.686 | -4.829 | -4.843 |
| ZINC000000000565 | fda.687 | -4.829 | -4.843 |
| ZINC000000155269 | fda.1832 | -4.829 | -4.843 |
| ZINC000000155269 | fda.1833 | -4.829 | -4.843 |
| ZINC000003978005 | fda.1235 | -4.828 | -6.555 |
| ZINC000003978005 | fda.1236 | -4.828 | -6.555 |
| ZINC000003978005 | fda.1237 | -4.828 | -6.555 |
| ZINC000001530625 | fda.1796 | -4.827 | -4.827 |
| ZINC000000000905 | fda.1155 | -4.826 | -4.826 |
| ZINC000012360535 | fda.100 | -4.826 | -5.151 |
| ZINC000001493878 | fda.1526 | -4.824 | -4.836 |
| ZINC000013597823 | fda.750 | -4.824 | -4.906 |
| ZINC000000002005 | fda.288 | -4.823 | -4.823 |
| ZINC000003875980 | fda.963 | -4.822 | -4.822 |
| ZINC000003803652 | fda.830 | -4.822 | -5.328 |
| ZINC000100006770 | fda.278 | -4.821 | -4.821 |
| ZINC000000002647 | fda.939 | -4.82 | -4.821 |
| ZINC000000113398 | fda.1776 | -4.82 | -4.821 |
| ZINC000003817234 | fda.2100 | -4.819 | -4.819 |
| ZINC000003874950 | fda.229 | -4.819 | -6.35 |
| ZINC000003874950 | fda.230 | -4.819 | -6.35 |
| ZINC000000968328 | fda.402 | -4.819 | -5.343 |
| ZINC000000968330 | fda.1172 | -4.819 | -5.343 |
| ZINC000022116608 | fda.647 | -4.819 | -4.845 |
| ZINC000022116612 | fda.1167 | -4.819 | -4.845 |
| ZINC000084843283 | fda.1809 | -4.817 | -4.851 |
| ZINC000000000693 | fda.1182 | -4.816 | -4.816 |
| ZINC000000020241 | fda.1543 | -4.813 | -4.814 |
| ZINC000003816287 | fda.67 | -4.812 | -4.815 |
| ZINC000011616882 | fda.1132 | -4.812 | -4.815 |
| ZINC000068202099 | fda.214 | -4.812 | -4.812 |
| ZINC000003812974 | fda.832 | -4.811 | -4.812 |
| ZINC000003831165 | fda.1702 | -4.811 | -4.812 |
| ZINC000003930376 | fda.1754 | -4.811 | -4.811 |
| ZINC000000001899 | fda.292 | -4.81 | -4.81 |
| ZINC000000000693 | fda.1182 | -4.809 | -4.809 |
| ZINC000000004319 | fda.317 | -4.809 | -6.747 |
| ZINC000000403010 | fda.1457 | -4.809 | -6.747 |
| ZINC000003830713 | fda.1389 | -4.808 | -4.811 |
| ZINC000030691763 | fda.1339 | -4.808 | -4.808 |
| ZINC000030691760 | fda.1342 | -4.808 | -4.808 |
| ZINC000001530618 | fda.929 | -4.808 | -4.813 |
| ZINC000001530617 | fda.1138 | -4.808 | -4.813 |
| ZINC000011681534 | fda.2022 | -4.807 | -4.827 |
| ZINC000000001958 | fda.587 | -4.807 | -4.807 |
| ZINC000000968233 | fda.611 | -4.804 | -4.804 |
| ZINC000016052277 | fda.1897 | -4.799 | -4.832 |
| ZINC000003812869 | fda.2047 | -4.799 | -5.432 |
| ZINC000011681534 | fda.2021 | -4.797 | -4.816 |
| ZINC000002020233 | fda.406 | -4.796 | -4.796 |
| ZINC000003814422 | fda.1878 | -4.796 | -4.796 |
| ZINC000003813042 | fda.1814 | -4.796 | -5.142 |
| ZINC000148723177 | fda.338 | -4.796 | -4.809 |
| ZINC000148723177 | fda.339 | -4.796 | -4.809 |
| ZINC000000075126 | fda.393 | -4.794 | -5.777 |
| ZINC000000075126 | fda.394 | -4.794 | -5.777 |
| ZINC000000004448 | fda.107 | -4.794 | -5.777 |
| ZINC000000004448 | fda.108 | -4.794 | -5.777 |
| ZINC000004212809 | fda.84 | -4.794 | -4.795 |
| ZINC000003938684 | fda.1676 | -4.794 | -4.794 |
| ZINC000000056646 | fda.986 | -4.791 | -4.793 |
| ZINC000000056645 | fda.1270 | -4.791 | -4.793 |
| ZINC000004340269 | fda.998 | -4.79 | -4.791 |
| ZINC000000601250 | fda.1504 | -4.79 | -5.122 |
| ZINC000013512456 | fda.344 | -4.79 | -4.795 |
| ZINC000000001505 | fda.1439 | -4.789 | -4.789 |
| ZINC000000002055 | fda.1046 | -4.789 | -6.436 |
| ZINC000000002055 | fda.1047 | -4.789 | -6.436 |
| ZINC000013585233 | fda.272 | -4.786 | -6.813 |
| ZINC000013585233 | fda.273 | -4.786 | -6.813 |
| ZINC000013585233 | fda.274 | -4.786 | -6.813 |
| ZINC000000968310 | fda.331 | -4.786 | -4.874 |
| ZINC000000968310 | fda.332 | -4.786 | -4.874 |
| ZINC000000968310 | fda.333 | -4.786 | -4.874 |
| ZINC000013512456 | fda.345 | -4.784 | -4.789 |
| ZINC000000895081 | fda.1043 | -4.784 | -4.784 |
| ZINC000011679756 | fda.468 | -4.783 | -5.647 |
| ZINC000000527386 | fda.999 | -4.783 | -4.784 |
| ZINC000038945666 | fda.2039 | -4.783 | -4.783 |
| ZINC000000601317 | fda.2043 | -4.782 | -4.798 |
| ZINC000003872605 | fda.1731 | -4.782 | -4.785 |
| ZINC000008101109 | fda.424 | -4.781 | -4.787 |
| ZINC000003875560 | fda.1392 | -4.78 | -4.78 |
| ZINC000019632917 | fda.710 | -4.779 | -6.644 |
| ZINC000000001795 | fda.1423 | -4.778 | -4.788 |
| ZINC000004640636 | fda.874 | -4.778 | -4.816 |
| ZINC000019796018 | fda.742 | -4.776 | -5.057 |
| ZINC000019796018 | fda.743 | -4.776 | -5.057 |
| ZINC000019796018 | fda.744 | -4.776 | -5.057 |
| ZINC000000007295 | fda.816 | -4.776 | -4.776 |
| ZINC000000006481 | fda.467 | -4.774 | -4.774 |
| ZINC000003806721 | fda.1715 | -4.773 | -4.819 |
| ZINC000019702309 | fda.724 | -4.772 | -5.609 |
| ZINC000022010379 | fda.637 | -4.771 | -4.985 |
| ZINC000022010375 | fda.2030 | -4.771 | -4.985 |
| ZINC000000000856 | fda.1153 | -4.769 | -4.782 |
| ZINC000000897258 | fda.2012 | -4.769 | -4.782 |
| ZINC000004640636 | fda.875 | -4.769 | -4.807 |
| ZINC000000000740 | fda.1413 | -4.769 | -4.769 |
| ZINC000000896634 | fda.1079 | -4.769 | -4.769 |
| ZINC000000020228 | fda.326 | -4.768 | -5.005 |
| ZINC000000057206 | fda.988 | -4.768 | -5.005 |
| ZINC000003941496 | fda.420 | -4.767 | -4.775 |
| ZINC000100016084 | fda.515 | -4.767 | -4.767 |
| ZINC000003860453 | fda.960 | -4.765 | -5.476 |
| ZINC000000105196 | fda.610 | -4.765 | -4.765 |
| ZINC000019702309 | fda.725 | -4.763 | -5.6 |
| ZINC000001843099 | fda.1782 | -4.762 | -4.77 |
| ZINC000000002299 | fda.78 | -4.761 | -4.765 |
| ZINC000000002299 | fda.79 | -4.761 | -4.765 |
| ZINC000000002299 | fda.80 | -4.761 | -4.765 |
| ZINC000053084692 | fda.74 | -4.761 | -4.947 |
| ZINC000022059926 | fda.645 | -4.76 | -4.801 |
| ZINC000022059930 | fda.1165 | -4.76 | -4.801 |
| ZINC000003813010 | fda.104 | -4.76 | -4.789 |
| ZINC000000537822 | fda.437 | -4.757 | -4.77 |
| ZINC000000537822 | fda.438 | -4.757 | -4.77 |
| ZINC000013129998 | fda.1661 | -4.756 | -4.771 |
| ZINC000000057534 | fda.372 | -4.755 | -4.755 |
| ZINC000118912450 | fda.376 | -4.753 | -4.753 |
| ZINC000001529323 | fda.16 | -4.753 | -4.818 |
| ZINC000003823475 | fda.558 | -4.752 | -4.754 |
| ZINC000003823475 | fda.559 | -4.752 | -4.754 |
| ZINC000003823475 | fda.560 | -4.752 | -4.754 |
| ZINC000100009278 | fda.1242 | -4.75 | -4.753 |
| ZINC000001548097 | fda.1857 | -4.749 | -4.762 |
| ZINC000001548097 | fda.1858 | -4.749 | -4.762 |
| ZINC000000643138 | fda.1027 | -4.748 | -5.297 |
| ZINC000000643138 | fda.1028 | -4.748 | -5.297 |
| ZINC000000643143 | fda.252 | -4.748 | -5.297 |
| ZINC000000643143 | fda.253 | -4.748 | -5.297 |
| ZINC000004099200 | fda.836 | -4.748 | -5.05 |
| ZINC000004676424 | fda.881 | -4.748 | -5.05 |
| ZINC000000120286 | fda.6 | -4.746 | -5.299 |
| ZINC000000601229 | fda.1610 | -4.746 | -4.747 |
| ZINC000000601229 | fda.1611 | -4.746 | -4.747 |
| ZINC000000601229 | fda.1612 | -4.746 | -4.747 |
| ZINC000019594599 | fda.773 | -4.746 | -4.92 |
| ZINC000019594599 | fda.774 | -4.746 | -4.92 |
| ZINC000019594599 | fda.775 | -4.746 | -4.92 |
| ZINC000100013500 | fda.92 | -4.745 | -4.747 |
| ZINC000003842753 | fda.1734 | -4.745 | -4.829 |
| ZINC000000000373 | fda.504 | -4.744 | -4.744 |
| ZINC000001690604 | fda.66 | -4.744 | -4.744 |
| ZINC000001542930 | fda.327 | -4.744 | -4.744 |
| ZINC000019168887 | fda.1590 | -4.74 | -4.74 |
| ZINC000003816514 | fda.1697 | -4.74 | -5.956 |
| ZINC000003830405 | fda.1880 | -4.74 | -4.74 |
| ZINC000004097467 | fda.1987 | -4.738 | -4.738 |
| ZINC000035801098 | fda.1547 | -4.738 | -4.78 |
| ZINC000003816514 | fda.1698 | -4.735 | -5.95 |
| ZINC000003976838 | fda.1761 | -4.734 | -4.917 |
| ZINC000003976838 | fda.1762 | -4.734 | -4.917 |
| ZINC000003812851 | fda.265 | -4.734 | -5.711 |
| ZINC000008552123 | fda.1569 | -4.734 | -4.783 |
| ZINC000003875393 | fda.1718 | -4.734 | -4.783 |
| ZINC000003875393 | fda.1720 | -4.734 | -4.783 |
| ZINC000003782599 | fda.392 | -4.734 | -4.734 |
| ZINC000012503291 | fda.137 | -4.733 | -4.733 |
| ZINC000084400879 | fda.302 | -4.732 | -5.425 |
| ZINC000084400879 | fda.303 | -4.732 | -5.425 |
| ZINC000084400879 | fda.304 | -4.732 | -5.425 |
| ZINC000019796087 | fda.305 | -4.732 | -5.425 |
| ZINC000019796087 | fda.306 | -4.732 | -5.425 |
| ZINC000019796087 | fda.307 | -4.732 | -5.425 |
| ZINC000003830215 | fda.1245 | -4.732 | -4.924 |
| ZINC000001542392 | fda.142 | -4.731 | -4.731 |
| ZINC000000057001 | fda.1070 | -4.729 | -4.729 |
| ZINC000087515509 | fda.1287 | -4.727 | -5.599 |
| ZINC000003831475 | fda.1009 | -4.725 | -4.725 |
| ZINC000003831477 | fda.1974 | -4.725 | -4.725 |
| ZINC000001530974 | fda.689 | -4.725 | -5.169 |
| ZINC000001530974 | fda.690 | -4.725 | -5.169 |
| ZINC000001530973 | fda.883 | -4.725 | -5.169 |
| ZINC000001530973 | fda.884 | -4.725 | -5.169 |
| ZINC000000004319 | fda.317 | -4.724 | -4.752 |
| ZINC000000004319 | fda.318 | -4.724 | -4.752 |
| ZINC000000403010 | fda.1457 | -4.724 | -4.752 |
| ZINC000000403010 | fda.1458 | -4.724 | -4.752 |
| ZINC000000089763 | fda.453 | -4.723 | -4.732 |
| ZINC000000089763 | fda.454 | -4.723 | -4.732 |
| ZINC000000596881 | fda.310 | -4.723 | -4.93 |
| ZINC000000643055 | fda.1499 | -4.723 | -4.93 |
| ZINC000008577218 | fda.251 | -4.723 | -5.606 |
| ZINC000000015515 | fda.1604 | -4.722 | -4.726 |
| ZINC000014210455 | fda.736 | -4.72 | -4.72 |
| ZINC000003801919 | fda.1467 | -4.72 | -4.919 |
| ZINC000003927200 | fda.97 | -4.719 | -4.719 |
| ZINC000003812974 | fda.832 | -4.717 | -4.718 |
| ZINC000003831165 | fda.1702 | -4.717 | -4.718 |
| ZINC000029571072 | fda.2032 | -4.716 | -5.148 |
| ZINC000049637509 | fda.2041 | -4.716 | -5.148 |
| ZINC000001550477 | fda.328 | -4.716 | -4.728 |
| ZINC000003820029 | fda.143 | -4.715 | -4.715 |
| ZINC000035328014 | fda.1554 | -4.714 | -4.714 |
| ZINC000051133897 | fda.13 | -4.713 | -4.718 |
| ZINC000000897002 | fda.1082 | -4.713 | -4.767 |
| ZINC000000897002 | fda.1083 | -4.713 | -4.767 |
| ZINC000000897002 | fda.1084 | -4.713 | -4.767 |
| ZINC000003812869 | fda.2048 | -4.71 | -4.959 |
| ZINC000001542392 | fda.142 | -4.709 | -4.709 |
| ZINC000004097426 | fda.957 | -4.709 | -4.79 |
| ZINC000004097426 | fda.958 | -4.709 | -4.79 |
| ZINC000004097426 | fda.959 | -4.709 | -4.79 |
| ZINC000022065398 | fda.346 | -4.708 | -4.708 |
| ZINC000002510358 | fda.912 | -4.707 | -5.069 |
| ZINC000053683345 | fda.1183 | -4.706 | -4.706 |
| ZINC000003881640 | fda.1884 | -4.705 | -4.705 |
| ZINC000003875357 | fda.416 | -4.704 | -4.704 |
| ZINC000003830961 | fda.2069 | -4.701 | -4.701 |
| ZINC000036294079 | fda.678 | -4.699 | -4.803 |
| ZINC000035999642 | fda.745 | -4.699 | -4.803 |
| ZINC000095626706 | fda.1318 | -4.697 | -5.413 |
| ZINC000011680067 | fda.1619 | -4.696 | -4.696 |
| ZINC000000075126 | fda.394 | -4.692 | -4.817 |
| ZINC000000004448 | fda.108 | -4.692 | -4.817 |
| ZINC000052971887 | fda.1188 | -4.691 | -4.972 |
| ZINC000053022902 | fda.1209 | -4.691 | -4.972 |
| ZINC000053022902 | fda.1210 | -4.691 | -4.972 |
| ZINC000000968305 | fda.1433 | -4.689 | -4.729 |
| ZINC000003875392 | fda.562 | -4.689 | -4.738 |
| ZINC000000000751 | fda.1414 | -4.689 | -4.738 |
| ZINC000000002216 | fda.569 | -4.689 | -5.247 |
| ZINC000000002216 | fda.570 | -4.689 | -5.247 |
| ZINC000085205448 | fda.35 | -4.688 | -4.688 |
| ZINC000003875392 | fda.563 | -4.688 | -4.737 |
| ZINC000003875392 | fda.564 | -4.688 | -4.737 |
| ZINC000000000751 | fda.1415 | -4.688 | -4.737 |
| ZINC000000000751 | fda.1416 | -4.688 | -4.737 |
| ZINC000043200832 | fda.1848 | -4.687 | -4.918 |
| ZINC000043200832 | fda.1849 | -4.687 | -4.918 |
| ZINC000043200832 | fda.1850 | -4.687 | -4.918 |
| ZINC000000000490 | fda.1253 | -4.687 | -4.775 |
| ZINC000000113442 | fda.138 | -4.685 | -4.696 |
| ZINC000000601316 | fda.1225 | -4.685 | -4.685 |
| ZINC000003830579 | fda.1687 | -4.685 | -4.685 |
| ZINC000012503151 | fda.566 | -4.684 | -4.967 |
| ZINC000253917094 | fda.2094 | -4.684 | -4.967 |
| ZINC000006021043 | fda.764 | -4.684 | -4.685 |
| ZINC000008466459 | fda.71 | -4.684 | -4.685 |
| ZINC000001611274 | fda.585 | -4.684 | -5.515 |
| ZINC000001611274 | fda.586 | -4.684 | -5.515 |
| ZINC000003815424 | fda.132 | -4.683 | -4.683 |
| ZINC000000897385 | fda.1426 | -4.682 | -4.941 |
| ZINC000000608101 | fda.1228 | -4.682 | -4.941 |
| ZINC000000968328 | fda.402 | -4.68 | -5.204 |
| ZINC000000968330 | fda.1172 | -4.68 | -5.204 |
| ZINC000011679756 | fda.469 | -4.678 | -4.889 |
| ZINC000000000655 | fda.1219 | -4.678 | -4.678 |
| ZINC000000403566 | fda.1679 | -4.678 | -4.678 |
| ZINC000003861599 | fda.1882 | -4.678 | -4.678 |
| ZINC000004097304 | fda.1986 | -4.676 | -4.676 |
| ZINC000003875357 | fda.416 | -4.676 | -4.676 |
| ZINC000000896740 | fda.1497 | -4.675 | -4.882 |
| ZINC000000607971 | fda.1506 | -4.675 | -4.882 |
| ZINC000001482049 | fda.165 | -4.673 | -4.673 |
| ZINC000003812989 | fda.1712 | -4.673 | -4.703 |
| ZINC000003812989 | fda.1713 | -4.673 | -4.703 |
| ZINC000001530579 | fda.1087 | -4.672 | -4.715 |
| ZINC000001530580 | fda.1089 | -4.672 | -4.715 |
| ZINC000001850376 | fda.1777 | -4.672 | -4.672 |
| ZINC000004468778 | fda.321 | -4.672 | -4.672 |
| ZINC000001995484 | fda.1275 | -4.671 | -4.884 |
| ZINC000003794794 | fda.2044 | -4.671 | -4.671 |
| ZINC000001849548 | fda.1749 | -4.67 | -4.67 |
| ZINC000000643114 | fda.1026 | -4.669 | -4.672 |
| ZINC000000601305 | fda.1613 | -4.669 | -4.672 |
| ZINC000003791297 | fda.1651 | -4.669 | -5.42 |
| ZINC000003932831 | fda.1751 | -4.668 | -4.668 |
| ZINC000070466416 | fda.537 | -4.668 | -4.668 |
| ZINC000003782818 | fda.2059 | -4.668 | -5.247 |
| ZINC000022010382 | fda.639 | -4.667 | -4.882 |
| ZINC000022010382 | fda.640 | -4.667 | -4.882 |
| ZINC000022010387 | fda.2101 | -4.667 | -4.882 |
| ZINC000022010387 | fda.2102 | -4.667 | -4.882 |
| ZINC000000006251 | fda.2007 | -4.667 | -4.698 |
| ZINC000000006251 | fda.2008 | -4.667 | -4.698 |
| ZINC000000075008 | fda.674 | -4.667 | -4.861 |
| ZINC000000075008 | fda.675 | -4.667 | -4.861 |
| ZINC000000901791 | fda.234 | -4.667 | -4.667 |
| ZINC000014210876 | fda.1798 | -4.666 | -5.425 |
| ZINC000034089131 | fda.73 | -4.666 | -4.681 |
| ZINC000011677857 | fda.479 | -4.666 | -6.577 |
| ZINC000003872605 | fda.1730 | -4.666 | -4.669 |
| ZINC000000968328 | fda.402 | -4.665 | -5.999 |
| ZINC000000968330 | fda.1172 | -4.665 | -5.999 |
| ZINC000002020233 | fda.406 | -4.665 | -4.665 |
| ZINC000000968326 | fda.114 | -4.664 | -5.147 |
| ZINC000000968326 | fda.115 | -4.664 | -5.147 |
| ZINC000000968327 | fda.1747 | -4.664 | -5.147 |
| ZINC000000968327 | fda.1748 | -4.664 | -5.147 |
| ZINC000000537791 | fda.1370 | -4.664 | -4.687 |
| ZINC000000537791 | fda.1371 | -4.664 | -4.687 |
| ZINC000000537791 | fda.1372 | -4.664 | -4.687 |
| ZINC000000000856 | fda.1152 | -4.663 | -4.675 |
| ZINC000000897258 | fda.2011 | -4.663 | -4.675 |
| ZINC000087515509 | fda.1287 | -4.662 | -5.855 |
| ZINC000000538273 | fda.952 | -4.66 | -4.804 |
| ZINC000000538273 | fda.953 | -4.66 | -4.804 |
| ZINC000000537891 | fda.1373 | -4.66 | -4.804 |
| ZINC000000537891 | fda.1374 | -4.66 | -4.804 |
| ZINC000003872994 | fda.218 | -4.66 | -4.985 |
| ZINC000000643153 | fda.1820 | -4.66 | -4.985 |
| ZINC000000005823 | fda.45 | -4.66 | -4.66 |
| ZINC000000538275 | fda.308 | -4.659 | -4.674 |
| ZINC000000538275 | fda.309 | -4.659 | -4.674 |
| ZINC000000000128 | fda.688 | -4.659 | -4.66 |
| ZINC000000896463 | fda.1077 | -4.659 | -4.66 |
| ZINC000021982937 | fda.2026 | -4.659 | -4.839 |
| ZINC000021982937 | fda.2027 | -4.659 | -4.839 |
| ZINC000021982937 | fda.2028 | -4.659 | -4.839 |
| ZINC000033965961 | fda.2036 | -4.659 | -4.839 |
| ZINC000033965961 | fda.2037 | -4.659 | -4.839 |
| ZINC000033965961 | fda.2038 | -4.659 | -4.839 |
| ZINC000004340269 | fda.997 | -4.656 | -4.657 |
| ZINC000001530761 | fda.1118 | -4.655 | -4.655 |
| ZINC000000968345 | fda.116 | -4.655 | -4.725 |
| ZINC000003651680 | fda.1689 | -4.655 | -4.725 |
| ZINC000001530567 | fda.1139 | -4.654 | -4.655 |
| ZINC000001530568 | fda.1140 | -4.654 | -4.655 |
| ZINC000101489663 | fda.1971 | -4.653 | -4.653 |
| ZINC000001530625 | fda.1796 | -4.653 | -4.653 |
| ZINC000001542113 | fda.329 | -4.652 | -4.753 |
| ZINC000001542113 | fda.330 | -4.652 | -4.753 |
| ZINC000004175630 | fda.948 | -4.651 | -4.658 |
| ZINC000004175630 | fda.949 | -4.651 | -4.658 |
| ZINC000000895099 | fda.612 | -4.65 | -4.65 |
| ZINC000000968375 | fda.506 | -4.65 | -4.65 |
| ZINC000000968326 | fda.114 | -4.649 | -5.089 |
| ZINC000000968327 | fda.1747 | -4.649 | -5.089 |
| ZINC000000000905 | fda.1155 | -4.648 | -4.648 |
| ZINC000100037020 | fda.1535 | -4.648 | -5.358 |
| ZINC000004658290 | fda.14 | -4.648 | -4.77 |
| ZINC000000056568 | fda.1417 | -4.647 | -4.65 |
| ZINC000031274852 | fda.347 | -4.647 | -4.652 |
| ZINC000031274852 | fda.348 | -4.647 | -4.652 |
| ZINC000052971887 | fda.1187 | -4.646 | -4.926 |
| ZINC000002510358 | fda.913 | -4.646 | -5.007 |
| ZINC000003872994 | fda.218 | -4.645 | -4.97 |
| ZINC000000643153 | fda.1820 | -4.645 | -4.97 |
| ZINC000003831474 | fda.1973 | -4.645 | -4.645 |
| ZINC000000005823 | fda.44 | -4.644 | -4.719 |
| ZINC000003798734 | fda.1647 | -4.643 | -4.647 |
| ZINC000003818808 | fda.491 | -4.643 | -4.655 |
| ZINC000003831490 | fda.374 | -4.643 | -4.863 |
| ZINC000002008310 | fda.1966 | -4.643 | -4.643 |
| ZINC000000602128 | fda.49 | -4.642 | -4.647 |
| ZINC000003871703 | fda.1451 | -4.642 | -4.647 |
| ZINC000100003902 | fda.60 | -4.641 | -4.641 |
| ZINC000019632912 | fda.713 | -4.641 | -5.128 |
| ZINC000003872931 | fda.547 | -4.639 | -4.645 |
| ZINC000084758479 | fda.1807 | -4.638 | -4.774 |
| ZINC000084758479 | fda.1808 | -4.638 | -4.774 |
| ZINC000000004778 | fda.148 | -4.637 | -4.637 |
| ZINC000028639340 | fda.177 | -4.637 | -4.654 |
| ZINC000013545634 | fda.703 | -4.636 | -4.705 |
| ZINC000013545636 | fda.387 | -4.636 | -4.705 |
| ZINC000013831141 | fda.2075 | -4.636 | -4.636 |
| ZINC000000968345 | fda.117 | -4.636 | -4.705 |
| ZINC000003651680 | fda.1690 | -4.636 | -4.705 |
| ZINC000001530930 | fda.1953 | -4.634 | -4.691 |
| ZINC000003876069 | fda.1979 | -4.634 | -4.691 |
| ZINC000021982937 | fda.2024 | -4.634 | -4.814 |
| ZINC000021982937 | fda.2025 | -4.634 | -4.814 |
| ZINC000033965961 | fda.2034 | -4.634 | -4.814 |
| ZINC000033965961 | fda.2035 | -4.634 | -4.814 |
| ZINC000000075126 | fda.394 | -4.633 | -4.758 |
| ZINC000000004448 | fda.108 | -4.633 | -4.758 |
| ZINC000001849548 | fda.1749 | -4.632 | -4.632 |
| ZINC000008214573 | fda.241 | -4.631 | -4.631 |
| ZINC000004658562 | fda.622 | -4.631 | -4.631 |
| ZINC000004658560 | fda.1464 | -4.631 | -4.631 |
| ZINC000003830500 | fda.596 | -4.63 | -4.877 |
| ZINC000004099008 | fda.2015 | -4.63 | -4.63 |
| ZINC000001531009 | fda.932 | -4.627 | -5.42 |
| ZINC000003830500 | fda.595 | -4.626 | -4.873 |
| ZINC000000000431 | fda.1518 | -4.626 | -4.626 |
| ZINC000000896595 | fda.1643 | -4.626 | -4.626 |
| ZINC000001530930 | fda.1952 | -4.626 | -4.683 |
| ZINC000003876069 | fda.1978 | -4.626 | -4.683 |
| ZINC000000968345 | fda.117 | -4.626 | -4.695 |
| ZINC000003651680 | fda.1690 | -4.626 | -4.695 |
| ZINC000001530654 | fda.1622 | -4.625 | -4.675 |
| ZINC000001530654 | fda.1623 | -4.625 | -4.675 |
| ZINC000000601250 | fda.1504 | -4.624 | -5.126 |
| ZINC000000601250 | fda.1505 | -4.624 | -5.126 |
| ZINC000003873371 | fda.1092 | -4.624 | -5.126 |
| ZINC000003873371 | fda.1093 | -4.624 | -5.126 |
| ZINC000003781943 | fda.1693 | -4.62 | -4.976 |
| ZINC000034676245 | fda.285 | -4.619 | -4.866 |
| ZINC000034676245 | fda.286 | -4.619 | -4.866 |
| ZINC000034676245 | fda.287 | -4.619 | -4.866 |
| ZINC000001530713 | fda.788 | -4.619 | -4.825 |
| ZINC000004217732 | fda.364 | -4.617 | -4.621 |
| ZINC000003812865 | fda.831 | -4.617 | -4.617 |
| ZINC000003776970 | fda.1692 | -4.617 | -4.899 |
| ZINC000013540519 | fda.1066 | -4.617 | -4.617 |
| ZINC000052971887 | fda.1187 | -4.614 | -4.895 |
| ZINC000001853550 | fda.1688 | -4.613 | -4.626 |
| ZINC000008214703 | fda.511 | -4.612 | -4.616 |
| ZINC000003956788 | fda.58 | -4.611 | -4.611 |
| ZINC000000896663 | fda.487 | -4.61 | -4.639 |
| ZINC000001639567 | fda.1646 | -4.61 | -4.639 |
| ZINC000003833821 | fda.1881 | -4.61 | -4.61 |
| ZINC000101489663 | fda.1971 | -4.609 | -4.609 |
| ZINC000000006251 | fda.2007 | -4.607 | -4.637 |
| ZINC000000006251 | fda.2008 | -4.607 | -4.637 |
| ZINC000000075008 | fda.674 | -4.606 | -4.801 |
| ZINC000000075008 | fda.675 | -4.606 | -4.801 |
| ZINC000000000416 | fda.1487 | -4.606 | -4.634 |
| ZINC000000000416 | fda.1488 | -4.606 | -4.634 |
| ZINC000000403011 | fda.1771 | -4.606 | -4.634 |
| ZINC000000403011 | fda.1772 | -4.606 | -4.634 |
| ZINC000012360535 | fda.101 | -4.605 | -5.129 |
| ZINC000001530652 | fda.461 | -4.605 | -4.605 |
| ZINC000001530652 | fda.462 | -4.605 | -4.605 |
| ZINC000001530654 | fda.1624 | -4.605 | -4.605 |
| ZINC000001530654 | fda.1625 | -4.605 | -4.605 |
| ZINC000001482197 | fda.456 | -4.604 | -4.633 |
| ZINC000000896666 | fda.81 | -4.604 | -4.633 |
| ZINC000019632912 | fda.713 | -4.604 | -4.947 |
| ZINC000001530764 | fda.895 | -4.602 | -4.602 |
| ZINC000000000596 | fda.1441 | -4.602 | -4.614 |
| ZINC000000000596 | fda.1442 | -4.602 | -4.614 |
| ZINC000058581064 | fda.1199 | -4.6 | -5.225 |
| ZINC000095616601 | fda.18 | -4.6 | -4.606 |
| ZINC000012495062 | fda.1269 | -4.599 | -4.602 |
| ZINC000000607986 | fda.62 | -4.599 | -4.619 |
| ZINC000000000373 | fda.504 | -4.597 | -4.597 |
| ZINC000001690604 | fda.66 | -4.597 | -4.597 |
| ZINC000003875484 | fda.1003 | -4.597 | -4.597 |
| ZINC000012414057 | fda.312 | -4.597 | -4.646 |
| ZINC000003823475 | fda.558 | -4.597 | -4.598 |
| ZINC000003823475 | fda.559 | -4.597 | -4.598 |
| ZINC000003823475 | fda.560 | -4.597 | -4.598 |
| ZINC000003812913 | fda.975 | -4.596 | -4.596 |
| ZINC000003831138 | fda.1007 | -4.596 | -4.596 |
| ZINC000003776633 | fda.2052 | -4.596 | -4.601 |
| ZINC000003776633 | fda.2053 | -4.596 | -4.601 |
| ZINC000100009280 | fda.1244 | -4.595 | -4.598 |
| ZINC000000897089 | fda.1345 | -4.595 | -4.598 |
| ZINC000003874185 | fda.1975 | -4.595 | -4.598 |
| ZINC000000007673 | fda.817 | -4.593 | -4.593 |
| ZINC000000601229 | fda.1610 | -4.591 | -4.592 |
| ZINC000000601229 | fda.1611 | -4.591 | -4.592 |
| ZINC000000601229 | fda.1612 | -4.591 | -4.592 |
| ZINC000003861806 | fda.24 | -4.591 | -4.591 |
| ZINC000003874715 | fda.1386 | -4.591 | -4.591 |
| ZINC000000608382 | fda.51 | -4.59 | -4.6 |
| ZINC000000608382 | fda.52 | -4.59 | -4.6 |
| ZINC000000014360 | fda.1603 | -4.59 | -4.594 |
| ZINC000001280665 | fda.412 | -4.59 | -4.619 |
| ZINC000001280665 | fda.414 | -4.59 | -4.619 |
| ZINC000004097467 | fda.1987 | -4.588 | -4.588 |
| ZINC000003801163 | fda.1709 | -4.588 | -5.462 |
| ZINC000085205451 | fda.1933 | -4.588 | -4.588 |
| ZINC000003791297 | fda.1651 | -4.588 | -4.783 |
| ZINC000013545634 | fda.704 | -4.586 | -4.654 |
| ZINC000013545636 | fda.388 | -4.586 | -4.654 |
| ZINC000100008319 | fda.191 | -4.585 | -4.585 |
| ZINC000030691797 | fda.513 | -4.583 | -4.583 |
| ZINC000096006009 | fda.1290 | -4.582 | -4.582 |
| ZINC000096006009 | fda.1291 | -4.582 | -4.582 |
| ZINC000001530641 | fda.898 | -4.582 | -4.585 |
| ZINC000043100953 | fda.1806 | -4.582 | -5.775 |
| ZINC000002169830 | fda.1400 | -4.582 | -4.582 |
| ZINC000064033452 | fda.1201 | -4.582 | -4.582 |
| ZINC000000000449 | fda.1486 | -4.581 | -4.581 |
| ZINC000003813083 | fda.1744 | -4.581 | -4.581 |
| ZINC000095616599 | fda.1289 | -4.579 | -4.58 |
| ZINC000001850374 | fda.2049 | -4.578 | -4.578 |
| ZINC000001530814 | fda.798 | -4.578 | -4.632 |
| ZINC000001530814 | fda.799 | -4.578 | -4.632 |
| ZINC000001530814 | fda.800 | -4.578 | -4.632 |
| ZINC000000039089 | fda.1157 | -4.577 | -6.922 |
| ZINC000006716957 | fda.871 | -4.577 | -5.502 |
| ZINC000085537017 | fda.2092 | -4.576 | -6.803 |
| ZINC000000005151 | fda.1418 | -4.576 | -4.582 |
| ZINC000000005151 | fda.1419 | -4.576 | -4.582 |
| ZINC000003806262 | fda.410 | -4.574 | -6.364 |
| ZINC000003806262 | fda.411 | -4.574 | -6.364 |
| ZINC000007997897 | fda.1128 | -4.574 | -4.653 |
| ZINC000007997897 | fda.1129 | -4.574 | -4.653 |
| ZINC000007997897 | fda.1130 | -4.574 | -4.653 |
| ZINC000000001728 | fda.1151 | -4.573 | -4.579 |
| ZINC000000057254 | fda.1071 | -4.572 | -4.577 |
| ZINC000000057254 | fda.1072 | -4.572 | -4.577 |
| ZINC000000057253 | fda.1272 | -4.572 | -4.577 |
| ZINC000000057253 | fda.1273 | -4.572 | -4.577 |
| ZINC000003801919 | fda.1466 | -4.57 | -4.77 |
| ZINC000095619101 | fda.1305 | -4.57 | -4.601 |
| ZINC000095619101 | fda.1306 | -4.57 | -4.601 |
| ZINC000095619100 | fda.1327 | -4.57 | -4.601 |
| ZINC000095619100 | fda.1328 | -4.57 | -4.601 |
| ZINC000003882036 | fda.441 | -4.57 | -4.57 |
| ZINC000003812863 | fda.1649 | -4.569 | -4.691 |
| ZINC000003806262 | fda.410 | -4.569 | -6.359 |
| ZINC000003806262 | fda.411 | -4.569 | -6.359 |
| ZINC000000001773 | fda.1843 | -4.568 | -4.621 |
| ZINC000000001773 | fda.1844 | -4.568 | -4.621 |
| ZINC000013585233 | fda.273 | -4.566 | -4.585 |
| ZINC000000607939 | fda.50 | -4.565 | -4.565 |
| ZINC000100001918 | fda.1319 | -4.565 | -4.565 |
| ZINC000070466416 | fda.537 | -4.564 | -4.564 |
| ZINC000000057435 | fda.371 | -4.564 | -4.564 |
| ZINC000084589076 | fda.1911 | -4.561 | -6.559 |
| ZINC000100006264 | fda.1917 | -4.561 | -6.559 |
| ZINC000000000507 | fda.1564 | -4.561 | -4.669 |
| ZINC000000403079 | fda.1678 | -4.561 | -4.669 |
| ZINC000000020257 | fda.1607 | -4.561 | -4.566 |
| ZINC000000057062 | fda.1765 | -4.561 | -4.566 |
| ZINC000000538627 | fda.863 | -4.559 | -4.559 |
| ZINC000013585233 | fda.272 | -4.559 | -4.579 |
| ZINC000013585233 | fda.274 | -4.559 | -4.579 |
| ZINC000000895360 | fda.1378 | -4.556 | -4.557 |
| ZINC000035801098 | fda.1547 | -4.556 | -4.598 |
| ZINC000019632706 | fda.382 | -4.555 | -4.555 |
| ZINC000000591993 | fda.383 | -4.555 | -4.555 |
| ZINC000003812851 | fda.266 | -4.555 | -4.682 |
| ZINC000013540519 | fda.1066 | -4.555 | -4.555 |
| ZINC000004468778 | fda.321 | -4.553 | -4.553 |
| ZINC000164760756 | fda.1091 | -4.552 | -4.559 |
| ZINC000012360535 | fda.100 | -4.551 | -5.075 |
| ZINC000001530579 | fda.1086 | -4.546 | -4.589 |
| ZINC000001530580 | fda.1088 | -4.546 | -4.589 |
| ZINC000003830391 | fda.361 | -4.545 | -4.755 |
| ZINC000000538266 | fda.136 | -4.545 | -4.545 |
| ZINC000003814395 | fda.1106 | -4.544 | -4.544 |
| ZINC000008552123 | fda.1569 | -4.544 | -4.594 |
| ZINC000003875393 | fda.1718 | -4.544 | -4.594 |
| ZINC000003875393 | fda.1720 | -4.544 | -4.594 |
| ZINC000000596731 | fda.296 | -4.544 | -4.61 |
| ZINC000000596731 | fda.297 | -4.544 | -4.61 |
| ZINC000003803652 | fda.830 | -4.543 | -5.087 |
| ZINC000003798763 | fda.439 | -4.542 | -4.544 |
| ZINC000169621220 | fda.275 | -4.542 | -4.574 |
| ZINC000003881958 | fda.283 | -4.539 | -4.539 |
| ZINC000043202140 | fda.1828 | -4.538 | -4.738 |
| ZINC000043202140 | fda.1829 | -4.538 | -4.738 |
| ZINC000000901552 | fda.1531 | -4.538 | -4.545 |
| ZINC000000901555 | fda.1532 | -4.538 | -4.545 |
| ZINC000004074875 | fda.1213 | -4.538 | -4.694 |
| ZINC000000621893 | fda.1869 | -4.537 | -4.554 |
| ZINC000000621893 | fda.1870 | -4.537 | -4.554 |
| ZINC000000057464 | fda.1076 | -4.537 | -5.323 |
| ZINC000004097427 | fda.1215 | -4.536 | -4.692 |
| ZINC000000004076 | fda.291 | -4.536 | -4.537 |
| ZINC000000537931 | fda.1375 | -4.536 | -4.561 |
| ZINC000034676245 | fda.285 | -4.535 | -4.782 |
| ZINC000003830813 | fda.1653 | -4.534 | -4.78 |
| ZINC000003920719 | fda.384 | -4.533 | -4.535 |
| ZINC000011615926 | fda.478 | -4.533 | -4.535 |
| ZINC000003830391 | fda.362 | -4.533 | -4.742 |
| ZINC000000000856 | fda.1153 | -4.533 | -4.545 |
| ZINC000000897258 | fda.2012 | -4.533 | -4.545 |
| ZINC000000057512 | fda.1014 | -4.533 | -4.533 |
| ZINC000001530862 | fda.207 | -4.532 | -4.532 |
| ZINC000001530863 | fda.223 | -4.532 | -4.532 |
| ZINC000000000507 | fda.1564 | -4.531 | -4.639 |
| ZINC000000403079 | fda.1678 | -4.531 | -4.639 |
| ZINC000019144216 | fda.1600 | -4.531 | -4.536 |
| ZINC000019144216 | fda.1601 | -4.531 | -4.536 |
| ZINC000035801098 | fda.1547 | -4.529 | -6.867 |
| ZINC000085537017 | fda.2090 | -4.527 | -6.754 |
| ZINC000085537017 | fda.2091 | -4.527 | -6.754 |
| ZINC000004097392 | fda.954 | -4.526 | -4.526 |
| ZINC000003776875 | fda.1706 | -4.526 | -4.526 |
| ZINC000003913937 | fda.1885 | -4.525 | -4.527 |
| ZINC000003831531 | fda.1010 | -4.525 | -4.525 |
| ZINC000003831531 | fda.1011 | -4.525 | -4.525 |
| ZINC000000000507 | fda.1563 | -4.524 | -4.632 |
| ZINC000000403079 | fda.1677 | -4.524 | -4.632 |
| ZINC000000057464 | fda.1075 | -4.524 | -5.641 |
| ZINC000003802417 | fda.2064 | -4.523 | -4.528 |
| ZINC000000000346 | fda.289 | -4.523 | -4.523 |
| ZINC000000896968 | fda.1657 | -4.523 | -4.523 |
| ZINC000000622123 | fda.1786 | -4.522 | -4.522 |
| ZINC000000001979 | fda.1725 | -4.521 | -4.521 |
| ZINC000012661824 | fda.619 | -4.521 | -4.525 |
| ZINC000095616599 | fda.1289 | -4.517 | -4.518 |
| ZINC000022448696 | fda.1823 | -4.516 | -5.663 |
| ZINC000022448696 | fda.1824 | -4.516 | -5.663 |
| ZINC000022448696 | fda.1825 | -4.516 | -5.663 |
| ZINC000022448696 | fda.1826 | -4.516 | -5.663 |
| ZINC000022448696 | fda.1827 | -4.516 | -5.663 |
| ZINC000000403533 | fda.1176 | -4.516 | -4.613 |
| ZINC000000403533 | fda.1177 | -4.516 | -4.613 |
| ZINC000000403533 | fda.1178 | -4.516 | -4.613 |
| ZINC000004632106 | fda.1232 | -4.514 | -5.004 |
| ZINC000005029557 | fda.1064 | -4.514 | -4.514 |
| ZINC000003881958 | fda.283 | -4.513 | -4.513 |
| ZINC000004212851 | fda.994 | -4.513 | -4.513 |
| ZINC000034676245 | fda.286 | -4.51 | -4.757 |
| ZINC000034676245 | fda.287 | -4.51 | -4.757 |
| ZINC000003881640 | fda.1884 | -4.508 | -4.508 |
| ZINC000000403533 | fda.1176 | -4.505 | -5.626 |
| ZINC000000403533 | fda.1177 | -4.505 | -5.626 |
| ZINC000000403533 | fda.1178 | -4.505 | -5.626 |
| ZINC000003814395 | fda.1106 | -4.504 | -4.504 |
| ZINC000094566093 | fda.1304 | -4.504 | -4.951 |
| ZINC000000001281 | fda.1557 | -4.502 | -4.504 |
| ZINC000003938744 | fda.1889 | -4.502 | -4.502 |
| ZINC000000897089 | fda.1345 | -4.501 | -4.503 |
| ZINC000003874185 | fda.1975 | -4.501 | -4.503 |
| ZINC000100070937 | fda.535 | -4.5 | -4.5 |
| ZINC000003813078 | fda.603 | -4.5 | -4.502 |
| ZINC000000014864 | fda.451 | -4.5 | -4.5 |
| ZINC000003787060 | fda.1875 | -4.498 | -4.498 |
| ZINC000001530638 | fda.1621 | -4.498 | -4.498 |
| ZINC000001530637 | fda.1098 | -4.498 | -4.498 |
| ZINC000003986735 | fda.990 | -4.494 | -4.928 |
| ZINC000003986735 | fda.991 | -4.494 | -4.928 |
| ZINC000084757007 | fda.540 | -4.493 | -4.493 |
| ZINC000095616600 | fda.1326 | -4.493 | -4.494 |
| ZINC000003812867 | fda.1743 | -4.49 | -4.745 |
| ZINC000000004319 | fda.317 | -4.488 | -4.516 |
| ZINC000000004319 | fda.318 | -4.488 | -4.516 |
| ZINC000000403010 | fda.1457 | -4.488 | -4.516 |
| ZINC000000403010 | fda.1458 | -4.488 | -4.516 |
| ZINC000019702309 | fda.725 | -4.485 | -4.651 |
| ZINC000000075126 | fda.393 | -4.484 | -4.609 |
| ZINC000000004448 | fda.107 | -4.484 | -4.609 |
| ZINC000006661227 | fda.1196 | -4.482 | -4.939 |
| ZINC000100018854 | fda.500 | -4.482 | -4.483 |
| ZINC000100009278 | fda.1241 | -4.481 | -4.484 |
| ZINC000052509463 | fda.1204 | -4.481 | -4.481 |
| ZINC000052509463 | fda.1205 | -4.481 | -4.481 |
| ZINC000052509463 | fda.1206 | -4.481 | -4.481 |
| ZINC000004097426 | fda.957 | -4.48 | -4.562 |
| ZINC000004097426 | fda.958 | -4.48 | -4.562 |
| ZINC000004097426 | fda.959 | -4.48 | -4.562 |
| ZINC000013298313 | fda.594 | -4.48 | -4.506 |
| ZINC000164528615 | fda.2089 | -4.48 | -4.482 |
| ZINC000000000856 | fda.1152 | -4.479 | -4.491 |
| ZINC000000897258 | fda.2011 | -4.479 | -4.491 |
| ZINC000016052277 | fda.1895 | -4.479 | -4.56 |
| ZINC000016052277 | fda.1896 | -4.479 | -4.56 |
| ZINC000016052277 | fda.1898 | -4.479 | -4.56 |
| ZINC000004097225 | fda.956 | -4.478 | -4.478 |
| ZINC000000000346 | fda.289 | -4.477 | -4.477 |
| ZINC000000896968 | fda.1657 | -4.477 | -4.477 |
| ZINC000003830405 | fda.1880 | -4.476 | -4.476 |
| ZINC000000000347 | fda.1938 | -4.476 | -4.477 |
| ZINC000003922770 | fda.554 | -4.476 | -4.876 |
| ZINC000004097305 | fda.945 | -4.475 | -4.475 |
| ZINC000003812869 | fda.2048 | -4.474 | -5.107 |
| ZINC000118912517 | fda.1314 | -4.471 | -4.471 |
| ZINC000019594599 | fda.773 | -4.47 | -4.645 |
| ZINC000019594599 | fda.774 | -4.47 | -4.645 |
| ZINC000019594599 | fda.775 | -4.47 | -4.645 |
| ZINC000100070937 | fda.535 | -4.47 | -4.47 |
| ZINC000003872055 | fda.1398 | -4.47 | -5.004 |
| ZINC000000000490 | fda.1253 | -4.47 | -5.642 |
| ZINC000000020220 | fda.370 | -4.469 | -5.573 |
| ZINC000000968310 | fda.331 | -4.469 | -5.731 |
| ZINC000000968310 | fda.332 | -4.469 | -5.731 |
| ZINC000000968310 | fda.333 | -4.469 | -5.731 |
| ZINC000000000509 | fda.1549 | -4.469 | -5.731 |
| ZINC000000000509 | fda.1550 | -4.469 | -5.731 |
| ZINC000000000509 | fda.1551 | -4.469 | -5.731 |
| ZINC000003807172 | fda.1682 | -4.468 | -4.468 |
| ZINC000000968273 | fda.373 | -4.468 | -4.469 |
| ZINC000000968274 | fda.1435 | -4.468 | -4.469 |
| ZINC000003875259 | fda.962 | -4.468 | -4.468 |
| ZINC000014879992 | fda.732 | -4.467 | -4.502 |
| ZINC000014879992 | fda.734 | -4.467 | -4.502 |
| ZINC000014879992 | fda.735 | -4.467 | -4.502 |
| ZINC000000006300 | fda.433 | -4.466 | -4.466 |
| ZINC000095616600 | fda.1326 | -4.465 | -4.466 |
| ZINC000011617039 | fda.482 | -4.465 | -4.471 |
| ZINC000000001773 | fda.1842 | -4.464 | -6.509 |
| ZINC000000897251 | fda.112 | -4.464 | -4.467 |
| ZINC000000897251 | fda.113 | -4.464 | -4.467 |
| ZINC000000597013 | fda.1510 | -4.464 | -4.467 |
| ZINC000000597013 | fda.1511 | -4.464 | -4.467 |
| ZINC000000020228 | fda.325 | -4.46 | -4.697 |
| ZINC000000057206 | fda.987 | -4.46 | -4.697 |
| ZINC000000020257 | fda.1607 | -4.459 | -4.464 |
| ZINC000000057062 | fda.1765 | -4.459 | -4.464 |
| ZINC000003872738 | fda.1717 | -4.458 | -4.463 |
| ZINC000000391812 | fda.1054 | -4.457 | -4.481 |
| ZINC000000391812 | fda.1055 | -4.457 | -4.481 |
| ZINC000000391812 | fda.1056 | -4.457 | -4.481 |
| ZINC000000968257 | fda.1746 | -4.456 | -4.459 |
| ZINC000012466082 | fda.858 | -4.451 | -4.455 |
| ZINC000252678020 | fda.1935 | -4.451 | -4.455 |
| ZINC000000057340 | fda.1074 | -4.447 | -4.447 |
| ZINC000000057341 | fda.1764 | -4.447 | -4.447 |
| ZINC000100018854 | fda.499 | -4.447 | -4.448 |
| ZINC000100018854 | fda.501 | -4.447 | -4.448 |
| ZINC000100074252 | fda.1300 | -4.446 | -4.449 |
| ZINC000000006310 | fda.466 | -4.444 | -4.444 |
| ZINC000004097392 | fda.954 | -4.442 | -4.442 |
| ZINC000003776875 | fda.1706 | -4.442 | -4.442 |
| ZINC000000012346 | fda.1592 | -4.442 | -4.442 |
| ZINC000052955754 | fda.129 | -4.441 | -6.78 |
| ZINC000052955754 | fda.130 | -4.441 | -6.78 |
| ZINC000052955754 | fda.131 | -4.441 | -6.78 |
| ZINC000053683345 | fda.1183 | -4.441 | -4.441 |
| ZINC000003830215 | fda.1245 | -4.44 | -5.206 |
| ZINC000000001773 | fda.1842 | -4.44 | -4.493 |
| ZINC000001552174 | fda.1840 | -4.439 | -4.439 |
| ZINC000000602632 | fda.301 | -4.439 | -4.439 |
| ZINC000003830218 | fda.282 | -4.436 | -4.664 |
| ZINC000000968326 | fda.114 | -4.434 | -4.874 |
| ZINC000000968327 | fda.1747 | -4.434 | -4.874 |
| ZINC000003992105 | fda.2014 | -4.433 | -4.433 |
| ZINC000003812869 | fda.2048 | -4.432 | -5.065 |
| ZINC000000001798 | fda.1135 | -4.431 | -4.456 |
| ZINC000000001798 | fda.1136 | -4.431 | -4.456 |
| ZINC000000001798 | fda.1137 | -4.431 | -4.456 |
| ZINC000003951740 | fda.673 | -4.431 | -4.431 |
| ZINC000004258316 | fda.180 | -4.43 | -5.334 |
| ZINC000001530725 | fda.886 | -4.43 | -4.43 |
| ZINC000001530652 | fda.461 | -4.43 | -4.43 |
| ZINC000001530652 | fda.462 | -4.43 | -4.43 |
| ZINC000001530654 | fda.1624 | -4.43 | -4.43 |
| ZINC000001530654 | fda.1625 | -4.43 | -4.43 |
| ZINC000003812862 | fda.431 | -4.429 | -4.54 |
| ZINC000000000903 | fda.1839 | -4.429 | -4.431 |
| ZINC000013545634 | fda.703 | -4.428 | -4.497 |
| ZINC000013545634 | fda.704 | -4.428 | -4.497 |
| ZINC000013545636 | fda.387 | -4.428 | -4.497 |
| ZINC000013545636 | fda.388 | -4.428 | -4.497 |
| ZINC000008015016 | fda.1597 | -4.428 | -4.741 |
| ZINC000002032615 | fda.943 | -4.427 | -4.427 |
| ZINC000001850377 | fda.1430 | -4.427 | -4.427 |
| ZINC000003876136 | fda.565 | -4.427 | -4.427 |
| ZINC000003875332 | fda.249 | -4.427 | -4.427 |
| ZINC000003875334 | fda.1060 | -4.426 | -4.426 |
| ZINC000001542002 | fda.1874 | -4.425 | -4.468 |
| ZINC000004474443 | fda.510 | -4.425 | -5 |
| ZINC000084758235 | fda.828 | -4.424 | -4.451 |
| ZINC000053084692 | fda.75 | -4.424 | -4.61 |
| ZINC000000601301 | fda.320 | -4.424 | -4.491 |
| ZINC000000897222 | fda.1658 | -4.424 | -4.491 |
| ZINC000003826253 | fda.2057 | -4.424 | -4.522 |
| ZINC000100032379 | fda.1294 | -4.423 | -5.231 |
| ZINC000001530427 | fda.1 | -4.421 | -5.046 |
| ZINC000001530427 | fda.2 | -4.421 | -5.046 |
| ZINC000001530427 | fda.3 | -4.421 | -5.046 |
| ZINC000019166991 | fda.1578 | -4.421 | -4.775 |
| ZINC000003833821 | fda.1881 | -4.42 | -4.42 |
| ZINC000049783788 | fda.2085 | -4.417 | -4.418 |
| ZINC000021303210 | fda.1447 | -4.417 | -4.466 |
| ZINC000000020251 | fda.1541 | -4.417 | -4.417 |
| ZINC000001995484 | fda.1276 | -4.417 | -4.63 |
| ZINC000003812863 | fda.1648 | -4.415 | -5.412 |
| ZINC000003830999 | fda.604 | -4.414 | -4.517 |
| ZINC000003876186 | fda.1390 | -4.411 | -4.411 |
| ZINC000004693575 | fda.324 | -4.411 | -4.445 |
| ZINC000004693574 | fda.1218 | -4.411 | -4.445 |
| ZINC000012402836 | fda.1446 | -4.41 | -4.43 |
| ZINC000000000655 | fda.1219 | -4.41 | -4.41 |
| ZINC000000403566 | fda.1679 | -4.41 | -4.41 |
| ZINC000000113446 | fda.419 | -4.409 | -4.442 |
| ZINC000006661227 | fda.1196 | -4.407 | -4.864 |
| ZINC000013682481 | fda.753 | -4.407 | -5.363 |
| ZINC000008577218 | fda.251 | -4.407 | -5.29 |
| ZINC000000004028 | fda.1615 | -4.407 | -4.413 |
| ZINC000000004028 | fda.1616 | -4.407 | -4.413 |
| ZINC000000607986 | fda.62 | -4.406 | -4.426 |
| ZINC000022448696 | fda.1823 | -4.406 | -4.797 |
| ZINC000022448696 | fda.1824 | -4.406 | -4.797 |
| ZINC000022448696 | fda.1827 | -4.406 | -4.797 |
| ZINC000094566093 | fda.1303 | -4.405 | -4.536 |
| ZINC000094566092 | fda.1315 | -4.405 | -4.536 |
| ZINC000000020220 | fda.369 | -4.403 | -5.507 |
| ZINC000019875504 | fda.1901 | -4.401 | -4.818 |
| ZINC000003875483 | fda.548 | -4.398 | -5.485 |
| ZINC000003875483 | fda.549 | -4.398 | -5.485 |
| ZINC000003875483 | fda.550 | -4.398 | -5.485 |
| ZINC000004693575 | fda.324 | -4.397 | -6.107 |
| ZINC000004693574 | fda.1218 | -4.397 | -6.107 |
| ZINC000004097416 | fda.1618 | -4.393 | -4.393 |
| ZINC000012466082 | fda.859 | -4.391 | -4.394 |
| ZINC000252678020 | fda.1936 | -4.391 | -4.394 |
| ZINC000000016154 | fda.452 | -4.39 | -4.399 |
| ZINC000019875504 | fda.1902 | -4.385 | -4.803 |
| ZINC000001530427 | fda.1 | -4.384 | -5.01 |
| ZINC000001530427 | fda.2 | -4.384 | -5.01 |
| ZINC000001530427 | fda.3 | -4.384 | -5.01 |
| ZINC000003978005 | fda.1235 | -4.383 | -6.569 |
| ZINC000003978005 | fda.1236 | -4.383 | -6.569 |
| ZINC000003978005 | fda.1237 | -4.383 | -6.569 |
| ZINC000000968273 | fda.373 | -4.382 | -4.383 |
| ZINC000000968274 | fda.1435 | -4.382 | -4.383 |
| ZINC000000968263 | fda.1171 | -4.379 | -4.383 |
| ZINC000003993855 | fda.1279 | -4.378 | -4.378 |
| ZINC000000537822 | fda.437 | -4.378 | -4.391 |
| ZINC000000537822 | fda.438 | -4.378 | -4.391 |
| ZINC000001552908 | fda.1034 | -4.377 | -4.379 |
| ZINC000003873160 | fda.1455 | -4.375 | -4.375 |
| ZINC000004474443 | fda.509 | -4.373 | -4.948 |
| ZINC000001530935 | fda.682 | -4.373 | -6.073 |
| ZINC000004618208 | fda.873 | -4.373 | -6.073 |
| ZINC000004102194 | fda.1109 | -4.373 | -4.376 |
| ZINC000003871701 | fda.597 | -4.373 | -4.373 |
| ZINC000000114124 | fda.589 | -4.372 | -4.386 |
| ZINC000000114127 | fda.590 | -4.372 | -4.386 |
| ZINC000100015048 | fda.254 | -4.372 | -4.372 |
| ZINC000003830218 | fda.281 | -4.372 | -4.6 |
| ZINC000001530725 | fda.886 | -4.372 | -4.372 |
| ZINC000043207851 | fda.1830 | -4.371 | -4.371 |
| ZINC000000000973 | fda.475 | -4.369 | -4.694 |
| ZINC000003978005 | fda.1235 | -4.369 | -6.555 |
| ZINC000003978005 | fda.1236 | -4.369 | -6.555 |
| ZINC000003978005 | fda.1237 | -4.369 | -6.555 |
| ZINC000011679756 | fda.469 | -4.368 | -5.233 |
| ZINC000001531008 | fda.235 | -4.365 | -4.365 |
| ZINC000001530600 | fda.1143 | -4.364 | -4.917 |
| ZINC000001530600 | fda.1144 | -4.364 | -4.917 |
| ZINC000001530600 | fda.1145 | -4.364 | -4.917 |
| ZINC000035024346 | fda.1252 | -4.363 | -4.366 |
| ZINC000253632968 | fda.2093 | -4.36 | -4.367 |
| ZINC000003830960 | fda.83 | -4.36 | -4.36 |
| ZINC000003830958 | fda.168 | -4.36 | -4.36 |
| ZINC000013682481 | fda.753 | -4.359 | -5.315 |
| ZINC000085537014 | fda.906 | -4.358 | -4.888 |
| ZINC000085537014 | fda.907 | -4.358 | -4.888 |
| ZINC000019632618 | fda.757 | -4.356 | -4.563 |
| ZINC000019632618 | fda.758 | -4.356 | -4.563 |
| ZINC000019632618 | fda.759 | -4.356 | -4.563 |
| ZINC000008101109 | fda.423 | -4.356 | -4.363 |
| ZINC000008101109 | fda.425 | -4.356 | -4.363 |
| ZINC000003794711 | fda.407 | -4.356 | -4.375 |
| ZINC000003794711 | fda.408 | -4.356 | -4.375 |
| ZINC000003794711 | fda.409 | -4.356 | -4.375 |
| ZINC000003871923 | fda.1397 | -4.355 | -4.355 |
| ZINC000003976838 | fda.1761 | -4.354 | -4.536 |
| ZINC000003976838 | fda.1762 | -4.354 | -4.536 |
| ZINC000004097286 | fda.944 | -4.353 | -4.353 |
| ZINC000003938751 | fda.1756 | -4.353 | -4.353 |
| ZINC000021982937 | fda.2024 | -4.351 | -5.805 |
| ZINC000021982937 | fda.2025 | -4.351 | -5.805 |
| ZINC000006021043 | fda.764 | -4.349 | -4.35 |
| ZINC000008466459 | fda.71 | -4.349 | -4.35 |
| ZINC000000968326 | fda.115 | -4.349 | -4.789 |
| ZINC000000968327 | fda.1748 | -4.349 | -4.789 |
| ZINC000003873936 | fda.1732 | -4.348 | -4.594 |
| ZINC000003873936 | fda.1733 | -4.348 | -4.594 |
| ZINC000003813088 | fda.1460 | -4.345 | -4.348 |
| ZINC000000896958 | fda.1357 | -4.344 | -4.344 |
| ZINC000000968301 | fda.1921 | -4.344 | -4.344 |
| ZINC000004083606 | fda.385 | -4.342 | -4.342 |
| ZINC000034608502 | fda.2082 | -4.341 | -4.341 |
| ZINC000003871832 | fda.1727 | -4.34 | -4.346 |
| ZINC000003871832 | fda.1728 | -4.34 | -4.346 |
| ZINC000004097309 | fda.900 | -4.34 | -4.34 |
| ZINC000003920355 | fda.1394 | -4.34 | -4.34 |
| ZINC000003830993 | fda.1461 | -4.339 | -4.462 |
| ZINC000003830993 | fda.1462 | -4.339 | -4.462 |
| ZINC000001698306 | fda.1963 | -4.338 | -4.34 |
| ZINC000001550499 | fda.1859 | -4.338 | -4.349 |
| ZINC000003807804 | fda.5 | -4.336 | -4.735 |
| ZINC000001530621 | fda.812 | -4.336 | -4.4 |
| ZINC000000897322 | fda.1434 | -4.336 | -4.336 |
| ZINC000000000471 | fda.1484 | -4.336 | -4.336 |
| ZINC000001530713 | fda.789 | -4.336 | -4.542 |
| ZINC000000596731 | fda.296 | -4.336 | -4.401 |
| ZINC000000596731 | fda.297 | -4.336 | -4.401 |
| ZINC000000005895 | fda.46 | -4.335 | -4.339 |
| ZINC000002008866 | fda.654 | -4.334 | -4.334 |
| ZINC000001530601 | fda.923 | -4.334 | -4.354 |
| ZINC000000896958 | fda.1357 | -4.334 | -4.334 |
| ZINC000000968301 | fda.1921 | -4.334 | -4.334 |
| ZINC000003812306 | fda.2065 | -4.334 | -4.487 |
| ZINC000017146904 | fda.717 | -4.332 | -4.417 |
| ZINC000022447798 | fda.1821 | -4.33 | -4.43 |
| ZINC000022447798 | fda.1822 | -4.33 | -4.43 |
| ZINC000001530621 | fda.813 | -4.329 | -4.393 |
| ZINC000000896711 | fda.1080 | -4.329 | -4.33 |
| ZINC000000896709 | fda.1655 | -4.329 | -4.33 |
| ZINC000014164617 | fda.190 | -4.329 | -4.334 |
| ZINC000253530025 | fda.343 | -4.329 | -4.329 |
| ZINC000021303210 | fda.1448 | -4.329 | -4.378 |
| ZINC000000897251 | fda.112 | -4.328 | -4.331 |
| ZINC000000897251 | fda.113 | -4.328 | -4.331 |
| ZINC000000597013 | fda.1510 | -4.328 | -4.331 |
| ZINC000000597013 | fda.1511 | -4.328 | -4.331 |
| ZINC000000538621 | fda.854 | -4.328 | -4.426 |
| ZINC000000643138 | fda.1027 | -4.326 | -4.651 |
| ZINC000000643143 | fda.252 | -4.326 | -4.651 |
| ZINC000036294079 | fda.679 | -4.326 | -4.43 |
| ZINC000035999642 | fda.746 | -4.326 | -4.43 |
| ZINC000019203912 | fda.1583 | -4.325 | -4.872 |
| ZINC000019203912 | fda.1584 | -4.325 | -4.872 |
| ZINC000019203912 | fda.1585 | -4.325 | -4.872 |
| ZINC000003830500 | fda.595 | -4.325 | -4.962 |
| ZINC000000075126 | fda.393 | -4.324 | -4.449 |
| ZINC000000004448 | fda.107 | -4.324 | -4.449 |
| ZINC000000020231 | fda.293 | -4.324 | -4.327 |
| ZINC000001489478 | fda.457 | -4.323 | -4.325 |
| ZINC000001530688 | fda.683 | -4.323 | -4.323 |
| ZINC000003781943 | fda.1693 | -4.319 | -4.789 |
| ZINC000003776970 | fda.1691 | -4.319 | -4.601 |
| ZINC000000000128 | fda.688 | -4.318 | -4.319 |
| ZINC000000896463 | fda.1077 | -4.318 | -4.319 |
| ZINC000017146904 | fda.718 | -4.318 | -4.402 |
| ZINC000018099446 | fda.720 | -4.317 | -4.33 |
| ZINC000100019007 | fda.1312 | -4.317 | -4.33 |
| ZINC000004629876 | fda.256 | -4.317 | -4.317 |
| ZINC000003803652 | fda.830 | -4.316 | -5.848 |
| ZINC000000000449 | fda.1486 | -4.316 | -4.316 |
| ZINC000003813083 | fda.1744 | -4.316 | -4.316 |
| ZINC000004632106 | fda.1233 | -4.315 | -4.804 |
| ZINC000003830441 | fda.2061 | -4.315 | -4.315 |
| ZINC000006094354 | fda.765 | -4.314 | -4.315 |
| ZINC000006094354 | fda.766 | -4.314 | -4.315 |
| ZINC000003795819 | fda.1876 | -4.314 | -4.315 |
| ZINC000003795819 | fda.1877 | -4.314 | -4.315 |
| ZINC000000009073 | fda.1036 | -4.314 | -5.878 |
| ZINC000000009073 | fda.1037 | -4.314 | -5.878 |
| ZINC000000009073 | fda.1038 | -4.314 | -5.878 |
| ZINC000000402830 | fda.1174 | -4.312 | -4.313 |
| ZINC000000402830 | fda.1175 | -4.312 | -4.313 |
| ZINC000253476025 | fda.366 | -4.312 | -4.312 |
| ZINC000100015780 | fda.1283 | -4.312 | -4.312 |
| ZINC000004658562 | fda.622 | -4.312 | -4.312 |
| ZINC000004658560 | fda.1464 | -4.312 | -4.312 |
| ZINC000004099200 | fda.835 | -4.311 | -5.026 |
| ZINC000004676424 | fda.880 | -4.311 | -5.026 |
| ZINC000006382803 | fda.1024 | -4.311 | -5.65 |
| ZINC000005844788 | fda.188 | -4.311 | -4.33 |
| ZINC000003917708 | fda.1886 | -4.31 | -4.338 |
| ZINC000005456939 | fda.1127 | -4.308 | -4.312 |
| ZINC000036294079 | fda.678 | -4.307 | -4.412 |
| ZINC000035999642 | fda.745 | -4.307 | -4.412 |
| ZINC000000895099 | fda.612 | -4.307 | -4.307 |
| ZINC000000020240 | fda.1542 | -4.307 | -4.309 |
| ZINC000000056556 | fda.1722 | -4.307 | -4.309 |
| ZINC000035902489 | fda.1537 | -4.306 | -4.642 |
| ZINC000016052277 | fda.1895 | -4.304 | -4.385 |
| ZINC000016052277 | fda.1896 | -4.304 | -4.385 |
| ZINC000016052277 | fda.1898 | -4.304 | -4.385 |
| ZINC000000057512 | fda.1015 | -4.3 | -4.324 |
| ZINC000000057512 | fda.1016 | -4.3 | -4.324 |
| ZINC000000057512 | fda.1017 | -4.3 | -4.324 |
| ZINC000003875259 | fda.961 | -4.299 | -4.299 |
| ZINC000000114124 | fda.589 | -4.298 | -4.312 |
| ZINC000000114127 | fda.590 | -4.298 | -4.312 |
| ZINC000004693575 | fda.323 | -4.293 | -4.387 |
| ZINC000004693574 | fda.1217 | -4.293 | -4.387 |
| ZINC000001543475 | fda.1852 | -4.293 | -4.875 |
| ZINC000001543475 | fda.1853 | -4.293 | -4.875 |
| ZINC000001543475 | fda.1854 | -4.293 | -4.875 |
| ZINC000094566092 | fda.1316 | -4.292 | -4.725 |
| ZINC000150338699 | fda.2088 | -4.292 | -4.293 |
| ZINC000001690324 | fda.1961 | -4.291 | -5.916 |
| ZINC000001690324 | fda.1962 | -4.291 | -5.916 |
| ZINC000001576892 | fda.1259 | -4.288 | -4.29 |
| ZINC000003830500 | fda.596 | -4.288 | -4.926 |
| ZINC000022448696 | fda.1823 | -4.288 | -4.679 |
| ZINC000022448696 | fda.1824 | -4.288 | -4.679 |
| ZINC000022448696 | fda.1827 | -4.288 | -4.679 |
| ZINC000000097996 | fda.1948 | -4.288 | -4.698 |
| ZINC000004099200 | fda.836 | -4.285 | -4.992 |
| ZINC000004676424 | fda.881 | -4.285 | -4.992 |
| ZINC000001843099 | fda.1782 | -4.285 | -4.293 |
| ZINC000004474443 | fda.509 | -4.284 | -4.858 |
| ZINC000000643138 | fda.1027 | -4.283 | -4.608 |
| ZINC000000643143 | fda.252 | -4.283 | -4.608 |
| ZINC000001531008 | fda.235 | -4.283 | -4.283 |
| ZINC000004215234 | fda.2016 | -4.282 | -4.282 |
| ZINC000001530611 | fda.440 | -4.281 | -4.281 |
| ZINC000003812851 | fda.265 | -4.28 | -4.407 |
| ZINC000008577218 | fda.251 | -4.279 | -5.399 |
| ZINC000003779042 | fda.2054 | -4.279 | -4.28 |
| ZINC000000001115 | fda.1763 | -4.278 | -4.28 |
| ZINC000003812851 | fda.266 | -4.276 | -5.253 |
| ZINC000007997897 | fda.1128 | -4.274 | -4.354 |
| ZINC000007997897 | fda.1129 | -4.274 | -4.354 |
| ZINC000007997897 | fda.1130 | -4.274 | -4.354 |
| ZINC000000895360 | fda.1378 | -4.273 | -4.273 |
| ZINC000006661227 | fda.1197 | -4.272 | -4.728 |
| ZINC000006661227 | fda.1198 | -4.272 | -4.728 |
| ZINC000004392649 | fda.495 | -4.271 | -4.271 |
| ZINC000003882036 | fda.441 | -4.271 | -4.271 |
| ZINC000003833846 | fda.94 | -4.27 | -4.284 |
| ZINC000003833846 | fda.96 | -4.27 | -4.284 |
| ZINC000000897240 | fda.1362 | -4.27 | -4.271 |
| ZINC000000897240 | fda.1363 | -4.27 | -4.271 |
| ZINC000000897240 | fda.1364 | -4.27 | -4.271 |
| ZINC000003812867 | fda.1743 | -4.266 | -4.521 |
| ZINC000012484958 | fda.381 | -4.265 | -4.269 |
| ZINC000003830842 | fda.561 | -4.265 | -4.265 |
| ZINC000000896543 | fda.1078 | -4.265 | -4.265 |
| ZINC000019594557 | fda.755 | -4.263 | -4.456 |
| ZINC000019594557 | fda.756 | -4.263 | -4.456 |
| ZINC000019361042 | fda.762 | -4.263 | -4.456 |
| ZINC000019361042 | fda.763 | -4.263 | -4.456 |
| ZINC000003803652 | fda.830 | -4.263 | -5.626 |
| ZINC000004641374 | fda.340 | -4.262 | -4.262 |
| ZINC000022010382 | fda.641 | -4.262 | -4.476 |
| ZINC000022010387 | fda.2103 | -4.262 | -4.476 |
| ZINC000035902489 | fda.1538 | -4.261 | -4.261 |
| ZINC000021297660 | fda.121 | -4.261 | -4.331 |
| ZINC000021297660 | fda.122 | -4.261 | -4.331 |
| ZINC000004097286 | fda.944 | -4.26 | -4.26 |
| ZINC000003938751 | fda.1756 | -4.26 | -4.26 |
| ZINC000100378061 | fda.530 | -4.259 | -5.911 |
| ZINC000100378061 | fda.531 | -4.259 | -5.911 |
| ZINC000100378061 | fda.532 | -4.259 | -5.911 |
| ZINC000000000353 | fda.705 | -4.258 | -4.258 |
| ZINC000000394284 | fda.1181 | -4.258 | -4.258 |
| ZINC000003817234 | fda.2099 | -4.257 | -4.257 |
| ZINC000004658290 | fda.15 | -4.253 | -4.77 |
| ZINC000001546066 | fda.222 | -4.252 | -4.252 |
| ZINC000000005823 | fda.44 | -4.252 | -4.326 |
| ZINC000022010382 | fda.639 | -4.251 | -4.465 |
| ZINC000022010382 | fda.640 | -4.251 | -4.465 |
| ZINC000022010387 | fda.2101 | -4.251 | -4.465 |
| ZINC000022010387 | fda.2102 | -4.251 | -4.465 |
| ZINC000001530600 | fda.1143 | -4.249 | -4.545 |
| ZINC000001530600 | fda.1145 | -4.249 | -4.545 |
| ZINC000118912450 | fda.376 | -4.248 | -4.248 |
| ZINC000000001267 | fda.855 | -4.247 | -4.248 |
| ZINC000002522648 | fda.909 | -4.247 | -4.248 |
| ZINC000003920027 | fda.1471 | -4.247 | -4.247 |
| ZINC000003780893 | fda.248 | -4.245 | -4.245 |
| ZINC000000003876 | fda.313 | -4.245 | -4.588 |
| ZINC000003995811 | fda.2074 | -4.245 | -4.245 |
| ZINC000003876136 | fda.565 | -4.244 | -4.244 |
| ZINC000003875332 | fda.249 | -4.244 | -4.244 |
| ZINC000087515509 | fda.1287 | -4.244 | -4.852 |
| ZINC000001530977 | fda.890 | -4.243 | -4.323 |
| ZINC000001530977 | fda.891 | -4.243 | -4.323 |
| ZINC000001530977 | fda.892 | -4.243 | -4.323 |
| ZINC000006409735 | fda.1512 | -4.243 | -4.424 |
| ZINC000006409735 | fda.1513 | -4.243 | -4.424 |
| ZINC000004474443 | fda.509 | -4.242 | -4.524 |
| ZINC000000388081 | fda.1474 | -4.242 | -4.749 |
| ZINC000000538483 | fda.1367 | -4.241 | -4.644 |
| ZINC000000538483 | fda.1368 | -4.241 | -4.644 |
| ZINC000008552123 | fda.1567 | -4.24 | -4.289 |
| ZINC000008552123 | fda.1568 | -4.24 | -4.289 |
| ZINC000003875393 | fda.1719 | -4.24 | -4.289 |
| ZINC000022010379 | fda.636 | -4.239 | -4.945 |
| ZINC000022010379 | fda.637 | -4.239 | -4.945 |
| ZINC000022010379 | fda.638 | -4.239 | -4.945 |
| ZINC000022010375 | fda.2029 | -4.239 | -4.945 |
| ZINC000022010375 | fda.2030 | -4.239 | -4.945 |
| ZINC000022010375 | fda.2031 | -4.239 | -4.945 |
| ZINC000000388081 | fda.1475 | -4.239 | -4.747 |
| ZINC000004474443 | fda.510 | -4.239 | -4.521 |
| ZINC000035328014 | fda.1555 | -4.237 | -4.676 |
| ZINC000000001267 | fda.855 | -4.237 | -4.238 |
| ZINC000002522648 | fda.909 | -4.237 | -4.238 |
| ZINC000000388081 | fda.1476 | -4.237 | -4.744 |
| ZINC000003944422 | fda.127 | -4.235 | -4.235 |
| ZINC000100037020 | fda.1535 | -4.235 | -4.945 |
| ZINC000000968305 | fda.1432 | -4.234 | -4.274 |
| ZINC000003787097 | fda.555 | -4.233 | -4.299 |
| ZINC000003875392 | fda.563 | -4.233 | -4.283 |
| ZINC000003875392 | fda.564 | -4.233 | -4.283 |
| ZINC000000000751 | fda.1415 | -4.233 | -4.283 |
| ZINC000000000751 | fda.1416 | -4.233 | -4.283 |
| ZINC000003872994 | fda.217 | -4.233 | -4.558 |
| ZINC000000643153 | fda.1819 | -4.233 | -4.558 |
| ZINC000003776970 | fda.1692 | -4.232 | -4.807 |
| ZINC000030691727 | fda.1340 | -4.232 | -4.232 |
| ZINC000003589203 | fda.624 | -4.232 | -4.232 |
| ZINC000000896569 | fda.1353 | -4.231 | -4.245 |
| ZINC000068153186 | fda.212 | -4.231 | -4.647 |
| ZINC000068153186 | fda.213 | -4.231 | -4.647 |
| ZINC000003830716 | fda.1308 | -4.228 | -4.612 |
| ZINC000003830716 | fda.1309 | -4.228 | -4.612 |
| ZINC000150338699 | fda.2087 | -4.228 | -4.23 |
| ZINC000005224188 | fda.386 | -4.228 | -4.228 |
| ZINC000100036536 | fda.1297 | -4.228 | -4.229 |
| ZINC000001482094 | fda.455 | -4.225 | -4.225 |
| ZINC000001481910 | fda.794 | -4.225 | -4.225 |
| ZINC000004632106 | fda.1233 | -4.225 | -5.011 |
| ZINC000000897085 | fda.1170 | -4.222 | -4.224 |
| ZINC000000537964 | fda.2067 | -4.222 | -4.224 |
| ZINC000000266964 | fda.1479 | -4.221 | -4.221 |
| ZINC000001530638 | fda.1621 | -4.22 | -4.22 |
| ZINC000001530637 | fda.1098 | -4.22 | -4.22 |
| ZINC000000001798 | fda.1135 | -4.219 | -4.244 |
| ZINC000000001798 | fda.1136 | -4.219 | -4.244 |
| ZINC000000001798 | fda.1137 | -4.219 | -4.244 |
| ZINC000410428674 | fda.829 | -4.219 | -4.219 |
| ZINC000001533877 | fda.821 | -4.219 | -4.31 |
| ZINC000003873296 | fda.1768 | -4.218 | -4.331 |
| ZINC000003873296 | fda.1769 | -4.218 | -4.331 |
| ZINC000100037020 | fda.1533 | -4.217 | -4.43 |
| ZINC000100037020 | fda.1534 | -4.217 | -4.43 |
| ZINC000100037020 | fda.1535 | -4.217 | -4.43 |
| ZINC000003833846 | fda.94 | -4.217 | -4.231 |
| ZINC000003833846 | fda.96 | -4.217 | -4.231 |
| ZINC000003926298 | fda.1767 | -4.217 | -4.217 |
| ZINC000000020228 | fda.326 | -4.216 | -4.453 |
| ZINC000000057206 | fda.988 | -4.216 | -4.453 |
| ZINC000035801098 | fda.1547 | -4.214 | -5.995 |
| ZINC000035801098 | fda.1548 | -4.214 | -5.995 |
| ZINC000000001148 | fda.1361 | -4.212 | -4.216 |
| ZINC000000896918 | fda.1169 | -4.211 | -4.218 |
| ZINC000084441937 | fda.1909 | -4.211 | -4.236 |
| ZINC000000389149 | fda.805 | -4.211 | -4.212 |
| ZINC000000000494 | fda.1485 | -4.211 | -4.212 |
| ZINC000003830215 | fda.1245 | -4.21 | -4.403 |
| ZINC000019702309 | fda.724 | -4.209 | -4.375 |
| ZINC000008015016 | fda.1596 | -4.207 | -4.521 |
| ZINC000003830453 | fda.1695 | -4.205 | -4.205 |
| ZINC000012503177 | fda.1264 | -4.204 | -4.208 |
| ZINC000003875368 | fda.336 | -4.204 | -5.485 |
| ZINC000003875368 | fda.337 | -4.204 | -5.485 |
| ZINC000003938695 | fda.826 | -4.204 | -4.204 |
| ZINC000011615927 | fda.480 | -4.203 | -4.205 |
| ZINC000011615928 | fda.481 | -4.203 | -4.205 |
| ZINC000019594599 | fda.773 | -4.203 | -5.013 |
| ZINC000019594599 | fda.774 | -4.203 | -5.013 |
| ZINC000019594599 | fda.775 | -4.203 | -5.013 |
| ZINC000001482197 | fda.456 | -4.201 | -4.23 |
| ZINC000000896666 | fda.81 | -4.201 | -4.23 |
| ZINC000000402954 | fda.965 | -4.2 | -4.262 |
| ZINC000000402954 | fda.966 | -4.2 | -4.262 |
| ZINC000000402954 | fda.967 | -4.2 | -4.262 |
| ZINC000003872994 | fda.217 | -4.199 | -4.747 |
| ZINC000003872994 | fda.218 | -4.199 | -4.747 |
| ZINC000000643153 | fda.1819 | -4.199 | -4.747 |
| ZINC000000643153 | fda.1820 | -4.199 | -4.747 |
| ZINC000058581064 | fda.1199 | -4.198 | -4.486 |
| ZINC000001530427 | fda.1 | -4.198 | -4.451 |
| ZINC000001530427 | fda.2 | -4.197 | -4.451 |
| ZINC000001530427 | fda.3 | -4.197 | -4.451 |
| ZINC000003875483 | fda.548 | -4.197 | -4.32 |
| ZINC000003875483 | fda.549 | -4.197 | -4.32 |
| ZINC000003875483 | fda.550 | -4.197 | -4.32 |
| ZINC000003816514 | fda.1698 | -4.197 | -5.413 |
| ZINC000001842633 | fda.1429 | -4.195 | -4.196 |
| ZINC000001539579 | fda.1522 | -4.195 | -4.195 |
| ZINC000000968328 | fda.403 | -4.194 | -4.593 |
| ZINC000000968330 | fda.1173 | -4.194 | -4.593 |
| ZINC000000538273 | fda.952 | -4.194 | -4.337 |
| ZINC000000538273 | fda.953 | -4.194 | -4.337 |
| ZINC000000537891 | fda.1373 | -4.194 | -4.337 |
| ZINC000000537891 | fda.1374 | -4.194 | -4.337 |
| ZINC000000968336 | fda.1359 | -4.194 | -4.194 |
| ZINC000000020255 | fda.1422 | -4.194 | -4.194 |
| ZINC000003830999 | fda.604 | -4.194 | -4.296 |
| ZINC000003782599 | fda.392 | -4.189 | -4.189 |
| ZINC000003918453 | fda.1456 | -4.189 | -5.69 |
| ZINC000003931840 | fda.396 | -4.188 | -4.19 |
| ZINC000003876023 | fda.545 | -4.188 | -4.188 |
| ZINC000003875439 | fda.1883 | -4.188 | -4.188 |
| ZINC000095619105 | fda.1194 | -4.185 | -4.812 |
| ZINC000095619105 | fda.1195 | -4.185 | -4.812 |
| ZINC000043100953 | fda.1804 | -4.183 | -5.376 |
| ZINC000043100953 | fda.1805 | -4.183 | -5.376 |
| ZINC000003925861 | fda.1778 | -4.182 | -4.194 |
| ZINC000008034120 | fda.1565 | -4.182 | -4.191 |
| ZINC000100009383 | fda.25 | -4.182 | -4.182 |
| ZINC000000643138 | fda.1027 | -4.18 | -4.728 |
| ZINC000000643138 | fda.1028 | -4.18 | -4.728 |
| ZINC000000643143 | fda.252 | -4.18 | -4.728 |
| ZINC000000643143 | fda.253 | -4.18 | -4.728 |
| ZINC000001536779 | fda.797 | -4.179 | -4.18 |
| ZINC000003802417 | fda.2064 | -4.179 | -4.183 |
| ZINC000000004840 | fda.295 | -4.178 | -4.178 |
| ZINC000019632628 | fda.1899 | -4.176 | -4.265 |
| ZINC000019632628 | fda.1900 | -4.176 | -4.265 |
| ZINC000004393164 | fda.844 | -4.176 | -4.181 |
| ZINC000013597823 | fda.750 | -4.175 | -5.383 |
| ZINC000003875484 | fda.1003 | -4.174 | -4.174 |
| ZINC000008015016 | fda.1595 | -4.174 | -4.488 |
| ZINC000001996784 | fda.982 | -4.174 | -4.342 |
| ZINC000003927870 | fda.1491 | -4.172 | -5.321 |
| ZINC000003927870 | fda.1492 | -4.172 | -5.321 |
| ZINC000003872994 | fda.217 | -4.171 | -4.496 |
| ZINC000000643153 | fda.1819 | -4.171 | -4.496 |
| ZINC000005844788 | fda.187 | -4.171 | -4.191 |
| ZINC000003830212 | fda.1502 | -4.17 | -4.19 |
| ZINC000003830212 | fda.1503 | -4.17 | -4.19 |
| ZINC000035328014 | fda.1555 | -4.17 | -4.608 |
| ZINC000003977764 | fda.653 | -4.17 | -4.17 |
| ZINC000004097310 | fda.901 | -4.17 | -4.17 |
| ZINC000003921872 | fda.198 | -4.169 | -4.169 |
| ZINC000000537805 | fda.580 | -4.168 | -4.168 |
| ZINC000001530599 | fda.919 | -4.168 | -4.168 |
| ZINC000043100953 | fda.1804 | -4.165 | -5.358 |
| ZINC000043100953 | fda.1805 | -4.165 | -5.358 |
| ZINC000003812913 | fda.975 | -4.165 | -4.165 |
| ZINC000003831138 | fda.1007 | -4.165 | -4.165 |
| ZINC000022010379 | fda.636 | -4.165 | -4.38 |
| ZINC000022010379 | fda.638 | -4.165 | -4.38 |
| ZINC000022010375 | fda.2029 | -4.165 | -4.38 |
| ZINC000022010375 | fda.2031 | -4.165 | -4.38 |
| ZINC000012503187 | fda.568 | -4.164 | -4.556 |
| ZINC000018203737 | fda.367 | -4.164 | -4.957 |
| ZINC000018203737 | fda.368 | -4.164 | -4.957 |
| ZINC000052509366 | fda.1211 | -4.163 | -4.607 |
| ZINC000052509366 | fda.1212 | -4.163 | -4.607 |
| ZINC000003806721 | fda.1714 | -4.163 | -5.708 |
| ZINC000003806721 | fda.1715 | -4.163 | -5.708 |
| ZINC000006627681 | fda.1095 | -4.162 | -5.106 |
| ZINC000022448696 | fda.1825 | -4.161 | -4.803 |
| ZINC000022448696 | fda.1826 | -4.161 | -4.803 |
| ZINC000003812869 | fda.2047 | -4.161 | -4.794 |
| ZINC000012503177 | fda.1264 | -4.16 | -4.164 |
| ZINC000035801098 | fda.1548 | -4.16 | -4.202 |
| ZINC000000004319 | fda.317 | -4.159 | -6.097 |
| ZINC000000403010 | fda.1457 | -4.159 | -6.097 |
| ZINC000001530886 | fda.404 | -4.159 | -4.433 |
| ZINC000001530886 | fda.405 | -4.159 | -4.433 |
| ZINC000003926298 | fda.1767 | -4.159 | -4.159 |
| ZINC000000001681 | fda.1668 | -4.159 | -4.173 |
| ZINC000000001681 | fda.1669 | -4.159 | -4.173 |
| ZINC000003830993 | fda.1461 | -4.157 | -4.281 |
| ZINC000003830993 | fda.1462 | -4.157 | -4.281 |
| ZINC000001530427 | fda.2 | -4.155 | -4.408 |
| ZINC000001530427 | fda.3 | -4.155 | -4.408 |
| ZINC000003830999 | fda.605 | -4.155 | -4.257 |
| ZINC000006661227 | fda.1197 | -4.155 | -4.589 |
| ZINC000006661227 | fda.1198 | -4.155 | -4.589 |
| ZINC000000538312 | fda.840 | -4.154 | -4.164 |
| ZINC000000538312 | fda.841 | -4.154 | -4.164 |
| ZINC000004175630 | fda.948 | -4.154 | -4.161 |
| ZINC000004175630 | fda.949 | -4.154 | -4.161 |
| ZINC000006409735 | fda.1513 | -4.153 | -4.943 |
| ZINC000003806063 | fda.106 | -4.152 | -4.155 |
| ZINC000001530862 | fda.207 | -4.151 | -4.151 |
| ZINC000001530863 | fda.223 | -4.151 | -4.151 |
| ZINC000002599970 | fda.57 | -4.15 | -6.458 |
| ZINC000000897256 | fda.2010 | -4.15 | -6.458 |
| ZINC000006627681 | fda.1096 | -4.15 | -4.15 |
| ZINC000003914808 | fda.606 | -4.149 | -4.153 |
| ZINC000001846431 | fda.183 | -4.149 | -4.152 |
| ZINC000003977978 | fda.415 | -4.148 | -4.148 |
| ZINC000000020231 | fda.294 | -4.147 | -4.151 |
| ZINC000100014909 | fda.1280 | -4.147 | -4.97 |
| ZINC000100014909 | fda.1281 | -4.147 | -4.97 |
| ZINC000036701290 | fda.519 | -4.146 | -4.901 |
| ZINC000036701290 | fda.520 | -4.146 | -4.901 |
| ZINC000021982937 | fda.2024 | -4.145 | -4.325 |
| ZINC000021982937 | fda.2025 | -4.145 | -4.325 |
| ZINC000033965961 | fda.2034 | -4.145 | -4.325 |
| ZINC000033965961 | fda.2035 | -4.145 | -4.325 |
| ZINC000068204830 | fda.215 | -4.145 | -5.316 |
| ZINC000011681534 | fda.2021 | -4.142 | -6.165 |
| ZINC000060325170 | fda.1200 | -4.14 | -4.143 |
| ZINC000003830813 | fda.1652 | -4.139 | -4.779 |
| ZINC000003830813 | fda.1654 | -4.139 | -4.779 |
| ZINC000013973998 | fda.498 | -4.137 | -4.139 |
| ZINC000001554010 | fda.82 | -4.137 | -4.139 |
| ZINC000003812851 | fda.266 | -4.134 | -5.111 |
| ZINC000003812851 | fda.265 | -4.134 | -5.111 |
| ZINC000000537795 | fda.848 | -4.134 | -4.156 |
| ZINC000000537795 | fda.849 | -4.134 | -4.156 |
| ZINC000000537795 | fda.850 | -4.134 | -4.156 |
| ZINC000000538273 | fda.952 | -4.134 | -5.355 |
| ZINC000000538273 | fda.953 | -4.134 | -5.355 |
| ZINC000000537891 | fda.1373 | -4.134 | -5.355 |
| ZINC000000537891 | fda.1374 | -4.134 | -5.355 |
| ZINC000000000347 | fda.1937 | -4.134 | -4.135 |
| ZINC000000000347 | fda.1939 | -4.134 | -4.135 |
| ZINC000006661227 | fda.1196 | -4.134 | -4.568 |
| ZINC000019364222 | fda.663 | -4.133 | -4.451 |
| ZINC000019364222 | fda.664 | -4.133 | -4.451 |
| ZINC000019364222 | fda.665 | -4.133 | -4.451 |
| ZINC000019364224 | fda.666 | -4.133 | -4.451 |
| ZINC000019364224 | fda.667 | -4.133 | -4.451 |
| ZINC000019364224 | fda.668 | -4.133 | -4.451 |
| ZINC000001999441 | fda.489 | -4.132 | -4.152 |
| ZINC000003995809 | fda.1239 | -4.131 | -4.131 |
| ZINC000000001331 | fda.856 | -4.131 | -4.135 |
| ZINC000000018087 | fda.1846 | -4.131 | -4.135 |
| ZINC000001542113 | fda.329 | -4.13 | -5.225 |
| ZINC000001542113 | fda.330 | -4.13 | -5.225 |
| ZINC000100016084 | fda.515 | -4.13 | -4.13 |
| ZINC000052955754 | fda.129 | -4.129 | -4.307 |
| ZINC000052955754 | fda.130 | -4.129 | -4.307 |
| ZINC000052955754 | fda.131 | -4.129 | -4.307 |
| ZINC000100055899 | fda.1313 | -4.127 | -4.127 |
| ZINC000013986658 | fda.542 | -4.125 | -4.146 |
| ZINC000001843099 | fda.1783 | -4.125 | -4.125 |
| ZINC000003873160 | fda.1454 | -4.125 | -4.125 |
| ZINC000004658290 | fda.15 | -4.124 | -4.594 |
| ZINC000003589203 | fda.624 | -4.123 | -4.123 |
| ZINC000013986658 | fda.541 | -4.123 | -4.144 |
| ZINC000003812862 | fda.429 | -4.121 | -5.175 |
| ZINC000003812862 | fda.430 | -4.121 | -5.175 |
| ZINC000028108825 | fda.89 | -4.119 | -5.511 |
| ZINC000028108825 | fda.90 | -4.119 | -5.511 |
| ZINC000028108825 | fda.91 | -4.119 | -5.511 |
| ZINC000014961096 | fda.19 | -4.119 | -4.119 |
| ZINC000004097304 | fda.1986 | -4.118 | -4.118 |
| ZINC000004632106 | fda.1233 | -4.117 | -4.904 |
| ZINC000003830391 | fda.361 | -4.114 | -4.838 |
| ZINC000003830391 | fda.362 | -4.114 | -4.838 |
| ZINC000012503068 | fda.574 | -4.114 | -4.114 |
| ZINC000000000507 | fda.1563 | -4.113 | -5.175 |
| ZINC000000000507 | fda.1564 | -4.113 | -5.175 |
| ZINC000000403079 | fda.1677 | -4.113 | -5.175 |
| ZINC000000403079 | fda.1678 | -4.113 | -5.175 |
| ZINC000013597823 | fda.751 | -4.112 | -5.32 |
| ZINC000003805768 | fda.1741 | -4.111 | -4.112 |
| ZINC000003805768 | fda.1742 | -4.111 | -4.112 |
| ZINC000004097427 | fda.1216 | -4.11 | -4.976 |
| ZINC000028957444 | fda.1332 | -4.109 | -4.64 |
| ZINC000022010379 | fda.637 | -4.108 | -4.323 |
| ZINC000022010375 | fda.2030 | -4.108 | -4.323 |
| ZINC000003805768 | fda.1741 | -4.108 | -4.11 |
| ZINC000003805768 | fda.1742 | -4.108 | -4.11 |
| ZINC000000968264 | fda.1644 | -4.107 | -4.116 |
| ZINC000000968264 | fda.1645 | -4.107 | -4.116 |
| ZINC000096942202 | fda.1307 | -4.107 | -4.107 |
| ZINC000096942201 | fda.1329 | -4.107 | -4.107 |
| ZINC000000057340 | fda.1074 | -4.106 | -4.106 |
| ZINC000000057341 | fda.1764 | -4.106 | -4.106 |
| ZINC000003794601 | fda.178 | -4.106 | -4.108 |
| ZINC000003800706 | fda.260 | -4.106 | -4.108 |
| ZINC000003830999 | fda.605 | -4.106 | -4.209 |
| ZINC000000622123 | fda.1786 | -4.106 | -4.106 |
| ZINC000000009342 | fda.314 | -4.106 | -4.106 |
| ZINC000000009342 | fda.315 | -4.106 | -4.106 |
| ZINC000002525885 | fda.878 | -4.106 | -4.106 |
| ZINC000002525885 | fda.879 | -4.106 | -4.106 |
| ZINC000019166988 | fda.1574 | -4.106 | -4.616 |
| ZINC000003830813 | fda.1653 | -4.105 | -4.744 |
| ZINC000009212654 | fda.109 | -4.101 | -4.105 |
| ZINC000004095858 | fda.250 | -4.1 | -4.1 |
| ZINC000000007601 | fda.1591 | -4.1 | -4.1 |
| ZINC000000020230 | fda.1608 | -4.1 | -4.1 |
| ZINC000005752191 | fda.1032 | -4.099 | -4.099 |
| ZINC000003915154 | fda.1004 | -4.099 | -4.099 |
| ZINC000014261579 | fda.1519 | -4.099 | -4.099 |
| ZINC000003978006 | fda.1890 | -4.098 | -4.098 |
| ZINC000000491073 | fda.977 | -4.096 | -6.212 |
| ZINC000000491073 | fda.978 | -4.096 | -6.212 |
| ZINC000000491073 | fda.979 | -4.096 | -6.212 |
| ZINC000003920027 | fda.1471 | -4.096 | -4.096 |
| ZINC000001489478 | fda.458 | -4.095 | -4.098 |
| ZINC000018089317 | fda.719 | -4.095 | -4.095 |
| ZINC000001611274 | fda.585 | -4.095 | -4.926 |
| ZINC000001611274 | fda.586 | -4.095 | -4.926 |
| ZINC000009212428 | fda.269 | -4.093 | -4.093 |
| ZINC000009212427 | fda.1350 | -4.093 | -4.093 |
| ZINC000058581064 | fda.1199 | -4.093 | -6.061 |
| ZINC000000113404 | fda.1773 | -4.092 | -4.092 |
| ZINC000000113410 | fda.1774 | -4.092 | -4.092 |
| ZINC000018516586 | fda.427 | -4.091 | -4.962 |
| ZINC000001846431 | fda.183 | -4.09 | -4.093 |
| ZINC000003977777 | fda.1277 | -4.09 | -4.09 |
| ZINC000003798537 | fda.2062 | -4.089 | -4.098 |
| ZINC000004632106 | fda.1232 | -4.089 | -4.876 |
| ZINC000001530713 | fda.789 | -4.088 | -4.295 |
| ZINC000001542392 | fda.140 | -4.088 | -4.088 |
| ZINC000001843099 | fda.1781 | -4.088 | -4.088 |
| ZINC000004212851 | fda.994 | -4.088 | -4.088 |
| ZINC000001530930 | fda.1952 | -4.086 | -5.499 |
| ZINC000001530930 | fda.1953 | -4.086 | -5.499 |
| ZINC000003876069 | fda.1978 | -4.086 | -5.499 |
| ZINC000003876069 | fda.1979 | -4.086 | -5.499 |
| ZINC000001851149 | fda.1784 | -4.086 | -4.279 |
| ZINC000001851149 | fda.1785 | -4.086 | -4.279 |
| ZINC000001530930 | fda.1952 | -4.085 | -5.497 |
| ZINC000001530930 | fda.1953 | -4.085 | -5.497 |
| ZINC000003876069 | fda.1978 | -4.085 | -5.497 |
| ZINC000003876069 | fda.1979 | -4.085 | -5.497 |
| ZINC000012468792 | fda.1445 | -4.084 | -4.084 |
| ZINC000022065398 | fda.346 | -4.084 | -4.084 |
| ZINC000004641374 | fda.340 | -4.084 | -4.084 |
| ZINC000000121541 | fda.1517 | -4.083 | -4.736 |
| ZINC000003920719 | fda.384 | -4.081 | -4.084 |
| ZINC000011615926 | fda.478 | -4.081 | -4.084 |
| ZINC000029571072 | fda.2032 | -4.081 | -4.585 |
| ZINC000049637509 | fda.2041 | -4.081 | -4.585 |
| ZINC000003916214 | fda.1980 | -4.081 | -4.263 |
| ZINC000003916214 | fda.1981 | -4.081 | -4.263 |
| ZINC000051951647 | fda.1090 | -4.079 | -4.107 |
| ZINC000001530697 | fda.692 | -4.078 | -4.079 |
| ZINC000001530695 | fda.1627 | -4.078 | -4.079 |
| ZINC000001530695 | fda.1628 | -4.078 | -4.079 |
| ZINC000096014710 | fda.1481 | -4.077 | -4.081 |
| ZINC000000009689 | fda.1298 | -4.076 | -4.076 |
| ZINC000003812983 | fda.1482 | -4.075 | -4.134 |
| ZINC000003812983 | fda.1483 | -4.075 | -4.134 |
| ZINC000003938704 | fda.156 | -4.072 | -4.099 |
| ZINC000003938704 | fda.157 | -4.072 | -4.099 |
| ZINC000001530938 | fda.1954 | -4.07 | -4.092 |
| ZINC000001530938 | fda.1955 | -4.07 | -4.092 |
| ZINC000003831490 | fda.375 | -4.069 | -4.29 |
| ZINC000003830891 | fda.239 | -4.066 | -4.073 |
| ZINC000000004319 | fda.318 | -4.065 | -6.003 |
| ZINC000000403010 | fda.1458 | -4.065 | -6.003 |
| ZINC000003875483 | fda.548 | -4.064 | -4.187 |
| ZINC000003875483 | fda.549 | -4.064 | -4.187 |
| ZINC000003875483 | fda.550 | -4.064 | -4.187 |
| ZINC000003831417 | fda.1465 | -4.063 | -4.063 |
| ZINC000000901061 | fda.1058 | -4.061 | -4.061 |
| ZINC000003830986 | fda.1012 | -4.061 | -6.094 |
| ZINC000000599734 | fda.1496 | -4.061 | -6.094 |
| ZINC000004658603 | fda.85 | -4.061 | -4.064 |
| ZINC000004658603 | fda.86 | -4.061 | -4.064 |
| ZINC000043100709 | fda.1803 | -4.06 | -4.06 |
| ZINC000004632106 | fda.1232 | -4.059 | -4.846 |
| ZINC000034676245 | fda.285 | -4.056 | -4.693 |
| ZINC000034676245 | fda.286 | -4.056 | -4.693 |
| ZINC000034676245 | fda.287 | -4.056 | -4.693 |
| ZINC000410428674 | fda.829 | -4.055 | -4.055 |
| ZINC000003830391 | fda.361 | -4.055 | -4.779 |
| ZINC000003781943 | fda.1693 | -4.054 | -4.411 |
| ZINC000003797541 | fda.1681 | -4.053 | -4.074 |
| ZINC000019362735 | fda.657 | -4.053 | -4.521 |
| ZINC000019362735 | fda.658 | -4.053 | -4.521 |
| ZINC000019362735 | fda.659 | -4.053 | -4.521 |
| ZINC000019362737 | fda.660 | -4.053 | -4.521 |
| ZINC000019362737 | fda.661 | -4.053 | -4.521 |
| ZINC000019362737 | fda.662 | -4.053 | -4.521 |
| ZINC000003927200 | fda.97 | -4.052 | -4.052 |
| ZINC000169621220 | fda.275 | -4.051 | -4.083 |
| ZINC000001530427 | fda.1 | -4.051 | -4.304 |
| ZINC000000001370 | fda.857 | -4.05 | -4.051 |
| ZINC000000057278 | fda.989 | -4.05 | -4.05 |
| ZINC000000003911 | fda.1680 | -4.05 | -4.05 |
| ZINC000022010382 | fda.639 | -4.049 | -4.755 |
| ZINC000022010382 | fda.640 | -4.049 | -4.755 |
| ZINC000022010382 | fda.641 | -4.049 | -4.755 |
| ZINC000022010387 | fda.2101 | -4.049 | -4.755 |
| ZINC000022010387 | fda.2102 | -4.049 | -4.755 |
| ZINC000022010387 | fda.2103 | -4.049 | -4.755 |
| ZINC000000057464 | fda.1076 | -4.049 | -4.527 |
| ZINC000001543916 | fda.1855 | -4.048 | -4.757 |
| ZINC000003915154 | fda.1004 | -4.046 | -4.046 |
| ZINC000014261579 | fda.1519 | -4.046 | -4.046 |
| ZINC000000057464 | fda.1076 | -4.045 | -4.781 |
| ZINC000003813010 | fda.104 | -4.045 | -5.831 |
| ZINC000003813010 | fda.105 | -4.045 | -5.831 |
| ZINC000003830339 | fda.1639 | -4.043 | -4.045 |
| ZINC000001853550 | fda.1688 | -4.043 | -4.055 |
| ZINC000002005550 | fda.154 | -4.041 | -4.138 |
| ZINC000003874950 | fda.229 | -4.041 | -4.138 |
| ZINC000003831040 | fda.621 | -4.041 | -4.043 |
| ZINC000100378061 | fda.531 | -4.041 | -4.664 |
| ZINC000009212428 | fda.271 | -4.04 | -4.041 |
| ZINC000009212427 | fda.1352 | -4.04 | -4.041 |
| ZINC000003830339 | fda.1639 | -4.04 | -4.041 |
| ZINC000003781664 | fda.1707 | -4.04 | -4.04 |
| ZINC000001530652 | fda.459 | -4.039 | -4.089 |
| ZINC000001530652 | fda.460 | -4.039 | -4.089 |
| ZINC000019362735 | fda.657 | -4.038 | -4.506 |
| ZINC000019362735 | fda.658 | -4.038 | -4.506 |
| ZINC000019362735 | fda.659 | -4.038 | -4.506 |
| ZINC000019362737 | fda.660 | -4.038 | -4.506 |
| ZINC000019362737 | fda.661 | -4.038 | -4.506 |
| ZINC000019362737 | fda.662 | -4.038 | -4.506 |
| ZINC000096942202 | fda.1307 | -4.037 | -4.037 |
| ZINC000096942201 | fda.1329 | -4.037 | -4.037 |
| ZINC000012503099 | fda.576 | -4.035 | -4.038 |
| ZINC000012503099 | fda.577 | -4.035 | -4.038 |
| ZINC000052509366 | fda.1211 | -4.035 | -4.414 |
| ZINC000000020240 | fda.1542 | -4.035 | -4.036 |
| ZINC000000056556 | fda.1722 | -4.035 | -4.036 |
| ZINC000003916214 | fda.1980 | -4.034 | -4.937 |
| ZINC000003916214 | fda.1981 | -4.034 | -4.937 |
| ZINC000100009280 | fda.1243 | -4.034 | -4.036 |
| ZINC000000537791 | fda.1369 | -4.033 | -4.033 |
| ZINC000030691736 | fda.1341 | -4.033 | -4.033 |
| ZINC000008214614 | fda.1562 | -4.033 | -4.033 |
| ZINC000018089317 | fda.719 | -4.032 | -4.032 |
| ZINC000002005550 | fda.155 | -4.032 | -4.129 |
| ZINC000003874950 | fda.230 | -4.032 | -4.129 |
| ZINC000100009280 | fda.1244 | -4.03 | -4.033 |
| ZINC000000057624 | fda.630 | -4.029 | -6.124 |
| ZINC000027428713 | fda.1301 | -4.026 | -4.026 |
| ZINC000008214619 | fda.827 | -4.026 | -4.026 |
| ZINC000094566093 | fda.1303 | -4.023 | -4.153 |
| ZINC000094566092 | fda.1315 | -4.023 | -4.153 |
| ZINC000019144226 | fda.1589 | -4.023 | -4.023 |
| ZINC000019144231 | fda.1864 | -4.023 | -4.023 |
| ZINC000003842753 | fda.1734 | -4.022 | -5.217 |
| ZINC000003842753 | fda.1735 | -4.022 | -5.217 |
| ZINC000013648755 | fda.417 | -4.022 | -5.063 |
| ZINC000001482094 | fda.455 | -4.021 | -4.021 |
| ZINC000001481910 | fda.794 | -4.021 | -4.021 |
| ZINC000004693575 | fda.323 | -4.021 | -5.416 |
| ZINC000004693574 | fda.1217 | -4.021 | -5.416 |
| ZINC000001530599 | fda.920 | -4.02 | -4.027 |
| ZINC000001530599 | fda.921 | -4.02 | -4.027 |
| ZINC000001530599 | fda.922 | -4.02 | -4.027 |
| ZINC000000896663 | fda.487 | -4.02 | -4.049 |
| ZINC000001639567 | fda.1646 | -4.02 | -4.049 |
| ZINC000000968305 | fda.1433 | -4.019 | -4.059 |
| ZINC000008101126 | fda.1346 | -4.017 | -4.017 |
| ZINC000016159083 | fda.169 | -4.014 | -4.017 |
| ZINC000016159083 | fda.170 | -4.014 | -4.017 |
| ZINC000000601250 | fda.1505 | -4.014 | -4.345 |
| ZINC000003873371 | fda.1092 | -4.014 | -4.345 |
| ZINC000003873371 | fda.1093 | -4.014 | -4.345 |
| ZINC000000057512 | fda.1014 | -4.012 | -4.012 |
| ZINC000000968305 | fda.1432 | -4.012 | -4.052 |
| ZINC000001530974 | fda.689 | -4.012 | -4.456 |
| ZINC000001530974 | fda.690 | -4.012 | -4.456 |
| ZINC000001530973 | fda.883 | -4.012 | -4.456 |
| ZINC000001530973 | fda.884 | -4.012 | -4.456 |
| ZINC000003792789 | fda.602 | -4.011 | -4.015 |
| ZINC000019594557 | fda.755 | -4.01 | -4.825 |
| ZINC000019594557 | fda.756 | -4.01 | -4.825 |
| ZINC000019361042 | fda.762 | -4.01 | -4.825 |
| ZINC000019361042 | fda.763 | -4.01 | -4.825 |
| ZINC000000000711 | fda.1940 | -4.01 | -4.02 |
| ZINC000001843047 | fda.1964 | -4.01 | -4.02 |
| ZINC000000000096 | fda.946 | -4.009 | -4.009 |
| ZINC000000896455 | fda.1452 | -4.009 | -4.009 |
| ZINC000013537284 | fda.749 | -4.009 | -4.912 |
| ZINC000002599970 | fda.56 | -4.007 | -6.315 |
| ZINC000000897256 | fda.2009 | -4.007 | -6.315 |
| ZINC000001530579 | fda.1087 | -4.006 | -4.05 |
| ZINC000001530580 | fda.1089 | -4.006 | -4.05 |
| ZINC000003812888 | fda.1683 | -4.006 | -4.011 |
| ZINC000003812888 | fda.1684 | -4.006 | -4.011 |
| ZINC000003992105 | fda.2014 | -4.006 | -4.006 |
| ZINC000000897385 | fda.1426 | -4.006 | -4.62 |
| ZINC000000897385 | fda.1427 | -4.006 | -4.62 |
| ZINC000000608101 | fda.1228 | -4.006 | -4.62 |
| ZINC000000608101 | fda.1229 | -4.006 | -4.62 |
| ZINC000002539827 | fda.1383 | -4.006 | -6.033 |
| ZINC000002539827 | fda.1384 | -4.006 | -6.033 |
| ZINC000002539827 | fda.1385 | -4.006 | -6.033 |
| ZINC000000895154 | fda.1406 | -4.006 | -6.033 |
| ZINC000000895154 | fda.1407 | -4.006 | -6.033 |
| ZINC000000895154 | fda.1408 | -4.006 | -6.033 |
| ZINC000001535101 | fda.1667 | -4.003 | -4.005 |
| ZINC000003830716 | fda.1308 | -4.003 | -4.442 |
| ZINC000003830716 | fda.1309 | -4.003 | -4.442 |
| ZINC000005029557 | fda.1064 | -4.003 | -4.003 |
| ZINC000011616925 | fda.144 | -4.002 | -4.044 |
| ZINC000001536109 | fda.221 | -4.002 | -4.044 |
| ZINC000000121541 | fda.1517 | -4.002 | -4.587 |
| ZINC000018516586 | fda.428 | -4.002 | -4.423 |
| ZINC000008034121 | fda.1358 | -4.001 | -4.023 |
| ZINC000026664090 | fda.351 | -4 | -6.26 |
| ZINC000026664090 | fda.352 | -4 | -6.26 |
| ZINC000026664090 | fda.353 | -4 | -6.26 |
| ZINC000028957444 | fda.1332 | -4 | -4.531 |
| ZINC000003842753 | fda.1734 | -4 | -5.194 |
| ZINC000003842753 | fda.1735 | -4 | -5.194 |
| ZINC000253476027 | fda.497 | -4 | -4 |
| ZINC000100015775 | fda.1282 | -4 | -4 |
| ZINC000001543916 | fda.1856 | -3.999 | -4.212 |
| ZINC000003871832 | fda.1727 | -3.997 | -4.002 |
| ZINC000003871832 | fda.1728 | -3.997 | -4.002 |
| ZINC000019796080 | fda.714 | -3.997 | -5.109 |
| ZINC000019796080 | fda.715 | -3.997 | -5.109 |
| ZINC000019796080 | fda.716 | -3.997 | -5.109 |
| ZINC000012503156 | fda.512 | -3.997 | -3.997 |
| ZINC000100061056 | fda.192 | -3.997 | -3.997 |
| ZINC000000001773 | fda.1842 | -3.997 | -4.05 |
| ZINC000001530760 | fda.808 | -3.996 | -4 |
| ZINC000001530759 | fda.894 | -3.996 | -4 |
| ZINC000001611274 | fda.585 | -3.995 | -5.273 |
| ZINC000001530981 | fda.204 | -3.995 | -4.116 |
| ZINC000001530981 | fda.205 | -3.995 | -4.116 |
| ZINC000001530981 | fda.206 | -3.995 | -4.116 |
| ZINC000100009278 | fda.1241 | -3.995 | -3.997 |
| ZINC000001530981 | fda.204 | -3.993 | -4.114 |
| ZINC000001530981 | fda.205 | -3.993 | -4.114 |
| ZINC000001530981 | fda.206 | -3.993 | -4.114 |
| ZINC000013682481 | fda.752 | -3.993 | -4.949 |
| ZINC000001530713 | fda.788 | -3.992 | -4.198 |
| ZINC000003776970 | fda.1691 | -3.992 | -4.567 |
| ZINC000003201907 | fda.2051 | -3.991 | -3.995 |
| ZINC000014210876 | fda.1797 | -3.989 | -4.717 |
| ZINC000003929508 | fda.1436 | -3.989 | -5.201 |
| ZINC000000057513 | fda.1946 | -3.989 | -3.993 |
| ZINC000000057513 | fda.1947 | -3.989 | -3.993 |
| ZINC000014879972 | fda.731 | -3.986 | -4.069 |
| ZINC000001530713 | fda.789 | -3.986 | -4.711 |
| ZINC000000006427 | fda.815 | -3.984 | -3.984 |
| ZINC000043100953 | fda.1806 | -3.984 | -5.176 |
| ZINC000014210457 | fda.730 | -3.983 | -3.983 |
| ZINC000034676245 | fda.285 | -3.982 | -4.619 |
| ZINC000034676245 | fda.286 | -3.982 | -4.619 |
| ZINC000034676245 | fda.287 | -3.982 | -4.619 |
| ZINC000001530621 | fda.813 | -3.981 | -4.045 |
| ZINC000004428529 | fda.1382 | -3.981 | -3.981 |
| ZINC000003809490 | fda.238 | -3.98 | -3.982 |
| ZINC000019228902 | fda.1575 | -3.979 | -4.526 |
| ZINC000019228902 | fda.1576 | -3.979 | -4.526 |
| ZINC000019228902 | fda.1577 | -3.979 | -4.526 |
| ZINC000000056427 | fda.1069 | -3.978 | -3.978 |
| ZINC000001554588 | fda.118 | -3.975 | -3.975 |
| ZINC000095619105 | fda.1193 | -3.974 | -4.226 |
| ZINC000095619105 | fda.1194 | -3.974 | -4.226 |
| ZINC000095619105 | fda.1195 | -3.974 | -4.226 |
| ZINC000003873295 | fda.494 | -3.973 | -5.011 |
| ZINC000001853205 | fda.1750 | -3.97 | -4.175 |
| ZINC000003776970 | fda.1691 | -3.969 | -4.544 |
| ZINC000029416466 | fda.158 | -3.969 | -6.229 |
| ZINC000029416466 | fda.159 | -3.969 | -6.229 |
| ZINC000029416466 | fda.160 | -3.969 | -6.229 |
| ZINC000003826253 | fda.2057 | -3.967 | -4.065 |
| ZINC000004632106 | fda.1233 | -3.967 | -4.685 |
| ZINC000004099200 | fda.835 | -3.966 | -5.589 |
| ZINC000004676424 | fda.880 | -3.966 | -5.589 |
| ZINC000000097996 | fda.1948 | -3.966 | -4.376 |
| ZINC000094566092 | fda.1316 | -3.964 | -6.343 |
| ZINC000003791297 | fda.1651 | -3.964 | -4.715 |
| ZINC000008015016 | fda.1597 | -3.964 | -4.49 |
| ZINC000004212854 | fda.833 | -3.963 | -3.963 |
| ZINC000003876023 | fda.545 | -3.963 | -3.963 |
| ZINC000001530621 | fda.812 | -3.962 | -4.026 |
| ZINC000003831405 | fda.28 | -3.962 | -3.977 |
| ZINC000003831405 | fda.29 | -3.962 | -3.977 |
| ZINC000049036447 | fda.1202 | -3.961 | -3.961 |
| ZINC000003801919 | fda.1468 | -3.96 | -4.159 |
| ZINC000000537752 | fda.845 | -3.958 | -3.97 |
| ZINC000000537752 | fda.846 | -3.958 | -3.97 |
| ZINC000022448696 | fda.1823 | -3.957 | -5.103 |
| ZINC000022448696 | fda.1824 | -3.957 | -5.103 |
| ZINC000022448696 | fda.1825 | -3.957 | -5.103 |
| ZINC000022448696 | fda.1826 | -3.957 | -5.103 |
| ZINC000022448696 | fda.1827 | -3.957 | -5.103 |
| ZINC000084441937 | fda.1909 | -3.956 | -3.981 |
| ZINC000019796018 | fda.742 | -3.956 | -4.565 |
| ZINC000019796018 | fda.743 | -3.956 | -4.565 |
| ZINC000019796018 | fda.744 | -3.956 | -4.565 |
| ZINC000003982483 | fda.1891 | -3.956 | -3.966 |
| ZINC000003982483 | fda.1893 | -3.956 | -3.966 |
| ZINC000001543916 | fda.1856 | -3.956 | -4.664 |
| ZINC000030691754 | fda.1997 | -3.955 | -3.956 |
| ZINC000003978005 | fda.1235 | -3.954 | -5.68 |
| ZINC000003978005 | fda.1236 | -3.954 | -5.68 |
| ZINC000003978005 | fda.1237 | -3.954 | -5.68 |
| ZINC000000266964 | fda.1479 | -3.953 | -3.953 |
| ZINC000000538627 | fda.863 | -3.952 | -3.952 |
| ZINC000008015016 | fda.1595 | -3.951 | -4.477 |
| ZINC000002016037 | fda.656 | -3.946 | -4.206 |
| ZINC000000606383 | fda.1227 | -3.946 | -4.206 |
| ZINC000003985982 | fda.1278 | -3.946 | -3.946 |
| ZINC000003981610 | fda.1001 | -3.945 | -3.945 |
| ZINC000005819214 | fda.1299 | -3.945 | -3.945 |
| ZINC000000895032 | fda.1381 | -3.941 | -3.941 |
| ZINC000003872994 | fda.217 | -3.941 | -4.489 |
| ZINC000003872994 | fda.218 | -3.941 | -4.489 |
| ZINC000000643153 | fda.1819 | -3.941 | -4.489 |
| ZINC000000643153 | fda.1820 | -3.941 | -4.489 |
| ZINC000001530579 | fda.1086 | -3.938 | -3.981 |
| ZINC000001530580 | fda.1088 | -3.938 | -3.981 |
| ZINC000095619101 | fda.1305 | -3.938 | -3.968 |
| ZINC000095619101 | fda.1306 | -3.938 | -3.968 |
| ZINC000095619100 | fda.1327 | -3.938 | -3.968 |
| ZINC000095619100 | fda.1328 | -3.938 | -3.968 |
| ZINC000003806104 | fda.2046 | -3.937 | -3.937 |
| ZINC000012468792 | fda.1445 | -3.936 | -3.936 |
| ZINC000000508068 | fda.976 | -3.936 | -3.936 |
| ZINC000000000607 | fda.1801 | -3.936 | -3.936 |
| ZINC000001530939 | fda.927 | -3.936 | -3.94 |
| ZINC000000601316 | fda.1225 | -3.935 | -3.935 |
| ZINC000003830579 | fda.1687 | -3.935 | -3.935 |
| ZINC000085537017 | fda.2090 | -3.934 | -6.16 |
| ZINC000085537017 | fda.2091 | -3.934 | -6.16 |
| ZINC000001542199 | fda.517 | -3.933 | -3.933 |
| ZINC000003794601 | fda.178 | -3.932 | -3.934 |
| ZINC000003800706 | fda.260 | -3.932 | -3.934 |
| ZINC000000001408 | fda.1560 | -3.931 | -3.942 |
| ZINC000001552908 | fda.1034 | -3.931 | -3.933 |
| ZINC000022056030 | fda.145 | -3.931 | -4.032 |
| ZINC000022056030 | fda.146 | -3.931 | -4.032 |
| ZINC000022056030 | fda.147 | -3.931 | -4.032 |
| ZINC000019632633 | fda.224 | -3.931 | -4.032 |
| ZINC000019632633 | fda.225 | -3.931 | -4.032 |
| ZINC000019632633 | fda.226 | -3.931 | -4.032 |
| ZINC000004228258 | fda.1061 | -3.93 | -5.957 |
| ZINC000004228258 | fda.1062 | -3.93 | -5.957 |
| ZINC000004228258 | fda.1063 | -3.93 | -5.957 |
| ZINC000004228257 | fda.257 | -3.93 | -5.957 |
| ZINC000004228257 | fda.258 | -3.93 | -5.957 |
| ZINC000004228257 | fda.259 | -3.93 | -5.957 |
| ZINC000003800008 | fda.193 | -3.93 | -4.598 |
| ZINC000003800008 | fda.194 | -3.93 | -4.598 |
| ZINC000003800008 | fda.195 | -3.93 | -4.598 |
| ZINC000000056646 | fda.986 | -3.929 | -3.931 |
| ZINC000000056645 | fda.1270 | -3.929 | -3.931 |
| ZINC000000014037 | fda.1602 | -3.929 | -3.93 |
| ZINC000026011099 | fda.39 | -3.927 | -4.04 |
| ZINC000026011099 | fda.40 | -3.927 | -4.04 |
| ZINC000000389747 | fda.32 | -3.926 | -5.013 |
| ZINC000000389747 | fda.33 | -3.926 | -5.013 |
| ZINC000000389747 | fda.34 | -3.926 | -5.013 |
| ZINC000004474443 | fda.510 | -3.926 | -4.501 |
| ZINC000014210876 | fda.1797 | -3.926 | -5.199 |
| ZINC000095564694 | fda.2095 | -3.925 | -4.011 |
| ZINC000095564694 | fda.2096 | -3.925 | -4.011 |
| ZINC000095564694 | fda.2097 | -3.925 | -4.011 |
| ZINC000000000506 | fda.290 | -3.924 | -3.925 |
| ZINC000003920266 | fda.1888 | -3.923 | -3.95 |
| ZINC000000057464 | fda.1075 | -3.922 | -5.039 |
| ZINC000001530912 | fda.809 | -3.922 | -4.216 |
| ZINC000001530912 | fda.810 | -3.922 | -4.216 |
| ZINC000001530912 | fda.811 | -3.922 | -4.216 |
| ZINC000000895103 | fda.613 | -3.922 | -3.922 |
| ZINC000001530775 | fda.887 | -3.921 | -3.921 |
| ZINC000022010379 | fda.636 | -3.921 | -4.135 |
| ZINC000022010379 | fda.638 | -3.921 | -4.135 |
| ZINC000022010375 | fda.2029 | -3.921 | -4.135 |
| ZINC000022010375 | fda.2031 | -3.921 | -4.135 |
| ZINC000003830500 | fda.595 | -3.921 | -4.559 |
| ZINC000000391812 | fda.1054 | -3.92 | -3.945 |
| ZINC000000391812 | fda.1055 | -3.92 | -3.945 |
| ZINC000000391812 | fda.1056 | -3.92 | -3.945 |
| ZINC000003929508 | fda.1436 | -3.92 | -4.002 |
| ZINC000003955219 | fda.650 | -3.919 | -3.919 |
| ZINC000095616599 | fda.1288 | -3.917 | -4.04 |
| ZINC000095616600 | fda.1325 | -3.917 | -4.04 |
| ZINC000000643114 | fda.1026 | -3.915 | -3.918 |
| ZINC000000601305 | fda.1613 | -3.915 | -3.918 |
| ZINC000012503291 | fda.137 | -3.914 | -3.914 |
| ZINC000000006157 | fda.814 | -3.913 | -5.557 |
| ZINC000001999487 | fda.1757 | -3.913 | -5.557 |
| ZINC000018516586 | fda.427 | -3.912 | -4.073 |
| ZINC000034781704 | fda.1192 | -3.911 | -3.933 |
| ZINC000008015016 | fda.1596 | -3.91 | -4.437 |
| ZINC000003830500 | fda.596 | -3.908 | -4.546 |
| ZINC000028957444 | fda.1332 | -3.906 | -4.218 |
| ZINC000001995484 | fda.1276 | -3.906 | -4.615 |
| ZINC000000002216 | fda.569 | -3.905 | -4.198 |
| ZINC000000002216 | fda.570 | -3.905 | -4.198 |
| ZINC000003830218 | fda.282 | -3.904 | -4.58 |
| ZINC000000113404 | fda.1773 | -3.902 | -3.902 |
| ZINC000000113410 | fda.1774 | -3.902 | -3.902 |
| ZINC000003831490 | fda.374 | -3.902 | -4.826 |
| ZINC000003831490 | fda.375 | -3.902 | -4.826 |
| ZINC000095616599 | fda.1288 | -3.902 | -4.024 |
| ZINC000095616600 | fda.1325 | -3.902 | -4.024 |
| ZINC000012503187 | fda.567 | -3.901 | -4.293 |
| ZINC000008577218 | fda.251 | -3.901 | -5.02 |
| ZINC000000005823 | fda.42 | -3.9 | -4.311 |
| ZINC000000005823 | fda.43 | -3.9 | -4.311 |
| ZINC000003802417 | fda.2063 | -3.9 | -3.905 |
| ZINC000008577218 | fda.251 | -3.898 | -4.178 |
| ZINC000003920266 | fda.1887 | -3.897 | -3.924 |
| ZINC000003929022 | fda.508 | -3.895 | -3.895 |
| ZINC000001543916 | fda.1855 | -3.893 | -4.602 |
| ZINC000022010379 | fda.636 | -3.892 | -4.598 |
| ZINC000022010379 | fda.637 | -3.892 | -4.598 |
| ZINC000022010379 | fda.638 | -3.892 | -4.598 |
| ZINC000022010375 | fda.2029 | -3.892 | -4.598 |
| ZINC000022010375 | fda.2030 | -3.892 | -4.598 |
| ZINC000022010375 | fda.2031 | -3.892 | -4.598 |
| ZINC000003079340 | fda.1470 | -3.892 | -3.892 |
| ZINC000003830215 | fda.1246 | -3.891 | -4.656 |
| ZINC000000968375 | fda.506 | -3.889 | -3.889 |
| ZINC000003812841 | fda.1716 | -3.889 | -3.889 |
| ZINC000000508068 | fda.976 | -3.889 | -3.889 |
| ZINC000000000607 | fda.1801 | -3.889 | -3.889 |
| ZINC000022002214 | fda.631 | -3.888 | -5.353 |
| ZINC000022002214 | fda.632 | -3.888 | -5.353 |
| ZINC000022002218 | fda.633 | -3.888 | -5.353 |
| ZINC000022002218 | fda.634 | -3.888 | -5.353 |
| ZINC000001532526 | fda.620 | -3.885 | -3.885 |
| ZINC000022059926 | fda.645 | -3.884 | -5.488 |
| ZINC000022059930 | fda.1165 | -3.884 | -5.488 |
| ZINC000066166864 | fda.443 | -3.884 | -4.036 |
| ZINC000066166864 | fda.444 | -3.884 | -4.036 |
| ZINC000100299039 | fda.1965 | -3.883 | -4.294 |
| ZINC000022448696 | fda.1825 | -3.882 | -4.272 |
| ZINC000022448696 | fda.1826 | -3.882 | -4.272 |
| ZINC000000000122 | fda.1122 | -3.881 | -3.887 |
| ZINC000000000122 | fda.1123 | -3.881 | -3.887 |
| ZINC000000002043 | fda.1799 | -3.881 | -3.887 |
| ZINC000000002043 | fda.1800 | -3.881 | -3.887 |
| ZINC000100036536 | fda.1296 | -3.879 | -3.881 |
| ZINC000003976838 | fda.1761 | -3.878 | -4.78 |
| ZINC000003976838 | fda.1762 | -3.878 | -4.78 |
| ZINC000003986735 | fda.992 | -3.877 | -4.311 |
| ZINC000000075008 | fda.674 | -3.875 | -4.63 |
| ZINC000000075008 | fda.675 | -3.875 | -4.63 |
| ZINC000000968336 | fda.1359 | -3.875 | -3.875 |
| ZINC000001543916 | fda.1856 | -3.873 | -4.582 |
| ZINC000085537053 | fda.904 | -3.87 | -3.872 |
| ZINC000001543916 | fda.1855 | -3.87 | -4.083 |
| ZINC000000004351 | fda.1723 | -3.869 | -3.876 |
| ZINC000000004351 | fda.1724 | -3.869 | -3.876 |
| ZINC000000538621 | fda.853 | -3.868 | -4.982 |
| ZINC000013597823 | fda.750 | -3.868 | -5.076 |
| ZINC000013597823 | fda.751 | -3.868 | -5.076 |
| ZINC000049841054 | fda.1203 | -3.867 | -3.87 |
| ZINC000002016037 | fda.655 | -3.864 | -4.479 |
| ZINC000002016037 | fda.656 | -3.864 | -4.479 |
| ZINC000000606383 | fda.1226 | -3.864 | -4.479 |
| ZINC000000606383 | fda.1227 | -3.864 | -4.479 |
| ZINC000001611274 | fda.586 | -3.864 | -5.141 |
| ZINC000003800008 | fda.193 | -3.864 | -4.097 |
| ZINC000003800008 | fda.194 | -3.864 | -4.097 |
| ZINC000003800008 | fda.195 | -3.864 | -4.097 |
| ZINC000018203737 | fda.367 | -3.863 | -5.396 |
| ZINC000018203737 | fda.368 | -3.863 | -5.396 |
| ZINC000003645145 | fda.1251 | -3.863 | -3.863 |
| ZINC000012358719 | fda.426 | -3.863 | -3.863 |
| ZINC000007997897 | fda.1128 | -3.863 | -5.092 |
| ZINC000007997897 | fda.1129 | -3.863 | -5.092 |
| ZINC000007997897 | fda.1130 | -3.863 | -5.092 |
| ZINC000100037020 | fda.1533 | -3.862 | -4.075 |
| ZINC000100037020 | fda.1534 | -3.862 | -4.075 |
| ZINC000100037020 | fda.1535 | -3.862 | -4.075 |
| ZINC000000643138 | fda.1028 | -3.862 | -4.187 |
| ZINC000000643143 | fda.253 | -3.862 | -4.187 |
| ZINC000000000122 | fda.1122 | -3.862 | -3.868 |
| ZINC000000000122 | fda.1123 | -3.862 | -3.868 |
| ZINC000000002043 | fda.1799 | -3.862 | -3.868 |
| ZINC000000002043 | fda.1800 | -3.862 | -3.868 |
| ZINC000014210876 | fda.1797 | -3.861 | -4.588 |
| ZINC000000968310 | fda.331 | -3.86 | -5.122 |
| ZINC000000968310 | fda.332 | -3.86 | -5.122 |
| ZINC000000968310 | fda.333 | -3.86 | -5.122 |
| ZINC000000000509 | fda.1549 | -3.86 | -5.122 |
| ZINC000000000509 | fda.1550 | -3.86 | -5.122 |
| ZINC000000000509 | fda.1551 | -3.86 | -5.122 |
| ZINC000033965961 | fda.2034 | -3.859 | -5.28 |
| ZINC000033965961 | fda.2035 | -3.859 | -5.28 |
| ZINC000004577910 | fda.1231 | -3.859 | -3.859 |
| ZINC000003936683 | fda.1437 | -3.858 | -3.861 |
| ZINC000003936683 | fda.1438 | -3.858 | -3.861 |
| ZINC000004632106 | fda.1232 | -3.858 | -4.576 |
| ZINC000003645145 | fda.1251 | -3.857 | -3.857 |
| ZINC000000000905 | fda.1154 | -3.857 | -5.362 |
| ZINC000003941496 | fda.420 | -3.856 | -3.864 |
| ZINC000003798757 | fda.1740 | -3.856 | -4.061 |
| ZINC000003977978 | fda.415 | -3.856 | -3.856 |
| ZINC000001612996 | fda.10 | -3.855 | -3.858 |
| ZINC000252679615 | fda.341 | -3.855 | -5.136 |
| ZINC000252679615 | fda.342 | -3.855 | -5.136 |
| ZINC000002568036 | fda.53 | -3.855 | -5.136 |
| ZINC000002568036 | fda.54 | -3.855 | -5.136 |
| ZINC000007997966 | fda.1990 | -3.855 | -5.136 |
| ZINC000007997966 | fda.1991 | -3.855 | -5.136 |
| ZINC000035902489 | fda.1537 | -3.854 | -4.35 |
| ZINC000003830218 | fda.281 | -3.854 | -4.531 |
| ZINC000001533877 | fda.818 | -3.853 | -3.944 |
| ZINC000001533877 | fda.819 | -3.853 | -3.944 |
| ZINC000001533877 | fda.822 | -3.853 | -3.944 |
| ZINC000001533877 | fda.823 | -3.853 | -3.944 |
| ZINC000035653007 | fda.1545 | -3.853 | -4.533 |
| ZINC000001533877 | fda.820 | -3.852 | -3.943 |
| ZINC000003581355 | fda.1250 | -3.851 | -3.851 |
| ZINC000004632106 | fda.1233 | -3.851 | -4.57 |
| ZINC000001532526 | fda.620 | -3.85 | -3.85 |
| ZINC000004474414 | fda.240 | -3.849 | -3.849 |
| ZINC000001530981 | fda.204 | -3.849 | -4.849 |
| ZINC000001530981 | fda.205 | -3.849 | -4.849 |
| ZINC000001530981 | fda.206 | -3.849 | -4.849 |
| ZINC000000896740 | fda.1497 | -3.848 | -4.056 |
| ZINC000000607971 | fda.1506 | -3.848 | -4.056 |
| ZINC000000388081 | fda.1476 | -3.845 | -4.3 |
| ZINC000003938686 | fda.1753 | -3.844 | -4.313 |
| ZINC000000000711 | fda.1940 | -3.843 | -3.854 |
| ZINC000001843047 | fda.1964 | -3.843 | -3.854 |
| ZINC000003806104 | fda.2046 | -3.843 | -3.843 |
| ZINC000003873936 | fda.1732 | -3.842 | -4.089 |
| ZINC000003873936 | fda.1733 | -3.842 | -4.089 |
| ZINC000001536779 | fda.797 | -3.841 | -3.842 |
| ZINC000001533877 | fda.818 | -3.841 | -3.932 |
| ZINC000001533877 | fda.819 | -3.841 | -3.932 |
| ZINC000001533877 | fda.822 | -3.841 | -3.932 |
| ZINC000001533877 | fda.823 | -3.841 | -3.932 |
| ZINC000084441937 | fda.1908 | -3.84 | -5.468 |
| ZINC000084441937 | fda.1910 | -3.84 | -5.468 |
| ZINC000038197764 | fda.1019 | -3.839 | -3.932 |
| ZINC000003607120 | fda.8 | -3.839 | -3.932 |
| ZINC000100037020 | fda.1533 | -3.838 | -4.548 |
| ZINC000100037020 | fda.1534 | -3.838 | -4.548 |
| ZINC000000020783 | fda.588 | -3.834 | -3.834 |
| ZINC000001542392 | fda.141 | -3.834 | -3.842 |
| ZINC000014210876 | fda.1797 | -3.834 | -5.138 |
| ZINC000003938686 | fda.1752 | -3.834 | -4.303 |
| ZINC000001530571 | fda.934 | -3.833 | -4.163 |
| ZINC000001530571 | fda.935 | -3.833 | -4.163 |
| ZINC000094566093 | fda.1303 | -3.833 | -4.871 |
| ZINC000094566092 | fda.1315 | -3.833 | -4.871 |
| ZINC000000389747 | fda.32 | -3.833 | -4.92 |
| ZINC000000389747 | fda.33 | -3.833 | -4.92 |
| ZINC000000389747 | fda.34 | -3.833 | -4.92 |
| ZINC000003875259 | fda.962 | -3.831 | -3.832 |
| ZINC000000896740 | fda.1498 | -3.829 | -4.036 |
| ZINC000000607971 | fda.1507 | -3.829 | -4.036 |
| ZINC000003812863 | fda.1649 | -3.828 | -4.825 |
| ZINC000043207238 | fda.1851 | -3.828 | -3.828 |
| ZINC000000000596 | fda.1441 | -3.827 | -3.839 |
| ZINC000000000596 | fda.1442 | -3.827 | -3.839 |
| ZINC000003820029 | fda.143 | -3.827 | -3.828 |
| ZINC000001280665 | fda.412 | -3.827 | -5.615 |
| ZINC000001280665 | fda.413 | -3.827 | -5.615 |
| ZINC000001280665 | fda.414 | -3.827 | -5.615 |
| ZINC000003079342 | fda.316 | -3.827 | -3.827 |
| ZINC000000896711 | fda.1080 | -3.826 | -3.827 |
| ZINC000000896709 | fda.1655 | -3.826 | -3.827 |
| ZINC000100378061 | fda.530 | -3.825 | -4.191 |
| ZINC000100378061 | fda.532 | -3.825 | -4.191 |
| ZINC000000000973 | fda.474 | -3.825 | -5.191 |
| ZINC000000389747 | fda.32 | -3.824 | -3.948 |
| ZINC000000389747 | fda.33 | -3.824 | -3.948 |
| ZINC000000389747 | fda.34 | -3.824 | -3.948 |
| ZINC000000020244 | fda.1049 | -3.823 | -3.837 |
| ZINC000000020244 | fda.1050 | -3.823 | -3.837 |
| ZINC000001534965 | fda.601 | -3.823 | -3.833 |
| ZINC000100009278 | fda.1242 | -3.823 | -3.825 |
| ZINC000095452610 | fda.1323 | -3.822 | -4.233 |
| ZINC000003960338 | fda.134 | -3.821 | -3.821 |
| ZINC000029416466 | fda.158 | -3.821 | -3.834 |
| ZINC000003995807 | fda.2073 | -3.82 | -3.82 |
| ZINC000000897002 | fda.1082 | -3.82 | -5.261 |
| ZINC000000897002 | fda.1083 | -3.82 | -5.261 |
| ZINC000000897002 | fda.1084 | -3.82 | -5.261 |
| ZINC000084400879 | fda.302 | -3.82 | -4.041 |
| ZINC000084400879 | fda.303 | -3.82 | -4.041 |
| ZINC000084400879 | fda.304 | -3.82 | -4.041 |
| ZINC000000020245 | fda.1812 | -3.82 | -3.82 |
| ZINC000003873296 | fda.1769 | -3.819 | -4.858 |
| ZINC000014879992 | fda.733 | -3.818 | -3.844 |
| ZINC000019418959 | fda.779 | -3.817 | -4.097 |
| ZINC000019418959 | fda.780 | -3.817 | -4.097 |
| ZINC000019418959 | fda.781 | -3.817 | -4.097 |
| ZINC000000005152 | fda.432 | -3.815 | -3.815 |
| ZINC000003812867 | fda.1743 | -3.815 | -4.438 |
| ZINC000000020228 | fda.326 | -3.814 | -4.471 |
| ZINC000000057206 | fda.988 | -3.814 | -4.471 |
| ZINC000001996784 | fda.982 | -3.813 | -3.981 |
| ZINC000006745272 | fda.365 | -3.813 | -3.813 |
| ZINC000013648755 | fda.418 | -3.813 | -4.854 |
| ZINC000001530756 | fda.1634 | -3.812 | -3.816 |
| ZINC000002847375 | fda.1527 | -3.812 | -3.956 |
| ZINC000002847375 | fda.1528 | -3.812 | -3.956 |
| ZINC000000000215 | fda.1837 | -3.812 | -3.956 |
| ZINC000000000215 | fda.1838 | -3.812 | -3.956 |
| ZINC000014210876 | fda.1798 | -3.811 | -5.115 |
| ZINC000000001931 | fda.1942 | -3.81 | -3.83 |
| ZINC000000001931 | fda.1943 | -3.81 | -3.83 |
| ZINC000036766734 | fda.1480 | -3.809 | -3.813 |
| ZINC000001996784 | fda.982 | -3.807 | -4.658 |
| ZINC000034089131 | fda.73 | -3.807 | -3.821 |
| ZINC000005733652 | fda.1265 | -3.806 | -5.574 |
| ZINC000005733652 | fda.1268 | -3.806 | -5.574 |
| ZINC000000000973 | fda.475 | -3.806 | -5.172 |
| ZINC000019796155 | fda.711 | -3.805 | -5.674 |
| ZINC000019796155 | fda.712 | -3.805 | -5.674 |
| ZINC000003964325 | fda.1760 | -3.805 | -3.809 |
| ZINC000000895032 | fda.1381 | -3.804 | -3.804 |
| ZINC000003944422 | fda.127 | -3.804 | -3.804 |
| ZINC000003991624 | fda.69 | -3.802 | -3.807 |
| ZINC000003938686 | fda.1752 | -3.802 | -4.209 |
| ZINC000000388081 | fda.1474 | -3.8 | -4.255 |
| ZINC000018516586 | fda.428 | -3.799 | -6.175 |
| ZINC000095616601 | fda.17 | -3.797 | -3.889 |
| ZINC000019166991 | fda.1578 | -3.796 | -4.576 |
| ZINC000001530977 | fda.890 | -3.795 | -5.025 |
| ZINC000001530977 | fda.891 | -3.795 | -5.025 |
| ZINC000001530977 | fda.892 | -3.795 | -5.025 |
| ZINC000000075126 | fda.393 | -3.794 | -4.777 |
| ZINC000000075126 | fda.394 | -3.794 | -4.777 |
| ZINC000000004448 | fda.107 | -3.794 | -4.777 |
| ZINC000000004448 | fda.108 | -3.794 | -4.777 |
| ZINC000002599970 | fda.56 | -3.794 | -3.806 |
| ZINC000000897256 | fda.2009 | -3.794 | -3.806 |
| ZINC000003953037 | fda.1758 | -3.793 | -3.839 |
| ZINC000003953037 | fda.1759 | -3.793 | -3.838 |
| ZINC000000000973 | fda.474 | -3.788 | -4.113 |
| ZINC000049933061 | fda.1907 | -3.787 | -3.859 |
| ZINC000019632917 | fda.709 | -3.786 | -3.803 |
| ZINC000011681534 | fda.2022 | -3.785 | -5.809 |
| ZINC000006661227 | fda.1197 | -3.785 | -4.219 |
| ZINC000006661227 | fda.1198 | -3.785 | -4.219 |
| ZINC000003781664 | fda.1707 | -3.784 | -3.784 |
| ZINC000000538621 | fda.854 | -3.784 | -4.897 |
| ZINC000066166864 | fda.443 | -3.783 | -4.663 |
| ZINC000066166864 | fda.444 | -3.783 | -4.663 |
| ZINC000038197764 | fda.1020 | -3.782 | -4.928 |
| ZINC000003607120 | fda.9 | -3.782 | -4.928 |
| ZINC000000002688 | fda.1945 | -3.781 | -4.44 |
| ZINC000094566093 | fda.1304 | -3.781 | -4.228 |
| ZINC000000006157 | fda.814 | -3.778 | -5.422 |
| ZINC000001999487 | fda.1757 | -3.778 | -5.422 |
| ZINC000034781704 | fda.1192 | -3.776 | -3.799 |
| ZINC000022010382 | fda.641 | -3.774 | -3.989 |
| ZINC000022010387 | fda.2103 | -3.774 | -3.989 |
| ZINC000003875392 | fda.562 | -3.773 | -5.268 |
| ZINC000003875392 | fda.563 | -3.773 | -5.268 |
| ZINC000003875392 | fda.564 | -3.773 | -5.268 |
| ZINC000000000751 | fda.1414 | -3.773 | -5.268 |
| ZINC000000000751 | fda.1415 | -3.773 | -5.268 |
| ZINC000000000751 | fda.1416 | -3.773 | -5.268 |
| ZINC000000075008 | fda.674 | -3.772 | -4.527 |
| ZINC000000075008 | fda.675 | -3.772 | -4.527 |
| ZINC000001530571 | fda.934 | -3.771 | -4.276 |
| ZINC000001530571 | fda.935 | -3.771 | -4.276 |
| ZINC000005752191 | fda.1032 | -3.771 | -3.771 |
| ZINC000000538312 | fda.840 | -3.769 | -3.779 |
| ZINC000000538312 | fda.841 | -3.769 | -3.779 |
| ZINC000072318121 | fda.2001 | -3.769 | -3.971 |
| ZINC000072318121 | fda.2002 | -3.769 | -3.971 |
| ZINC000019875504 | fda.1902 | -3.768 | -4.172 |
| ZINC000001532728 | fda.1421 | -3.768 | -3.768 |
| ZINC000004632106 | fda.1233 | -3.767 | -4.256 |
| ZINC000003938684 | fda.1676 | -3.767 | -3.767 |
| ZINC000038197764 | fda.1019 | -3.766 | -4.912 |
| ZINC000003607120 | fda.8 | -3.766 | -4.912 |
| ZINC000019875504 | fda.1901 | -3.766 | -4.183 |
| ZINC000019875504 | fda.1902 | -3.766 | -4.183 |
| ZINC000000601254 | fda.866 | -3.764 | -3.766 |
| ZINC000000896755 | fda.1656 | -3.764 | -3.766 |
| ZINC000003914810 | fda.267 | -3.763 | -3.767 |
| ZINC000000155905 | fda.505 | -3.763 | -3.775 |
| ZINC000003831551 | fda.68 | -3.763 | -3.775 |
| ZINC000035801098 | fda.1547 | -3.762 | -5.543 |
| ZINC000035801098 | fda.1548 | -3.762 | -5.543 |
| ZINC000019418959 | fda.779 | -3.762 | -4.371 |
| ZINC000019418959 | fda.780 | -3.762 | -4.371 |
| ZINC000019418959 | fda.781 | -3.762 | -4.371 |
| ZINC000004474460 | fda.255 | -3.761 | -3.761 |
| ZINC000000896484 | fda.298 | -3.76 | -5.693 |
| ZINC000000896484 | fda.299 | -3.76 | -5.693 |
| ZINC000003931840 | fda.395 | -3.759 | -3.761 |
| ZINC000008214402 | fda.1598 | -3.758 | -3.769 |
| ZINC000008214402 | fda.1599 | -3.758 | -3.769 |
| ZINC000000020248 | fda.1813 | -3.757 | -3.757 |
| ZINC000012503151 | fda.566 | -3.756 | -4.351 |
| ZINC000253917094 | fda.2094 | -3.756 | -4.351 |
| ZINC000000120286 | fda.6 | -3.756 | -4.052 |
| ZINC000003581355 | fda.1250 | -3.756 | -3.756 |
| ZINC000003871923 | fda.1397 | -3.756 | -3.756 |
| ZINC000000538273 | fda.952 | -3.756 | -4.977 |
| ZINC000000538273 | fda.953 | -3.756 | -4.977 |
| ZINC000000537891 | fda.1373 | -3.756 | -4.977 |
| ZINC000000537891 | fda.1374 | -3.756 | -4.977 |
| ZINC000003818726 | fda.185 | -3.753 | -3.775 |
| ZINC000000897240 | fda.1362 | -3.753 | -3.754 |
| ZINC000000897240 | fda.1363 | -3.753 | -3.754 |
| ZINC000000897240 | fda.1364 | -3.753 | -3.754 |
| ZINC000003831490 | fda.374 | -3.752 | -5.221 |
| ZINC000003831490 | fda.375 | -3.752 | -5.221 |
| ZINC000013537284 | fda.748 | -3.752 | -4.786 |
| ZINC000000001341 | fda.1558 | -3.751 | -5.041 |
| ZINC000000001341 | fda.1559 | -3.751 | -5.041 |
| ZINC000019594557 | fda.755 | -3.751 | -3.944 |
| ZINC000019594557 | fda.756 | -3.751 | -3.944 |
| ZINC000019361042 | fda.762 | -3.751 | -3.944 |
| ZINC000019361042 | fda.763 | -3.751 | -3.944 |
| ZINC000012404516 | fda.199 | -3.75 | -3.77 |
| ZINC000012404516 | fda.200 | -3.75 | -3.77 |
| ZINC000095619105 | fda.1193 | -3.747 | -4 |
| ZINC000095619105 | fda.1194 | -3.747 | -4 |
| ZINC000095619105 | fda.1195 | -3.747 | -4 |
| ZINC000005733652 | fda.1266 | -3.747 | -5.515 |
| ZINC000005733652 | fda.1267 | -3.747 | -5.515 |
| ZINC000000005152 | fda.432 | -3.745 | -3.745 |
| ZINC000100299039 | fda.1965 | -3.745 | -4.156 |
| ZINC000035653009 | fda.1546 | -3.744 | -4.424 |
| ZINC000006627681 | fda.1095 | -3.743 | -4.213 |
| ZINC000014210876 | fda.1797 | -3.743 | -5.047 |
| ZINC000002510358 | fda.912 | -3.741 | -5.268 |
| ZINC000002510358 | fda.913 | -3.741 | -5.268 |
| ZINC000000000083 | fda.754 | -3.74 | -3.74 |
| ZINC000003830314 | fda.1815 | -3.74 | -3.74 |
| ZINC000000000242 | fda.684 | -3.738 | -3.742 |
| ZINC000000000242 | fda.685 | -3.738 | -3.742 |
| ZINC000007997952 | fda.1572 | -3.738 | -3.742 |
| ZINC000007997952 | fda.1573 | -3.738 | -3.742 |
| ZINC000029571072 | fda.2033 | -3.738 | -4.17 |
| ZINC000049637509 | fda.2042 | -3.738 | -4.17 |
| ZINC000001530613 | fda.915 | -3.738 | -3.755 |
| ZINC000001530613 | fda.916 | -3.738 | -3.755 |
| ZINC000004632106 | fda.1232 | -3.737 | -4.455 |
| ZINC000096272772 | fda.1930 | -3.733 | -3.733 |
| ZINC000014210876 | fda.1798 | -3.733 | -5.007 |
| ZINC000000000096 | fda.946 | -3.73 | -3.73 |
| ZINC000000896455 | fda.1452 | -3.73 | -3.73 |
| ZINC000000034157 | fda.635 | -3.723 | -5.818 |
| ZINC000003972949 | fda.1984 | -3.719 | -3.733 |
| ZINC000003872994 | fda.218 | -3.718 | -6.193 |
| ZINC000000643153 | fda.1820 | -3.718 | -6.193 |
| ZINC000003808779 | fda.974 | -3.716 | -5.517 |
| ZINC000086040406 | fda.1240 | -3.715 | -3.729 |
| ZINC000036294079 | fda.679 | -3.714 | -4.796 |
| ZINC000035999642 | fda.746 | -3.714 | -4.796 |
| ZINC000000007673 | fda.817 | -3.712 | -3.712 |
| ZINC000013831130 | fda.728 | -3.711 | -3.982 |
| ZINC000013831130 | fda.729 | -3.711 | -3.982 |
| ZINC000013986658 | fda.542 | -3.71 | -3.73 |
| ZINC000014210876 | fda.1798 | -3.71 | -4.437 |
| ZINC000100378061 | fda.530 | -3.707 | -4.331 |
| ZINC000100378061 | fda.532 | -3.707 | -4.331 |
| ZINC000000388081 | fda.1475 | -3.706 | -4.161 |
| ZINC000016052277 | fda.1897 | -3.706 | -3.738 |
| ZINC000003812983 | fda.1482 | -3.705 | -5.192 |
| ZINC000003812983 | fda.1483 | -3.705 | -5.192 |
| ZINC000004102194 | fda.1109 | -3.705 | -3.708 |
| ZINC000003873936 | fda.1732 | -3.704 | -4.667 |
| ZINC000003873936 | fda.1733 | -3.704 | -4.667 |
| ZINC000001533877 | fda.821 | -3.704 | -3.795 |
| ZINC000000001681 | fda.1668 | -3.703 | -3.718 |
| ZINC000000001681 | fda.1669 | -3.703 | -3.718 |
| ZINC000000537957 | fda.852 | -3.703 | -3.749 |
| ZINC000003833846 | fda.95 | -3.701 | -3.715 |
| ZINC000003818808 | fda.492 | -3.699 | -4.11 |
| ZINC000000538065 | fda.838 | -3.699 | -4.102 |
| ZINC000000538065 | fda.839 | -3.699 | -4.102 |
| ZINC000003812988 | fda.2055 | -3.699 | -6.077 |
| ZINC000003812988 | fda.2056 | -3.699 | -6.077 |
| ZINC000003813042 | fda.1814 | -3.698 | -4.621 |
| ZINC000026985532 | fda.356 | -3.698 | -3.711 |
| ZINC000026985532 | fda.357 | -3.698 | -3.711 |
| ZINC000004658557 | fda.268 | -3.698 | -3.698 |
| ZINC000001543916 | fda.1855 | -3.697 | -3.911 |
| ZINC000003931840 | fda.396 | -3.697 | -3.699 |
| ZINC000000002009 | fda.1726 | -3.696 | -3.696 |
| ZINC000084400879 | fda.302 | -3.696 | -3.916 |
| ZINC000084400879 | fda.303 | -3.696 | -3.916 |
| ZINC000084400879 | fda.304 | -3.696 | -3.916 |
| ZINC000019632614 | fda.1114 | -3.693 | -4.099 |
| ZINC000019632614 | fda.1115 | -3.693 | -4.099 |
| ZINC000001530713 | fda.788 | -3.692 | -4.418 |
| ZINC000000005823 | fda.44 | -3.691 | -4.957 |
| ZINC000003787097 | fda.555 | -3.689 | -5.022 |
| ZINC000003830716 | fda.1308 | -3.688 | -4.073 |
| ZINC000003830716 | fda.1309 | -3.688 | -4.073 |
| ZINC000095564694 | fda.2095 | -3.686 | -4.932 |
| ZINC000095564694 | fda.2096 | -3.686 | -4.932 |
| ZINC000095564694 | fda.2097 | -3.686 | -4.932 |
| ZINC000001530968 | fda.1520 | -3.685 | -3.694 |
| ZINC000003812306 | fda.2065 | -3.684 | -4.558 |
| ZINC000000002281 | fda.1029 | -3.681 | -5.897 |
| ZINC000000000746 | fda.149 | -3.681 | -5.897 |
| ZINC000003918138 | fda.964 | -3.679 | -3.679 |
| ZINC000019875504 | fda.1901 | -3.679 | -4.083 |
| ZINC000095616599 | fda.1288 | -3.676 | -4.75 |
| ZINC000095616600 | fda.1325 | -3.676 | -4.75 |
| ZINC000095616599 | fda.1288 | -3.675 | -4.748 |
| ZINC000095616600 | fda.1325 | -3.675 | -4.748 |
| ZINC000004474460 | fda.255 | -3.673 | -3.673 |
| ZINC000019419017 | fda.771 | -3.673 | -4.489 |
| ZINC000019419017 | fda.772 | -3.673 | -4.489 |
| ZINC000100036924 | fda.1105 | -3.671 | -3.711 |
| ZINC000008214514 | fda.1263 | -3.671 | -3.671 |
| ZINC000003830993 | fda.1462 | -3.67 | -4.659 |
| ZINC000001530912 | fda.809 | -3.667 | -4.237 |
| ZINC000001530912 | fda.810 | -3.667 | -4.237 |
| ZINC000001530912 | fda.811 | -3.667 | -4.237 |
| ZINC000004099200 | fda.836 | -3.667 | -4.374 |
| ZINC000004676424 | fda.881 | -3.667 | -4.374 |
| ZINC000000001644 | fda.1412 | -3.666 | -5.455 |
| ZINC000001530968 | fda.1521 | -3.665 | -3.674 |
| ZINC000001530600 | fda.1144 | -3.665 | -3.961 |
| ZINC000004474414 | fda.240 | -3.665 | -3.665 |
| ZINC000001529323 | fda.16 | -3.663 | -5.099 |
| ZINC000100036536 | fda.1296 | -3.663 | -3.665 |
| ZINC000008552123 | fda.1567 | -3.663 | -5.158 |
| ZINC000008552123 | fda.1568 | -3.663 | -5.158 |
| ZINC000008552123 | fda.1569 | -3.663 | -5.158 |
| ZINC000003875393 | fda.1718 | -3.663 | -5.158 |
| ZINC000003875393 | fda.1719 | -3.663 | -5.158 |
| ZINC000003875393 | fda.1720 | -3.663 | -5.158 |
| ZINC000030691763 | fda.1339 | -3.663 | -3.663 |
| ZINC000030691760 | fda.1342 | -3.663 | -3.663 |
| ZINC000019362735 | fda.657 | -3.662 | -4.057 |
| ZINC000019362735 | fda.658 | -3.662 | -4.057 |
| ZINC000019362735 | fda.659 | -3.662 | -4.057 |
| ZINC000019362737 | fda.660 | -3.662 | -4.057 |
| ZINC000019362737 | fda.661 | -3.662 | -4.057 |
| ZINC000019362737 | fda.662 | -3.662 | -4.057 |
| ZINC000004099200 | fda.835 | -3.662 | -4.376 |
| ZINC000004676424 | fda.880 | -3.662 | -4.376 |
| ZINC000100378061 | fda.531 | -3.662 | -4.028 |
| ZINC000019228902 | fda.1575 | -3.661 | -3.997 |
| ZINC000019228902 | fda.1576 | -3.661 | -3.997 |
| ZINC000019228902 | fda.1577 | -3.661 | -3.997 |
| ZINC000022010382 | fda.639 | -3.661 | -4.367 |
| ZINC000022010382 | fda.640 | -3.661 | -4.367 |
| ZINC000022010382 | fda.641 | -3.661 | -4.367 |
| ZINC000022010387 | fda.2101 | -3.661 | -4.367 |
| ZINC000022010387 | fda.2102 | -3.661 | -4.367 |
| ZINC000022010387 | fda.2103 | -3.661 | -4.367 |
| ZINC000019632718 | fda.111 | -3.659 | -3.659 |
| ZINC000019632713 | fda.126 | -3.659 | -3.659 |
| ZINC000000000565 | fda.686 | -3.658 | -3.672 |
| ZINC000000000565 | fda.687 | -3.658 | -3.672 |
| ZINC000000155269 | fda.1832 | -3.658 | -3.672 |
| ZINC000000155269 | fda.1833 | -3.658 | -3.672 |
| ZINC000003776970 | fda.1692 | -3.658 | -4.232 |
| ZINC000006094354 | fda.765 | -3.654 | -3.655 |
| ZINC000006094354 | fda.766 | -3.654 | -3.655 |
| ZINC000003795819 | fda.1876 | -3.654 | -3.655 |
| ZINC000003795819 | fda.1877 | -3.654 | -3.655 |
| ZINC000006467621 | fda.1988 | -3.65 | -4.927 |
| ZINC000003831404 | fda.30 | -3.65 | -5.927 |
| ZINC000003831404 | fda.31 | -3.65 | -5.927 |
| ZINC000001530636 | fda.1524 | -3.65 | -4.565 |
| ZINC000222731806 | fda.1365 | -3.649 | -3.649 |
| ZINC000000601301 | fda.320 | -3.649 | -5.023 |
| ZINC000000897222 | fda.1658 | -3.649 | -5.023 |
| ZINC000004097305 | fda.945 | -3.648 | -3.648 |
| ZINC000001530654 | fda.1622 | -3.648 | -3.698 |
| ZINC000001530654 | fda.1623 | -3.648 | -3.698 |
| ZINC000001538857 | fda.139 | -3.647 | -3.648 |
| ZINC000000527386 | fda.999 | -3.647 | -3.648 |
| ZINC000038197764 | fda.1019 | -3.645 | -4.791 |
| ZINC000003607120 | fda.8 | -3.645 | -4.791 |
| ZINC000001842633 | fda.1429 | -3.641 | -3.641 |
| ZINC000000901552 | fda.1531 | -3.641 | -3.647 |
| ZINC000000901555 | fda.1532 | -3.641 | -3.647 |
| ZINC000003801163 | fda.1709 | -3.638 | -3.792 |
| ZINC000000897385 | fda.1426 | -3.638 | -4.253 |
| ZINC000000897385 | fda.1427 | -3.638 | -4.253 |
| ZINC000000608101 | fda.1228 | -3.638 | -4.253 |
| ZINC000000608101 | fda.1229 | -3.638 | -4.253 |
| ZINC000002522669 | fda.910 | -3.635 | -3.652 |
| ZINC000002522669 | fda.911 | -3.635 | -3.652 |
| ZINC000019632614 | fda.1114 | -3.634 | -4.049 |
| ZINC000019632614 | fda.1115 | -3.634 | -4.049 |
| ZINC000003995811 | fda.2074 | -3.633 | -3.633 |
| ZINC000036294079 | fda.679 | -3.633 | -3.737 |
| ZINC000035999642 | fda.746 | -3.633 | -3.737 |
| ZINC000000968275 | fda.1428 | -3.633 | -3.633 |
| ZINC000003831586 | fda.1817 | -3.633 | -3.633 |
| ZINC000003818808 | fda.492 | -3.633 | -4.043 |
| ZINC000000895042 | fda.231 | -3.631 | -3.646 |
| ZINC000003830449 | fda.1699 | -3.631 | -3.631 |
| ZINC000001533877 | fda.818 | -3.63 | -4.8 |
| ZINC000001533877 | fda.819 | -3.63 | -4.8 |
| ZINC000001533877 | fda.822 | -3.63 | -4.8 |
| ZINC000001533877 | fda.823 | -3.63 | -4.8 |
| ZINC000001611274 | fda.585 | -3.63 | -4.907 |
| ZINC000001611274 | fda.586 | -3.63 | -4.907 |
| ZINC000053084692 | fda.75 | -3.628 | -4.407 |
| ZINC000000000973 | fda.475 | -3.628 | -4.954 |
| ZINC000002016037 | fda.655 | -3.628 | -4.242 |
| ZINC000002016037 | fda.656 | -3.628 | -4.242 |
| ZINC000000606383 | fda.1226 | -3.628 | -4.242 |
| ZINC000000606383 | fda.1227 | -3.628 | -4.242 |
| ZINC000003917708 | fda.1886 | -3.627 | -3.654 |
| ZINC000002016037 | fda.655 | -3.627 | -3.886 |
| ZINC000000606383 | fda.1226 | -3.627 | -3.886 |
| ZINC000019166991 | fda.1578 | -3.626 | -4.633 |
| ZINC000085537014 | fda.906 | -3.626 | -3.937 |
| ZINC000085537014 | fda.907 | -3.626 | -3.937 |
| ZINC000000156792 | fda.905 | -3.625 | -3.627 |
| ZINC000000968255 | fda.1779 | -3.625 | -3.627 |
| ZINC000022056030 | fda.145 | -3.625 | -3.726 |
| ZINC000022056030 | fda.146 | -3.625 | -3.726 |
| ZINC000022056030 | fda.147 | -3.625 | -3.726 |
| ZINC000019632633 | fda.224 | -3.625 | -3.726 |
| ZINC000019632633 | fda.225 | -3.625 | -3.726 |
| ZINC000019632633 | fda.226 | -3.625 | -3.726 |
| ZINC000004099200 | fda.836 | -3.625 | -5.317 |
| ZINC000004676424 | fda.881 | -3.625 | -5.317 |
| ZINC000100036536 | fda.1297 | -3.617 | -3.619 |
| ZINC000000538065 | fda.838 | -3.617 | -4.036 |
| ZINC000000538065 | fda.839 | -3.617 | -4.036 |
| ZINC000001548097 | fda.1857 | -3.617 | -3.63 |
| ZINC000001548097 | fda.1858 | -3.617 | -3.63 |
| ZINC000001530572 | fda.914 | -3.617 | -3.617 |
| ZINC000000002688 | fda.1945 | -3.616 | -3.853 |
| ZINC000003945984 | fda.1983 | -3.614 | -3.614 |
| ZINC000003830218 | fda.282 | -3.613 | -4.29 |
| ZINC000003830993 | fda.1461 | -3.613 | -4.601 |
| ZINC000001481956 | fda.795 | -3.612 | -3.622 |
| ZINC000001481956 | fda.796 | -3.612 | -3.622 |
| ZINC000004214700 | fda.995 | -3.612 | -3.622 |
| ZINC000004214700 | fda.996 | -3.612 | -3.622 |
| ZINC000000388081 | fda.1476 | -3.612 | -4.943 |
| ZINC000008214402 | fda.1598 | -3.611 | -3.622 |
| ZINC000008214402 | fda.1599 | -3.611 | -3.622 |
| ZINC000003938704 | fda.156 | -3.611 | -5.451 |
| ZINC000003816514 | fda.1697 | -3.61 | -3.692 |
| ZINC000000388081 | fda.1475 | -3.61 | -4.941 |
| ZINC000018043251 | fda.284 | -3.61 | -5.442 |
| ZINC000019632917 | fda.709 | -3.609 | -5.742 |
| ZINC000003830218 | fda.281 | -3.609 | -4.285 |
| ZINC000000491073 | fda.977 | -3.608 | -5.724 |
| ZINC000000491073 | fda.978 | -3.608 | -5.724 |
| ZINC000000491073 | fda.979 | -3.608 | -5.724 |
| ZINC000000596881 | fda.310 | -3.608 | -4.331 |
| ZINC000000596881 | fda.311 | -3.608 | -4.331 |
| ZINC000000643055 | fda.1499 | -3.608 | -4.331 |
| ZINC000000643055 | fda.1500 | -3.608 | -4.331 |
| ZINC000004099200 | fda.835 | -3.608 | -5.232 |
| ZINC000004676424 | fda.880 | -3.608 | -5.232 |
| ZINC000003830391 | fda.362 | -3.607 | -4.331 |
| ZINC000000013156 | fda.861 | -3.607 | -5.427 |
| ZINC000000000456 | fda.1220 | -3.605 | -4.736 |
| ZINC000000000456 | fda.1221 | -3.605 | -4.736 |
| ZINC000000000456 | fda.1222 | -3.605 | -4.736 |
| ZINC000000154964 | fda.1834 | -3.605 | -4.736 |
| ZINC000000154964 | fda.1835 | -3.605 | -4.736 |
| ZINC000000154964 | fda.1836 | -3.605 | -4.736 |
| ZINC000014210457 | fda.730 | -3.602 | -3.602 |
| ZINC000013973998 | fda.498 | -3.601 | -3.604 |
| ZINC000001554010 | fda.82 | -3.601 | -3.604 |
| ZINC000003938746 | fda.1673 | -3.599 | -3.636 |
| ZINC000003938746 | fda.1674 | -3.599 | -3.636 |
| ZINC000000000456 | fda.1220 | -3.598 | -3.693 |
| ZINC000000000456 | fda.1222 | -3.598 | -3.693 |
| ZINC000000154964 | fda.1836 | -3.598 | -3.693 |
| ZINC000085537017 | fda.2092 | -3.597 | -5.824 |
| ZINC000100008319 | fda.191 | -3.597 | -3.598 |
| ZINC000004632106 | fda.1232 | -3.597 | -4.087 |
| ZINC000003938652 | fda.1982 | -3.597 | -3.597 |
| ZINC000022448983 | fda.399 | -3.595 | -3.708 |
| ZINC000022448983 | fda.400 | -3.595 | -3.708 |
| ZINC000022448983 | fda.401 | -3.595 | -3.708 |
| ZINC000003812989 | fda.1712 | -3.594 | -3.624 |
| ZINC000003812989 | fda.1713 | -3.594 | -3.624 |
| ZINC000000901061 | fda.1058 | -3.592 | -3.592 |
| ZINC000003830347 | fda.196 | -3.589 | -5.08 |
| ZINC000003830347 | fda.197 | -3.589 | -5.08 |
| ZINC000003775644 | fda.970 | -3.588 | -5.355 |
| ZINC000001999441 | fda.490 | -3.587 | -5.611 |
| ZINC000003875392 | fda.562 | -3.587 | -5.082 |
| ZINC000003875392 | fda.563 | -3.587 | -5.082 |
| ZINC000003875392 | fda.564 | -3.587 | -5.082 |
| ZINC000000000751 | fda.1414 | -3.587 | -5.082 |
| ZINC000000000751 | fda.1415 | -3.587 | -5.082 |
| ZINC000000000751 | fda.1416 | -3.587 | -5.082 |
| ZINC000000968305 | fda.1432 | -3.587 | -5.199 |
| ZINC000000968305 | fda.1433 | -3.587 | -5.199 |
| ZINC000019144226 | fda.1589 | -3.587 | -3.587 |
| ZINC000019144231 | fda.1864 | -3.587 | -3.587 |
| ZINC000100014909 | fda.1280 | -3.583 | -4.438 |
| ZINC000100014909 | fda.1281 | -3.583 | -4.438 |
| ZINC000094566092 | fda.1316 | -3.583 | -4.016 |
| ZINC000003079337 | fda.1249 | -3.579 | -3.579 |
| ZINC000001530929 | fda.1950 | -3.578 | -4.99 |
| ZINC000001530929 | fda.1951 | -3.578 | -4.99 |
| ZINC000003876068 | fda.1976 | -3.578 | -4.99 |
| ZINC000003876068 | fda.1977 | -3.578 | -4.99 |
| ZINC000001995484 | fda.1275 | -3.578 | -4.287 |
| ZINC000013831130 | fda.728 | -3.577 | -4.17 |
| ZINC000000896731 | fda.1355 | -3.575 | -5.635 |
| ZINC000000896731 | fda.1356 | -3.575 | -5.635 |
| ZINC000000000850 | fda.1146 | -3.575 | -5.635 |
| ZINC000000000850 | fda.1147 | -3.575 | -5.635 |
| ZINC000001530718 | fda.882 | -3.574 | -3.576 |
| ZINC000001530717 | fda.1116 | -3.574 | -3.576 |
| ZINC000000002176 | fda.648 | -3.572 | -3.573 |
| ZINC000000403609 | fda.1179 | -3.572 | -3.573 |
| ZINC000035801098 | fda.1548 | -3.571 | -5.909 |
| ZINC000003932831 | fda.1751 | -3.569 | -3.569 |
| ZINC000018516586 | fda.428 | -3.569 | -4.205 |
| ZINC000000004319 | fda.318 | -3.568 | -5.506 |
| ZINC000000403010 | fda.1458 | -3.568 | -5.506 |
| ZINC000000968326 | fda.114 | -3.563 | -5.439 |
| ZINC000000968326 | fda.115 | -3.563 | -5.439 |
| ZINC000000968327 | fda.1747 | -3.563 | -5.439 |
| ZINC000000968327 | fda.1748 | -3.563 | -5.439 |
| ZINC000003831128 | fda.824 | -3.563 | -3.579 |
| ZINC000000896717 | fda.1168 | -3.563 | -3.569 |
| ZINC000053683151 | fda.1189 | -3.562 | -4.399 |
| ZINC000053683151 | fda.1190 | -3.562 | -4.399 |
| ZINC000001530567 | fda.1139 | -3.56 | -3.562 |
| ZINC000001530568 | fda.1140 | -3.56 | -3.562 |
| ZINC000019594557 | fda.755 | -3.56 | -4.375 |
| ZINC000019594557 | fda.756 | -3.56 | -4.375 |
| ZINC000019361042 | fda.762 | -3.56 | -4.375 |
| ZINC000019361042 | fda.763 | -3.56 | -4.375 |
| ZINC000100378061 | fda.530 | -3.559 | -5.903 |
| ZINC000100378061 | fda.531 | -3.559 | -5.903 |
| ZINC000100378061 | fda.532 | -3.559 | -5.903 |
| ZINC000000003876 | fda.313 | -3.559 | -4.045 |
| ZINC000000121480 | fda.1949 | -3.559 | -3.559 |
| ZINC000001995484 | fda.1275 | -3.555 | -4.263 |
| ZINC000000002176 | fda.648 | -3.555 | -3.556 |
| ZINC000000403609 | fda.1179 | -3.555 | -3.556 |
| ZINC000000000456 | fda.1221 | -3.554 | -3.649 |
| ZINC000000154964 | fda.1834 | -3.554 | -3.649 |
| ZINC000000154964 | fda.1835 | -3.554 | -3.649 |
| ZINC000095616601 | fda.17 | -3.553 | -4.795 |
| ZINC000072267023 | fda.1703 | -3.553 | -3.559 |
| ZINC000072267023 | fda.1704 | -3.553 | -3.559 |
| ZINC000003830999 | fda.604 | -3.551 | -4.642 |
| ZINC000003830999 | fda.605 | -3.551 | -4.642 |
| ZINC000004097343 | fda.955 | -3.551 | -5.523 |
| ZINC000004097344 | fda.1094 | -3.551 | -5.523 |
| ZINC000000001411 | fda.245 | -3.551 | -3.551 |
| ZINC000000601250 | fda.1504 | -3.551 | -4.053 |
| ZINC000000601250 | fda.1505 | -3.551 | -4.053 |
| ZINC000003873371 | fda.1092 | -3.551 | -4.053 |
| ZINC000003873371 | fda.1093 | -3.551 | -4.053 |
| ZINC000003873296 | fda.1768 | -3.55 | -4.589 |
| ZINC000000895103 | fda.613 | -3.548 | -3.548 |
| ZINC000001481956 | fda.795 | -3.548 | -3.558 |
| ZINC000001481956 | fda.796 | -3.548 | -3.558 |
| ZINC000004214700 | fda.995 | -3.548 | -3.558 |
| ZINC000004214700 | fda.996 | -3.548 | -3.558 |
| ZINC000004099200 | fda.836 | -3.548 | -5.239 |
| ZINC000004676424 | fda.881 | -3.548 | -5.239 |
| ZINC000001529323 | fda.16 | -3.547 | -6.009 |
| ZINC000011726211 | fda.591 | -3.547 | -3.556 |
| ZINC000011726211 | fda.592 | -3.547 | -3.556 |
| ZINC000000538483 | fda.1367 | -3.545 | -3.963 |
| ZINC000000538483 | fda.1368 | -3.545 | -3.963 |
| ZINC000003816514 | fda.1698 | -3.542 | -3.623 |
| ZINC000038197764 | fda.1019 | -3.54 | -3.633 |
| ZINC000038197764 | fda.1020 | -3.54 | -3.633 |
| ZINC000003607120 | fda.8 | -3.54 | -3.633 |
| ZINC000003607120 | fda.9 | -3.54 | -3.633 |
| ZINC000001530636 | fda.1524 | -3.54 | -4.224 |
| ZINC000245204949 | fda.335 | -3.539 | -3.549 |
| ZINC000005764759 | fda.1331 | -3.539 | -3.549 |
| ZINC000000897002 | fda.1082 | -3.539 | -4.98 |
| ZINC000000897002 | fda.1083 | -3.539 | -4.98 |
| ZINC000000897002 | fda.1084 | -3.539 | -4.98 |
| ZINC000019419017 | fda.770 | -3.536 | -3.774 |
| ZINC000100001964 | fda.1286 | -3.535 | -3.539 |
| ZINC000100001965 | fda.1330 | -3.535 | -3.539 |
| ZINC000019632628 | fda.1899 | -3.533 | -4.714 |
| ZINC000019632628 | fda.1900 | -3.533 | -4.714 |
| ZINC000000538509 | fda.162 | -3.533 | -3.553 |
| ZINC000000538509 | fda.163 | -3.533 | -3.553 |
| ZINC000003008621 | fda.1104 | -3.532 | -3.534 |
| ZINC000100036924 | fda.1105 | -3.531 | -5.158 |
| ZINC000002847375 | fda.1527 | -3.53 | -4.437 |
| ZINC000002847375 | fda.1528 | -3.53 | -4.437 |
| ZINC000000000215 | fda.1837 | -3.53 | -4.437 |
| ZINC000000000215 | fda.1838 | -3.53 | -4.437 |
| ZINC000003816514 | fda.1697 | -3.529 | -3.611 |
| ZINC000000402830 | fda.1174 | -3.529 | -3.53 |
| ZINC000000402830 | fda.1175 | -3.529 | -3.53 |
| ZINC000003920657 | fda.825 | -3.528 | -3.542 |
| ZINC000028957444 | fda.1332 | -3.526 | -3.838 |
| ZINC000022448983 | fda.399 | -3.525 | -4.68 |
| ZINC000022448983 | fda.400 | -3.525 | -4.68 |
| ZINC000022448983 | fda.401 | -3.525 | -4.68 |
| ZINC000003792417 | fda.557 | -3.525 | -5.738 |
| ZINC000022851765 | fda.171 | -3.522 | -4.408 |
| ZINC000022851765 | fda.172 | -3.522 | -4.408 |
| ZINC000022851765 | fda.173 | -3.522 | -4.408 |
| ZINC000022448097 | fda.2104 | -3.522 | -4.408 |
| ZINC000022448097 | fda.2105 | -3.522 | -4.408 |
| ZINC000022448097 | fda.2106 | -3.522 | -4.408 |
| ZINC000095626706 | fda.1317 | -3.521 | -3.731 |
| ZINC000095626706 | fda.1318 | -3.521 | -3.731 |
| ZINC000003812867 | fda.1743 | -3.521 | -4.144 |
| ZINC000019796168 | fda.389 | -3.521 | -3.703 |
| ZINC000019796168 | fda.390 | -3.521 | -3.703 |
| ZINC000019796168 | fda.391 | -3.521 | -3.703 |
| ZINC000000004166 | fda.449 | -3.52 | -5.502 |
| ZINC000000896819 | fda.1081 | -3.52 | -5.502 |
| ZINC000003801919 | fda.1468 | -3.517 | -4.26 |
| ZINC000001482113 | fda.1041 | -3.516 | -3.516 |
| ZINC000003787097 | fda.555 | -3.515 | -3.581 |
| ZINC000022116608 | fda.647 | -3.515 | -5.503 |
| ZINC000022116612 | fda.1167 | -3.515 | -5.503 |
| ZINC000003785268 | fda.1694 | -3.51 | -3.515 |
| ZINC000003799072 | fda.2045 | -3.51 | -3.515 |
| ZINC000003982483 | fda.1891 | -3.509 | -5.921 |
| ZINC000003982483 | fda.1892 | -3.509 | -5.921 |
| ZINC000003982483 | fda.1893 | -3.509 | -5.921 |
| ZINC000022116608 | fda.646 | -3.508 | -5.497 |
| ZINC000022116612 | fda.1166 | -3.508 | -5.497 |
| ZINC000000000242 | fda.684 | -3.508 | -3.512 |
| ZINC000000000242 | fda.685 | -3.508 | -3.512 |
| ZINC000007997952 | fda.1572 | -3.508 | -3.512 |
| ZINC000007997952 | fda.1573 | -3.508 | -3.512 |
| ZINC000019796087 | fda.305 | -3.508 | -3.728 |
| ZINC000019796087 | fda.306 | -3.508 | -3.728 |
| ZINC000019796087 | fda.307 | -3.508 | -3.728 |
| ZINC000000000456 | fda.1221 | -3.507 | -3.602 |
| ZINC000000154964 | fda.1834 | -3.507 | -3.602 |
| ZINC000000154964 | fda.1835 | -3.507 | -3.602 |
| ZINC000019875504 | fda.1901 | -3.506 | -3.91 |
| ZINC000019156872 | fda.1580 | -3.505 | -3.899 |
| ZINC000019156872 | fda.1581 | -3.505 | -3.899 |
| ZINC000019156872 | fda.1582 | -3.505 | -3.899 |
| ZINC000001530569 | fda.1141 | -3.505 | -3.506 |
| ZINC000001530570 | fda.1142 | -3.505 | -3.506 |
| ZINC000058581064 | fda.1199 | -3.504 | -5.473 |
| ZINC000003812306 | fda.2065 | -3.502 | -3.656 |
| ZINC000003806721 | fda.1714 | -3.499 | -5.044 |
| ZINC000003806721 | fda.1715 | -3.499 | -5.044 |
| ZINC000016052277 | fda.1897 | -3.496 | -5.544 |
| ZINC000004083606 | fda.385 | -3.496 | -3.496 |
| ZINC000019875504 | fda.1902 | -3.495 | -3.9 |
| ZINC000001530974 | fda.690 | -3.494 | -3.873 |
| ZINC000001530973 | fda.884 | -3.494 | -3.873 |
| ZINC000014210876 | fda.1798 | -3.493 | -4.221 |
| ZINC000000001792 | fda.446 | -3.492 | -3.492 |
| ZINC000084441937 | fda.1908 | -3.488 | -5.115 |
| ZINC000084441937 | fda.1910 | -3.488 | -5.115 |
| ZINC000000968328 | fda.402 | -3.488 | -4.822 |
| ZINC000000968330 | fda.1172 | -3.488 | -4.822 |
| ZINC000003875483 | fda.548 | -3.488 | -4.575 |
| ZINC000003875483 | fda.549 | -3.488 | -4.575 |
| ZINC000003875483 | fda.550 | -3.488 | -4.575 |
| ZINC000000025958 | fda.61 | -3.485 | -5.671 |
| ZINC000001530575 | fda.247 | -3.485 | -3.485 |
| ZINC000019796080 | fda.714 | -3.485 | -4.597 |
| ZINC000019796080 | fda.715 | -3.485 | -4.597 |
| ZINC000019796080 | fda.716 | -3.485 | -4.597 |
| ZINC000003826253 | fda.2057 | -3.484 | -4.6 |
| ZINC000008220878 | fda.1586 | -3.484 | -3.495 |
| ZINC000008220878 | fda.1587 | -3.484 | -3.495 |
| ZINC000033943508 | fda.243 | -3.483 | -3.487 |
| ZINC000000537805 | fda.580 | -3.482 | -3.482 |
| ZINC000003812862 | fda.431 | -3.481 | -4.535 |
| ZINC000094566093 | fda.1304 | -3.481 | -3.914 |
| ZINC000053683151 | fda.1189 | -3.48 | -4.318 |
| ZINC000053683151 | fda.1190 | -3.48 | -4.318 |
| ZINC000094566092 | fda.1316 | -3.478 | -3.926 |
| ZINC000035902489 | fda.1537 | -3.478 | -3.814 |
| ZINC000100013500 | fda.93 | -3.478 | -3.48 |
| ZINC000001482197 | fda.456 | -3.477 | -5.286 |
| ZINC000000896666 | fda.81 | -3.477 | -5.286 |
| ZINC000053084692 | fda.74 | -3.476 | -4.254 |
| ZINC000100378061 | fda.530 | -3.475 | -5.81 |
| ZINC000100378061 | fda.532 | -3.475 | -5.81 |
| ZINC000003831405 | fda.28 | -3.475 | -5.752 |
| ZINC000003831405 | fda.29 | -3.475 | -5.752 |
| ZINC000004212945 | fda.837 | -3.474 | -3.474 |
| ZINC000095551509 | fda.503 | -3.471 | -3.477 |
| ZINC000100378061 | fda.531 | -3.468 | -4.092 |
| ZINC000001530636 | fda.1523 | -3.466 | -4.874 |
| ZINC000000000882 | fda.642 | -3.464 | -5.461 |
| ZINC000000000882 | fda.643 | -3.464 | -5.461 |
| ZINC000000009073 | fda.1036 | -3.463 | -5.027 |
| ZINC000000009073 | fda.1037 | -3.463 | -5.027 |
| ZINC000000009073 | fda.1038 | -3.463 | -5.027 |
| ZINC000001996117 | fda.980 | -3.462 | -3.464 |
| ZINC000000000507 | fda.1564 | -3.461 | -4.523 |
| ZINC000000403079 | fda.1678 | -3.461 | -4.523 |
| ZINC000003830215 | fda.1246 | -3.456 | -4.221 |
| ZINC000003816514 | fda.1697 | -3.455 | -4.671 |
| ZINC000001533877 | fda.821 | -3.455 | -4.625 |
| ZINC000014210876 | fda.1797 | -3.452 | -4.726 |
| ZINC000019362735 | fda.657 | -3.452 | -3.847 |
| ZINC000019362735 | fda.658 | -3.452 | -3.847 |
| ZINC000019362735 | fda.659 | -3.452 | -3.847 |
| ZINC000019362737 | fda.660 | -3.452 | -3.847 |
| ZINC000019362737 | fda.661 | -3.452 | -3.847 |
| ZINC000019362737 | fda.662 | -3.452 | -3.847 |
| ZINC000003843198 | fda.1002 | -3.452 | -3.454 |
| ZINC000084400879 | fda.302 | -3.451 | -4.143 |
| ZINC000084400879 | fda.303 | -3.451 | -4.143 |
| ZINC000084400879 | fda.304 | -3.451 | -4.143 |
| ZINC000019796087 | fda.305 | -3.451 | -4.143 |
| ZINC000019796087 | fda.306 | -3.451 | -4.143 |
| ZINC000019796087 | fda.307 | -3.451 | -4.143 |
| ZINC000013911941 | fda.2076 | -3.45 | -3.45 |
| ZINC000100378061 | fda.531 | -3.449 | -5.784 |
| ZINC000000896731 | fda.1355 | -3.449 | -5.51 |
| ZINC000000896731 | fda.1356 | -3.449 | -5.51 |
| ZINC000000000850 | fda.1146 | -3.449 | -5.51 |
| ZINC000000000850 | fda.1147 | -3.449 | -5.51 |
| ZINC000019796158 | fda.726 | -3.446 | -5.193 |
| ZINC000019796158 | fda.727 | -3.446 | -5.193 |
| ZINC000000896740 | fda.1497 | -3.445 | -4.168 |
| ZINC000000896740 | fda.1498 | -3.445 | -4.168 |
| ZINC000000607971 | fda.1506 | -3.445 | -4.168 |
| ZINC000000607971 | fda.1507 | -3.445 | -4.168 |
| ZINC000001532529 | fda.617 | -3.443 | -3.443 |
| ZINC000000005823 | fda.44 | -3.44 | -4.706 |
| ZINC000000000083 | fda.754 | -3.439 | -3.439 |
| ZINC000003830314 | fda.1815 | -3.439 | -3.439 |
| ZINC000004213946 | fda.951 | -3.437 | -5.461 |
| ZINC000005844792 | fda.1311 | -3.437 | -5.461 |
| ZINC000001995484 | fda.1276 | -3.436 | -4.145 |
| ZINC000001530736 | fda.806 | -3.435 | -3.973 |
| ZINC000001530736 | fda.807 | -3.435 | -3.973 |
| ZINC000001530737 | fda.64 | -3.435 | -3.973 |
| ZINC000001530737 | fda.65 | -3.435 | -3.973 |
| ZINC000003792417 | fda.557 | -3.434 | -5.648 |
| ZINC000003873295 | fda.493 | -3.433 | -4.472 |
| ZINC000043200832 | fda.1848 | -3.431 | -4.101 |
| ZINC000043200832 | fda.1849 | -3.431 | -4.101 |
| ZINC000000607986 | fda.63 | -3.429 | -5.453 |
| ZINC000014879992 | fda.732 | -3.428 | -3.464 |
| ZINC000014879992 | fda.734 | -3.428 | -3.464 |
| ZINC000014879992 | fda.735 | -3.428 | -3.464 |
| ZINC000001530975 | fda.888 | -3.428 | -4.377 |
| ZINC000001530975 | fda.889 | -3.428 | -4.377 |
| ZINC000000005823 | fda.42 | -3.426 | -3.851 |
| ZINC000000005823 | fda.43 | -3.426 | -3.851 |
| ZINC000068204830 | fda.215 | -3.424 | -4.292 |
| ZINC000004095858 | fda.250 | -3.422 | -3.422 |
| ZINC000002847375 | fda.1527 | -3.421 | -4.328 |
| ZINC000002847375 | fda.1528 | -3.421 | -4.328 |
| ZINC000000000215 | fda.1837 | -3.421 | -4.328 |
| ZINC000000000215 | fda.1838 | -3.421 | -4.328 |
| ZINC000001530713 | fda.788 | -3.421 | -4.146 |
| ZINC000000596731 | fda.296 | -3.419 | -4.967 |
| ZINC000000596731 | fda.297 | -3.419 | -4.967 |
| ZINC000003830960 | fda.83 | -3.418 | -3.418 |
| ZINC000003830958 | fda.168 | -3.418 | -3.418 |
| ZINC000003918453 | fda.1456 | -3.418 | -4.918 |
| ZINC000000403533 | fda.1176 | -3.417 | -4.538 |
| ZINC000000403533 | fda.1177 | -3.417 | -4.538 |
| ZINC000000403533 | fda.1178 | -3.417 | -4.538 |
| ZINC000001280665 | fda.412 | -3.414 | -5.202 |
| ZINC000001280665 | fda.413 | -3.414 | -5.202 |
| ZINC000001280665 | fda.414 | -3.414 | -5.202 |
| ZINC000008214614 | fda.1561 | -3.412 | -3.412 |
| ZINC000004099009 | fda.993 | -3.411 | -3.411 |
| ZINC000000020228 | fda.326 | -3.408 | -4.065 |
| ZINC000000057206 | fda.988 | -3.408 | -4.065 |
| ZINC000001530618 | fda.929 | -3.404 | -3.41 |
| ZINC000001530617 | fda.1138 | -3.404 | -3.41 |
| ZINC000003818808 | fda.491 | -3.402 | -5.743 |
| ZINC000001999441 | fda.489 | -3.401 | -5.424 |
| ZINC000030691727 | fda.1340 | -3.401 | -3.401 |
| ZINC000014210876 | fda.1798 | -3.401 | -4.694 |
| ZINC000003976838 | fda.1761 | -3.398 | -4.301 |
| ZINC000003976838 | fda.1762 | -3.398 | -4.301 |
| ZINC000004097426 | fda.957 | -3.398 | -4.616 |
| ZINC000004097426 | fda.958 | -3.398 | -4.616 |
| ZINC000004097426 | fda.959 | -3.398 | -4.616 |
| ZINC000019796087 | fda.305 | -3.397 | -3.617 |
| ZINC000019796087 | fda.306 | -3.397 | -3.617 |
| ZINC000019796087 | fda.307 | -3.397 | -3.617 |
| ZINC000053683151 | fda.1189 | -3.395 | -3.574 |
| ZINC000053683151 | fda.1190 | -3.395 | -3.574 |
| ZINC000053683151 | fda.1191 | -3.395 | -3.574 |
| ZINC000019144226 | fda.1588 | -3.395 | -3.431 |
| ZINC000019144231 | fda.1863 | -3.395 | -3.431 |
| ZINC000013537284 | fda.749 | -3.388 | -4.422 |
| ZINC000019364229 | fda.669 | -3.388 | -3.852 |
| ZINC000019364229 | fda.670 | -3.388 | -3.852 |
| ZINC000019364229 | fda.671 | -3.388 | -3.852 |
| ZINC000019364230 | fda.776 | -3.388 | -3.852 |
| ZINC000019364230 | fda.777 | -3.388 | -3.852 |
| ZINC000019364230 | fda.778 | -3.388 | -3.852 |
| ZINC000019166988 | fda.1574 | -3.386 | -4.179 |
| ZINC000006409735 | fda.1512 | -3.381 | -4.171 |
| ZINC000100036924 | fda.1105 | -3.38 | -5.008 |
| ZINC000100013500 | fda.93 | -3.377 | -3.379 |
| ZINC000019632668 | fda.707 | -3.373 | -3.721 |
| ZINC000019632668 | fda.708 | -3.373 | -3.721 |
| ZINC000019632670 | fda.737 | -3.373 | -3.721 |
| ZINC000019632670 | fda.738 | -3.373 | -3.721 |
| ZINC000003826253 | fda.2057 | -3.372 | -4.488 |
| ZINC000027428713 | fda.1302 | -3.372 | -3.372 |
| ZINC000000113415 | fda.584 | -3.372 | -3.374 |
| ZINC000000014007 | fda.1593 | -3.372 | -3.374 |
| ZINC000003807804 | fda.4 | -3.371 | -3.769 |
| ZINC000019364229 | fda.669 | -3.37 | -3.781 |
| ZINC000019364229 | fda.670 | -3.37 | -3.781 |
| ZINC000019364229 | fda.671 | -3.37 | -3.781 |
| ZINC000019364230 | fda.776 | -3.37 | -3.781 |
| ZINC000019364230 | fda.777 | -3.37 | -3.781 |
| ZINC000019364230 | fda.778 | -3.37 | -3.781 |
| ZINC000087515509 | fda.1287 | -3.37 | -4.947 |
| ZINC000095564694 | fda.2095 | -3.369 | -4.615 |
| ZINC000095564694 | fda.2096 | -3.369 | -4.615 |
| ZINC000095564694 | fda.2097 | -3.369 | -4.615 |
| ZINC000043450326 | fda.1906 | -3.367 | -4.405 |
| ZINC000043450324 | fda.1920 | -3.367 | -4.405 |
| ZINC000001530701 | fda.1629 | -3.366 | -3.366 |
| ZINC000000155905 | fda.505 | -3.363 | -3.375 |
| ZINC000003831551 | fda.68 | -3.363 | -3.375 |
| ZINC000003875259 | fda.961 | -3.357 | -3.357 |
| ZINC000019419017 | fda.771 | -3.356 | -3.594 |
| ZINC000019419017 | fda.772 | -3.356 | -3.594 |
| ZINC000003873921 | fda.363 | -3.355 | -5.696 |
| ZINC000004213946 | fda.950 | -3.354 | -5.378 |
| ZINC000005844792 | fda.1310 | -3.354 | -5.378 |
| ZINC000000607986 | fda.62 | -3.354 | -5.378 |
| ZINC000003920266 | fda.1888 | -3.354 | -3.381 |
| ZINC000013545634 | fda.703 | -3.354 | -5.075 |
| ZINC000013545634 | fda.704 | -3.354 | -5.075 |
| ZINC000013545636 | fda.387 | -3.354 | -5.075 |
| ZINC000013545636 | fda.388 | -3.354 | -5.075 |
| ZINC000008214619 | fda.827 | -3.352 | -3.352 |
| ZINC000000601301 | fda.320 | -3.349 | -3.416 |
| ZINC000000897222 | fda.1658 | -3.349 | -3.416 |
| ZINC000004097427 | fda.1215 | -3.349 | -4.215 |
| ZINC000001533877 | fda.820 | -3.348 | -3.439 |
| ZINC000052971887 | fda.1187 | -3.348 | -3.927 |
| ZINC000100070954 | fda.76 | -3.348 | -3.371 |
| ZINC000000895042 | fda.231 | -3.347 | -3.361 |
| ZINC000006661227 | fda.1196 | -3.344 | -3.779 |
| ZINC000001530600 | fda.1144 | -3.342 | -3.637 |
| ZINC000013986658 | fda.542 | -3.341 | -5.338 |
| ZINC000006716957 | fda.870 | -3.34 | -4.265 |
| ZINC000033965961 | fda.2034 | -3.337 | -4.791 |
| ZINC000033965961 | fda.2035 | -3.337 | -4.791 |
| ZINC000003920266 | fda.1887 | -3.335 | -3.362 |
| ZINC000001482113 | fda.1041 | -3.335 | -3.335 |
| ZINC000068204830 | fda.215 | -3.333 | -4.636 |
| ZINC000100037020 | fda.1533 | -3.331 | -4.041 |
| ZINC000100037020 | fda.1534 | -3.331 | -4.041 |
| ZINC000068247389 | fda.1999 | -3.33 | -3.869 |
| ZINC000068247389 | fda.2000 | -3.33 | -3.869 |
| ZINC000013986658 | fda.541 | -3.328 | -5.326 |
| ZINC000001532529 | fda.617 | -3.326 | -3.326 |
| ZINC000014879972 | fda.731 | -3.326 | -4.874 |
| ZINC000004099009 | fda.993 | -3.325 | -3.325 |
| ZINC000001530803 | fda.1617 | -3.323 | -3.323 |
| ZINC000008220878 | fda.1586 | -3.322 | -3.333 |
| ZINC000008220878 | fda.1587 | -3.322 | -3.333 |
| ZINC000001530816 | fda.676 | -3.322 | -3.384 |
| ZINC000001530816 | fda.677 | -3.322 | -3.384 |
| ZINC000001530817 | fda.801 | -3.322 | -3.384 |
| ZINC000001530817 | fda.802 | -3.322 | -3.384 |
| ZINC000000895034 | fda.1379 | -3.321 | -3.321 |
| ZINC000000968275 | fda.1428 | -3.321 | -3.321 |
| ZINC000003831586 | fda.1817 | -3.321 | -3.321 |
| ZINC000013298313 | fda.593 | -3.32 | -5.828 |
| ZINC000000596881 | fda.310 | -3.319 | -4.042 |
| ZINC000000596881 | fda.311 | -3.319 | -4.042 |
| ZINC000000643055 | fda.1499 | -3.319 | -4.042 |
| ZINC000000643055 | fda.1500 | -3.319 | -4.042 |
| ZINC000001530929 | fda.1950 | -3.319 | -4.731 |
| ZINC000001530929 | fda.1951 | -3.319 | -4.731 |
| ZINC000003876068 | fda.1976 | -3.319 | -4.731 |
| ZINC000003876068 | fda.1977 | -3.319 | -4.731 |
| ZINC000003831128 | fda.824 | -3.317 | -3.333 |
| ZINC000043195697 | fda.1926 | -3.317 | -3.562 |
| ZINC000035653007 | fda.1545 | -3.317 | -3.997 |
| ZINC000043200832 | fda.1848 | -3.317 | -3.987 |
| ZINC000043200832 | fda.1849 | -3.317 | -3.987 |
| ZINC000003873295 | fda.493 | -3.316 | -3.428 |
| ZINC000003873295 | fda.494 | -3.316 | -3.428 |
| ZINC000000001982 | fda.1802 | -3.316 | -3.316 |
| ZINC000001548097 | fda.1857 | -3.314 | -5.588 |
| ZINC000001548097 | fda.1858 | -3.314 | -5.588 |
| ZINC000002561203 | fda.1260 | -3.314 | -3.315 |
| ZINC000043194409 | fda.2040 | -3.314 | -3.318 |
| ZINC000004097427 | fda.1216 | -3.312 | -3.468 |
| ZINC000001530579 | fda.1086 | -3.31 | -4.885 |
| ZINC000001530580 | fda.1088 | -3.31 | -4.885 |
| ZINC000003871978 | fda.1729 | -3.31 | -3.31 |
| ZINC000003800008 | fda.193 | -3.309 | -3.542 |
| ZINC000003800008 | fda.194 | -3.309 | -3.542 |
| ZINC000003800008 | fda.195 | -3.309 | -3.542 |
| ZINC000000020237 | fda.1539 | -3.307 | -3.327 |
| ZINC000000020237 | fda.1540 | -3.307 | -3.327 |
| ZINC000003813042 | fda.1814 | -3.304 | -4.815 |
| ZINC000003808779 | fda.974 | -3.303 | -5.104 |
| ZINC000003780893 | fda.248 | -3.303 | -3.303 |
| ZINC000004658290 | fda.14 | -3.303 | -4.35 |
| ZINC000001530697 | fda.691 | -3.301 | -3.302 |
| ZINC000001530697 | fda.693 | -3.301 | -3.302 |
| ZINC000001530695 | fda.1626 | -3.301 | -3.302 |
| ZINC000003938695 | fda.826 | -3.301 | -3.301 |
| ZINC000000000931 | fda.1670 | -3.3 | -5.55 |
| ZINC000000000931 | fda.1671 | -3.3 | -5.55 |
| ZINC000000537791 | fda.1369 | -3.297 | -3.297 |
| ZINC000003831050 | fda.626 | -3.297 | -3.297 |
| ZINC000003831051 | fda.627 | -3.297 | -3.297 |
| ZINC000011681534 | fda.2021 | -3.296 | -5.32 |
| ZINC000000895034 | fda.1380 | -3.296 | -3.296 |
| ZINC000004097426 | fda.957 | -3.296 | -4.513 |
| ZINC000004097426 | fda.958 | -3.296 | -4.513 |
| ZINC000004097426 | fda.959 | -3.296 | -4.513 |
| ZINC000001530935 | fda.682 | -3.295 | -4.995 |
| ZINC000004618208 | fda.873 | -3.295 | -4.995 |
| ZINC000003914596 | fda.551 | -3.29 | -5.55 |
| ZINC000003914596 | fda.552 | -3.29 | -5.55 |
| ZINC000003914596 | fda.553 | -3.29 | -5.55 |
| ZINC000004629876 | fda.256 | -3.285 | -3.285 |
| ZINC000000537928 | fda.851 | -3.285 | -3.314 |
| ZINC000019632668 | fda.707 | -3.284 | -3.765 |
| ZINC000019632668 | fda.708 | -3.284 | -3.765 |
| ZINC000019632670 | fda.737 | -3.284 | -3.765 |
| ZINC000019632670 | fda.738 | -3.284 | -3.765 |
| ZINC000022016976 | fda.1158 | -3.282 | -3.314 |
| ZINC000022016976 | fda.1159 | -3.282 | -3.314 |
| ZINC000022016976 | fda.1160 | -3.282 | -3.314 |
| ZINC000022016981 | fda.1161 | -3.282 | -3.314 |
| ZINC000022016981 | fda.1162 | -3.282 | -3.314 |
| ZINC000022016981 | fda.1163 | -3.282 | -3.314 |
| ZINC000052955754 | fda.129 | -3.278 | -5.617 |
| ZINC000052955754 | fda.130 | -3.278 | -5.617 |
| ZINC000052955754 | fda.131 | -3.278 | -5.617 |
| ZINC000003827556 | fda.1972 | -3.275 | -4.841 |
| ZINC000001530912 | fda.809 | -3.275 | -3.569 |
| ZINC000001530912 | fda.810 | -3.275 | -3.569 |
| ZINC000001530912 | fda.811 | -3.275 | -3.569 |
| ZINC000012402836 | fda.1446 | -3.274 | -5.287 |
| ZINC000043195697 | fda.1924 | -3.273 | -3.518 |
| ZINC000004215234 | fda.2016 | -3.269 | -3.269 |
| ZINC000004258316 | fda.180 | -3.269 | -4.45 |
| ZINC000001530652 | fda.459 | -3.261 | -3.31 |
| ZINC000001530652 | fda.460 | -3.261 | -3.31 |
| ZINC000001530697 | fda.692 | -3.261 | -3.261 |
| ZINC000001530695 | fda.1627 | -3.261 | -3.261 |
| ZINC000001530695 | fda.1628 | -3.261 | -3.261 |
| ZINC000003977764 | fda.653 | -3.26 | -3.261 |
| ZINC000004097310 | fda.901 | -3.26 | -3.261 |
| ZINC000002019953 | fda.903 | -3.258 | -3.308 |
| ZINC000002019954 | fda.937 | -3.258 | -3.308 |
| ZINC000026985532 | fda.355 | -3.256 | -3.27 |
| ZINC000008214692 | fda.1111 | -3.255 | -4.609 |
| ZINC000003813010 | fda.104 | -3.252 | -5.037 |
| ZINC000003813010 | fda.105 | -3.252 | -5.037 |
| ZINC000000538273 | fda.952 | -3.251 | -4.793 |
| ZINC000000538273 | fda.953 | -3.251 | -4.793 |
| ZINC000000537891 | fda.1373 | -3.251 | -4.793 |
| ZINC000000537891 | fda.1374 | -3.251 | -4.793 |
| ZINC000004693575 | fda.323 | -3.246 | -4.642 |
| ZINC000004693574 | fda.1217 | -3.246 | -4.642 |
| ZINC000003873936 | fda.1732 | -3.244 | -4.402 |
| ZINC000003873936 | fda.1733 | -3.244 | -4.402 |
| ZINC000011677857 | fda.479 | -3.243 | -5.154 |
| ZINC000049841054 | fda.1203 | -3.242 | -3.245 |
| ZINC000164528615 | fda.2089 | -3.236 | -3.239 |
| ZINC000004097343 | fda.955 | -3.234 | -5.206 |
| ZINC000004097344 | fda.1094 | -3.234 | -5.206 |
| ZINC000019632668 | fda.707 | -3.233 | -3.714 |
| ZINC000019632668 | fda.708 | -3.233 | -3.714 |
| ZINC000019632670 | fda.737 | -3.233 | -3.714 |
| ZINC000019632670 | fda.738 | -3.233 | -3.714 |
| ZINC000000395010 | fda.534 | -3.232 | -4.56 |
| ZINC000003960338 | fda.134 | -3.23 | -3.23 |
| ZINC000000057533 | fda.985 | -3.23 | -3.238 |
| ZINC000000057532 | fda.1018 | -3.23 | -3.238 |
| ZINC000011681534 | fda.2022 | -3.229 | -5.252 |
| ZINC000003993846 | fda.2072 | -3.229 | -3.234 |
| ZINC000036294079 | fda.678 | -3.228 | -4.309 |
| ZINC000035999642 | fda.745 | -3.228 | -4.309 |
| ZINC000003812888 | fda.1683 | -3.227 | -3.232 |
| ZINC000003812888 | fda.1684 | -3.227 | -3.232 |
| ZINC000001530694 | fda.464 | -3.227 | -3.227 |
| ZINC000100013500 | fda.92 | -3.226 | -3.228 |
| ZINC000001533877 | fda.820 | -3.226 | -4.396 |
| ZINC000003801163 | fda.1709 | -3.224 | -4.098 |
| ZINC000022448696 | fda.1825 | -3.22 | -3.611 |
| ZINC000022448696 | fda.1826 | -3.22 | -3.611 |
| ZINC000001530935 | fda.682 | -3.218 | -3.252 |
| ZINC000004618208 | fda.873 | -3.218 | -3.252 |
| ZINC000004074875 | fda.1214 | -3.216 | -3.372 |
| ZINC000087515509 | fda.1287 | -3.216 | -4.088 |
| ZINC000001996117 | fda.981 | -3.214 | -3.216 |
| ZINC000000057522 | fda.1068 | -3.214 | -3.214 |
| ZINC000000020228 | fda.325 | -3.213 | -3.87 |
| ZINC000000057206 | fda.987 | -3.213 | -3.87 |
| ZINC000003785268 | fda.1694 | -3.212 | -3.217 |
| ZINC000003799072 | fda.2045 | -3.212 | -3.217 |
| ZINC000003830842 | fda.561 | -3.211 | -3.211 |
| ZINC000000896543 | fda.1078 | -3.211 | -3.211 |
| ZINC000011615927 | fda.480 | -3.206 | -3.208 |
| ZINC000011615928 | fda.481 | -3.206 | -3.208 |
| ZINC000004099200 | fda.836 | -3.206 | -5.292 |
| ZINC000004676424 | fda.881 | -3.206 | -5.292 |
| ZINC000021297660 | fda.121 | -3.202 | -4.894 |
| ZINC000021297660 | fda.122 | -3.202 | -4.894 |
| ZINC000001530816 | fda.677 | -3.202 | -3.263 |
| ZINC000001530817 | fda.802 | -3.202 | -3.263 |
| ZINC000095619105 | fda.1193 | -3.201 | -3.829 |
| ZINC000001532805 | fda.263 | -3.199 | -3.201 |
| ZINC000012503187 | fda.568 | -3.199 | -4.112 |
| ZINC000070466416 | fda.536 | -3.198 | -4.748 |
| ZINC000000601317 | fda.2043 | -3.198 | -5.366 |
| ZINC000095619105 | fda.1194 | -3.197 | -3.824 |
| ZINC000095619105 | fda.1195 | -3.197 | -3.824 |
| ZINC000001529323 | fda.16 | -3.195 | -3.26 |
| ZINC000000074836 | fda.319 | -3.195 | -5.857 |
| ZINC000019632718 | fda.111 | -3.194 | -3.194 |
| ZINC000019632713 | fda.126 | -3.194 | -3.194 |
| ZINC000003797541 | fda.1681 | -3.191 | -5.192 |
| ZINC000019156872 | fda.1580 | -3.191 | -3.659 |
| ZINC000019156872 | fda.1581 | -3.191 | -3.659 |
| ZINC000019156872 | fda.1582 | -3.191 | -3.659 |
| ZINC000019364229 | fda.669 | -3.19 | -3.655 |
| ZINC000019364229 | fda.670 | -3.19 | -3.655 |
| ZINC000019364229 | fda.671 | -3.19 | -3.655 |
| ZINC000019364230 | fda.776 | -3.19 | -3.655 |
| ZINC000019364230 | fda.777 | -3.19 | -3.655 |
| ZINC000019364230 | fda.778 | -3.19 | -3.655 |
| ZINC000003920657 | fda.825 | -3.19 | -3.204 |
| ZINC000001530935 | fda.682 | -3.19 | -3.224 |
| ZINC000004618208 | fda.873 | -3.19 | -3.224 |
| ZINC000052971887 | fda.1187 | -3.19 | -3.768 |
| ZINC000004074875 | fda.1213 | -3.189 | -3.345 |
| ZINC000021303210 | fda.1447 | -3.189 | -4.69 |
| ZINC000021303210 | fda.1448 | -3.189 | -4.69 |
| ZINC000000896740 | fda.1497 | -3.187 | -3.91 |
| ZINC000000896740 | fda.1498 | -3.187 | -3.91 |
| ZINC000000607971 | fda.1506 | -3.187 | -3.91 |
| ZINC000000607971 | fda.1507 | -3.187 | -3.91 |
| ZINC000006661227 | fda.1197 | -3.187 | -4.907 |
| ZINC000006661227 | fda.1198 | -3.187 | -4.907 |
| ZINC000022448696 | fda.1825 | -3.181 | -3.823 |
| ZINC000022448696 | fda.1826 | -3.181 | -3.823 |
| ZINC000001530728 | fda.543 | -3.178 | -3.178 |
| ZINC000001530728 | fda.544 | -3.178 | -3.178 |
| ZINC000000897002 | fda.1082 | -3.176 | -3.23 |
| ZINC000000897002 | fda.1083 | -3.176 | -3.23 |
| ZINC000000897002 | fda.1084 | -3.176 | -3.23 |
| ZINC000004693575 | fda.324 | -3.173 | -4.883 |
| ZINC000004693574 | fda.1218 | -3.173 | -4.883 |
| ZINC000014879992 | fda.733 | -3.173 | -3.199 |
| ZINC000001530912 | fda.809 | -3.168 | -3.738 |
| ZINC000001530912 | fda.810 | -3.168 | -3.738 |
| ZINC000001530912 | fda.811 | -3.168 | -3.738 |
| ZINC000003830212 | fda.1502 | -3.166 | -5.179 |
| ZINC000003830212 | fda.1503 | -3.166 | -5.179 |
| ZINC000001530636 | fda.1523 | -3.166 | -3.85 |
| ZINC000021982937 | fda.2024 | -3.166 | -4.62 |
| ZINC000021982937 | fda.2025 | -3.166 | -4.62 |
| ZINC000000402954 | fda.965 | -3.166 | -4.983 |
| ZINC000000402954 | fda.966 | -3.166 | -4.983 |
| ZINC000000402954 | fda.967 | -3.166 | -4.983 |
| ZINC000000002273 | fda.938 | -3.164 | -5.376 |
| ZINC000000000469 | fda.1514 | -3.164 | -5.376 |
| ZINC000000000506 | fda.290 | -3.164 | -3.165 |
| ZINC000003931840 | fda.395 | -3.164 | -3.165 |
| ZINC000003818726 | fda.184 | -3.161 | -3.183 |
| ZINC000087515509 | fda.1287 | -3.158 | -4.35 |
| ZINC000014210876 | fda.1798 | -3.156 | -4.588 |
| ZINC000009302201 | fda.397 | -3.154 | -3.158 |
| ZINC000035902489 | fda.1537 | -3.149 | -3.645 |
| ZINC000003977777 | fda.1277 | -3.148 | -3.148 |
| ZINC000095616937 | fda.1934 | -3.148 | -3.158 |
| ZINC000003929508 | fda.1436 | -3.148 | -4.36 |
| ZINC000019632668 | fda.707 | -3.147 | -3.496 |
| ZINC000019632668 | fda.708 | -3.147 | -3.496 |
| ZINC000019632670 | fda.737 | -3.147 | -3.496 |
| ZINC000019632670 | fda.738 | -3.147 | -3.496 |
| ZINC000004640636 | fda.874 | -3.142 | -4.811 |
| ZINC000004640636 | fda.875 | -3.142 | -4.811 |
| ZINC000095616603 | fda.1376 | -3.141 | -5.563 |
| ZINC000000000973 | fda.474 | -3.141 | -4.467 |
| ZINC000002519740 | fda.1124 | -3.139 | -4.547 |
| ZINC000094566092 | fda.1316 | -3.138 | -3.585 |
| ZINC000150338698 | fda.1039 | -3.137 | -3.649 |
| ZINC000003830993 | fda.1462 | -3.137 | -4.125 |
| ZINC000033965961 | fda.2034 | -3.131 | -4.585 |
| ZINC000033965961 | fda.2035 | -3.131 | -4.585 |
| ZINC000004655029 | fda.876 | -3.131 | -3.135 |
| ZINC000004655029 | fda.877 | -3.131 | -3.135 |
| ZINC000001529323 | fda.16 | -3.127 | -4.563 |
| ZINC000003798757 | fda.1740 | -3.127 | -3.854 |
| ZINC000004693575 | fda.323 | -3.125 | -4.894 |
| ZINC000004693574 | fda.1217 | -3.125 | -4.894 |
| ZINC000019632618 | fda.757 | -3.124 | -3.879 |
| ZINC000019632618 | fda.758 | -3.124 | -3.879 |
| ZINC000019632618 | fda.759 | -3.124 | -3.879 |
| ZINC000001999441 | fda.490 | -3.123 | -5.147 |
| ZINC000000000456 | fda.1220 | -3.122 | -3.217 |
| ZINC000000000456 | fda.1222 | -3.122 | -3.217 |
| ZINC000000154964 | fda.1836 | -3.122 | -3.217 |
| ZINC000009164421 | fda.2017 | -3.119 | -3.121 |
| ZINC000003787097 | fda.555 | -3.117 | -4.451 |
| ZINC000000596731 | fda.296 | -3.117 | -5.159 |
| ZINC000000596731 | fda.297 | -3.117 | -5.159 |
| ZINC000001530579 | fda.1087 | -3.116 | -4.69 |
| ZINC000001530580 | fda.1089 | -3.116 | -4.69 |
| ZINC000004097404 | fda.940 | -3.113 | -3.117 |
| ZINC000043195697 | fda.1924 | -3.113 | -3.794 |
| ZINC000003914596 | fda.552 | -3.112 | -3.125 |
| ZINC000003914596 | fda.553 | -3.112 | -3.125 |
| ZINC000018324776 | fda.701 | -3.111 | -3.882 |
| ZINC000003794711 | fda.407 | -3.11 | -5.156 |
| ZINC000003794711 | fda.408 | -3.11 | -5.156 |
| ZINC000003794711 | fda.409 | -3.11 | -5.156 |
| ZINC000003943279 | fda.1420 | -3.108 | -4.579 |
| ZINC000000002041 | fda.211 | -3.106 | -3.108 |
| ZINC000000113415 | fda.584 | -3.105 | -3.107 |
| ZINC000000014007 | fda.1593 | -3.105 | -3.107 |
| ZINC000019144226 | fda.1588 | -3.103 | -3.139 |
| ZINC000019144231 | fda.1863 | -3.103 | -3.139 |
| ZINC000245204949 | fda.335 | -3.1 | -3.109 |
| ZINC000005764759 | fda.1331 | -3.1 | -3.109 |
| ZINC000000000856 | fda.1153 | -3.099 | -5.407 |
| ZINC000000897258 | fda.2012 | -3.099 | -5.407 |
| ZINC000001530621 | fda.813 | -3.097 | -4.509 |
| ZINC000000020259 | fda.706 | -3.097 | -5.758 |
| ZINC000000402954 | fda.965 | -3.095 | -4.844 |
| ZINC000000402954 | fda.966 | -3.095 | -4.844 |
| ZINC000000402954 | fda.967 | -3.095 | -4.844 |
| ZINC000002599970 | fda.57 | -3.094 | -5.402 |
| ZINC000000897256 | fda.2010 | -3.094 | -5.402 |
| ZINC000003797541 | fda.1681 | -3.093 | -5.094 |
| ZINC000000020228 | fda.325 | -3.091 | -3.748 |
| ZINC000000057206 | fda.987 | -3.091 | -3.748 |
| ZINC000001530736 | fda.806 | -3.09 | -3.396 |
| ZINC000001530736 | fda.807 | -3.09 | -3.396 |
| ZINC000001530737 | fda.64 | -3.09 | -3.396 |
| ZINC000001530737 | fda.65 | -3.09 | -3.396 |
| ZINC000009224016 | fda.124 | -3.087 | -5.152 |
| ZINC000009224016 | fda.125 | -3.087 | -5.152 |
| ZINC000072316335 | fda.1928 | -3.086 | -4.487 |
| ZINC000002599970 | fda.56 | -3.086 | -5.394 |
| ZINC000000897256 | fda.2009 | -3.086 | -5.394 |
| ZINC000000968326 | fda.114 | -3.084 | -5.002 |
| ZINC000000968326 | fda.115 | -3.084 | -5.002 |
| ZINC000000968327 | fda.1747 | -3.084 | -5.002 |
| ZINC000000968327 | fda.1748 | -3.084 | -5.002 |
| ZINC000100004343 | fda.1321 | -3.082 | -3.106 |
| ZINC000100004345 | fda.1322 | -3.082 | -3.106 |
| ZINC000003801919 | fda.1466 | -3.081 | -3.824 |
| ZINC000003801919 | fda.1467 | -3.081 | -3.824 |
| ZINC000000389149 | fda.805 | -3.08 | -3.082 |
| ZINC000000000494 | fda.1485 | -3.08 | -3.082 |
| ZINC000013298313 | fda.594 | -3.076 | -5.559 |
| ZINC000003861768 | fda.135 | -3.075 | -5.591 |
| ZINC000000968326 | fda.114 | -3.075 | -4.994 |
| ZINC000000968326 | fda.115 | -3.075 | -4.994 |
| ZINC000000968327 | fda.1747 | -3.075 | -4.994 |
| ZINC000000968327 | fda.1748 | -3.075 | -4.994 |
| ZINC000000020237 | fda.1539 | -3.074 | -5.074 |
| ZINC000000020237 | fda.1540 | -3.074 | -5.074 |
| ZINC000052971887 | fda.1188 | -3.073 | -3.652 |
| ZINC000053022902 | fda.1209 | -3.073 | -3.652 |
| ZINC000053022902 | fda.1210 | -3.073 | -3.652 |
| ZINC000033965961 | fda.2036 | -3.073 | -4.527 |
| ZINC000033965961 | fda.2037 | -3.073 | -4.527 |
| ZINC000033965961 | fda.2038 | -3.073 | -4.527 |
| ZINC000043200832 | fda.1848 | -3.07 | -3.301 |
| ZINC000043200832 | fda.1849 | -3.07 | -3.301 |
| ZINC000043200832 | fda.1850 | -3.07 | -3.301 |
| ZINC000003938482 | fda.1755 | -3.069 | -5.158 |
| ZINC000000538273 | fda.952 | -3.067 | -4.609 |
| ZINC000000538273 | fda.953 | -3.067 | -4.609 |
| ZINC000000537891 | fda.1373 | -3.067 | -4.609 |
| ZINC000000537891 | fda.1374 | -3.067 | -4.609 |
| ZINC000001530812 | fda.924 | -3.067 | -3.121 |
| ZINC000001530812 | fda.925 | -3.067 | -3.121 |
| ZINC000001530812 | fda.926 | -3.067 | -3.121 |
| ZINC000043202140 | fda.1828 | -3.064 | -3.806 |
| ZINC000043202140 | fda.1829 | -3.064 | -3.806 |
| ZINC000000001317 | fda.486 | -3.064 | -3.109 |
| ZINC000003079336 | fda.1459 | -3.06 | -3.06 |
| ZINC000000388081 | fda.1474 | -3.06 | -4.391 |
| ZINC000084843283 | fda.1809 | -3.058 | -4.78 |
| ZINC000004074875 | fda.1214 | -3.058 | -3.214 |
| ZINC000014164617 | fda.190 | -3.057 | -3.061 |
| ZINC000008220909 | fda.1347 | -3.054 | -3.086 |
| ZINC000035653007 | fda.1545 | -3.053 | -3.28 |
| ZINC000035653009 | fda.1546 | -3.053 | -3.28 |
| ZINC000000113355 | fda.1053 | -3.051 | -5.713 |
| ZINC000000601301 | fda.320 | -3.051 | -4.425 |
| ZINC000000897222 | fda.1658 | -3.051 | -4.425 |
| ZINC000022059926 | fda.645 | -3.051 | -4.654 |
| ZINC000022059930 | fda.1165 | -3.051 | -4.654 |
| ZINC000003830993 | fda.1461 | -3.051 | -4.039 |
| ZINC000169621200 | fda.1663 | -3.049 | -4.052 |
| ZINC000000621893 | fda.1869 | -3.045 | -3.062 |
| ZINC000000621893 | fda.1870 | -3.045 | -3.062 |
| ZINC000000074836 | fda.319 | -3.043 | -5.705 |
| ZINC000000968345 | fda.116 | -3.043 | -4.757 |
| ZINC000000968345 | fda.117 | -3.043 | -4.757 |
| ZINC000003651680 | fda.1689 | -3.043 | -4.757 |
| ZINC000003651680 | fda.1690 | -3.043 | -4.757 |
| ZINC000001999441 | fda.489 | -3.042 | -5.065 |
| ZINC000011679756 | fda.468 | -3.039 | -4.644 |
| ZINC000000049154 | fda.644 | -3.036 | -5.69 |
| ZINC000001543916 | fda.1856 | -3.035 | -3.249 |
| ZINC000043450326 | fda.1905 | -3.032 | -4.069 |
| ZINC000043450324 | fda.1919 | -3.032 | -4.069 |
| ZINC000019419017 | fda.770 | -3.031 | -4.87 |
| ZINC000068204830 | fda.215 | -3.03 | -3.832 |
| ZINC000003875342 | fda.1770 | -3.029 | -3.029 |
| ZINC000003873936 | fda.1732 | -3.026 | -3.989 |
| ZINC000003873936 | fda.1733 | -3.026 | -3.989 |
| ZINC000003812989 | fda.1712 | -3.022 | -4.981 |
| ZINC000003812989 | fda.1713 | -3.022 | -4.981 |
| ZINC000052957434 | fda.1207 | -3.02 | -4.679 |
| ZINC000052957434 | fda.1208 | -3.02 | -4.679 |
| ZINC000253632968 | fda.2093 | -3.016 | -5.687 |
| ZINC000003952881 | fda.649 | -3.014 | -5.737 |
| ZINC000004213946 | fda.951 | -3.008 | -5.031 |
| ZINC000005844792 | fda.1311 | -3.008 | -5.031 |
| ZINC000000001773 | fda.1842 | -3.006 | -4.741 |
| ZINC000000001773 | fda.1843 | -3.006 | -4.741 |
| ZINC000000001773 | fda.1844 | -3.006 | -4.741 |
| ZINC000003830276 | fda.2068 | -3.002 | -3.002 |
| ZINC000003986735 | fda.992 | -3.002 | -5.099 |
| ZINC000003972949 | fda.1984 | -3 | -3.014 |
| ZINC000035801098 | fda.1548 | -3 | -5.338 |
| ZINC000000039089 | fda.1157 | -2.999 | -5.344 |
| ZINC000003938686 | fda.1753 | -2.998 | -3.405 |
| ZINC000000006251 | fda.2007 | -2.998 | -4.775 |
| ZINC000000006251 | fda.2008 | -2.998 | -4.775 |
| ZINC000013298313 | fda.594 | -2.997 | -5.133 |
| ZINC000019364229 | fda.669 | -2.997 | -3.407 |
| ZINC000019364229 | fda.670 | -2.997 | -3.407 |
| ZINC000019364229 | fda.671 | -2.997 | -3.407 |
| ZINC000019364230 | fda.776 | -2.997 | -3.407 |
| ZINC000019364230 | fda.777 | -2.997 | -3.407 |
| ZINC000019364230 | fda.778 | -2.997 | -3.407 |
| ZINC000003782550 | fda.1388 | -2.996 | -3.695 |
| ZINC000000537791 | fda.1370 | -2.995 | -3.019 |
| ZINC000000537791 | fda.1371 | -2.995 | -3.019 |
| ZINC000000537791 | fda.1372 | -2.995 | -3.019 |
| ZINC000012414057 | fda.312 | -2.994 | -4.493 |
| ZINC000001530636 | fda.1523 | -2.992 | -4.171 |
| ZINC000003938704 | fda.157 | -2.992 | -3.019 |
| ZINC000094566092 | fda.1316 | -2.991 | -5.369 |
| ZINC000053683151 | fda.1189 | -2.99 | -3.169 |
| ZINC000053683151 | fda.1190 | -2.99 | -3.169 |
| ZINC000053683151 | fda.1191 | -2.99 | -3.169 |
| ZINC000012859773 | fda.2023 | -2.99 | -3.337 |
| ZINC000002519740 | fda.1125 | -2.988 | -4.397 |
| ZINC000002519740 | fda.1126 | -2.988 | -4.397 |
| ZINC000085537014 | fda.906 | -2.988 | -3.517 |
| ZINC000085537014 | fda.907 | -2.988 | -3.517 |
| ZINC000000000973 | fda.474 | -2.987 | -4.313 |
| ZINC000000000973 | fda.475 | -2.987 | -4.313 |
| ZINC000001530713 | fda.789 | -2.987 | -3.713 |
| ZINC000004097427 | fda.1216 | -2.987 | -3.853 |
| ZINC000028639340 | fda.177 | -2.985 | -5.074 |
| ZINC000100005670 | fda.1958 | -2.985 | -4.761 |
| ZINC000019364222 | fda.663 | -2.983 | -3.587 |
| ZINC000019364222 | fda.664 | -2.983 | -3.587 |
| ZINC000019364222 | fda.665 | -2.983 | -3.587 |
| ZINC000019364224 | fda.666 | -2.983 | -3.587 |
| ZINC000019364224 | fda.667 | -2.983 | -3.587 |
| ZINC000019364224 | fda.668 | -2.983 | -3.587 |
| ZINC000004213946 | fda.950 | -2.983 | -5.006 |
| ZINC000005844792 | fda.1310 | -2.983 | -5.006 |
| ZINC000068204830 | fda.215 | -2.979 | -4.15 |
| ZINC000001530814 | fda.798 | -2.979 | -4.42 |
| ZINC000001530814 | fda.799 | -2.979 | -4.42 |
| ZINC000001530814 | fda.800 | -2.979 | -4.42 |
| ZINC000001530812 | fda.924 | -2.979 | -4.42 |
| ZINC000001530812 | fda.925 | -2.979 | -4.42 |
| ZINC000001530812 | fda.926 | -2.979 | -4.42 |
| ZINC000019364229 | fda.669 | -2.978 | -4.838 |
| ZINC000019364229 | fda.670 | -2.978 | -4.838 |
| ZINC000019364229 | fda.671 | -2.978 | -4.838 |
| ZINC000019364230 | fda.776 | -2.978 | -4.838 |
| ZINC000019364230 | fda.777 | -2.978 | -4.838 |
| ZINC000019364230 | fda.778 | -2.978 | -4.838 |
| ZINC000006827693 | fda.1402 | -2.978 | -4.022 |
| ZINC000006827693 | fda.1403 | -2.978 | -4.022 |
| ZINC000000001773 | fda.1842 | -2.977 | -4.712 |
| ZINC000000001773 | fda.1843 | -2.977 | -4.712 |
| ZINC000000001773 | fda.1844 | -2.977 | -4.712 |
| ZINC000000968326 | fda.114 | -2.976 | -4.851 |
| ZINC000000968327 | fda.1747 | -2.976 | -4.851 |
| ZINC000001530579 | fda.1087 | -2.973 | -4.548 |
| ZINC000001530580 | fda.1089 | -2.973 | -4.548 |
| ZINC000019166988 | fda.1574 | -2.971 | -3.656 |
| ZINC000003831332 | fda.1472 | -2.971 | -2.971 |
| ZINC000100070954 | fda.76 | -2.969 | -2.992 |
| ZINC000019632917 | fda.710 | -2.969 | -4.457 |
| ZINC000004097308 | fda.899 | -2.965 | -2.965 |
| ZINC000001530636 | fda.1523 | -2.961 | -3.876 |
| ZINC000014879992 | fda.732 | -2.961 | -4.65 |
| ZINC000014879992 | fda.734 | -2.961 | -4.65 |
| ZINC000014879992 | fda.735 | -2.961 | -4.65 |
| ZINC000052509366 | fda.1212 | -2.961 | -3.34 |
| ZINC000001530816 | fda.676 | -2.96 | -3.022 |
| ZINC000001530817 | fda.801 | -2.96 | -3.022 |
| ZINC000012503187 | fda.567 | -2.958 | -3.872 |
| ZINC000003812841 | fda.1716 | -2.957 | -2.957 |
| ZINC000000895034 | fda.1380 | -2.952 | -2.952 |
| ZINC000169621220 | fda.275 | -2.951 | -4.697 |
| ZINC000001530752 | fda.152 | -2.95 | -4.183 |
| ZINC000001530752 | fda.153 | -2.95 | -4.183 |
| ZINC000001530751 | fda.202 | -2.95 | -4.183 |
| ZINC000001530751 | fda.203 | -2.95 | -4.183 |
| ZINC000001530707 | fda.698 | -2.95 | -2.953 |
| ZINC000001530706 | fda.786 | -2.95 | -2.953 |
| ZINC000003938704 | fda.156 | -2.949 | -2.976 |
| ZINC000000113446 | fda.419 | -2.947 | -4.668 |
| ZINC000001530940 | fda.1630 | -2.946 | -2.952 |
| ZINC000001530940 | fda.1631 | -2.946 | -2.952 |
| ZINC000034051848 | fda.1224 | -2.946 | -5.626 |
| ZINC000000002273 | fda.938 | -2.945 | -5.156 |
| ZINC000000000469 | fda.1514 | -2.945 | -5.156 |
| ZINC000022851765 | fda.171 | -2.943 | -3.829 |
| ZINC000022851765 | fda.172 | -2.943 | -3.829 |
| ZINC000022851765 | fda.173 | -2.943 | -3.829 |
| ZINC000022448097 | fda.2104 | -2.943 | -3.829 |
| ZINC000022448097 | fda.2105 | -2.943 | -3.829 |
| ZINC000022448097 | fda.2106 | -2.943 | -3.829 |
| ZINC000100070954 | fda.76 | -2.942 | -4.865 |
| ZINC000000537791 | fda.1370 | -2.942 | -4.865 |
| ZINC000000537791 | fda.1371 | -2.942 | -4.865 |
| ZINC000000537791 | fda.1372 | -2.942 | -4.865 |
| ZINC000000000856 | fda.1152 | -2.942 | -5.25 |
| ZINC000000897258 | fda.2011 | -2.942 | -5.25 |
| ZINC000004658290 | fda.15 | -2.942 | -4.324 |
| ZINC000150338698 | fda.1040 | -2.94 | -3.452 |
| ZINC000000004166 | fda.449 | -2.934 | -4.917 |
| ZINC000000896819 | fda.1081 | -2.934 | -4.917 |
| ZINC000000968345 | fda.116 | -2.933 | -4.647 |
| ZINC000000968345 | fda.117 | -2.933 | -4.647 |
| ZINC000003651680 | fda.1689 | -2.933 | -4.647 |
| ZINC000003651680 | fda.1690 | -2.933 | -4.647 |
| ZINC000000136138 | fda.150 | -2.932 | -4.613 |
| ZINC000000136138 | fda.151 | -2.932 | -4.613 |
| ZINC000011616925 | fda.144 | -2.932 | -4.527 |
| ZINC000001536109 | fda.221 | -2.932 | -4.527 |
| ZINC000000004413 | fda.522 | -2.926 | -2.975 |
| ZINC000000004413 | fda.523 | -2.926 | -2.975 |
| ZINC000008214635 | fda.98 | -2.925 | -2.925 |
| ZINC000018324776 | fda.700 | -2.925 | -3.604 |
| ZINC000018324776 | fda.701 | -2.925 | -3.604 |
| ZINC000018324776 | fda.702 | -2.925 | -3.604 |
| ZINC000000895034 | fda.1379 | -2.924 | -2.924 |
| ZINC000034051848 | fda.1223 | -2.923 | -5.603 |
| ZINC000013682481 | fda.752 | -2.917 | -3.873 |
| ZINC000018324776 | fda.700 | -2.917 | -3.781 |
| ZINC000018324776 | fda.701 | -2.917 | -3.781 |
| ZINC000018324776 | fda.702 | -2.917 | -3.781 |
| ZINC000052971887 | fda.1188 | -2.915 | -3.494 |
| ZINC000053022902 | fda.1209 | -2.915 | -3.494 |
| ZINC000053022902 | fda.1210 | -2.915 | -3.494 |
| ZINC000011679756 | fda.469 | -2.91 | -4.514 |
| ZINC000019632917 | fda.710 | -2.906 | -5.073 |
| ZINC000003873936 | fda.1732 | -2.905 | -4.062 |
| ZINC000003873936 | fda.1733 | -2.905 | -4.062 |
| ZINC000003938482 | fda.1755 | -2.902 | -4.991 |
| ZINC000018516586 | fda.428 | -2.902 | -4.085 |
| ZINC000072318121 | fda.2001 | -2.9 | -5.053 |
| ZINC000072318121 | fda.2002 | -2.9 | -5.053 |
| ZINC000003981610 | fda.1001 | -2.897 | -2.897 |
| ZINC000005819214 | fda.1299 | -2.897 | -2.897 |
| ZINC000001530814 | fda.798 | -2.893 | -2.947 |
| ZINC000001530814 | fda.799 | -2.893 | -2.947 |
| ZINC000001530814 | fda.800 | -2.893 | -2.947 |
| ZINC000000004413 | fda.522 | -2.892 | -2.941 |
| ZINC000000004413 | fda.523 | -2.892 | -2.941 |
| ZINC000000001773 | fda.1843 | -2.892 | -4.937 |
| ZINC000000001773 | fda.1844 | -2.892 | -4.937 |
| ZINC000021982937 | fda.2026 | -2.891 | -3.07 |
| ZINC000021982937 | fda.2027 | -2.891 | -3.07 |
| ZINC000021982937 | fda.2028 | -2.891 | -3.07 |
| ZINC000033965961 | fda.2036 | -2.891 | -3.07 |
| ZINC000033965961 | fda.2037 | -2.891 | -3.07 |
| ZINC000033965961 | fda.2038 | -2.891 | -3.07 |
| ZINC000029319828 | fda.1284 | -2.889 | -4.624 |
| ZINC000029319828 | fda.1285 | -2.889 | -4.624 |
| ZINC000095619105 | fda.1193 | -2.887 | -3.514 |
| ZINC000000008492 | fda.7 | -2.885 | -4.964 |
| ZINC000000000416 | fda.1488 | -2.882 | -4.82 |
| ZINC000000403011 | fda.1772 | -2.882 | -4.82 |
| ZINC000003812863 | fda.1648 | -2.882 | -3.878 |
| ZINC000003812983 | fda.1482 | -2.881 | -4.368 |
| ZINC000003812983 | fda.1483 | -2.881 | -4.368 |
| ZINC000036294079 | fda.678 | -2.879 | -3.961 |
| ZINC000036294079 | fda.679 | -2.879 | -3.961 |
| ZINC000035999642 | fda.745 | -2.879 | -3.961 |
| ZINC000035999642 | fda.746 | -2.879 | -3.961 |
| ZINC000001530694 | fda.464 | -2.878 | -2.878 |
| ZINC000003986735 | fda.990 | -2.877 | -4.973 |
| ZINC000003986735 | fda.991 | -2.877 | -4.973 |
| ZINC000003812989 | fda.1712 | -2.876 | -4.834 |
| ZINC000003812989 | fda.1713 | -2.876 | -4.834 |
| ZINC000013545634 | fda.703 | -2.876 | -4.597 |
| ZINC000013545634 | fda.704 | -2.876 | -4.597 |
| ZINC000013545636 | fda.387 | -2.876 | -4.597 |
| ZINC000013545636 | fda.388 | -2.876 | -4.597 |
| ZINC000003938704 | fda.157 | -2.874 | -4.714 |
| ZINC000034636383 | fda.2083 | -2.873 | -5.498 |
| ZINC000008214692 | fda.1111 | -2.871 | -4.45 |
| ZINC000000895316 | fda.233 | -2.87 | -2.87 |
| ZINC000000895318 | fda.246 | -2.87 | -2.87 |
| ZINC000003929022 | fda.508 | -2.868 | -2.868 |
| ZINC000000113442 | fda.138 | -2.864 | -5.241 |
| ZINC000003938704 | fda.156 | -2.859 | -4.699 |
| ZINC000003833846 | fda.95 | -2.856 | -2.87 |
| ZINC000014210876 | fda.1797 | -2.856 | -3.614 |
| ZINC000000000416 | fda.1487 | -2.854 | -4.792 |
| ZINC000000403011 | fda.1771 | -2.854 | -4.792 |
| ZINC000029571072 | fda.2033 | -2.853 | -4.758 |
| ZINC000049637509 | fda.2042 | -2.853 | -4.758 |
| ZINC000003807804 | fda.4 | -2.851 | -3.275 |
| ZINC000003807804 | fda.5 | -2.851 | -3.275 |
| ZINC000000000196 | fda.447 | -2.849 | -2.869 |
| ZINC000000000196 | fda.448 | -2.849 | -2.869 |
| ZINC000000155531 | fda.917 | -2.849 | -2.869 |
| ZINC000000155531 | fda.918 | -2.849 | -2.869 |
| ZINC000013831130 | fda.729 | -2.846 | -3.439 |
| ZINC000022002214 | fda.631 | -2.845 | -4.31 |
| ZINC000022002214 | fda.632 | -2.845 | -4.31 |
| ZINC000022002218 | fda.633 | -2.845 | -4.31 |
| ZINC000022002218 | fda.634 | -2.845 | -4.31 |
| ZINC000000049153 | fda.277 | -2.843 | -4.258 |
| ZINC000003830999 | fda.604 | -2.842 | -3.933 |
| ZINC000003830999 | fda.605 | -2.842 | -3.933 |
| ZINC000004097427 | fda.1216 | -2.839 | -2.996 |
| ZINC000053683151 | fda.1191 | -2.839 | -3.676 |
| ZINC000000895316 | fda.233 | -2.836 | -2.836 |
| ZINC000000895318 | fda.246 | -2.836 | -2.836 |
| ZINC000000896918 | fda.1169 | -2.835 | -5.445 |
| ZINC000022059268 | fda.1164 | -2.835 | -2.899 |
| ZINC000000000856 | fda.1152 | -2.832 | -5.139 |
| ZINC000000897258 | fda.2011 | -2.832 | -5.139 |
| ZINC000043195697 | fda.1925 | -2.828 | -3.073 |
| ZINC000043195697 | fda.1927 | -2.828 | -3.073 |
| ZINC000022116608 | fda.647 | -2.823 | -4.811 |
| ZINC000022116612 | fda.1167 | -2.823 | -4.811 |
| ZINC000000000973 | fda.474 | -2.818 | -4.184 |
| ZINC000000000973 | fda.475 | -2.818 | -4.184 |
| ZINC000003782550 | fda.1387 | -2.817 | -3.516 |
| ZINC000000001758 | fda.1672 | -2.815 | -4.529 |
| ZINC000095626706 | fda.1317 | -2.812 | -3.529 |
| ZINC000028639340 | fda.177 | -2.811 | -4.9 |
| ZINC000100004343 | fda.1321 | -2.809 | -2.833 |
| ZINC000100004345 | fda.1322 | -2.809 | -2.833 |
| ZINC000043450326 | fda.1905 | -2.806 | -2.918 |
| ZINC000043450326 | fda.1906 | -2.806 | -2.918 |
| ZINC000043450324 | fda.1919 | -2.806 | -2.918 |
| ZINC000043450324 | fda.1920 | -2.806 | -2.918 |
| ZINC000068204830 | fda.215 | -2.803 | -4.067 |
| ZINC000001530752 | fda.152 | -2.801 | -4.034 |
| ZINC000001530752 | fda.153 | -2.801 | -4.034 |
| ZINC000001530751 | fda.202 | -2.801 | -4.034 |
| ZINC000001530751 | fda.203 | -2.801 | -4.034 |
| ZINC000002016257 | fda.1969 | -2.801 | -2.801 |
| ZINC000002016258 | fda.1970 | -2.801 | -2.801 |
| ZINC000000896523 | fda.1453 | -2.8 | -2.801 |
| ZINC000000000257 | fda.1775 | -2.8 | -2.801 |
| ZINC000043200832 | fda.1850 | -2.8 | -3.47 |
| ZINC000021982937 | fda.2026 | -2.799 | -4.253 |
| ZINC000021982937 | fda.2027 | -2.799 | -4.253 |
| ZINC000021982937 | fda.2028 | -2.799 | -4.253 |
| ZINC000003831531 | fda.1010 | -2.797 | -2.797 |
| ZINC000003831531 | fda.1011 | -2.797 | -2.797 |
| ZINC000001530707 | fda.698 | -2.794 | -2.797 |
| ZINC000001530706 | fda.786 | -2.794 | -2.797 |
| ZINC000019364222 | fda.663 | -2.789 | -4.514 |
| ZINC000019364222 | fda.664 | -2.789 | -4.514 |
| ZINC000019364222 | fda.665 | -2.789 | -4.514 |
| ZINC000019364224 | fda.666 | -2.789 | -4.514 |
| ZINC000019364224 | fda.667 | -2.789 | -4.514 |
| ZINC000019364224 | fda.668 | -2.789 | -4.514 |
| ZINC000095616599 | fda.1288 | -2.788 | -5.017 |
| ZINC000095616600 | fda.1325 | -2.788 | -5.017 |
| ZINC000019203912 | fda.1583 | -2.783 | -3.119 |
| ZINC000019203912 | fda.1584 | -2.783 | -3.119 |
| ZINC000019203912 | fda.1585 | -2.783 | -3.119 |
| ZINC000006661227 | fda.1196 | -2.78 | -4.501 |
| ZINC000002005550 | fda.154 | -2.78 | -4.31 |
| ZINC000002005550 | fda.155 | -2.78 | -4.31 |
| ZINC000000643046 | fda.1606 | -2.778 | -2.779 |
| ZINC000242437514 | fda.360 | -2.777 | -2.962 |
| ZINC000003813042 | fda.1814 | -2.776 | -4.091 |
| ZINC000004215736 | fda.421 | -2.773 | -4.994 |
| ZINC000004215736 | fda.422 | -2.773 | -4.994 |
| ZINC000001530569 | fda.1141 | -2.772 | -2.773 |
| ZINC000001530570 | fda.1142 | -2.772 | -2.773 |
| ZINC000000643138 | fda.1028 | -2.771 | -5.246 |
| ZINC000000643143 | fda.253 | -2.771 | -5.246 |
| ZINC000100018854 | fda.499 | -2.77 | -2.771 |
| ZINC000100018854 | fda.501 | -2.77 | -2.771 |
| ZINC000003794711 | fda.407 | -2.769 | -4.815 |
| ZINC000003794711 | fda.408 | -2.769 | -4.815 |
| ZINC000003794711 | fda.409 | -2.769 | -4.815 |
| ZINC000068247389 | fda.1999 | -2.767 | -3.288 |
| ZINC000068247389 | fda.2000 | -2.767 | -3.288 |
| ZINC000021982937 | fda.2026 | -2.766 | -4.188 |
| ZINC000021982937 | fda.2027 | -2.766 | -4.188 |
| ZINC000021982937 | fda.2028 | -2.766 | -4.188 |
| ZINC000004097476 | fda.834 | -2.763 | -2.763 |
| ZINC000003991624 | fda.69 | -2.761 | -2.766 |
| ZINC000000000347 | fda.1937 | -2.758 | -2.759 |
| ZINC000000000347 | fda.1939 | -2.758 | -2.759 |
| ZINC000019594557 | fda.755 | -2.756 | -4.918 |
| ZINC000019594557 | fda.756 | -2.756 | -4.918 |
| ZINC000019361042 | fda.762 | -2.756 | -4.918 |
| ZINC000019361042 | fda.763 | -2.756 | -4.918 |
| ZINC000014768621 | fda.760 | -2.755 | -3.541 |
| ZINC000000643138 | fda.1027 | -2.755 | -5.229 |
| ZINC000000643143 | fda.252 | -2.755 | -5.229 |
| ZINC000150338698 | fda.1039 | -2.754 | -3.266 |
| ZINC000000000456 | fda.1220 | -2.753 | -3.884 |
| ZINC000000000456 | fda.1221 | -2.753 | -3.884 |
| ZINC000000000456 | fda.1222 | -2.753 | -3.884 |
| ZINC000000154964 | fda.1834 | -2.753 | -3.884 |
| ZINC000000154964 | fda.1835 | -2.753 | -3.884 |
| ZINC000000154964 | fda.1836 | -2.753 | -3.884 |
| ZINC000001550499 | fda.1859 | -2.749 | -5.117 |
| ZINC000034636383 | fda.2083 | -2.743 | -5.367 |
| ZINC000095616599 | fda.1288 | -2.742 | -4.971 |
| ZINC000095616600 | fda.1325 | -2.742 | -4.971 |
| ZINC000003782550 | fda.1388 | -2.74 | -2.958 |
| ZINC000001550477 | fda.328 | -2.737 | -5.051 |
| ZINC000001530886 | fda.404 | -2.733 | -3.443 |
| ZINC000001530886 | fda.405 | -2.733 | -3.443 |
| ZINC000017146904 | fda.717 | -2.733 | -4.147 |
| ZINC000003816514 | fda.1698 | -2.732 | -2.814 |
| ZINC000068204830 | fda.215 | -2.729 | -3.598 |
| ZINC000043195697 | fda.1924 | -2.728 | -3.409 |
| ZINC000043195697 | fda.1926 | -2.728 | -3.409 |
| ZINC000021982937 | fda.2024 | -2.727 | -4.148 |
| ZINC000021982937 | fda.2025 | -2.727 | -4.148 |
| ZINC000003938686 | fda.1752 | -2.726 | -4.638 |
| ZINC000005844788 | fda.188 | -2.725 | -4.749 |
| ZINC000003782550 | fda.1387 | -2.722 | -2.941 |
| ZINC000000968326 | fda.115 | -2.722 | -4.597 |
| ZINC000000968327 | fda.1748 | -2.722 | -4.597 |
| ZINC000005179119 | fda.1031 | -2.718 | -4.811 |
| ZINC000150338698 | fda.1040 | -2.718 | -3.23 |
| ZINC000000057533 | fda.985 | -2.716 | -5.274 |
| ZINC000000057532 | fda.1018 | -2.716 | -5.274 |
| ZINC000001530654 | fda.1622 | -2.715 | -4.419 |
| ZINC000001530654 | fda.1623 | -2.715 | -4.419 |
| ZINC000084758235 | fda.828 | -2.715 | -4.575 |
| ZINC000003943279 | fda.1420 | -2.714 | -3.45 |
| ZINC000000000711 | fda.1940 | -2.71 | -5.088 |
| ZINC000001843047 | fda.1964 | -2.71 | -5.088 |
| ZINC000019156872 | fda.1580 | -2.709 | -4.741 |
| ZINC000019156872 | fda.1581 | -2.709 | -4.741 |
| ZINC000019156872 | fda.1582 | -2.709 | -4.741 |
| ZINC000022116608 | fda.646 | -2.708 | -4.696 |
| ZINC000022116612 | fda.1166 | -2.708 | -4.696 |
| ZINC000004655029 | fda.876 | -2.707 | -2.71 |
| ZINC000004655029 | fda.877 | -2.707 | -2.71 |
| ZINC000001530950 | fda.893 | -2.702 | -2.702 |
| ZINC000001530951 | fda.1633 | -2.702 | -2.702 |
| ZINC000000001317 | fda.486 | -2.698 | -4.24 |
| ZINC000001530636 | fda.1523 | -2.697 | -4.096 |
| ZINC000003781943 | fda.1693 | -2.696 | -3.166 |
| ZINC000019364242 | fda.768 | -2.69 | -2.705 |
| ZINC000019364242 | fda.769 | -2.69 | -2.705 |
| ZINC000005844788 | fda.187 | -2.69 | -4.713 |
| ZINC000019594557 | fda.755 | -2.689 | -4.851 |
| ZINC000019594557 | fda.756 | -2.689 | -4.851 |
| ZINC000019361042 | fda.762 | -2.689 | -4.851 |
| ZINC000019361042 | fda.763 | -2.689 | -4.851 |
| ZINC000000537822 | fda.437 | -2.688 | -4.94 |
| ZINC000000537822 | fda.438 | -2.688 | -4.94 |
| ZINC000000896663 | fda.487 | -2.687 | -4.496 |
| ZINC000001639567 | fda.1646 | -2.687 | -4.496 |
| ZINC000084441937 | fda.1909 | -2.685 | -4.998 |
| ZINC000022016976 | fda.1158 | -2.683 | -2.714 |
| ZINC000022016976 | fda.1159 | -2.683 | -2.714 |
| ZINC000022016976 | fda.1160 | -2.683 | -2.714 |
| ZINC000022016981 | fda.1161 | -2.683 | -2.714 |
| ZINC000022016981 | fda.1162 | -2.683 | -2.714 |
| ZINC000022016981 | fda.1163 | -2.683 | -2.714 |
| ZINC000000000856 | fda.1153 | -2.682 | -4.99 |
| ZINC000000897258 | fda.2012 | -2.682 | -4.99 |
| ZINC000003812988 | fda.2055 | -2.679 | -2.69 |
| ZINC000003812988 | fda.2056 | -2.679 | -2.69 |
| ZINC000029416466 | fda.158 | -2.678 | -4.938 |
| ZINC000029416466 | fda.159 | -2.678 | -4.938 |
| ZINC000029416466 | fda.160 | -2.678 | -4.938 |
| ZINC000021982937 | fda.2026 | -2.678 | -4.099 |
| ZINC000021982937 | fda.2027 | -2.678 | -4.099 |
| ZINC000021982937 | fda.2028 | -2.678 | -4.099 |
| ZINC000003818726 | fda.184 | -2.676 | -5.207 |
| ZINC000000000507 | fda.1563 | -2.675 | -3.736 |
| ZINC000000403079 | fda.1677 | -2.675 | -3.736 |
| ZINC000019796168 | fda.389 | -2.674 | -3.493 |
| ZINC000019796168 | fda.390 | -2.674 | -3.493 |
| ZINC000019796168 | fda.391 | -2.674 | -3.493 |
| ZINC000100378061 | fda.530 | -2.674 | -5.009 |
| ZINC000100378061 | fda.532 | -2.674 | -5.009 |
| ZINC000003941829 | fda.571 | -2.672 | -2.746 |
| ZINC000003941829 | fda.572 | -2.672 | -2.746 |
| ZINC000001530652 | fda.459 | -2.672 | -4.375 |
| ZINC000001530652 | fda.460 | -2.672 | -4.375 |
| ZINC000017146904 | fda.718 | -2.671 | -4.085 |
| ZINC000001533877 | fda.818 | -2.67 | -3.84 |
| ZINC000001533877 | fda.819 | -2.67 | -3.84 |
| ZINC000001533877 | fda.820 | -2.67 | -3.84 |
| ZINC000001533877 | fda.822 | -2.67 | -3.84 |
| ZINC000001533877 | fda.823 | -2.67 | -3.84 |
| ZINC000006661227 | fda.1197 | -2.668 | -4.389 |
| ZINC000006661227 | fda.1198 | -2.668 | -4.389 |
| ZINC000000621853 | fda.599 | -2.665 | -2.665 |
| ZINC000000607790 | fda.908 | -2.665 | -2.665 |
| ZINC000000968310 | fda.331 | -2.663 | -5.138 |
| ZINC000000968310 | fda.332 | -2.663 | -5.138 |
| ZINC000000968310 | fda.333 | -2.663 | -5.138 |
| ZINC000000000509 | fda.1549 | -2.663 | -5.138 |
| ZINC000000000509 | fda.1550 | -2.663 | -5.138 |
| ZINC000000000509 | fda.1551 | -2.663 | -5.138 |
| ZINC000001532525 | fda.616 | -2.662 | -2.662 |
| ZINC000000001408 | fda.1560 | -2.66 | -5.041 |
| ZINC000001481956 | fda.795 | -2.657 | -5.079 |
| ZINC000001481956 | fda.796 | -2.657 | -5.079 |
| ZINC000004214700 | fda.995 | -2.657 | -5.079 |
| ZINC000004214700 | fda.996 | -2.657 | -5.079 |
| ZINC000019632834 | fda.739 | -2.655 | -3.718 |
| ZINC000019632834 | fda.740 | -2.655 | -3.718 |
| ZINC000019632834 | fda.741 | -2.655 | -3.718 |
| ZINC000001530621 | fda.812 | -2.654 | -4.066 |
| ZINC000004099200 | fda.836 | -2.648 | -4.734 |
| ZINC000004676424 | fda.881 | -2.648 | -4.734 |
| ZINC000052955754 | fda.129 | -2.646 | -3.511 |
| ZINC000052955754 | fda.130 | -2.646 | -3.511 |
| ZINC000052955754 | fda.131 | -2.646 | -3.511 |
| ZINC000018203737 | fda.367 | -2.64 | -3.948 |
| ZINC000018203737 | fda.368 | -2.64 | -3.948 |
| ZINC000001532517 | fda.262 | -2.638 | -2.638 |
| ZINC000100022637 | fda.1292 | -2.638 | -2.775 |
| ZINC000100016058 | fda.2078 | -2.638 | -2.775 |
| ZINC000035328014 | fda.1555 | -2.636 | -5.335 |
| ZINC000000402954 | fda.965 | -2.635 | -4.384 |
| ZINC000000402954 | fda.966 | -2.635 | -4.384 |
| ZINC000000402954 | fda.967 | -2.635 | -4.384 |
| ZINC000004228258 | fda.1061 | -2.633 | -4.66 |
| ZINC000004228258 | fda.1062 | -2.633 | -4.66 |
| ZINC000004228258 | fda.1063 | -2.633 | -4.66 |
| ZINC000004228257 | fda.257 | -2.633 | -4.66 |
| ZINC000004228257 | fda.258 | -2.633 | -4.66 |
| ZINC000004228257 | fda.259 | -2.633 | -4.66 |
| ZINC000019364222 | fda.663 | -2.632 | -2.95 |
| ZINC000019364222 | fda.664 | -2.632 | -2.95 |
| ZINC000019364222 | fda.665 | -2.632 | -2.95 |
| ZINC000019364224 | fda.666 | -2.632 | -2.95 |
| ZINC000019364224 | fda.667 | -2.632 | -2.95 |
| ZINC000019364224 | fda.668 | -2.632 | -2.95 |
| ZINC000006845963 | fda.1989 | -2.631 | -2.631 |
| ZINC000000391812 | fda.1054 | -2.63 | -4.538 |
| ZINC000000391812 | fda.1055 | -2.63 | -4.538 |
| ZINC000000391812 | fda.1056 | -2.63 | -4.538 |
| ZINC000000001798 | fda.1135 | -2.63 | -4.538 |
| ZINC000000001798 | fda.1136 | -2.63 | -4.538 |
| ZINC000000001798 | fda.1137 | -2.63 | -4.538 |
| ZINC000003938686 | fda.1753 | -2.629 | -4.541 |
| ZINC000100022637 | fda.1293 | -2.628 | -2.766 |
| ZINC000100016058 | fda.2079 | -2.628 | -2.766 |
| ZINC000004074875 | fda.1214 | -2.627 | -3.493 |
| ZINC000008220909 | fda.1347 | -2.626 | -4.373 |
| ZINC000000403618 | fda.516 | -2.622 | -2.622 |
| ZINC000003798750 | fda.228 | -2.622 | -2.622 |
| ZINC000008552123 | fda.1567 | -2.621 | -4.116 |
| ZINC000008552123 | fda.1568 | -2.621 | -4.116 |
| ZINC000008552123 | fda.1569 | -2.621 | -4.116 |
| ZINC000003875393 | fda.1718 | -2.621 | -4.116 |
| ZINC000003875393 | fda.1719 | -2.621 | -4.116 |
| ZINC000003875393 | fda.1720 | -2.621 | -4.116 |
| ZINC000003976838 | fda.1761 | -2.62 | -4.435 |
| ZINC000003976838 | fda.1762 | -2.62 | -4.435 |
| ZINC000004474603 | fda.1262 | -2.62 | -2.623 |
| ZINC000003874950 | fda.229 | -2.619 | -4.15 |
| ZINC000003874950 | fda.230 | -2.619 | -4.15 |
| ZINC000095551509 | fda.503 | -2.619 | -2.625 |
| ZINC000033965961 | fda.2036 | -2.619 | -4.222 |
| ZINC000033965961 | fda.2037 | -2.619 | -4.222 |
| ZINC000033965961 | fda.2038 | -2.619 | -4.222 |
| ZINC000003916214 | fda.1980 | -2.617 | -4.432 |
| ZINC000003916214 | fda.1981 | -2.617 | -4.432 |
| ZINC000028108825 | fda.89 | -2.616 | -4.758 |
| ZINC000028108825 | fda.90 | -2.616 | -4.758 |
| ZINC000028108825 | fda.91 | -2.616 | -4.758 |
| ZINC000043195697 | fda.1926 | -2.615 | -2.861 |
| ZINC000002005305 | fda.21 | -2.615 | -4.847 |
| ZINC000003920266 | fda.1888 | -2.612 | -4.452 |
| ZINC000003920266 | fda.1887 | -2.611 | -4.451 |
| ZINC000068204830 | fda.215 | -2.609 | -3.913 |
| ZINC000001530689 | fda.1133 | -2.608 | -2.629 |
| ZINC000001530689 | fda.1134 | -2.608 | -2.629 |
| ZINC000001532522 | fda.236 | -2.607 | -2.607 |
| ZINC000008214629 | fda.1993 | -2.606 | -2.606 |
| ZINC000003929508 | fda.1436 | -2.603 | -2.685 |
| ZINC000000538312 | fda.840 | -2.602 | -5.024 |
| ZINC000000538312 | fda.841 | -2.602 | -5.024 |
| ZINC000003831490 | fda.374 | -2.599 | -5.016 |
| ZINC000003831490 | fda.375 | -2.599 | -5.016 |
| ZINC000004099200 | fda.835 | -2.599 | -4.617 |
| ZINC000004676424 | fda.880 | -2.599 | -4.617 |
| ZINC000001530703 | fda.694 | -2.592 | -2.592 |
| ZINC000001530636 | fda.1524 | -2.588 | -3.771 |
| ZINC000021982937 | fda.2026 | -2.586 | -4.041 |
| ZINC000021982937 | fda.2027 | -2.586 | -4.041 |
| ZINC000021982937 | fda.2028 | -2.586 | -4.041 |
| ZINC000001482197 | fda.456 | -2.586 | -4.395 |
| ZINC000000896666 | fda.81 | -2.586 | -4.395 |
| ZINC000001529323 | fda.16 | -2.585 | -5.047 |
| ZINC000003831332 | fda.1472 | -2.585 | -2.585 |
| ZINC000001530621 | fda.813 | -2.582 | -3.994 |
| ZINC000000643138 | fda.1027 | -2.58 | -5.279 |
| ZINC000000643138 | fda.1028 | -2.58 | -5.279 |
| ZINC000000643143 | fda.252 | -2.58 | -5.279 |
| ZINC000000643143 | fda.253 | -2.58 | -5.279 |
| ZINC000068247389 | fda.1999 | -2.579 | -4.056 |
| ZINC000068247389 | fda.2000 | -2.579 | -4.056 |
| ZINC000001530636 | fda.1523 | -2.579 | -3.762 |
| ZINC000100378061 | fda.530 | -2.579 | -4.922 |
| ZINC000100378061 | fda.531 | -2.579 | -4.922 |
| ZINC000100378061 | fda.532 | -2.579 | -4.922 |
| ZINC000002019953 | fda.902 | -2.579 | -2.628 |
| ZINC000002019954 | fda.936 | -2.579 | -2.628 |
| ZINC000000538273 | fda.952 | -2.577 | -5.13 |
| ZINC000000538273 | fda.953 | -2.577 | -5.13 |
| ZINC000000537891 | fda.1373 | -2.577 | -5.13 |
| ZINC000000537891 | fda.1374 | -2.577 | -5.13 |
| ZINC000008214692 | fda.1111 | -2.574 | -4.183 |
| ZINC000100378061 | fda.531 | -2.57 | -4.905 |
| ZINC000014210876 | fda.1798 | -2.569 | -3.874 |
| ZINC000019362735 | fda.657 | -2.569 | -4.6 |
| ZINC000019362735 | fda.658 | -2.569 | -4.6 |
| ZINC000019362735 | fda.659 | -2.569 | -4.6 |
| ZINC000019362737 | fda.660 | -2.569 | -4.6 |
| ZINC000019362737 | fda.661 | -2.569 | -4.6 |
| ZINC000019362737 | fda.662 | -2.569 | -4.6 |
| ZINC000100001964 | fda.1286 | -2.568 | -2.572 |
| ZINC000100001965 | fda.1330 | -2.568 | -2.572 |
| ZINC000001853550 | fda.1688 | -2.568 | -4.866 |
| ZINC000000607986 | fda.63 | -2.567 | -4.59 |
| ZINC000001530621 | fda.812 | -2.564 | -3.976 |
| ZINC000000537928 | fda.851 | -2.563 | -2.593 |
| ZINC000000002281 | fda.1029 | -2.559 | -4.774 |
| ZINC000000000746 | fda.149 | -2.559 | -4.774 |
| ZINC000006661227 | fda.1196 | -2.558 | -4.278 |
| ZINC000003830986 | fda.1012 | -2.557 | -4.591 |
| ZINC000000599734 | fda.1496 | -2.557 | -4.591 |
| ZINC000033965961 | fda.2036 | -2.557 | -3.978 |
| ZINC000033965961 | fda.2037 | -2.557 | -3.978 |
| ZINC000033965961 | fda.2038 | -2.557 | -3.978 |
| ZINC000000896569 | fda.1353 | -2.556 | -4.807 |
| ZINC000003953037 | fda.1759 | -2.554 | -2.6 |
| ZINC000084441937 | fda.1909 | -2.552 | -4.865 |
| ZINC000001533877 | fda.821 | -2.551 | -3.721 |
| ZINC000003976838 | fda.1761 | -2.551 | -4.366 |
| ZINC000003976838 | fda.1762 | -2.551 | -4.366 |
| ZINC000095619101 | fda.1305 | -2.549 | -4.403 |
| ZINC000095619101 | fda.1306 | -2.549 | -4.403 |
| ZINC000095619100 | fda.1327 | -2.549 | -4.403 |
| ZINC000095619100 | fda.1328 | -2.549 | -4.403 |
| ZINC000018115268 | fda.722 | -2.542 | -3.084 |
| ZINC000018115268 | fda.723 | -2.542 | -3.084 |
| ZINC000019632633 | fda.224 | -2.542 | -3.64 |
| ZINC000019632633 | fda.225 | -2.542 | -3.64 |
| ZINC000019632633 | fda.226 | -2.542 | -3.64 |
| ZINC000035801098 | fda.1547 | -2.54 | -4.878 |
| ZINC000001532522 | fda.236 | -2.539 | -2.539 |
| ZINC000019419017 | fda.770 | -2.535 | -4.711 |
| ZINC000043450326 | fda.1906 | -2.533 | -3.571 |
| ZINC000043450324 | fda.1920 | -2.533 | -3.571 |
| ZINC000001530654 | fda.1622 | -2.531 | -4.235 |
| ZINC000001530654 | fda.1623 | -2.531 | -4.235 |
| ZINC000017285869 | fda.2080 | -2.531 | -2.531 |
| ZINC000017285872 | fda.2081 | -2.531 | -2.531 |
| ZINC000002016257 | fda.1969 | -2.525 | -2.525 |
| ZINC000002016258 | fda.1970 | -2.525 | -2.525 |
| ZINC000022056030 | fda.145 | -2.517 | -3.615 |
| ZINC000022056030 | fda.146 | -2.517 | -3.615 |
| ZINC000022056030 | fda.147 | -2.517 | -3.615 |
| ZINC000003875483 | fda.548 | -2.516 | -4.628 |
| ZINC000003875483 | fda.549 | -2.516 | -4.628 |
| ZINC000003875483 | fda.550 | -2.516 | -4.628 |
| ZINC000084441937 | fda.1909 | -2.515 | -4.811 |
| ZINC000021297660 | fda.121 | -2.514 | -4.271 |
| ZINC000021297660 | fda.122 | -2.514 | -4.271 |
| ZINC000003875334 | fda.1060 | -2.512 | -2.512 |
| ZINC000000391812 | fda.1054 | -2.511 | -4.418 |
| ZINC000000391812 | fda.1055 | -2.511 | -4.418 |
| ZINC000000391812 | fda.1056 | -2.511 | -4.418 |
| ZINC000000001798 | fda.1135 | -2.511 | -4.418 |
| ZINC000000001798 | fda.1136 | -2.511 | -4.418 |
| ZINC000000001798 | fda.1137 | -2.511 | -4.418 |
| ZINC000001481956 | fda.795 | -2.504 | -4.926 |
| ZINC000001481956 | fda.796 | -2.504 | -4.926 |
| ZINC000004214700 | fda.995 | -2.504 | -4.926 |
| ZINC000004214700 | fda.996 | -2.504 | -4.926 |
| ZINC000022443609 | fda.1847 | -2.503 | -3.241 |
| ZINC000000004413 | fda.522 | -2.501 | -4.001 |
| ZINC000000004413 | fda.523 | -2.501 | -4.001 |
| ZINC000000538658 | fda.864 | -2.5 | -2.5 |
| ZINC000001530579 | fda.1086 | -2.498 | -4.073 |
| ZINC000001530580 | fda.1088 | -2.498 | -4.073 |
| ZINC000000537931 | fda.1375 | -2.498 | -4.368 |
| ZINC000011677857 | fda.479 | -2.497 | -4.794 |
| ZINC000033965961 | fda.2034 | -2.492 | -4.095 |
| ZINC000033965961 | fda.2035 | -2.492 | -4.095 |
| ZINC000003798763 | fda.439 | -2.491 | -2.494 |
| ZINC000033965961 | fda.2034 | -2.49 | -4.094 |
| ZINC000033965961 | fda.2035 | -2.49 | -4.094 |
| ZINC000003977981 | fda.1013 | -2.489 | -2.489 |
| ZINC000000020259 | fda.706 | -2.489 | -5.151 |
| ZINC000001550499 | fda.1859 | -2.488 | -4.856 |
| ZINC000245204949 | fda.335 | -2.486 | -4.933 |
| ZINC000005764759 | fda.1331 | -2.486 | -4.933 |
| ZINC000000004351 | fda.1723 | -2.485 | -5.061 |
| ZINC000000004351 | fda.1724 | -2.485 | -5.061 |
| ZINC000019632917 | fda.710 | -2.485 | -3.973 |
| ZINC000100378061 | fda.530 | -2.484 | -3.107 |
| ZINC000100378061 | fda.532 | -2.484 | -3.107 |
| ZINC000003938652 | fda.1982 | -2.482 | -2.482 |
| ZINC000053683151 | fda.1189 | -2.481 | -5.071 |
| ZINC000053683151 | fda.1190 | -2.481 | -5.071 |
| ZINC000053683151 | fda.1191 | -2.481 | -5.071 |
| ZINC000014879972 | fda.731 | -2.481 | -4.029 |
| ZINC000001534965 | fda.601 | -2.478 | -4.934 |
| ZINC000000968305 | fda.1432 | -2.478 | -4.09 |
| ZINC000000968305 | fda.1433 | -2.478 | -4.09 |
| ZINC000021982937 | fda.2024 | -2.477 | -4.081 |
| ZINC000021982937 | fda.2025 | -2.477 | -4.081 |
| ZINC000003812989 | fda.1712 | -2.473 | -5.036 |
| ZINC000003812989 | fda.1713 | -2.473 | -5.036 |
| ZINC000095616603 | fda.1376 | -2.472 | -4.894 |
| ZINC000019228902 | fda.1575 | -2.471 | -4.441 |
| ZINC000019228902 | fda.1576 | -2.471 | -4.441 |
| ZINC000019228902 | fda.1577 | -2.471 | -4.441 |
| ZINC000001319780 | fda.1790 | -2.469 | -2.473 |
| ZINC000001530636 | fda.1524 | -2.462 | -3.87 |
| ZINC000000968328 | fda.402 | -2.461 | -3.838 |
| ZINC000000968330 | fda.1172 | -2.461 | -3.838 |
| ZINC000008214625 | fda.1566 | -2.461 | -2.461 |
| ZINC000000896695 | fda.1530 | -2.46 | -2.7 |
| ZINC000018279854 | fda.747 | -2.458 | -5.179 |
| ZINC000001530814 | fda.798 | -2.458 | -3.898 |
| ZINC000001530814 | fda.799 | -2.458 | -3.898 |
| ZINC000001530814 | fda.800 | -2.458 | -3.898 |
| ZINC000001530812 | fda.924 | -2.458 | -3.898 |
| ZINC000001530812 | fda.925 | -2.458 | -3.898 |
| ZINC000001530812 | fda.926 | -2.458 | -3.898 |
| ZINC000017285869 | fda.2080 | -2.455 | -2.455 |
| ZINC000017285872 | fda.2081 | -2.455 | -2.455 |
| ZINC000006827695 | fda.1410 | -2.451 | -4.789 |
| ZINC000006827695 | fda.1411 | -2.451 | -4.789 |
| ZINC000068204830 | fda.215 | -2.45 | -3.601 |
| ZINC000000000416 | fda.1487 | -2.444 | -4.381 |
| ZINC000000403011 | fda.1771 | -2.444 | -4.381 |
| ZINC000084441937 | fda.1909 | -2.44 | -4.736 |
| ZINC000094566093 | fda.1304 | -2.44 | -5.038 |
| ZINC000095564694 | fda.2095 | -2.437 | -2.523 |
| ZINC000095564694 | fda.2096 | -2.437 | -2.523 |
| ZINC000095564694 | fda.2097 | -2.437 | -2.523 |
| ZINC000038197764 | fda.1020 | -2.436 | -3.582 |
| ZINC000003607120 | fda.9 | -2.436 | -3.582 |
| ZINC000000402954 | fda.965 | -2.434 | -4.251 |
| ZINC000000402954 | fda.966 | -2.434 | -4.251 |
| ZINC000000402954 | fda.967 | -2.434 | -4.251 |
| ZINC000004658553 | fda.1469 | -2.431 | -2.431 |
| ZINC000095616601 | fda.17 | -2.428 | -4.729 |
| ZINC000035653009 | fda.1546 | -2.428 | -3.108 |
| ZINC000000000196 | fda.447 | -2.426 | -2.446 |
| ZINC000000000196 | fda.448 | -2.426 | -2.446 |
| ZINC000000155531 | fda.917 | -2.426 | -2.446 |
| ZINC000000155531 | fda.918 | -2.426 | -2.446 |
| ZINC000019364222 | fda.663 | -2.425 | -3.029 |
| ZINC000019364222 | fda.664 | -2.425 | -3.029 |
| ZINC000019364222 | fda.665 | -2.425 | -3.029 |
| ZINC000019364224 | fda.666 | -2.425 | -3.029 |
| ZINC000019364224 | fda.667 | -2.425 | -3.029 |
| ZINC000019364224 | fda.668 | -2.425 | -3.029 |
| ZINC000008214635 | fda.98 | -2.425 | -2.425 |
| ZINC000016929327 | fda.1113 | -2.418 | -4.92 |
| ZINC000017146904 | fda.718 | -2.411 | -4.298 |
| ZINC000100014909 | fda.1280 | -2.41 | -3.233 |
| ZINC000100014909 | fda.1281 | -2.41 | -3.233 |
| ZINC000005844788 | fda.187 | -2.41 | -4.433 |
| ZINC000001530816 | fda.676 | -2.41 | -3.78 |
| ZINC000001530817 | fda.801 | -2.41 | -3.78 |
| ZINC000001530703 | fda.695 | -2.409 | -2.427 |
| ZINC000001530703 | fda.696 | -2.409 | -2.427 |
| ZINC000001530703 | fda.697 | -2.409 | -2.427 |
| ZINC000003812944 | fda.161 | -2.406 | -2.406 |
| ZINC000018516586 | fda.428 | -2.405 | -4.974 |
| ZINC000100004343 | fda.1321 | -2.405 | -4.306 |
| ZINC000100004345 | fda.1322 | -2.405 | -4.306 |
| ZINC000012503151 | fda.566 | -2.402 | -5.067 |
| ZINC000253917094 | fda.2094 | -2.402 | -5.067 |
| ZINC000095452610 | fda.1323 | -2.401 | -2.811 |
| ZINC000035328014 | fda.1555 | -2.4 | -5.078 |
| ZINC000100378061 | fda.531 | -2.398 | -5.036 |
| ZINC000029571072 | fda.2032 | -2.397 | -4.169 |
| ZINC000049637509 | fda.2041 | -2.397 | -4.169 |
| ZINC000029571072 | fda.2033 | -2.396 | -4.168 |
| ZINC000049637509 | fda.2042 | -2.396 | -4.168 |
| ZINC000000001773 | fda.1842 | -2.385 | -4.43 |
| ZINC000004074875 | fda.1213 | -2.383 | -3.249 |
| ZINC000001530886 | fda.405 | -2.382 | -4.254 |
| ZINC000000000711 | fda.1940 | -2.377 | -4.755 |
| ZINC000001843047 | fda.1964 | -2.377 | -4.755 |
| ZINC000034220093 | fda.1366 | -2.377 | -4.599 |
| ZINC000001853205 | fda.1750 | -2.374 | -3.103 |
| ZINC000000896523 | fda.1453 | -2.374 | -2.375 |
| ZINC000000000257 | fda.1775 | -2.374 | -2.375 |
| ZINC000001633887 | fda.20 | -2.368 | -2.368 |
| ZINC000001633889 | fda.37 | -2.368 | -2.368 |
| ZINC000001530636 | fda.1524 | -2.359 | -3.538 |
| ZINC000002522669 | fda.910 | -2.358 | -2.375 |
| ZINC000002522669 | fda.911 | -2.358 | -2.375 |
| ZINC000000001795 | fda.1423 | -2.356 | -4.804 |
| ZINC000000389747 | fda.32 | -2.354 | -4.467 |
| ZINC000000389747 | fda.33 | -2.354 | -4.467 |
| ZINC000000389747 | fda.34 | -2.354 | -4.467 |
| ZINC000033965961 | fda.2036 | -2.35 | -3.954 |
| ZINC000033965961 | fda.2037 | -2.35 | -3.954 |
| ZINC000033965961 | fda.2038 | -2.35 | -3.954 |
| ZINC000001530697 | fda.691 | -2.35 | -2.351 |
| ZINC000001530697 | fda.693 | -2.35 | -2.351 |
| ZINC000001530695 | fda.1626 | -2.35 | -2.351 |
| ZINC000016929327 | fda.1113 | -2.348 | -4.85 |
| ZINC000017146904 | fda.717 | -2.347 | -4.234 |
| ZINC000003872994 | fda.217 | -2.342 | -4.817 |
| ZINC000000643153 | fda.1819 | -2.342 | -4.817 |
| ZINC000011677857 | fda.479 | -2.335 | -2.372 |
| ZINC000000001681 | fda.1668 | -2.334 | -4.527 |
| ZINC000000001681 | fda.1669 | -2.334 | -4.527 |
| ZINC000003943279 | fda.1420 | -2.332 | -3.869 |
| ZINC000072316335 | fda.1929 | -2.33 | -3.732 |
| ZINC000000113355 | fda.1053 | -2.328 | -4.99 |
| ZINC000000538273 | fda.952 | -2.324 | -4.877 |
| ZINC000000538273 | fda.953 | -2.324 | -4.877 |
| ZINC000000537891 | fda.1373 | -2.324 | -4.877 |
| ZINC000000537891 | fda.1374 | -2.324 | -4.877 |
| ZINC000003818808 | fda.491 | -2.321 | -4.662 |
| ZINC000004693575 | fda.323 | -2.314 | -4.083 |
| ZINC000004693574 | fda.1217 | -2.314 | -4.083 |
| ZINC000019203912 | fda.1583 | -2.313 | -4.283 |
| ZINC000019203912 | fda.1584 | -2.313 | -4.283 |
| ZINC000019203912 | fda.1585 | -2.313 | -4.283 |
| ZINC000000607986 | fda.62 | -2.313 | -4.336 |
| ZINC000035328014 | fda.1555 | -2.312 | -4.99 |
| ZINC000001529425 | fda.928 | -2.309 | -2.309 |
| ZINC000001853550 | fda.1688 | -2.309 | -4.607 |
| ZINC000014210876 | fda.1797 | -2.305 | -3.737 |
| ZINC000014210876 | fda.1798 | -2.305 | -3.737 |
| ZINC000000389747 | fda.32 | -2.3 | -4.413 |
| ZINC000000389747 | fda.33 | -2.3 | -4.413 |
| ZINC000000389747 | fda.34 | -2.3 | -4.413 |
| ZINC000094566092 | fda.1316 | -2.298 | -4.895 |
| ZINC000003813042 | fda.1814 | -2.297 | -4.13 |
| ZINC000033965961 | fda.2034 | -2.29 | -3.712 |
| ZINC000033965961 | fda.2035 | -2.29 | -3.712 |
| ZINC000005844788 | fda.188 | -2.285 | -4.309 |
| ZINC000003953037 | fda.1759 | -2.283 | -4.018 |
| ZINC000000968310 | fda.331 | -2.283 | -4.758 |
| ZINC000000968310 | fda.332 | -2.283 | -4.758 |
| ZINC000000968310 | fda.333 | -2.283 | -4.758 |
| ZINC000000000509 | fda.1549 | -2.283 | -4.758 |
| ZINC000000000509 | fda.1550 | -2.283 | -4.758 |
| ZINC000000000509 | fda.1551 | -2.283 | -4.758 |
| ZINC000019364229 | fda.669 | -2.281 | -4.14 |
| ZINC000019364229 | fda.670 | -2.281 | -4.14 |
| ZINC000019364229 | fda.671 | -2.281 | -4.14 |
| ZINC000019364230 | fda.776 | -2.281 | -4.14 |
| ZINC000019364230 | fda.777 | -2.281 | -4.14 |
| ZINC000019364230 | fda.778 | -2.281 | -4.14 |
| ZINC000000896663 | fda.487 | -2.279 | -4.088 |
| ZINC000001639567 | fda.1646 | -2.279 | -4.088 |
| ZINC000000538275 | fda.308 | -2.279 | -4.742 |
| ZINC000000538275 | fda.309 | -2.279 | -4.742 |
| ZINC000003982483 | fda.1891 | -2.277 | -4.69 |
| ZINC000003982483 | fda.1892 | -2.277 | -4.69 |
| ZINC000003982483 | fda.1893 | -2.277 | -4.69 |
| ZINC000000601281 | fda.867 | -2.274 | -2.342 |
| ZINC000000601281 | fda.868 | -2.274 | -2.342 |
| ZINC000051951647 | fda.1090 | -2.273 | -4.813 |
| ZINC000043195697 | fda.1923 | -2.273 | -2.954 |
| ZINC000043195697 | fda.1925 | -2.273 | -2.954 |
| ZINC000043195697 | fda.1927 | -2.273 | -2.954 |
| ZINC000094566093 | fda.1303 | -2.268 | -3.306 |
| ZINC000094566092 | fda.1315 | -2.268 | -3.306 |
| ZINC000019796018 | fda.742 | -2.268 | -4.611 |
| ZINC000019796018 | fda.743 | -2.268 | -4.611 |
| ZINC000019796018 | fda.744 | -2.268 | -4.611 |
| ZINC000002005550 | fda.154 | -2.267 | -3.798 |
| ZINC000002005550 | fda.155 | -2.267 | -3.798 |
| ZINC000003872994 | fda.218 | -2.267 | -4.741 |
| ZINC000000643153 | fda.1820 | -2.267 | -4.741 |
| ZINC000033965961 | fda.2036 | -2.265 | -3.719 |
| ZINC000033965961 | fda.2037 | -2.265 | -3.719 |
| ZINC000033965961 | fda.2038 | -2.265 | -3.719 |
| ZINC000003953037 | fda.1758 | -2.263 | -3.998 |
| ZINC000003800008 | fda.193 | -2.263 | -2.931 |
| ZINC000003800008 | fda.194 | -2.263 | -2.931 |
| ZINC000003800008 | fda.195 | -2.263 | -2.931 |
| ZINC000030690433 | fda.1333 | -2.262 | -3.52 |
| ZINC000030690433 | fda.1334 | -2.262 | -3.52 |
| ZINC000030690433 | fda.1335 | -2.262 | -3.52 |
| ZINC000003831405 | fda.28 | -2.257 | -4.534 |
| ZINC000003831405 | fda.29 | -2.257 | -4.534 |
| ZINC000001532525 | fda.616 | -2.255 | -2.255 |
| ZINC000003938746 | fda.1673 | -2.254 | -3.928 |
| ZINC000003938746 | fda.1674 | -2.254 | -3.928 |
| ZINC000003938746 | fda.1675 | -2.254 | -3.928 |
| ZINC000004099008 | fda.2015 | -2.246 | -2.246 |
| ZINC000034781704 | fda.1192 | -2.243 | -4.191 |
| ZINC000021982937 | fda.2026 | -2.242 | -3.845 |
| ZINC000021982937 | fda.2027 | -2.242 | -3.845 |
| ZINC000021982937 | fda.2028 | -2.242 | -3.845 |
| ZINC000019419017 | fda.771 | -2.239 | -4.079 |
| ZINC000019419017 | fda.772 | -2.239 | -4.079 |
| ZINC000004658553 | fda.1469 | -2.238 | -2.238 |
| ZINC000008035268 | fda.496 | -2.233 | -2.233 |
| ZINC000084758479 | fda.1807 | -2.23 | -3.171 |
| ZINC000084758479 | fda.1808 | -2.23 | -3.171 |
| ZINC000053683151 | fda.1189 | -2.226 | -4.816 |
| ZINC000053683151 | fda.1190 | -2.226 | -4.816 |
| ZINC000053683151 | fda.1191 | -2.226 | -4.816 |
| ZINC000002005305 | fda.22 | -2.226 | -4.458 |
| ZINC000002005305 | fda.23 | -2.226 | -4.458 |
| ZINC000019419017 | fda.771 | -2.225 | -4.401 |
| ZINC000019419017 | fda.772 | -2.225 | -4.401 |
| ZINC000116473771 | fda.539 | -2.224 | -4.869 |
| ZINC000100017856 | fda.1959 | -2.224 | -4.869 |
| ZINC000100017856 | fda.1960 | -2.224 | -4.869 |
| ZINC000051951647 | fda.1090 | -2.222 | -4.279 |
| ZINC000003812983 | fda.1482 | -2.217 | -4.788 |
| ZINC000003812983 | fda.1483 | -2.217 | -4.788 |
| ZINC000022447798 | fda.1821 | -2.216 | -3.727 |
| ZINC000022447798 | fda.1822 | -2.216 | -3.727 |
| ZINC000013129998 | fda.1661 | -2.215 | -4.384 |
| ZINC000018279854 | fda.747 | -2.211 | -4.932 |
| ZINC000019362735 | fda.657 | -2.21 | -4.242 |
| ZINC000019362735 | fda.658 | -2.21 | -4.242 |
| ZINC000019362735 | fda.659 | -2.21 | -4.242 |
| ZINC000019362737 | fda.660 | -2.21 | -4.242 |
| ZINC000019362737 | fda.661 | -2.21 | -4.242 |
| ZINC000019362737 | fda.662 | -2.21 | -4.242 |
| ZINC000001530636 | fda.1524 | -2.21 | -3.609 |
| ZINC000000000596 | fda.1441 | -2.206 | -4.546 |
| ZINC000000000596 | fda.1442 | -2.206 | -4.546 |
| ZINC000006827695 | fda.1409 | -2.203 | -4.54 |
| ZINC000000114124 | fda.589 | -2.202 | -4.421 |
| ZINC000000114127 | fda.590 | -2.202 | -4.421 |
| ZINC000003812989 | fda.1712 | -2.189 | -4.752 |
| ZINC000003812989 | fda.1713 | -2.189 | -4.752 |
| ZINC000001542002 | fda.1874 | -2.186 | -3.761 |
| ZINC000001530811 | fda.896 | -2.183 | -2.193 |
| ZINC000001530811 | fda.897 | -2.183 | -2.193 |
| ZINC000001493878 | fda.1526 | -2.181 | -4.495 |
| ZINC000049933061 | fda.1907 | -2.178 | -3.462 |
| ZINC000019632633 | fda.224 | -2.177 | -3.276 |
| ZINC000019632633 | fda.225 | -2.177 | -3.276 |
| ZINC000019632633 | fda.226 | -2.177 | -3.276 |
| ZINC000022010649 | fda.485 | -2.175 | -4.714 |
| ZINC000000901159 | fda.618 | -2.173 | -2.173 |
| ZINC000169621200 | fda.1663 | -2.17 | -3.173 |
| ZINC000018324776 | fda.701 | -2.169 | -3.927 |
| ZINC000022448696 | fda.1823 | -2.169 | -2.811 |
| ZINC000022448696 | fda.1824 | -2.169 | -2.811 |
| ZINC000022448696 | fda.1827 | -2.169 | -2.811 |
| ZINC000019418959 | fda.779 | -2.168 | -4.511 |
| ZINC000019418959 | fda.780 | -2.168 | -4.511 |
| ZINC000019418959 | fda.781 | -2.168 | -4.511 |
| ZINC000000537805 | fda.581 | -2.167 | -4.149 |
| ZINC000000537805 | fda.582 | -2.167 | -4.149 |
| ZINC000000537805 | fda.583 | -2.167 | -4.149 |
| ZINC000148723177 | fda.338 | -2.165 | -4.413 |
| ZINC000148723177 | fda.339 | -2.165 | -4.413 |
| ZINC000008220909 | fda.1347 | -2.165 | -2.197 |
| ZINC000022443609 | fda.1847 | -2.165 | -2.872 |
| ZINC000095619101 | fda.1305 | -2.162 | -4.017 |
| ZINC000095619101 | fda.1306 | -2.162 | -4.017 |
| ZINC000095619100 | fda.1327 | -2.162 | -4.017 |
| ZINC000095619100 | fda.1328 | -2.162 | -4.017 |
| ZINC000004658290 | fda.14 | -2.161 | -4.594 |
| ZINC000008214692 | fda.1111 | -2.156 | -2.428 |
| ZINC000014210876 | fda.1797 | -2.156 | -3.449 |
| ZINC000043195697 | fda.1923 | -2.155 | -2.4 |
| ZINC000043195697 | fda.1925 | -2.155 | -2.4 |
| ZINC000043195697 | fda.1927 | -2.155 | -2.4 |
| ZINC000000000596 | fda.1441 | -2.148 | -4.487 |
| ZINC000000000596 | fda.1442 | -2.148 | -4.487 |
| ZINC000003861768 | fda.135 | -2.147 | -4.662 |
| ZINC000043195697 | fda.1926 | -2.127 | -2.808 |
| ZINC000004095696 | fda.1659 | -2.126 | -2.133 |
| ZINC000100014909 | fda.1280 | -2.124 | -2.979 |
| ZINC000100014909 | fda.1281 | -2.124 | -2.979 |
| ZINC000003938704 | fda.157 | -2.121 | -3.961 |
| ZINC000003872994 | fda.217 | -2.12 | -4.595 |
| ZINC000000643153 | fda.1819 | -2.12 | -4.595 |
| ZINC000001530886 | fda.404 | -2.118 | -4.275 |
| ZINC000003872277 | fda.1738 | -2.117 | -4.687 |
| ZINC000001530816 | fda.677 | -2.117 | -3.488 |
| ZINC000001530817 | fda.802 | -2.117 | -3.488 |
| ZINC000094566093 | fda.1304 | -2.117 | -4.495 |
| ZINC000000538509 | fda.162 | -2.103 | -4.116 |
| ZINC000000538509 | fda.163 | -2.103 | -4.116 |
| ZINC000012404516 | fda.199 | -2.095 | -4.108 |
| ZINC000012404516 | fda.200 | -2.095 | -4.108 |
| ZINC000003830321 | fda.1879 | -2.093 | -2.105 |
| ZINC000000001931 | fda.1942 | -2.09 | -4.103 |
| ZINC000000001931 | fda.1943 | -2.09 | -4.103 |
| ZINC000000000565 | fda.686 | -2.089 | -4.326 |
| ZINC000000000565 | fda.687 | -2.089 | -4.326 |
| ZINC000000155269 | fda.1832 | -2.089 | -4.326 |
| ZINC000000155269 | fda.1833 | -2.089 | -4.326 |
| ZINC000000000416 | fda.1488 | -2.079 | -4.017 |
| ZINC000000403011 | fda.1772 | -2.079 | -4.017 |
| ZINC000036701290 | fda.519 | -2.065 | -4.569 |
| ZINC000036701290 | fda.520 | -2.065 | -4.569 |
| ZINC000018324776 | fda.700 | -2.065 | -2.836 |
| ZINC000018324776 | fda.702 | -2.065 | -2.836 |
| ZINC000008034121 | fda.1358 | -2.064 | -4.682 |
| ZINC000022010649 | fda.485 | -2.059 | -4.116 |
| ZINC000014210876 | fda.1797 | -2.057 | -3.489 |
| ZINC000019144226 | fda.1588 | -2.057 | -3.733 |
| ZINC000019144231 | fda.1863 | -2.057 | -3.733 |
| ZINC000003941829 | fda.571 | -2.053 | -2.127 |
| ZINC000003941829 | fda.572 | -2.053 | -2.127 |
| ZINC000003941829 | fda.573 | -2.053 | -2.127 |
| ZINC000000403618 | fda.516 | -2.048 | -2.048 |
| ZINC000003798750 | fda.228 | -2.048 | -2.048 |
| ZINC000019144226 | fda.1588 | -2.047 | -3.723 |
| ZINC000019144231 | fda.1863 | -2.047 | -3.723 |
| ZINC000003833846 | fda.94 | -2.045 | -4.277 |
| ZINC000003833846 | fda.95 | -2.045 | -4.277 |
| ZINC000003833846 | fda.96 | -2.045 | -4.277 |
| ZINC000002005305 | fda.22 | -2.033 | -4.265 |
| ZINC000002005305 | fda.23 | -2.033 | -4.265 |
| ZINC000018324776 | fda.700 | -2.024 | -3.689 |
| ZINC000018324776 | fda.701 | -2.024 | -3.689 |
| ZINC000018324776 | fda.702 | -2.024 | -3.689 |
| ZINC000003818726 | fda.184 | -2.021 | -4.274 |
| ZINC000003818726 | fda.185 | -2.021 | -4.274 |
| ZINC000003953037 | fda.1759 | -2.02 | -3.754 |
| ZINC000019594594 | fda.782 | -2.015 | -2.415 |
| ZINC000019594594 | fda.783 | -2.015 | -2.415 |
| ZINC000002570895 | fda.300 | -2.013 | -2.014 |
| ZINC000001530652 | fda.459 | -2.01 | -3.713 |
| ZINC000001530652 | fda.460 | -2.01 | -3.713 |
| ZINC000016052277 | fda.1897 | -2.005 | -4.281 |
| ZINC000000000565 | fda.686 | -2.003 | -4.24 |
| ZINC000000000565 | fda.687 | -2.003 | -4.24 |
| ZINC000000155269 | fda.1832 | -2.003 | -4.24 |
| ZINC000000155269 | fda.1833 | -2.003 | -4.24 |
| ZINC000004215736 | fda.421 | -2.003 | -4.224 |
| ZINC000004215736 | fda.422 | -2.003 | -4.224 |
| ZINC000001530762 | fda.1635 | -2.001 | -2.02 |
| ZINC000001530762 | fda.1636 | -2.001 | -2.02 |
| ZINC000000596731 | fda.296 | -1.999 | -4.042 |
| ZINC000000596731 | fda.297 | -1.999 | -4.042 |
| ZINC000000896695 | fda.1529 | -1.998 | -2.65 |
| ZINC000026985532 | fda.355 | -1.996 | -4.256 |
| ZINC000026985532 | fda.356 | -1.996 | -4.256 |
| ZINC000026985532 | fda.357 | -1.996 | -4.256 |
| ZINC000000114124 | fda.589 | -1.99 | -4.209 |
| ZINC000000114127 | fda.590 | -1.99 | -4.209 |
| ZINC000014768621 | fda.760 | -1.982 | -3.333 |
| ZINC000008214692 | fda.1111 | -1.981 | -3.508 |
| ZINC000094566093 | fda.1303 | -1.98 | -4.197 |
| ZINC000094566092 | fda.1315 | -1.98 | -4.197 |
| ZINC000003953037 | fda.1759 | -1.98 | -4.297 |
| ZINC000000896695 | fda.1529 | -1.978 | -2.218 |
| ZINC000003953037 | fda.1758 | -1.977 | -3.712 |
| ZINC000004658290 | fda.15 | -1.972 | -4.313 |
| ZINC000000895042 | fda.231 | -1.964 | -4.183 |
| ZINC000011680943 | fda.470 | -1.963 | -3.096 |
| ZINC000003818726 | fda.185 | -1.959 | -4.49 |
| ZINC000001530886 | fda.405 | -1.955 | -4.112 |
| ZINC000000895048 | fda.232 | -1.948 | -1.948 |
| ZINC000003812988 | fda.2055 | -1.947 | -4.325 |
| ZINC000003812988 | fda.2056 | -1.947 | -4.325 |
| ZINC000003953037 | fda.1758 | -1.944 | -1.99 |
| ZINC000006382803 | fda.1023 | -1.943 | -4.544 |
| ZINC000001530820 | fda.463 | -1.942 | -1.942 |
| ZINC000002005305 | fda.21 | -1.941 | -4.173 |
| ZINC000000896717 | fda.1168 | -1.938 | -4.599 |
| ZINC000019364222 | fda.663 | -1.928 | -3.653 |
| ZINC000019364222 | fda.664 | -1.928 | -3.653 |
| ZINC000019364222 | fda.665 | -1.928 | -3.653 |
| ZINC000019364224 | fda.666 | -1.928 | -3.653 |
| ZINC000019364224 | fda.667 | -1.928 | -3.653 |
| ZINC000019364224 | fda.668 | -1.928 | -3.653 |
| ZINC000022443609 | fda.1847 | -1.928 | -3.193 |
| ZINC000030690433 | fda.1333 | -1.928 | -2.026 |
| ZINC000030690433 | fda.1334 | -1.928 | -2.026 |
| ZINC000030690433 | fda.1335 | -1.928 | -2.026 |
| ZINC000043195697 | fda.1925 | -1.924 | -2.605 |
| ZINC000043195697 | fda.1927 | -1.924 | -2.605 |
| ZINC000004658290 | fda.15 | -1.924 | -4.475 |
| ZINC000011677857 | fda.479 | -1.918 | -4.215 |
| ZINC000022851765 | fda.171 | -1.913 | -3.636 |
| ZINC000022448097 | fda.2104 | -1.913 | -3.636 |
| ZINC000000968264 | fda.1644 | -1.896 | -4.371 |
| ZINC000000968264 | fda.1645 | -1.896 | -4.371 |
| ZINC000000643138 | fda.1028 | -1.889 | -4.364 |
| ZINC000000643143 | fda.253 | -1.889 | -4.364 |
| ZINC000001530816 | fda.676 | -1.882 | -3.253 |
| ZINC000001530817 | fda.801 | -1.882 | -3.253 |
| ZINC000011616925 | fda.144 | -1.882 | -3.477 |
| ZINC000001536109 | fda.221 | -1.882 | -3.477 |
| ZINC000000020244 | fda.1049 | -1.865 | -4.102 |
| ZINC000000020244 | fda.1050 | -1.865 | -4.102 |
| ZINC000014768621 | fda.760 | -1.864 | -3.215 |
| ZINC000011680943 | fda.471 | -1.852 | -1.947 |
| ZINC000084758235 | fda.828 | -1.845 | -3.704 |
| ZINC000014879992 | fda.733 | -1.842 | -4.189 |
| ZINC000003943279 | fda.1420 | -1.842 | -3.985 |
| ZINC000004658552 | fda.623 | -1.84 | -1.84 |
| ZINC000094566093 | fda.1304 | -1.839 | -4.217 |
| ZINC000072318121 | fda.2001 | -1.836 | -4.488 |
| ZINC000072318121 | fda.2002 | -1.836 | -4.488 |
| ZINC000043195697 | fda.1923 | -1.836 | -2.517 |
| ZINC000001530718 | fda.882 | -1.835 | -1.837 |
| ZINC000001530717 | fda.1116 | -1.835 | -1.837 |
| ZINC000029571072 | fda.2033 | -1.835 | -2.338 |
| ZINC000049637509 | fda.2042 | -1.835 | -2.338 |
| ZINC000096903163 | fda.1916 | -1.834 | -1.834 |
| ZINC000001530303 | fda.261 | -1.826 | -1.826 |
| ZINC000004175630 | fda.948 | -1.819 | -4.464 |
| ZINC000004175630 | fda.949 | -1.819 | -4.464 |
| ZINC000043200832 | fda.1850 | -1.816 | -2.486 |
| ZINC000100378061 | fda.530 | -1.811 | -3.463 |
| ZINC000100378061 | fda.531 | -1.811 | -3.463 |
| ZINC000100378061 | fda.532 | -1.811 | -3.463 |
| ZINC000001530816 | fda.677 | -1.807 | -3.177 |
| ZINC000001530817 | fda.802 | -1.807 | -3.177 |
| ZINC000003875483 | fda.548 | -1.799 | -3.911 |
| ZINC000003875483 | fda.549 | -1.799 | -3.911 |
| ZINC000003875483 | fda.550 | -1.799 | -3.911 |
| ZINC000008214692 | fda.1111 | -1.795 | -3.373 |
| ZINC000026011099 | fda.39 | -1.79 | -2.828 |
| ZINC000003938746 | fda.1673 | -1.78 | -3.454 |
| ZINC000003938746 | fda.1674 | -1.78 | -3.454 |
| ZINC000003938746 | fda.1675 | -1.78 | -3.454 |
| ZINC000021982937 | fda.2026 | -1.779 | -3.382 |
| ZINC000021982937 | fda.2027 | -1.779 | -3.382 |
| ZINC000021982937 | fda.2028 | -1.779 | -3.382 |
| ZINC000014210876 | fda.1797 | -1.774 | -3.067 |
| ZINC000003955219 | fda.650 | -1.773 | -1.773 |
| ZINC000022447798 | fda.1821 | -1.772 | -3.646 |
| ZINC000022447798 | fda.1822 | -1.772 | -3.646 |
| ZINC000001530703 | fda.694 | -1.771 | -1.771 |
| ZINC000086040406 | fda.1240 | -1.764 | -4.027 |
| ZINC000000621853 | fda.599 | -1.763 | -1.763 |
| ZINC000000607790 | fda.908 | -1.763 | -1.763 |
| ZINC000003872994 | fda.217 | -1.762 | -4.46 |
| ZINC000003872994 | fda.218 | -1.762 | -4.46 |
| ZINC000000643153 | fda.1819 | -1.762 | -4.46 |
| ZINC000000643153 | fda.1820 | -1.762 | -4.46 |
| ZINC000001530635 | fda.681 | -1.761 | -1.761 |
| ZINC000094566093 | fda.1304 | -1.759 | -4.357 |
| ZINC000008214692 | fda.1111 | -1.757 | -3.111 |
| ZINC000022016976 | fda.1158 | -1.756 | -3.864 |
| ZINC000022016976 | fda.1160 | -1.756 | -3.864 |
| ZINC000022016981 | fda.1161 | -1.756 | -3.864 |
| ZINC000022016981 | fda.1163 | -1.756 | -3.864 |
| ZINC000001530613 | fda.915 | -1.755 | -3.873 |
| ZINC000001530613 | fda.916 | -1.755 | -3.873 |
| ZINC000021982937 | fda.2024 | -1.754 | -3.357 |
| ZINC000021982937 | fda.2025 | -1.754 | -3.357 |
| ZINC000016159083 | fda.169 | -1.75 | -1.752 |
| ZINC000016159083 | fda.170 | -1.75 | -1.752 |
| ZINC000001530938 | fda.1954 | -1.746 | -3.732 |
| ZINC000001530938 | fda.1955 | -1.746 | -3.732 |
| ZINC000003830891 | fda.239 | -1.74 | -4.443 |
| ZINC000000006251 | fda.2007 | -1.728 | -3.505 |
| ZINC000000006251 | fda.2008 | -1.728 | -3.505 |
| ZINC000094566092 | fda.1316 | -1.72 | -4.318 |
| ZINC000022448983 | fda.399 | -1.713 | -3.762 |
| ZINC000022448983 | fda.400 | -1.713 | -3.762 |
| ZINC000022448983 | fda.401 | -1.713 | -3.762 |
| ZINC000008214692 | fda.1111 | -1.71 | -3.319 |
| ZINC000000538386 | fda.578 | -1.707 | -1.746 |
| ZINC000000538386 | fda.579 | -1.707 | -1.746 |
| ZINC000095616937 | fda.1934 | -1.704 | -4.123 |
| ZINC000008220878 | fda.1586 | -1.691 | -4.054 |
| ZINC000008220878 | fda.1587 | -1.691 | -4.054 |
| ZINC000000005151 | fda.1418 | -1.679 | -4.397 |
| ZINC000000005151 | fda.1419 | -1.679 | -4.397 |
| ZINC000000004028 | fda.1615 | -1.679 | -4.397 |
| ZINC000000004028 | fda.1616 | -1.679 | -4.397 |
| ZINC000003798537 | fda.2062 | -1.675 | -4.214 |
| ZINC000006827693 | fda.1402 | -1.674 | -1.786 |
| ZINC000006827693 | fda.1403 | -1.674 | -1.786 |
| ZINC000084589076 | fda.1911 | -1.672 | -3.67 |
| ZINC000100006264 | fda.1917 | -1.672 | -3.67 |
| ZINC000242437512 | fda.507 | -1.666 | -3.485 |
| ZINC000014881137 | fda.72 | -1.664 | -1.664 |
| ZINC000068247389 | fda.1999 | -1.663 | -3.139 |
| ZINC000068247389 | fda.2000 | -1.663 | -3.139 |
| ZINC000004074875 | fda.1213 | -1.663 | -2.529 |
| ZINC000029571072 | fda.2032 | -1.658 | -3.431 |
| ZINC000049637509 | fda.2041 | -1.658 | -3.431 |
| ZINC000001633887 | fda.20 | -1.658 | -1.658 |
| ZINC000001633889 | fda.37 | -1.658 | -1.658 |
| ZINC000008214402 | fda.1598 | -1.657 | -4.019 |
| ZINC000008214402 | fda.1599 | -1.657 | -4.019 |
| ZINC000000155905 | fda.505 | -1.648 | -3.969 |
| ZINC000003831551 | fda.68 | -1.648 | -3.969 |
| ZINC000000537791 | fda.1370 | -1.648 | -3.571 |
| ZINC000000537791 | fda.1371 | -1.648 | -3.571 |
| ZINC000000537791 | fda.1372 | -1.648 | -3.571 |
| ZINC000000896695 | fda.1530 | -1.647 | -2.299 |
| ZINC000022056030 | fda.145 | -1.647 | -2.745 |
| ZINC000022056030 | fda.146 | -1.647 | -2.745 |
| ZINC000022056030 | fda.147 | -1.647 | -2.745 |
| ZINC000003812983 | fda.1482 | -1.644 | -4.215 |
| ZINC000003812983 | fda.1483 | -1.644 | -4.215 |
| ZINC000002019953 | fda.902 | -1.644 | -1.693 |
| ZINC000002019954 | fda.936 | -1.644 | -1.693 |
| ZINC000000004413 | fda.522 | -1.628 | -3.129 |
| ZINC000000004413 | fda.523 | -1.628 | -3.129 |
| ZINC000008220909 | fda.1347 | -1.626 | -3.372 |
| ZINC000001853205 | fda.1750 | -1.622 | -2.35 |
| ZINC000253632968 | fda.2093 | -1.621 | -4.292 |
| ZINC000242437511 | fda.334 | -1.617 | -1.803 |
| ZINC000000608382 | fda.51 | -1.615 | -4.044 |
| ZINC000000608382 | fda.52 | -1.615 | -4.044 |
| ZINC000242437511 | fda.334 | -1.61 | -3.317 |
| ZINC000022443609 | fda.1847 | -1.592 | -3.725 |
| ZINC000000643138 | fda.1027 | -1.592 | -4.066 |
| ZINC000000643143 | fda.252 | -1.592 | -4.066 |
| ZINC000000155905 | fda.505 | -1.59 | -3.912 |
| ZINC000003831551 | fda.68 | -1.59 | -3.912 |
| ZINC000011680943 | fda.470 | -1.59 | -1.685 |
| ZINC000100378061 | fda.530 | -1.589 | -4.226 |
| ZINC000100378061 | fda.532 | -1.589 | -4.226 |
| ZINC000029571072 | fda.2033 | -1.575 | -3.48 |
| ZINC000049637509 | fda.2042 | -1.575 | -3.48 |
| ZINC000000621893 | fda.1869 | -1.566 | -3.662 |
| ZINC000000621893 | fda.1870 | -1.566 | -3.662 |
| ZINC000003938746 | fda.1673 | -1.564 | -1.6 |
| ZINC000003938746 | fda.1674 | -1.564 | -1.6 |
| ZINC000018324776 | fda.700 | -1.561 | -3.412 |
| ZINC000018324776 | fda.701 | -1.561 | -3.412 |
| ZINC000018324776 | fda.702 | -1.561 | -3.412 |
| ZINC000035328014 | fda.1555 | -1.556 | -4.255 |
| ZINC000003953037 | fda.1758 | -1.553 | -3.87 |
| ZINC000043195697 | fda.1923 | -1.55 | -3.982 |
| ZINC000043195697 | fda.1924 | -1.55 | -3.982 |
| ZINC000100378061 | fda.531 | -1.543 | -4.181 |
| ZINC000004097427 | fda.1215 | -1.529 | -2.395 |
| ZINC000003830635 | fda.38 | -1.517 | -1.517 |
| ZINC000052716421 | fda.1185 | -1.502 | -3.876 |
| ZINC000052716421 | fda.1186 | -1.502 | -3.876 |
| ZINC000034781704 | fda.1192 | -1.499 | -3.447 |
| ZINC000011726211 | fda.591 | -1.496 | -4.015 |
| ZINC000011726211 | fda.592 | -1.496 | -4.015 |
| ZINC000242437514 | fda.360 | -1.488 | -1.673 |
| ZINC000006920384 | fda.1110 | -1.482 | -1.482 |
| ZINC000018115268 | fda.722 | -1.481 | -2.255 |
| ZINC000018115268 | fda.723 | -1.481 | -2.255 |
| ZINC000000537752 | fda.845 | -1.473 | -3.771 |
| ZINC000000537752 | fda.846 | -1.473 | -3.771 |
| ZINC000100022637 | fda.1293 | -1.472 | -1.61 |
| ZINC000100016058 | fda.2079 | -1.472 | -1.61 |
| ZINC000068204830 | fda.215 | -1.47 | -2.621 |
| ZINC000001530689 | fda.1133 | -1.467 | -3.48 |
| ZINC000001530689 | fda.1134 | -1.467 | -3.48 |
| ZINC000022447798 | fda.1821 | -1.463 | -4.161 |
| ZINC000022447798 | fda.1822 | -1.463 | -4.161 |
| ZINC000094566093 | fda.1303 | -1.456 | -3.673 |
| ZINC000094566092 | fda.1315 | -1.456 | -3.673 |
| ZINC000022447798 | fda.1821 | -1.455 | -3.678 |
| ZINC000022447798 | fda.1822 | -1.455 | -3.678 |
| ZINC000019594594 | fda.782 | -1.452 | -1.873 |
| ZINC000019594594 | fda.783 | -1.452 | -1.873 |
| ZINC000014879972 | fda.731 | -1.448 | -3.364 |
| ZINC000003831404 | fda.30 | -1.446 | -3.723 |
| ZINC000003831404 | fda.31 | -1.446 | -3.723 |
| ZINC000008214692 | fda.1111 | -1.433 | -4.043 |
| ZINC000022443609 | fda.1847 | -1.432 | -3.487 |
| ZINC000003917708 | fda.1886 | -1.427 | -3.267 |
| ZINC000100022637 | fda.1292 | -1.426 | -2.358 |
| ZINC000100022637 | fda.1293 | -1.426 | -2.358 |
| ZINC000100016058 | fda.2078 | -1.426 | -2.358 |
| ZINC000100016058 | fda.2079 | -1.426 | -2.358 |
| ZINC000022443609 | fda.1847 | -1.42 | -2.685 |
| ZINC000016052277 | fda.1897 | -1.414 | -3.462 |
| ZINC000014879992 | fda.733 | -1.405 | -3.609 |
| ZINC000072267023 | fda.1703 | -1.397 | -4.081 |
| ZINC000072267023 | fda.1704 | -1.397 | -4.081 |
| ZINC000000001408 | fda.1560 | -1.396 | -3.776 |
| ZINC000018099446 | fda.720 | -1.381 | -3.744 |
| ZINC000100019007 | fda.1312 | -1.381 | -3.744 |
| ZINC000011680943 | fda.471 | -1.378 | -2.511 |
| ZINC000022443609 | fda.1847 | -1.375 | -3.84 |
| ZINC000019364225 | fda.1336 | -1.367 | -1.454 |
| ZINC000019364225 | fda.1337 | -1.367 | -1.454 |
| ZINC000001530303 | fda.261 | -1.359 | -1.359 |
| ZINC000001530968 | fda.1521 | -1.357 | -3.88 |
| ZINC000003812306 | fda.2065 | -1.35 | -2.224 |
| ZINC000008101109 | fda.423 | -1.344 | -4.031 |
| ZINC000008101109 | fda.424 | -1.344 | -4.031 |
| ZINC000008101109 | fda.425 | -1.344 | -4.031 |
| ZINC000001530886 | fda.404 | -1.333 | -3.205 |
| ZINC000001530601 | fda.923 | -1.326 | -3.339 |
| ZINC000016052277 | fda.1897 | -1.318 | -3.594 |
| ZINC000072267023 | fda.1703 | -1.315 | -3.999 |
| ZINC000072267023 | fda.1704 | -1.315 | -3.999 |
| ZINC000003925861 | fda.1778 | -1.311 | -3.656 |
| ZINC000011681563 | fda.472 | -1.309 | -3.201 |
| ZINC000011681563 | fda.473 | -1.309 | -3.201 |
| ZINC000026011099 | fda.40 | -1.304 | -2.342 |
| ZINC000003917708 | fda.1886 | -1.299 | -3.139 |
| ZINC000001530968 | fda.1520 | -1.295 | -3.817 |
| ZINC000019419017 | fda.770 | -1.291 | -2.107 |
| ZINC000245204949 | fda.335 | -1.286 | -3.733 |
| ZINC000005764759 | fda.1331 | -1.286 | -3.733 |
| ZINC000029571072 | fda.2033 | -1.28 | -3.053 |
| ZINC000049637509 | fda.2042 | -1.28 | -3.053 |
| ZINC000003943279 | fda.1420 | -1.271 | -3.632 |
| ZINC000043195697 | fda.1925 | -1.269 | -3.701 |
| ZINC000043195697 | fda.1926 | -1.269 | -3.701 |
| ZINC000043195697 | fda.1927 | -1.269 | -3.701 |
| ZINC000002522669 | fda.910 | -1.263 | -3.371 |
| ZINC000002522669 | fda.911 | -1.263 | -3.371 |
| ZINC000068204830 | fda.215 | -1.254 | -2.057 |
| ZINC000000000196 | fda.447 | -1.248 | -3.272 |
| ZINC000000000196 | fda.448 | -1.248 | -3.272 |
| ZINC000000155531 | fda.917 | -1.248 | -3.272 |
| ZINC000000155531 | fda.918 | -1.248 | -3.272 |
| ZINC000002019953 | fda.903 | -1.242 | -1.292 |
| ZINC000002019954 | fda.937 | -1.242 | -1.292 |
| ZINC000043195697 | fda.1925 | -1.225 | -3.657 |
| ZINC000043195697 | fda.1926 | -1.225 | -3.657 |
| ZINC000043195697 | fda.1927 | -1.225 | -3.657 |
| ZINC000000538386 | fda.578 | -1.225 | -2.859 |
| ZINC000000538386 | fda.579 | -1.225 | -2.859 |
| ZINC000022443609 | fda.1847 | -1.216 | -2.69 |
| ZINC000000538283 | fda.1449 | -1.2 | -1.486 |
[truncated: 21,897 more chars]
